# Supplementary material for: A unified approach to dissecting biphasic responses in cell signaling
Source: eLife. 2023 Dec 6;12:e86520. doi: 10.7554/eLife.86520 (PMC10928513; doi:10.7554/eLife.86520)
Supplement: Supplementary file 1. [file elife-86520-supp1.pdf]

A unified approach to dissecting biphasic responses in cell signalling

Vaidhiswaran Ramesh[1] and J. Krishnan[1,2,*]

[1]Department of Chemical Engineering, Sargent Centre for Process Systems Engineering, Imperial College London, London, SW7 2AZ, UK

[2]Institute for Systems and Synthetic Biology, Imperial College London, South Kensington Campus, London SW7 2AZ, UK

This document is a PDF version of the *Maple* supplementary file that is provided with the main text.

The following table presents the macro-organization of the *Maple* worksheets and results into different folders, and describes the various categories of results presented in them. The page numbers (pertaining to this PDF document) where these specific results/folders can be found in the following table.

Table S1: **Organization of the *Maple* supplementary file**

**Folder**	**Description**	**Pages**
-	*Tabular summary of analytical results*	*2 - 3*
1	Read me file	4
	**Propensity for biphasic responses in commonly observed substrate modification systems**	
1.2	Covalent modification system	6 - 10
1.3	Protein Protein Interaction system	11 - 20
2.1	Double site modification system (common enzymes)	21 - 32
3.2-4	Double site modification systems (different enzyme combinations)	33 - 63
4.2	Two tier cascaded systems	65 - 86
4.3	Coupled systems	87 - 105
	**Biphasic interactions within network motifs**	
5.1	Negative feedback motif (open system)	109 - 110
5.1	Justification for modelling approach to biphasic response interactions	110 - 111
5.2	Integral feedback motif	112 - 115
6.1	Biphasic dose responses in ERK regulation	116 - 126

The table below details the different analytical and semi-analytical results obtained within this study and their location within the PDF version of the *Maple* supplementary file. Where appropriate the table also references the figures in the main text that show or validate such results.

The organization of the results within this table follows the order in which the results are presented, by detailing results pertaining to the simplest models and then we show results in models and systems of increasing complexity. In the table, when a biphasic result is indicated as absent - the behavior is absent irrespective of kinetic regime. Likewise when indicated as present, the behavior is present for all kinetic regimes, and accessible at some total amounts of enzymes and substrates. Where kinetic constraints need to be satisfied to enable (or preclude) the behavior - this is indicated.

Table S2: **Table of contents**

**Model**	**Biphasic in**	**Brief description of analytical result**	**Pg. No.**	**Fig.**
		**Single site modification system**		
Covalent modification system	Substrate	Absence	9 (1.1)	2(A)
	Enzyme	Absence	8 (1.1)	
Protein Protein Interaction	Substrate	Absence	16 (1.2)	2(B)
	Enzyme	Present for all kinetic regimes	17 (1.2)	
		**Double site modification system**		
Common kinase and phosphatase	Substrate	Presence	29 (2.1)	2(C)
	Enzyme	Presence when kinetic constraint (involving catalytic constants) is satisfied	23 (2.1)	
Common kinase and separate phosphatase	Substrate	Presence	38 (3.1)	2(D)
	Enzyme	Absence	36 (3.1)	
Separate kinase and common phosphatase	Substrate	Absence	45 (3.2)	2(E)
	Enzyme (K1)	Absence	47 (3.2)	
	Enzyme (K2)	Presence when kinetic constant (involving catalytic constants) is satisfied	49 (3.2)	
Separate kinase and separate phosphatase	Substrate	Absence	57 (3.3)	2(F)
	Enzyme	Absence	59 (3.3)	
		**Beyond multisite systems**		
Two tier cascaded (Common phosphatase)	Substrate (B)	Presence when kinetic constraint (involving catalytic constants) is satisfied	74 (4.2)	2(G)
	Substrate (A)	Absence	71 (4.2)	
	Enzyme	Absence	67 (4.2)	
Two tier cascaded (Separate phosphatase)	Substrate (both)	Absence	82 (4.2)	2(H)
	Enzyme	Absence	78 (4.2)	
Coupled Covalent modification (Common enzymes)	Substrate	Present *in either (but not both) substrate* for all kinetic regimes	91 (4.3)	2(I)
	Enzyme	Absence	89 (4.3)	
Coupled Covalent modification (Separate kinases)	Substrate	Absence	96 (4.3)	2(J)
	Enzyme	Absence	98 (4.3)	
Coupled Covalent modification (Separate phosphatases)	Substrate	Absence	103 (4.3)	2(K)
	Enzyme	Absence	106 (4.3)	

Continued on next page

Table S2 – continued from previous page

Model	Biphasic in	Brief description of analytical result	Pg. No.	Fig.
		**Biphasic in interaction within network motifs**		
Negative feedback motif (Open system)	Neg. feedback	Absence of multistability	109 (5.1)	-
Negative feedback motif (Closed system)	None	Absence of multistability	-	-
	Neg. feedback	Presence of multistability		3(F)
Justification of modelling approach to biphasic interaction within canonical network motif			110 (5.1)	-
Biphasic interaction in integral feedback control motif - saturating effect, and loss of stability			112 (5.2)	-
		**Concrete pathways**		
ERK regulation	Substrate	Presence	119 (6.1)	4(A,C)
	Enzyme	Can be obtained	123 (6.1)	4(B,C)

A unified approach to dissecting biphasic responses in cell signalling

Vaidhiswaran Ramesh[1], J Krishnan[1,2,*]

Affiliations:
1 - Department of Chemical Engineering, Sargent Centre for Process Systems Engineering, Imperial College London, London, SW7 2AZ, UK
2 - Institute for Systems and Synthetic Biology, Imperial College London, South Kensington Campus, London SW7 2AZ, UK

* - Corresponding author (j.krishnan@ic.ac.uk)

This supplementary material provides detailed model descriptions and proofs for the analytical arguments presented in the main text. The document is organized into many fice folders (and many sub-folders), as described below. A PDF version of the file is also provided for easy accessibility.

Organization of the results

Read me file (this file)
Folder 1: Simple enzymatic networks
Folder 2: Multisite modification (double site ordered phosphorylation model with common enzymes)
Folder 3: Multisite modification - enzyme combinations (double site ordered phosphorylation model with different enzyme combinations acting on each modification site)
Folder 4: Beyond multisite modification (coupled covalent modifications and cascaded enzymatic networks)
Folder 5: Modeling approaches for biphasic responses within canonical network motif interactions
Folder 6: Biphasic responses in Erk Model

Each folder if presented with a Read_Me file that serves as a preamble to indicate the nature of results within and each file is accompanied with detailed instructions on how to run the code in Maple.

Key insight relevant to biphasic dose response

In the following documents, in order to establish the presence or absence of biphasic responses in steady state concentration of a given variable with a dose parameter, we use the mathematical definition of the behavior. This main tenet of the behavior forms an important part of the proofs that follow and is highlighted here.

If a system is capable of exhibiting biphasic response in a variable 'x' as a dose parameter 'p' is changed, then it requires that there exists a feasible steady state of the system satisfying the model description (the system of ODEs and the conservation equation), which also simultaneously satisfies the following mathematical expression

$$\frac{\partial x}{\partial p} = 0$$

This arises from the fact that, for a biphasic behavior to exist, there has to naturally exist a peak concentration of the variable 'x' where the gradient of the steady state curve is zero. The absence of such a point indicates the absence of biphasic response in the system.

An enzymatic network has two natural 'doses' from the perspective of the maximally modified substrate; the total substrate in the system and the total amount of kinase (the enzyme effecting the modifications). Enzymatic networks are capable of exhibiting biphasic responses in the modified form (or the maximally modified form) with change in both doses. Both of these biphasic dose responses are of interest as they belay simple intuitive expectations of the system to behave monotonically with change in dose. These different biphasic responses shown in the substrate are simply labelled as enzyme biphasic or substrate biphasic from here on within this text (refering to scenarios where the enzyme or the substrate is the dose, respectively).

Simple enzymatic network motifs

In this folder we detail analytical results pertaining to simple enzymatic network motifs (covalent modification cycle and protein-protein interaction model), specifically their capacity to exhibit biphasic responses (in steady state dose response). The study of these networks and the results provide an emerging synthesis on the different kinds of biphasic responses that can be seen in signaling systems and the minimal ingredients required to generate or enable such responses.

Covalent modification network
[single modification/demotification of a protein]

In this file we prove that the simple covalent modification network with a kinase and a phosphatase effecting the modifications and demodification (phosphorylation and dephosphorylation) is incapable of presenting any biphasic dose response behavior in the modified substrate form. More specifically, we show the

1. absence of enzyme biphasic response in concentration of Ap with changing total kinase concentration
2. absence of substrate biphasic response in concentration of Ap with changing total substrate concentration

Detailed definitions of these two different classes of biphasic behavior are described in the manuscript (see Read_Me file on page 1). We reiterate that should a biphasic behavior exist in the steady state concentration of the modified substrate form (Ap) with respect to any parameter 'p' (total enzyme amount or the total substrate amount), then there exists steady state concentrations of the variables where the following mathematical expression is necessarily satisfied.

$$\frac{\partial Ap}{\partial p} = 0$$

This takes the form $\frac{\partial Ap}{\partial K_{Total}} = 0$ for enzyme biphasic and $\frac{\partial Ap}{\partial A_{Total}} = 0$ for substrate biphasic response.

In this file, we begin by describing the mathematical model for a covalent modification network. Then by solving the system of ODEs at steady state, we obtain equations relating the steady state concentrations of the different variables and using these we proceed to show the absence of both biphasic dose response behaviors (subsection 1 and 2).

Covalent modification network: We first describe the mathematical model of a covalent modification network.

We initialize the Maple file with the *restart* command and load the relevant libraries of inbuilt Maple functions (*LinearAlgebra, VectorCalculus, Student[LinearAlgebra]*)

$restart : with(LinearAlgebra) : with(Student[LinearAlgebra]) : with(VectorCalculus) :$

The system is modeled as a set of ODEs using the kinetic nomenclature described in the main text (Models and Methods section). Here dA represents d[A]/dt and similarly in the case of the other variables. At steady state thus, the right hand sides of each of these expressions will be equal to zero.

$$dA := k_2 \cdot ApP - k_{b1} \cdot A \cdot K + k_{ub1} \cdot AK :$$
$$dAp := k_1 \cdot AK + k_{ub2} \cdot ApP - k_{b2} \cdot Ap \cdot P :$$

$$dAK := k_{b1} \cdot A \cdot K - \left(k_{ub1} + k_1\right) \cdot AK :$$
$$dApP := k_{b2} \cdot Ap \cdot P - \left(k_{ub2} + k_2\right) \cdot ApP :$$

$$dK := -k_{b1} \cdot A \cdot K + \left(k_{ub1} + k_1\right) \cdot AK :$$
$$dP := -k_{b2} \cdot Ap \cdot P + \left(k_{ub2} + k_2\right) \cdot ApP :$$

The model is also associated with conservation conditions which are described below. Here we store the conservation expressions as ACon, PCon and KCon for the substrate and the respective enzymes. Each of these expressions is always equal to zero (both in the transient and at steady state).

$$ACon := A + Ap + ApP + AK - A_{Total} :$$
$$KCon := K + AK - K_{Total} :$$
$$PCon := P + ApP - P_{Total} :$$

Now we begin solving the system of equations to obtain equations relating the steady state concentrations of the variables. Primarily to obtain relationships between steady state concentration of variables as a function of concentrations of Ap and the free kinase concentration K. For this purpose we use an inbuilt *Maple* command *solve* as shown with the example below.

$$AK := solve(dAK, AK) = \frac{k_{b1}\, A\, K}{k_{ub1} + k_1}$$
$$assign(solve(\{dApP, dA\}, \{ApP, A\})) :$$

Once this is done, we again solve for the steady state of phosphatase and the substrate involved in the covalent modification cycle using the conservation expression for the enzyme (PCon),

$$P := solve(PCon, P) :$$

Now having solved for the steady state of the system in terms of Ap and K, the only two equations that remain (which define the steady state of the system) are the conservation equations for the kinase and the substrate (KCon and PCon) respectively (see below).

$ACon$

$$\frac{Ap\, P_{Total}\, k_2\, k_{b2}\left(k_{ub1} + k_1\right)}{\left(k_{b2}\, Ap + k_2 + k_{ub2}\right) K\, k_1\, k_{b1}} + Ap + \frac{k_{b2}\, Ap\, P_{Total}}{k_{b2}\, Ap + k_2 + k_{ub2}} + \frac{Ap\, P_{Total}\, k_2\, k_{b2}}{\left(k_{b2}\, Ap + k_2 + k_{ub2}\right) k_1} - A_{Total} \qquad \textbf{(1)}$$

$KCon$

$$K+\frac{Ap\,P_{Total}\,k_2\,k_{b2}}{\left(k_{b2}\,Ap+k_2+k_{ub2}\right)\,k_1}-K_{Total} \quad \textbf{(2)}$$

1. Enzyme biphasic

Now in order to show the absence of enzyme biphasic response in Ap with total enzyme concentration, we proceed with a proof by contradiction. As mentioned earlier, for enzyme biphasic in Ap to exist, for some steady state of the system, $\frac{\partial Ap}{\partial K_{Total}}=0$ must be satisfied. Thus, we begin with the assumption that such a biphasic exists, satisfying the condition.

Now, differentiating ACon with respect to K_{Total} provides the following

$$\frac{dACon}{dK_{Total}}=0=\frac{\partial ACon}{\partial K}\cdot\frac{\partial K}{\partial K_{Total}}+\frac{\partial ACon}{\partial Ap}\cdot\frac{\partial Ap}{\partial K_{Total}}$$

$$\frac{dKCon}{dK_{Total}}=0=\frac{\partial KCon}{\partial K_{Total}}+\frac{\partial KCon}{\partial K}\cdot\frac{\partial K}{\partial K_{Total}}+\frac{\partial KCon}{\partial Ap}\cdot\frac{\partial Ap}{\partial K_{Total}}$$

With the assumption that there exists a biphasic behavior in the substrate with changing total substrate amounts, these equations simplify as shown below

$$0=\frac{\partial ACon}{\partial K}\cdot\frac{\partial K}{\partial K_{Total}}$$

$$1=\frac{\partial K}{\partial K_{Total}}$$

Note

This reduction was possible since $\frac{\partial KCon}{\partial Ap}$ and $\frac{\partial ACon}{\partial Ap}$ are finite and have non-zero denominators (as shown below), allowing the product with $\frac{\partial ACon}{\partial Ap}$ to be zero.

$$simplify(diff(KCon, Ap))=\frac{P_{Total}\,k_2\,k_{b2}\,\left(k_{ub2}+k_2\right)}{\left(k_{b2}\,Ap+k_2+k_{ub2}\right)^2\,k_1}$$

$$simplify(diff(ACon, Ap))=$$

$$\frac{1}{\left(k_{b2}\,Ap+k_2+k_{ub2}\right)^2\,K\,k_1\,k_{b1}}\left(Ap^2\,K\,k_1\,k_{b1}\,k_{b2}^{\,2}+2\left(K\left(\left(Ap+\frac{P_{Total}}{2}\right)k_1+\frac{k_2\,P_{Total}}{2}\right)k_{b1}+\frac{P_{Total}\,k_2\,\left(k_{ub1}+k_1\right)}{2}\right)\left(k_{ub2}+k_2\right)k_{b2}+K\,k_1\,k_{b1}\,\left(k_{ub2}+k_2\right)^2\right)$$

The above equation indicates that $\frac{\partial K}{\partial K_{Total}}$ is equal to 1. From this, we can observe that $\frac{\partial ACon}{\partial K}$ must be zero to satisfy the first expression (obtained from differentiating ACon). However as seen below, we can see that this is not possible for any feasible steady state of the system.

$$simplify(diff(ACon, K)) = -\frac{Ap\, P_{Total}\, k_2\, k_{b2}\, (k_{ub1} + k_1)}{(k_{b2}\, Ap + k_2 + k_{ub2})\, K^2\, k_1\, k_{b1}}$$

This is a contradiction and thus enzyme biphasic behavior in Ap with total enzyme concentration (K_{Total}) is not possible.

2. Substrate biphasic

Now in order to show the absence of substrate biphasic response in Ap with total substrate concentration, we proceed with a proof by contradiction. As mentioned earlier, for substrate biphasic in Ap to exist, for some steady state of the system, $\frac{\partial Ap}{\partial A_{Total}} = 0$ must be satisfied. Thus we begin with the assumption that such a biphasic exists, satisfying the condition.

This implies that differentiating ACon with respect to A_{Total} provides the following

$$\frac{dACon}{dA_{Total}} = 0 = \frac{\partial ACon}{\partial A_{Total}} + \frac{\partial ACon}{\partial K} \cdot \frac{\partial K}{\partial A_{Total}} + \frac{\partial ACon}{\partial Ap} \cdot \frac{\partial Ap}{\partial A_{Total}}$$

$$\frac{dKCon}{dA_{Total}} = 0 = \frac{\partial KCon}{\partial K} \cdot \frac{\partial K}{\partial A_{Total}} + \frac{\partial KCon}{\partial Ap} \cdot \frac{\partial Ap}{\partial A_{Total}}$$

With the assumption that there exists a biphasic behavior in the substrate with changing total substrate amounts, these equations simplify as shown below

$$1 = \frac{\partial ACon}{\partial K} \cdot \frac{\partial K}{\partial A_{Total}}$$

$$0 = \frac{\partial K}{\partial A_{Total}}$$

Note

This reduction was possible since $\frac{\partial KCon}{\partial Ap}$ and $\frac{\partial ACon}{\partial Ap}$ are finite and have non-zero denominators (as shown below), allowing the product with $\frac{\partial ACon}{\partial Ap}$ to be zero.

$$simplify(diff(KCon, Ap)) = \frac{P_{Total}\, k_2\, k_{b2}\, (k_{ub2} + k_2)}{(k_{b2}\, Ap + k_2 + k_{ub2})^2\, k_1}$$

$$simplify(diff(ACon, Ap)) = \frac{1}{(k_{b2}\, Ap + k_2 + k_{ub2})^2\, K\, k_1\, k_{b1}} \Bigg(Ap^2\, K\, k_1\, k_{b1}\, k_{b2}^2 + 2 \bigg(K \bigg(\Big(Ap + \frac{P_{Total}}{2} \Big) k_1 + \frac{k_2\, P_{Total}}{2} \bigg) k_{b1} + \frac{P_{Total}\, k_2\, (k_{ub1} + k_1)}{2} \bigg) (k_{ub2} + k_2)\, k_{b2} + K\, k_1\, k_{b1}\, (k_{ub2} + k_2)^2 \Bigg)$$

The first expression indicates that

$\frac{\partial K}{\partial A_{Total}}$ cannot be equal to 0 (since $\frac{\partial ACon}{\partial K}$ itself is always positive, as shown below). However the second expression (obtained through differentiating KCon) contradicts this.

$$simplify(diff(ACon, K)) = -\frac{Ap\, P_{Total}\, k_2\, k_{b2}\, (k_{ub1} + k_1)}{(k_{b2}\, Ap + k_2 + k_{ub2})\, K^2\, k_1\, k_{b1}}$$

This is a contradiction and thus substrate biphasic behavior in Ap with total substrate concentration (A_{Total}) is not possible.

Protein-protein interaction model (PPI)
[adopted from Kholodenko et al 2015., Interface.]

In this manuscript we analytically show the propensity of the PPI model (first described by Kholodenko et al., 2015) to exhibit biphasic behavior in the substrate form as the total enzyme concentration changes (enzyme biphasic).

This has been computationally established in the manuscript by Kholodenko et al., 2015, and here we show that this is analytically guaranteed in for the entire range of kinetic parameters due to the network structure of the system.

We build on the computational result of Kholodenko et al., 2015 to show that

1. The PPI system does accommodate a biphasic behavior in the modified substrate form for the entire range of kinetic parameters due to network structure.
2. We show that the full model (described using mass action kinetics without any assumptions on enzymatic regimes such as the ones assumed in Kholodenko et al., 2015) is also capable of enzyme biphasic response for any kinetic parameter choice while being incapable of substrate biphasic response in the modified substrate form irrespective of kinetic parameters.

Protein-protein interaction model [model from Kholodenko et al., 2015]

We initialize the Maple file with the *restart* command and load the relevant libraries of inbuilt Maple functions (*LinearAlgebra, VectorCalculus, Student[LinearAlgebra]*)

$restart : with(LinearAlgebra) : with(Student[LinearAlgebra]) : with(VectorCalculus) :$

The system is modeled as a set of ODEs using the kinetic nomenclature as described in Kholodenko et al. , 2015. Here dA represents d[A]/dt and similarly in the case of the other variables. At steady state thus, the right hand sides of each of these expressions will be equal to zero.

$$dA := -k_{b1} \cdot A \cdot B + k_{ub1} \cdot AB :$$

$$dB := -k_{b1} \cdot A \cdot B + k_{ub1} \cdot AB - \frac{k_2 \cdot B}{K_2 + B} + \frac{k_3 \cdot A \cdot Bs}{K_3 + Bs} :$$

$$dBs := \frac{k_2 \cdot B}{K_2 + B} - \frac{k_3 \cdot A \cdot Bs}{K_3 + Bs} :$$

$$dAB := k_{b1} \cdot A \cdot B - k_{ub1} \cdot AB :$$

The model is also associated with conservation conditions which are described below. Here we store the conservation expressions as ACon, BCon. Each of these expressions is always equal to zero (both in the transient and at steady state).

$$ACon := A_{Total} - A - AB :$$

$$BCon := B_{Total} - AB - B - Bs :$$

Now we begin by solving the system of equations to obtain correlations between the steady state concentrations of the variables. Primarily to obtain steady state correlations of variables as a function of concentrations of B and the Bs. For this purpose we use an inbuilt *Maple* command *solve* as shown with the example below.

$$AB := simplify(solve(dAB, AB)) = \frac{k_{b1}\, A\, B}{k_{ub1}}$$

$$A := simplify((solve(dB, A))) :$$

$$A = \frac{k_2\, B\, (K_3 + Bs)}{(K_2 + B)\, k_3\, Bs}$$

$$AB = \frac{k_{b1}\, k_2\, B^2\, (K_3 + Bs)}{(K_2 + B)\, k_3\, Bs\, k_{ub1}}$$

Note for positive concentrations of B and Bs, A and AB are positive as well. Thus this results in us solving all ODEs and conservation equations describing the system, except for the conservation expressions ACon and BCon.

$ACon$

$$A_{Total} - \frac{k_2\, B\, (K_3 + Bs)}{(K_2 + B)\, k_3\, Bs} - \frac{k_{b1}\, k_2\, B^2\, (K_3 + Bs)}{(K_2 + B)\, k_3\, Bs\, k_{ub1}} \quad \textbf{(1.1)}$$

$BCon$

$$B_{Total} - \frac{k_{b1}\, k_2\, B^2\, (K_3 + Bs)}{(K_2 + B)\, k_3\, Bs\, k_{ub1}} - B - Bs \tag{1.2}$$

If we differentiate both these with respect to the total enzyme concentration (A_{Total}) in the system, we get

$$\frac{dACon}{dA_{Total}} = 0 = \frac{\partial ACon}{\partial A_{Total}} + \frac{\partial ACon}{\partial B} \cdot \frac{\partial B}{\partial A_{Total}} + \frac{\partial ACon}{\partial Bs} \cdot \frac{\partial Bs}{\partial A_{Total}}$$
$$\frac{dBCon}{dA_{Total}} = 0 = \frac{\partial BCon}{\partial B} \cdot \frac{\partial B}{\partial A_{Total}} + \frac{\partial BCon}{\partial Bs} \cdot \frac{\partial Bs}{\partial A_{Total}}$$

Now, in order for there to exist a biphasic response in B with total enzyme concentration, $\frac{\partial B}{\partial A_{Total}}$ must equal zero (the gradient at the peak concentration achieved by B in the dose response curve is zero). Thus, we can simplify these expressions further as,

Note

This simplification was possible since $\frac{\partial ACon}{\partial B}$ and $\frac{\partial BCon}{\partial B}$ are finite and have non-zero denominators (see below)

$$simplify(diff(ACon, B)) = -\frac{((2\, k_{b1}\, B + k_{ub1})\, K_2 + k_{b1}\, B^2)\, k_2\, (K_3 + Bs)}{(K_2 + B)^2\, k_3\, Bs\, k_{ub1}}$$

$$simplify(diff(BCon, B)) = \frac{1}{(K_2 + B)^2\, k_3\, Bs\, k_{ub1}} \Big(((-k_2\, k_{b1} - k_3\, k_{ub1})\, Bs - K_3\, k_2\, k_{b1})\, B^2 - 2\, K_2\, ((k_2\, k_{b1} + k_3\, k_{ub1})\, Bs + K_3\, k_2\, k_{b1})\, B - Bs\, K_2^2\, k_3\, k_{ub1} \Big)$$

$$1 = \frac{\partial ACon}{\partial Bs} \cdot \frac{\partial Bs}{\partial A_{Total}}$$
$$0 = \frac{\partial BCon}{\partial Bs} \cdot \frac{\partial Bs}{\partial A_{Total}}$$

Now since, $\frac{\partial ACon}{\partial Bs}$ is always positive (see below), $\frac{\partial Bs}{\partial A_{Total}}$ has to be non-zero. Which implies, in order to achieve a biphasic response in B with total enzyme concentration, $\frac{\partial BCon}{\partial Bs}$ must equal zero. This is in fact a sufficiency condition as well, since for any concentration of B satisfying such a condition, we can suitably find a total substrate and enzyme concentration using ACon and BCon at which they are steady state concentrations as well.

$$simplify(diff(BCon, Bs)) = \frac{-(K_2 + B)\, k_3\, Bs^2\, k_{ub1} + k_{b1}\, k_2\, B^2\, K_3}{(K_2 + B)\, k_3\, Bs^2\, k_{ub1}}$$

Solving this for the concentration of Bs, we obtain the following correlation between the concentration of Bs and B, denoting the point at a biphasic peak achieved in the dose response curve of B with A_{Total}.

$Bs := simplify(solve(diff(BCon, Bs), Bs)[1])$

$$Bs := \frac{\sqrt{k_3\, k_{ub1}\, (K_2 + B)\, K_3\, k_2\, k_{b1}}\; B}{k_3\, k_{ub1}\, (K_2 + B)} \qquad \textbf{(1.3)}$$

Now thus, all that remains is to find a suitable total substrate and enzyme amount where this peak can exist. This however is trivial to show, as for every choice of concentration of B, there exists a unique Bs, A, AB (each identified using the relevant correlations between the steady state species concentrations)

$ACon =$

$$A_{Total} - \frac{k_2\, k_{ub1}\left(K_3 + \frac{\sqrt{k_3\, k_{ub1}\,(K_2+B)\, K_3\, k_2\, k_{b1}}\; B}{k_3\, k_{ub1}\,(K_2+B)}\right)}{\sqrt{k_3\, k_{ub1}\,(K_2+B)\, K_3\, k_2\, k_{b1}}} - \frac{k_{b1}\, k_2\left(K_3 + \frac{\sqrt{k_3\, k_{ub1}\,(K_2+B)\, K_3\, k_2\, k_{b1}}\; B}{k_3\, k_{ub1}\,(K_2+B)}\right) B}{\sqrt{k_3\, k_{ub1}\,(K_2+B)\, K_3\, k_2\, k_{b1}}}$$

$BCon =$

$$B_{Total} - \frac{k_{b1}\, k_2\left(K_3 + \frac{\sqrt{k_3\, k_{ub1}\,(K_2+B)\, K_3\, k_2\, k_{b1}}\; B}{k_3\, k_{ub1}\,(K_2+B)}\right) B}{\sqrt{k_3\, k_{ub1}\,(K_2+B)\, K_3\, k_2\, k_{b1}}} - B - \frac{\sqrt{k_3\, k_{ub1}\,(K_2+B)\, K_3\, k_2\, k_{b1}}\; B}{k_3\, k_{ub1}\,(K_2+B)}$$

And thus implies, that for every choice of underlying kinetics and for every feasible concentration of B at the biphasic peak, there exits total amount of substrate and enzyme, where it is possible to observe a enzyme biphasic response with total enzyme concentration (i.e. the behavior to guaranteed to occur for any given underlying kinetics, provided there is flexibility on the total concentrations of the substrate).

Below, we illustrate a parameter set where the biphasic behavior is analytically predicted

$k_{b1} := 0.000662316 : k_{ub1} := 0.000701878 : k_2 := 53.6473 : K_2 := 1880.36 : k_3 := 2.98182 : K_3 := 11.0657 :$

$B := 19.428458 :$

$simplify(AB) = 9.482758847$
$simplify(A) = 0.5172407784$
$simplify(Bs) = 6.109563569$

$A_{Total} := solve(ACon) = 9.999999625$
$B_{Total} := solve(BCon) = 35.02078042$

Thus in this subsection, we have shown how the PPI model illustrated by Kholodenko et al., 2015, which is a simple variation of a covalent modification system involving just an additional inactive complex formation with the enzyme (albeit with specific regimes of enzyme action - see next subsection) is capable of exhibiting enzyme biphasic dose response robustly in the entirety of intrinsic kinetic parameter space.

Protein-protein interaction model [Full system]

Note: In this subsection, we (re)model PPI network using mass action kinetic description. This kinetic description for all reactions assumes no limitations on the regime of enzyme action (unlike the PPI model described in Kholodenko et al., 2015 and used in the previous subsection) and thus is a more accurate representation of the variation of the covalent modification system it is. Using this model, we show that the PPI network is incapable of exhibiting substrate biphasic response, while (as shown in the previous subsection) it is capable of exhibiting robust enzyme biphasic responses in the modified substrate form.

We initialize the Maple file with the *restart* command and load the relevant libraries of inbuilt Maple functions (*LinearAlgebra, VectorCalculus, Student[LinearAlgebra]*)

$restart : with(LinearAlgebra) : with(Student[LinearAlgebra]) : with(VectorCalculus) :$

The system is modeled as a set of ODEs using the kinetic nomenclature described in the main text (Models and Methods section). Here dA represents d[A]/dt and similarly in the case of the other variables. At steady state thus, the right hand sides of each of these expressions will be equal to zero.

$dA := k_4 \cdot ApP + k_{ub1} \cdot AK - k_{b1} \cdot A \cdot K :$
$dAp := k_1 \cdot AK + k_{ub2} \cdot (ApK) + k_{ub4} \cdot (ApP) - k_{b2} \cdot (Ap) \cdot (K) - k_{b4} \cdot Ap \cdot P :$

$dAK := k_{b1} \cdot A \cdot K - (k_{ub1} + k_1) \cdot AK :$
$dApK := k_{b2} \cdot Ap \cdot K - (k_{ub2}) \cdot ApK :$
$dApP := k_{b4} \cdot Ap \cdot P - (k_{ub4} + k_4) \cdot ApP :$

$dK := -k_{b1}\, A\, K + (k_{ub1} + k_1)\, AK - k_{b2}\, Ap\, K + (k_{ub2})\, ApK :$
$dP := -k_{b4}\, Ap\, P + (k_{ub4} + k_4)\, ApP :$

The model is also associated with conservation conditions which are described below. Here we store the conservation expressions as ACon, KCon and PCon for the substrate and the respective enzymes. Each of these expressions is always equal to zero (both in the transient and at steady state).

$ACon := A_{Total} - A - ApP - ApK - AK - Ap :$
$KCon := K_{Total} - AK - K - ApK :$
$PCon := P_{Total} - P - ApP :$

Now we begin by solving the system of equations to obtain correlations between the steady state concentrations of the variables. Primarily to obtain steady state correlations of variables as a function of concentrations of Ap and the ratio ϵ (defined below). For this purpose we use an inbuilt *Maple* command *solve* as shown with the example below.

$$AK := solve(dAK, AK) = \frac{k_{b1}\, A\, K}{k_{ub1} + k_1}$$

$assign(solve(\{dA, dApK, dApP\}, \{A, ApK, ApP\}))$

We now introduce a new ratio, $\epsilon = \dfrac{K}{P}$ (ratio of the free enzymes), Simultaneously we introduce the following parameters (c_1, c_2, c_3, and c_4). This is done for the sake of brevity and easy tractability of the expressions obtained.

$K := \epsilon \cdot P :$
$k_{b1} := c_1 \cdot (k_1 + k_{ub1}) : k_{b2} := c_2 \cdot (k_{ub2}) : k_{b4} := c_4 \cdot (k_4 + k_{ub4}) :$

After this simplification, we solve the conservation equation of the phosphatase for the steady state concentration of the enzyme as shown below,

$P := solve(simplify(PCon), P)$:

Thus this results in us solving all ODEs and conservation equations describing the system, except for the conservation expressions ACon and KCon.

$$A = \frac{Ap\, k_4\, c_4}{\epsilon\, k_1\, c_1}$$
$$AK = \frac{Ap\, k_4\, c_4\, P_{Total}}{k_1 \left(Ap\, c_4 + 1\right)}$$
$$ApK = \frac{c_2\, Ap\, \epsilon\, P_{Total}}{Ap\, c_4 + 1}$$
$$ApP = \frac{c_4\, Ap\, P_{Total}}{Ap\, c_4 + 1}$$

The steady state of the system is now defined by feasible solutions (of Ap and ϵ) of the expressions ACon and BCon (Note for positive concentrations of Ap and ϵ, the remaining species concentrations are positive as well).

$ACon$

$$A_{Total} - \frac{Ap\, k_4\, c_4}{\epsilon\, k_1\, c_1} - \frac{c_4\, Ap\, P_{Total}}{Ap\, c_4 + 1} - \frac{c_2\, Ap\, \epsilon\, P_{Total}}{Ap\, c_4 + 1} - \frac{Ap\, k_4\, c_4\, P_{Total}}{k_1 \left(Ap\, c_4 + 1\right)} - Ap \tag{2.1}$$

$KCon$

$$K_{Total} - \frac{Ap\, k_4\, c_4\, P_{Total}}{k_1 \left(Ap\, c_4 + 1\right)} - \frac{\epsilon\, P_{Total}}{Ap\, c_4 + 1} - \frac{c_2\, Ap\, \epsilon\, P_{Total}}{Ap\, c_4 + 1} \tag{2.2}$$

Proof of absence of substrate biphasic response

Now, to show the absence of a substrate biphasic response in Ap with total substrate concentration, we proceed with a proof by contradiction. In order for there to exist a substrate biphasic, the following must be necessarily true at some steady state in the system (the gradient at the peak concentration achieved by Ap in the dose response curve is zero)

$$\frac{\partial Ap}{\partial A_{Total}} = 0$$

If we differentiate both conservation expressions with respect to the total enzyme concentration (A_{Total}) in the system, we get

$$\frac{dACon}{dA_{Total}} = 0 = \frac{\partial ACon}{\partial A_{Total}} + \frac{\partial ACon}{\partial Ap} \cdot \frac{\partial Ap}{\partial A_{Total}} + \frac{\partial ACon}{\partial \epsilon} \cdot \frac{\partial \epsilon}{\partial A_{Total}}$$
$$\frac{dKCon}{dA_{Total}} = 0 = \frac{\partial KCon}{\partial Ap} \cdot \frac{\partial Ap}{\partial A_{Total}} + \frac{\partial KCon}{\partial \epsilon} \cdot \frac{\partial \epsilon}{\partial A_{Total}}$$

Since we begin with the assumption that there exists such a biphasic , we can simplify these expressions further as

$$1 = \frac{\partial ACon}{\partial \epsilon} \cdot \frac{\partial \epsilon}{\partial A_{Total}}$$

$$0 = \frac{\partial KCon}{\partial \epsilon} \cdot \frac{\partial \epsilon}{\partial A_{Total}}$$

Note

This simplification was possible since $\frac{\partial ACon}{\partial Ap}$ and $\frac{\partial KCon}{\partial Ap}$ are finite and have non-zero denominators (see below)

$simplify(diff(ACon, Ap)) =$

$$\frac{1}{\epsilon k_1 c_1 (Ap\, c_4 + 1)^2} \left(-Ap^2 c_4^{\,3} k_4 - Ap\,(Ap\, c_1 \epsilon k_1 + 2 k_4)\, c_4^{\,2} + \left(-2 c_1 \left(\left(\left(Ap + \frac{P_{Total}}{2}\right) k_1 + \frac{k_4 P_{Total}}{2}\right) \epsilon - k_4\right) c_4 - c_1 k_1 \epsilon\,(c_2 \epsilon P_{Total} + 1)\right)\right)$$

$$simplify(diff(KCon, Ap)) = -\frac{((-\epsilon k_1 + k_4)\, c_4 + c_2 \epsilon k_1)\, P_{Total}}{k_1 (Ap\, c_4 + 1)^2}$$

Now since, $\frac{\partial KCon}{\partial \epsilon}$ is always negative (see below), $\frac{\partial \epsilon}{\partial A_{Total}}$ $\frac{\partial ACon}{\partial \epsilon}$ are finite and have non-zero denominators.

$$simplify(diff(KCon, \text{epsilon})) = -\frac{P_{Total}\,(Ap\, c_2 + 1)}{Ap\, c_4 + 1}$$

$$simplify(diff(ACon, \text{epsilon})) = \frac{Ap\,(-c_2 P_{Total} \epsilon^2 k_1 c_1 + Ap\, k_4 c_4^{\,2} + k_4 c_4)}{\epsilon^2 k_1 c_1 (Ap\, c_4 + 1)}$$

This is a contradiction and thus substrate biphasic behavior in Ap with total substrate concentration (A_{Total}) is not possible.

Enzyme biphasic

The full model description of the PPI system is capable of exhibiting enzyme biphasic response. In this subsection, we prove the existence of the behavior irrespective of kinetic parameter choice.

We begin with the assumption that there exists an enzyme biphasic response in Ap to total kinase concentration. We know that in order for there to exist an enzyme biphasic, the following must be necessarily true at some steady state in the system (the gradient at the peak concentration achieved by Ap in the dose response curve is zero)

$$\frac{\partial Ap}{\partial K_{Total}} = 0$$

If we differentiate both conservation expressions with respect to the total enzyme concentration (A

$_{Total}$) in the system, we get

$$\frac{dACon}{dK_{Total}} = 0 = \frac{\partial ACon}{\partial Ap} \cdot \frac{\partial Ap}{\partial K_{Total}} + \frac{\partial ACon}{\partial \epsilon} \cdot \frac{\partial \epsilon}{\partial K_{Total}}$$
$$\frac{dKCon}{dK_{Total}} = 0 = \frac{\partial KCon}{\partial K_{Total}} + \frac{\partial KCon}{\partial Ap} \cdot \frac{\partial Ap}{\partial K_{Total}} + \frac{\partial KCon}{\partial \epsilon} \cdot \frac{\partial \epsilon}{\partial K_{Total}}$$

Since we begin with the assumption that there exists such a biphasic, we can simplify these expressions further as

$$0 = \frac{\partial ACon}{\partial \epsilon} \cdot \frac{\partial \epsilon}{\partial K_{Total}}$$
$$1 = \frac{\partial KCon}{\partial \epsilon} \cdot \frac{\partial \epsilon}{\partial K_{Total}}$$

Note

This simplification was possible since $\frac{\partial ACon}{\partial Ap}$ and $\frac{\partial BCon}{\partial Ap}$ are finite and have non-zero denominators (see below)

$$simplify(diff(ACon, Ap)) = \frac{1}{\epsilon k_1 c_1 (Ap\, c_4 + 1)^2} \left(-Ap^2 c_4^3 k_4 - Ap\, (Ap\, c_1 \epsilon k_1 + 2 k_4) c_4^2 + \left(-2 \left(\left(Ap + \frac{P_{Total}}{2} \right) k_1 + \frac{k_4 P_{Total}}{2} \right) c_1 \epsilon - k_4 \right) c_4 - c_1 k_1 \epsilon (c_2 \epsilon P_{Total} + 1) \right)$$

$$simplify(diff(KCon, Ap))$$
$$-\frac{((-\epsilon k_1 + k_4) c_4 + c_2 \epsilon k_1) P_{Total}}{k_1 (Ap\, c_4 + 1)^2} \quad \textbf{(2.2.1.1)}$$

Now since, $\frac{\partial KCon}{\partial \epsilon}$ is always negative (see below), $\frac{\partial \epsilon}{\partial K_{Total}}$ has to be non-zero. Which implies, in order to achieve a biphasic response in Ap with total enzyme concentration, $\frac{\partial ACon}{\partial \epsilon}$ must equal zero. This is in fact a sufficiency condition as well, since for any concentration of ϵ and Ap satisfying such a condition, we can suitably find a total substrate and enzyme concentration using ACon and KCon at which they are steady state concentrations as well.

$$simplify(diff(KCon, \epsilon)) = -\frac{P_{Total} (Ap\, c_2 + 1)}{Ap\, c_4 + 1}$$

Solving $\frac{\partial ACon}{\partial \epsilon} = 0$ for the concentration of Ap, we obtain the following correlation between the concentration of Ap and ϵ, denoting the point at a biphasic peak achieved in the dose response curve of Ap with K_{Total}.

$$Ap := simplify(solve(diff(ACon, \epsilon), Ap)[2]) = \frac{c_1\, c_2\, \epsilon^2\, k_1\, P_{Total} - c_4\, k_4}{c_4^2\, k_4}$$

Now thus, all that remains is to find a suitable total substrate and enzyme amount where this peak can exist. This however is trivial to show, as for every choice of concentration of ϵ (when Ap is positive here), there exists a unique AK, ApK, ApP (each identified using the relevant correlations between the steady state species concentrations)

$$\epsilon \tag{2.2.1}$$

$$ACon = A_{Total} - \frac{c_2\, P_{Total}\, \epsilon^2\, k_1\, c_1 - k_4\, c_4}{c_4\, \epsilon\, k_1\, c_1} - \frac{\left(c_2\, P_{Total}\, \epsilon^2\, k_1\, c_1 - k_4\, c_4\right) P_{Total}}{c_4\, k_4 \left(\frac{c_2\, P_{Total}\, \epsilon^2\, k_1\, c_1 - k_4\, c_4}{c_4\, k_4} + 1\right)} - \frac{c_2 \left(c_2\, P_{Total}\, \epsilon^2\, k_1\, c_1 - k_4\, c_4\right) \epsilon\, P_{Total}}{c_4^2\, k_4 \left(\frac{c_2\, P_{Total}\, \epsilon^2\, k_1\, c_1 - k_4\, c_4}{c_4\, k_4} + 1\right)} - \frac{\left(c_2\, P_{Total}\, \epsilon^2\, k_1\, c_1 - k_4\, c_4\right) P_{Total}}{c_4\, k_1 \left(\frac{c_2\, P_{Total}\, \epsilon^2\, k_1\, c_1 - k_4\, c_4}{c_4\, k_4} + 1\right)} - \frac{c_2\, P_{Total}\, \epsilon^2\, k_1\, c_1 - k_4\, c_4}{c_4^2\, k_4}$$

$$KCon = K_{Total} - \frac{\left(c_2\, P_{Total}\, \epsilon^2\, k_1\, c_1 - k_4\, c_4\right) P_{Total}}{c_4\, k_1 \left(\frac{c_2\, P_{Total}\, \epsilon^2\, k_1\, c_1 - k_4\, c_4}{c_4\, k_4} + 1\right)} - \frac{\epsilon\, P_{Total}}{\frac{c_2\, P_{Total}\, \epsilon^2\, k_1\, c_1 - k_4\, c_4}{c_4\, k_4} + 1} - \frac{c_2 \left(c_2\, P_{Total}\, \epsilon^2\, k_1\, c_1 - k_4\, c_4\right) \epsilon\, P_{Total}}{c_4^2\, k_4 \left(\frac{c_2\, P_{Total}\, \epsilon^2\, k_1\, c_1 - k_4\, c_4}{c_4\, k_4} + 1\right)}$$

And thus implies, that for every choice of underlying kinetics and for every feasible concentration of ϵ (and Ap) at the biphasic peak, there exits total amount of substrate and enzyme, where it is possible to observe a enzyme biphasic response with total enzyme concentration (i.e. the behavior to guaranteed to occur for any given underlying kinetics, provided there is flexibility on the total concentrations of the substrate).

Below, we illustrate a parameter set where the biphasic behavior is analytically predicted (and this is shown and confirmed computationally in figure 2)

$$k_1 := 1 : k_4 := 1 : k_{b1} := 1 : k_{b2} := 20 : k_{b4} := 1 : k_{ub1} := 1 : k_{ub2} := 1 : k_{ub4} := 1 : c_1 := \frac{k_{b1}}{k_1 + k_{ub1}} : c_2 := \frac{k_{b2}}{k_{ub2}} : c_4 := \frac{k_{b4}}{k_4 + k_{ub4}} :$$

$$P_{Total} := 1 :$$

$Ap := 1.139478030 : \text{epsilon} := 2 :$

$simplify(AK) = 0.3629514268$
$simplify(A) = 0.5697390150$
$simplify(ApK) = 29.03611414$
$simplify(ApP) = 0.3629514266$

$A_{Total} := solve(ACon) = 31.47123404$
$K_{Total} := solve(KCon) = 30.67316271$

Thus, in this subsection we have shown how for every choice of underlying kinetics the PPI model (without any assumptions on regime of enzyme action) is capable of exhibiting enzyme biphasic in Ap dose response with changing total kinase concentration.

Double site phosphorylation system (DSP)
[common kinase and common phosphatase]
Features and requirements of obtaining biphasic response in the maximally modified substrate

In this file we analytically study the presence of enzyme and substrate biphasic in the maximally modified substrate form. In doing so we establish the following key results with regard to the behavior.

Enzyme biphasic response (biphasic behavior in the dose response curve of App as K_{Total} changes)
1. The maximally modified substrate is incapable of exhibiting enzyme biphasic response irrespective of parameter values.

Substrate biphasic response (biphasic behavior in the dose response curve of App as A_{Total} changes)
1. Substrate biphasic response is possible in App for any kinetic regime (i.e. for any choice of underlying kinetics the system is capable of exhibiting substrate biphasic dose response at some total concentration of enzymes)

We note that the key signature of biphasic behavior in the dose response curve of the system is the presence of a steady state of the system that satisfies the following condition.

$$\frac{dApp}{dK_{Total}} = 0 \text{ (for enzyme biphasic)}$$
$$\frac{dApp}{dA_{Total}} = 0 \text{ (for substrate biphasic)}$$

Model DSP: We first describe the model of double site phosphorylation with common kinase and common phosphatase enzyme action.

We initialize the *Maple* file with the *restart* command and load the relevant libraries of inbuilt *Maple* functions (*LinearAlgebra, VectorCalculus, Student[LinearAlgebra]*)

$restart : with(LinearAlgebra) : with(VectorCalculus) : with(Student[LinearAlgebra]) :$

The system is modeled as a set of ODEs using the kinetic nomenclature described in the main text. Here dA represents d[A]/dt and similarly in the case of the other variables. At steady state thus, the right hand sides of each of these expressions will be equal to zero.

$$dA := k_4 \cdot ApP + k_{ub1} \cdot AK - k_{b1} \cdot A \cdot K :$$
$$dAp := k_1 \cdot AK + k_3 \cdot AppP + k_{ub2} \cdot (ApK) + k_{ub4} \cdot (ApP) - k_{b2} \cdot (Ap) \cdot (K) - k_{b4} \cdot Ap \cdot P :$$
$$dApp := k_2 \cdot ApK + k_{ub3} \cdot AppP - k_{b3} \cdot App \cdot P :$$

$$dAK := k_{b1} \cdot A \cdot K - (k_{ub1} + k_1) \cdot AK :$$
$$dApK := k_{b2} \cdot Ap \cdot K - (k_{ub2} + k_2) \cdot ApK :$$
$$dAppP := k_{b3} \cdot App \cdot P - (k_{ub3} + k_3) \cdot AppP :$$
$$dApP := k_{b4} \cdot Ap \cdot P - (k_{ub4} + k_4) \cdot ApP :$$

$$dK := -k_{b1} A\, K + (k_{ub1} + k_1) AK - k_{b2} Ap\, K + (k_{ub2} + k_2) ApK :$$
$$dP := -k_{b3} App\, P + (k_{ub3} + k_3) AppP - k_{b4} Ap\, P + (k_{ub4} + k_4) ApP :$$

The model is also associated with conservation conditions which are described below. Here we store the conservation expressions as ACon, PCon and KCon for the substrate and the respective enzymes. Each of these expressions is always equal to zero (both in the transient and at steady state).

$$ACon := A + Ap + App + AK + ApK + AppP + ApP - A_{Total} :$$
$$PCon := P + AppP + ApP - P_{Total} :$$
$$KCon := K + AK + ApK - K_{Total} :$$

We now solve the system described at steady state to obtain expression linking the steady state concentrations of the various species. Here we use the Maple command *solve* to solve the equations for a given variable as shown below. We pursue this to finally obtain the steady state concentrations of most species in terms of App and a ratio ϵ (defined below).

$$assign(solve(\{dAK, dApK, dAppP, dApP\}, \{AK, ApK, AppP, ApP\}))$$
$$assign(solve(\{dA, dAp\}, \{A, Ap\}))$$

Further we now introduce a ratio, ϵ = K/P (defined as the ratio of the free enzymes). Simultaneously we introduce the following parameters (c_1, c_2, c_3, and c_4). This is done for the sake of brevity and easy tractability of the expressions obtained.

$$K := \epsilon \cdot P :$$
$$k_{b1} := c_1 \cdot (k_1 + k_{ub1}) : k_{b2} := c_2 \cdot (k_2 + k_{ub2}) :$$
$$k_{b3} := c_3 \cdot (k_3 + k_{ub3}) : k_{b4} := c_4 \cdot (k_4 + k_{ub4}) :$$

Once this is done, we again solve for the steady state of the phosphatase using the conservation expression for the enzyme (PCon).

$$P := simplify(solve(PCon, P)) = \frac{P_{Total}\,\epsilon\,k_2\,c_2}{c_2\,k_2\,(App\,c_3+1)\,\epsilon + c_4\,c_3\,App\,k_3}$$

This results in the following expressions for the steady state concentrations of the various species in terms of the steady state substrate concentration App and ϵ

$$A = \frac{k_4\,c_4\,c_3\,App\,k_3}{\epsilon^2\,k_2\,c_2\,k_1\,c_1}$$

$$Ap = \frac{c_3\,App\,k_3}{\epsilon\,k_2\,c_2}$$

$$AK = \frac{k_4\,c_4\,c_3\,App\,k_3\,P_{Total}}{k_1\,(c_2\,k_2\,(c_3\,App+1)\,\epsilon + c_4\,c_3\,App\,k_3)}$$

$$ApK = \frac{c_2\,c_3\,App\,k_3\,\epsilon\,P_{Total}}{c_2\,k_2\,(c_3\,App+1)\,\epsilon + c_4\,c_3\,App\,k_3}$$

$$AppP = \frac{c_3\,App\,P_{Total}\,\epsilon\,k_2\,c_2}{c_2\,k_2\,(c_3\,App+1)\,\epsilon + c_4\,c_3\,App\,k_3}$$

$$ApP = \frac{c_4\,c_3\,App\,k_3\,P_{Total}}{c_2\,k_2\,(c_3\,App+1)\,\epsilon + c_4\,c_3\,App\,k_3}$$

Note that when App and ϵ are positive, steady state concentrations of the other variable concentrations are positive as well. Thus we have solved the system of equations at steady state to arrive at expressions linking the steady state concentrations of the variables with that of App and ϵ. We now have two expressions, ACon (or ACon_Red if we are working in the enzyme limiting regime - see below) and KCon - the conservation of the substrate and kinase, whose solution for the variables define the steady state of the system.

Enzyme biphasic response

Impossibility of enzyme biphasic behavior in the limiting enzyme regime

In this subsection we show that the DSP model is incapable of enzyme biphasic behavior in the maximally modified substrate form when the enzymes are limiting (where the total substrate concentration is significantly higher than the total enzyme concentration). Thus in such a regime, the conservation expressions for the substrates can be written as shown below (ACon_Red = 0)

$$ACon\_Red := A + Ap + App - A_{Total}:$$

As noted earlier, the biphasic behavior is characterized by the following condition being satisfied for some steady state of the system,

$$\frac{dApp}{dK_{Total}} = 0$$

We now have two remaining conservations, KCon = 0 & ACon_Red = 0 (see below) whose solutions to the variables App and ϵ define the steady state of the system.

$$ACon\_Red = \frac{k_4\,c_4\,c_3\,App\,k_3}{\epsilon^2\,k_2\,c_2\,k_1\,c_1} + \frac{c_3\,App\,k_3}{\epsilon\,k_2\,c_2} + App - A_{Total}$$

$KCon =$

$$\frac{\epsilon^2 P_{Total} k_2 c_2}{c_2 k_2 \left(c_3 App + 1\right) \epsilon + c_4 c_3 App k_3} + \frac{k_4 c_4 c_3 App k_3 P_{Total}}{k_1 \left(c_2 k_2 \left(c_3 App + 1\right) \epsilon + c_4 c_3 App k_3\right)} + \frac{c_2 c_3 App k_3 \epsilon P_{Total}}{c_2 k_2 \left(c_3 App + 1\right) \epsilon + c_4 c_3 App k_3} - K_{Total}$$

If we differentiate both these with respect to the total kinase in the system, we get

$$\frac{dACon\_Red}{dK_{Total}} = 0 = \frac{\partial ACon\_Red}{\partial App} \cdot \frac{\partial App}{\partial K_{Total}} + \frac{\partial ACon\_Red}{\partial \epsilon} \cdot \frac{\partial \epsilon}{\partial K_{Total}}$$

$$\frac{dKCon}{dK_{Total}} = 0 = \frac{\partial KCon}{\partial K_{Total}} + \frac{\partial KCon}{\partial App} \cdot \frac{\partial App}{\partial K_{Total}} + \frac{\partial KCon}{\partial \epsilon} \cdot \frac{\partial \epsilon}{\partial K_{Total}}$$

Now in order to show the absence of an enzyme biphasic response in this regime of enzyme action, we use a proof by contradiction. If we are to assume that there exists a biphasic response, then $\frac{dApp}{dK_{Total}} = 0$ for some steady state.

This results in the above expressions being simplified as follows.

$$0 = \frac{\partial ACon\_Red}{\partial \epsilon} \cdot \frac{\partial \epsilon}{\partial K_{Total}}$$

$$1 = \frac{\partial KCon}{\partial \epsilon} \cdot \frac{\partial \epsilon}{\partial K_{Total}}$$

Note

This simplification is possible since the functions $\frac{\partial ACon\_Red}{\partial App}, \frac{\partial KCon}{\partial App}$ are finite and always have non-zero denominatorss, and thus the products involving $\frac{\partial App}{\partial K_{Total}}$ can be zero.

$simplify(diff(ACon\_Red, App)) =$

$$\frac{1}{\left(Ap\, c_2 + Bp\, d_2 + 1\right)^2 K k_1 c_1 p_1} \Big(\Big(\Big(Ap^2 k_1 c_2^2 + \left(\left(P_{Total} + 2\, Ap \right) k_1 + P_{Total} k_2 \right) \left(Bp\, d_2 + 1 \right) c_2 + k_1 \left(Bp\, d_2 + 1 \right)^2 \Big) K c_1 + P_{Total} c_2 k_2 \left(Bp\, d_2 + 1 \right) \Big) p_1 - d_2 Bp\, P_{Total} p_2 c_2 K k_1 c_1 \Big)$$

$simplify(diff(KCon, App)) =$

$$-\frac{\left(c_2 d_1 \left(p_1 + p_2\right) Ap^2 + 2\, p_2 c_2 Ap + p_2 \left(Bp\, d_2 + 1\right)\right) d_2 Bp\, P_{Total}}{\left(Ap\, c_2 + Bp\, d_2 + 1\right)^2 Ap^2 p_1 d_1}$$

However we know that $\frac{\partial ACon\_Red}{\partial \epsilon}$ is non-zero (see below).

$$simplify(diff(ACon\_Red, \epsilon)) = -\frac{c_3 App\, k_3 \left(c_1 \epsilon k_1 + 2\, c_4 k_4\right)}{\epsilon^3 k_2 c_2 k_1 c_1}$$

This implies that $\frac{\partial \epsilon}{\partial K_{Total}}$ is zero. However this leads to a contradiction as

$1 = \frac{\partial KCon}{\partial \epsilon} \cdot \frac{\partial \epsilon}{\partial K_{Total}}$. (Note that the denominator of $\frac{\partial KCon}{\partial \epsilon}$ is non-zero as well).

Hence our assumption must be wrong.

Thus enzyme biphasic behavior in App with K_{Total} is impossible when total substrate concentration is significantly higher than total enzyme concentrations.

Presence and necessary analytical condition (involving catalytic constants) for biphasic behavior to exist

In this subsection, we analytically show the presence of enzyme biphasic (in the full system) and extract kinetic constraints that enable (and preclude) biphasic behavior. As noted earlier, the biphasic behavior is characterized by the following condition being satisfied for some steady state of the system.

$$\frac{\partial App}{\partial K_{Total}} = 0$$

We now have two remaining conservations, KCon = 0 & ACon = 0 (see below) whose solutions to the variables App and ϵ define the steady state of the system.

$$ACon = \frac{k_4 c_4 c_3 App\, k_3}{\epsilon^2 k_2 c_2 k_1 c_1} + \frac{c_3 App\, k_3}{\epsilon k_2 c_2} + App + \frac{k_4 c_4 c_3 App\, k_3 P_{Total}}{k_1 \left(c_2 k_2 \left(c_3 App + 1\right) \epsilon + c_4 c_3 App\, k_3\right)} + \frac{c_2 c_3 App\, k_3 \epsilon P_{Total}}{c_2 k_2 \left(c_3 App + 1\right) \epsilon + c_4 c_3 App\, k_3} + \frac{c_3 App\, P_{Total} \epsilon k_2 c_2}{c_2 k_2 \left(c_3 App + 1\right) \epsilon + c_4 c_3 App\, k_3} + \frac{c_4 c_3 App\, k_3 P_{Total}}{c_2 k_2 \left(c_3 App + 1\right) \epsilon + c_4 c_3 App\, k_3} - A_{Total}$$

$$KCon = \frac{\epsilon^2 P_{Total} k_2 c_2}{c_2 k_2 \left(c_3 App + 1\right) \epsilon + c_4 c_3 App\, k_3} + \frac{k_4 c_4 c_3 App\, k_3 P_{Total}}{k_1 \left(c_2 k_2 \left(c_3 App + 1\right) \epsilon + c_4 c_3 App\, k_3\right)} + \frac{c_2 c_3 App\, k_3 \epsilon P_{Total}}{c_2 k_2 \left(c_3 App + 1\right) \epsilon + c_4 c_3 App\, k_3} - K_{Total}$$

If we differentiate both these with respect to the total kinase concentration in the system, we get

$$\frac{dACon}{dK_{Total}} = 0 = \frac{\partial ACon}{\partial App} \cdot \frac{\partial App}{\partial K_{Total}} + \frac{\partial ACon}{\partial \epsilon} \cdot \frac{\partial \epsilon}{\partial K_{Total}}$$

$$\frac{dKCon}{dK_{Total}} = 0 = \frac{\partial KCon}{\partial K_{Total}} + \frac{\partial KCon}{\partial App} \cdot \frac{\partial App}{\partial K_{Total}} + \frac{\partial KCon}{\partial \epsilon} \cdot \frac{\partial \epsilon}{\partial K_{Total}}$$

Now in order to show the presence of an enzyme biphasic response and study its features, we begin with exploring the necessary features that the system must satisfy for the behavior to exist. We begin with the basic tenet that for the behavior there should exist a steady state of the system where $\frac{dApp}{dK_{Total}} = 0$ is satisfied.

At this point then, the above expressions simply as follows

$$0 = \frac{\partial ACon}{\partial \epsilon} \cdot \frac{\partial \epsilon}{\partial K_{Total}}$$

$$1 = \frac{\partial KCon}{\partial \epsilon} \cdot \frac{\partial \epsilon}{\partial K_{Total}}$$

Note

This simplification is possible since the functions $\frac{\partial ACon}{\partial App}, \frac{\partial KCon}{\partial App}$ are finite and always have non-zero denominators, and thus the products involving $\frac{\partial App}{\partial K_{Total}}$ can be zero.

$simplify(diff(ACon, App)) =$

$$\left(k_2^2 c_2^3 \left(App^2 c_3^2 k_2 + \left(\left(2\, App + P_{Total}\right) k_2 + P_{Total} k_3\right) c_3 + k_2\right) k_1 c_1 \epsilon^4 + k_2^2 c_2^2 \left(App^2 c_3^2 k_1 + 2\, App\, k_1 \left(c_4 App + 1\right) c_3 + 2\, App\, c_4 k_1 + P_{Total} c_4 k_1 + P_{Total} c_4 k_4 + k_1\right) k_3 c_1 c_3 \epsilon^3 + 2\, k_2 c_2 c_4 \left(\frac{k_2 k_4 \left(c_3 App + 1\right)^2 c_2}{2} + App\, c_1 c_3 k_1 k_3 \left(c_3 App + \frac{1}{2} c_4 App + 1\right)\right) k_3 c_3 \epsilon^2 + \left(2\, k_2 k_4 \left(c_3 App + 1\right) c_2 + App\, c_1 c_3 k_1 k_3\right) c_4^2 App\, k_3^2 c_3^2 \epsilon + App^2 c_3^3 c_4^3 k_3^3 k_4\right) \Big/ \left(k_2 \left(c_2 k_2 \left(c_3 App + 1\right) \epsilon + c_4 c_3 App\, k_3\right)^2 c_2 k_1 c_1 \epsilon^2\right)$$

$$simplify(diff(KCon, App)) = -\frac{c_3 \epsilon k_2 \left(k_1 c_2 k_2 \epsilon^2 - k_1 k_3 \left(c_2 - c_4\right) \epsilon - k_4 c_4 k_3\right) c_2 P_{Total}}{\left(c_2 k_2 \left(c_3 App + 1\right) \epsilon + c_4 c_3 App\, k_3\right)^2 k_1}$$

We know that the denominator of $\frac{\partial KCon}{\partial \epsilon}$ is non-zero, thus this implies that $\frac{\partial \epsilon}{\partial K_{Total}}$ has to be non-zero in order to satisfy the second expression above.

This insight then informs us that in order to satisfy the first expression, $\frac{\partial ACon}{\partial \epsilon}$ must be equal to zero.

$Condition := simplify(diff(ACon, \epsilon))$

$$Condition := -\left(App\, k_3 \left(k_2 c_2^2 \left(\left(App^2 c_3^2 k_1 + App \left(P_{Total} c_4 k_4 + 2\, k_1\right) c_3 + P_{Total} \left(k_1 + k_4\right) c_4 + k_1\right) k_2 - App\, P_{Total} c_3 c_4 k_1 k_3\right) c_1 \epsilon^3 + 2\, k_2 \left(k_2 k_4 \left(c_3 App + 1\right) c_2 + App\, c_1 c_3 k_1 k_3\right) c_2 \left(c_3 App + 1\right) c_4 \epsilon^2 + \left(4\, k_2 k_4 \left(c_3 App + 1\right) c_2 + App\, c_1 c_3 k_1 k_3\right) c_4^2 App\, k_3 c_3 \epsilon + 2\, App^2 c_3^2 c_4^3 k_3^2 k_4\right) c_3\right) \Big/ \left(k_2 \left(c_2 k_2 \left(c_3 App + 1\right) \epsilon + c_4 c_3 App\, k_3\right)^2 c_2 k_1 c_1 \epsilon^3\right) \quad \textbf{(1.2.1)}$$

In order for this expression to equal zero, the numerator of it (which a polynomial in ϵ/App) to be equal to zero. Isolating and studying it as a polynomial in App (as shown below), we can observe that all except the coefficient of the second exponent are negative.

$collect(numer(Condition), App)$

$$-k_3 \left(c_1 c_2^2 c_3^2 \epsilon^3 k_1 k_2^2 + 2 c_1 c_2 c_3^2 c_4 \epsilon^2 k_1 k_2 k_3 + 2 c_2^2 c_3^2 c_4 \epsilon^2 k_2^2 k_4 + c_1 c_3^2 c_4^2 \epsilon k_1 k_3^2 + 4 c_2 c_3^2 c_4^2 \epsilon k_2 k_3 k_4 + 2 c_3^2 c_4^3 k_3^2 k_4\right) c_3 App^3 - k_3 \left(-c_1 c_2^2 c_3 c_4 \epsilon^3 k_1 k_2 k_3 P_{Total} + c_1 c_2^2 c_3 c_4 \epsilon^3 k_2^2 k_4 P_{Total} + 2 c_1 c_2^2 c_3 \epsilon^3 k_1 k_2^2 + 2 c_1 c_2 c_3 c_4 \epsilon^2 k_1 k_2 k_3 + 4 c_2^2 c_3 c_4 \epsilon^2 k_2^2 k_4 + 4 c_2 c_3 c_4^2 \epsilon k_2 k_3 k_4\right) c_3 App^2 - k_3 \left(c_1 c_2^2 c_4 \epsilon^3 k_1 k_2^2 P_{Total} + c_1 c_2^2 c_4 \epsilon^3 k_2^2 k_4 P_{Total} + c_1 c_2^2 \epsilon^3 k_1 k_2^2 + 2 c_2^2 c_4 \epsilon^2 k_2^2 k_4\right) c_3 App \quad \textbf{(1.2.2)}$$

Thus for the polynomial to equal zero, this coefficient must be positive. Isolating the coefficient further as shown below, reveals that $k_1 k_3 - k_2 k_4$ must be greater than zero for this to be true.

$$simplify\left(-k_3 \left(-c_1 c_2^2 c_3 c_4 \epsilon^3 k_1 k_2 k_3 P_{Total} + c_1 c_2^2 c_3 c_4 \epsilon^3 k_2^2 k_4 P_{Total} + 2 c_1 c_2^2 c_3 \epsilon^3 k_1 k_2^2 + 2 c_1 c_2 c_3 c_4 \epsilon^2 k_1 k_2 k_3 + 4 c_2^2 c_3 c_4 \epsilon^2 k_2^2 k_4 + 4 c_2 c_3 c_4^2 \epsilon k_2 k_3 k_4\right) c_3 App^2\right)$$

$$k_2 c_2 \left(\left(P_{Total} \left(k_1 k_3 - k_2 k_4\right) c_4 - 2 k_1 k_2\right) c_1 c_2 \epsilon^2 - 2 c_4 \left(c_1 k_1 k_3 + 2 c_2 k_2 k_4\right) \epsilon - 4 c_4^2 k_3 k_4\right) App^2 k_3 c_3^2 \epsilon \quad \textbf{(1.2.3)}$$

Thus, this becomes a necessary condition for the presence of biphasic dose response in the maximally modified substrate (App) with total enzyme concentration (K_{Total}).

Necessary condition for enzyme biphasic dose response: $k_1 k_3 - k_2 k_4 > 0$

It is worth noting that from a different study (C. Conradi and M. Mincheva, "Catalytic constants enable the emergence of bistability in dual phosphorylation," J. R. Soc. Interface, vol. 11, no. 95, 2014, doi: 10.1098/rsif.2014.0158) it was established that if $k_1 k_3 - k_2 k_4 < 0$ then multi-stationarity is guaranteed for some finite positive total enzyme, substrate concentrations.

We now make a comment regarding the contrast in the necessary and sufficient condition for obtaining biphasic behavior (result here) and the condition enabling bistability in the model (Conradi et al., 2014). While the conditions contrast it is possible to obtain both biphasic and bistability for the same underlying kinetic system (but different choice of total concentrations of substrates and enzymes). This is depicted in the main text in figure N-2.

Comment on guarantees of biphasic behavior:

In this discussion we make the augment to show that should the necessary condition above be satisfied, then there exists total concentrations of substrate (A_{Total}) and phosphatase (P_{Total}).

In order to make the argument, we recapitulate that the steady state of the system is defined by the solutions to the expressions ACon and KCon. However since we are

$ACon$

$$\frac{k_4 c_4 c_3 App\, k_3}{\epsilon^2 k_2 c_2 k_1 c_1} + \frac{c_3 App\, k_3}{\epsilon k_2 c_2} + App + \frac{k_4 c_4 c_3 App\, k_3 P_{Total}}{k_1 \left(c_2 k_2 \left(c_3 App + 1\right) \epsilon + c_4 c_3 App\, k_3\right)} + \frac{c_2 c_3 App\, k_3 \epsilon P_{Total}}{c_2 k_2 \left(c_3 App + 1\right) \epsilon + c_4 c_3 App\, k_3} + \frac{c_3 App\, P_{Total} \epsilon k_2 c_2}{c_2 k_2 \left(c_3 App + 1\right) \epsilon + c_4 c_3 App\, k_3} + \frac{c_4 c_3 App\, k_3 P_{Total}}{c_2 k_2 \left(c_3 App + 1\right) \epsilon + c_4 c_3 App\, k_3} - A_{Total} \quad \textbf{(1.2.4)}$$

$KCon$

$$\frac{\epsilon^2 P_{Total} k_2 c_2}{c_2 k_2 (c_3 App + 1) \epsilon + c_4 c_3 App k_3} + \frac{k_4 c_4 c_3 App k_3 P_{Total}}{k_1 (c_2 k_2 (c_3 App + 1) \epsilon + c_4 c_3 App k_3)} + \frac{c_2 c_3 App k_3 \epsilon P_{Total}}{c_2 k_2 (c_3 App + 1) \epsilon + c_4 c_3 App k_3} - K_{Total} \quad \textbf{(1.2.5)}$$

However, since we have complete flexibility for the total amounts, any feasible concentration of App and ϵ can be made to satisfy ACon and KCon by choosing A_{Total} and K_{Total}.

Thus in order to have a steady state which presents with a biphasic response, it is thus enough to find a feasible concentration of App and ϵ that satisfies the necessary condition for biphasic response from earlier ($\frac{dApp}{dK_{Total}} = 0$).
We recall that this line of reasoning resulted in requiring us to find a steady state of App and ϵ that satisfies $\frac{\partial ACon}{\partial \epsilon} = 0$. This resulted in the following expression.

$$collect\left(\frac{-numer(Condition)}{App}, App\right)$$

$$k_3 \left(c_1 c_2^2 c_3^2 \epsilon^3 k_1 k_2^2 + 2 c_1 c_2 c_3^2 c_4 \epsilon^2 k_1 k_2 k_3 + 2 c_2^2 c_3^2 c_4 \epsilon^2 k_2^2 k_4 + c_1 c_3^2 c_4^2 \epsilon k_1 k_3^2 + 4 c_2 c_3^2 c_4^2 \epsilon k_2 k_3 k_4 + 2 c_3^2 c_4^3 k_3^2 k_4\right) c_3 App^2 + k_3 \left(-c_1 c_2^2 c_3 c_4 \epsilon^3 k_1 k_2 k_3 P_{Total} + c_1 c_2^2 c_3 c_4 \epsilon^3 k_2^2 k_4 P_{Total} + 2 c_1 c_2^2 c_3 \epsilon^3 k_1 k_2^2 + 2 c_1 c_2 c_3 c_4 \epsilon^2 k_1 k_2 k_3 + 4 c_2^2 c_3 c_4 \epsilon^2 k_2^2 k_4 + 4 c_2 c_3 c_4^2 \epsilon k_2 k_3 k_4\right) c_3 App + k_3 \left(c_1 c_2^2 c_4 \epsilon^3 k_1 k_2^2 P_{Total} + c_1 c_2^2 c_4 \epsilon^3 k_2^2 k_4 P_{Total} + c_1 c_2^2 \epsilon^3 k_1 k_2^2 + 2 c_2^2 c_4 \epsilon^2 k_2^2 k_4\right) c_3 \quad \textbf{(1.2.6)}$$

This is a second order polynomial in App (after we have factored an App from the expression). Now we can observe (as we have observed earlier) that the leading coefficient and the constant term are always positive, while the coefficient of the first exponent can be negative (this is the coefficient from which we discerned the necessary condition).

$$simplify\left(-c_1 c_2^2 c_3 c_4 \epsilon^3 k_1 k_2 k_3 P_{Total} + c_1 c_2^2 c_3 c_4 \epsilon^3 k_2^2 k_4 P_{Total} + 2 c_1 c_2^2 c_3 \epsilon^3 k_1 k_2^2 + 2 c_1 c_2 c_3 c_4 \epsilon^2 k_1 k_2 k_3 + 4 c_2^2 c_3 c_4 \epsilon^2 k_2^2 k_4 + 4 c_2 c_3 c_4^2 \epsilon k_2 k_3 k_4\right)$$

$$-c_3 \epsilon \left(\left(P_{Total} (k_1 k_3 - k_2 k_4) c_4 - 2 k_1 k_2\right) c_1 c_2 \epsilon^2 - 2 c_4 (c_1 k_1 k_3 + 2 c_2 k_2 k_4) \epsilon - 4 c_4^2 k_3 k_4\right) k_2 c_2 \quad \textbf{(1.2.7)}$$

Now we can observe that if the necessary condition is satisfied ($k_1 k_3 - k_2 k_4 > 0$), the phosphatase concentration can be sufficiently increased to make the whole expression further negative. This implies that if the necessary condition is satisfied then, by varying (increasing) the total phosphatase concentration, the coefficient can be made more and more negative. Thus, this implies that for some arbitrary choice of ϵ there exists feasible (positive) values of App for which the expression is guaranteed to be 0.

Thus this implies that should the required necessary condition be satisfied, then for a high enough P_{Total}, there exits an App and ϵ (which provides A_{Total}, K_{Total} from there on), which satisfies, $\frac{\partial ACon}{\partial \epsilon} = 0$ and thus satisfies the necessary requirement of the enzyme biphasic response of App with K_{Total}. Thus ensuring the sufficiency of the necessary condition to guarantee the existence

enzyme biphasic response for some total concentration of substrate and enzymes.

Substrate biphasic response

In this subsection, we analytically show the presence of and study the features of substrate biphasic (in the full system) and extract kinetic constraints that enable (and preclude) biphasic behavior. As noted earlier, the biphasic behavior is characterized by the following condition being satisfied for some steady state of the system.

$$\frac{dApp}{dA_{Total}} = 0$$

We now have two remaining conservations, KCon = 0 & ACon = 0 (see below) whose solutions to the variables App and ϵ define the steady state of the system.

$$ACon = \frac{k_4 c_4 c_3 App\, k_3}{\epsilon^2 k_2 c_2 k_1 c_1} + \frac{c_3 App\, k_3}{\epsilon k_2 c_2} + App + \frac{k_4 c_4 c_3 App\, k_3 P_{Total}}{k_1 \left(c_2 k_2 \left(c_3 App + 1\right) \epsilon + c_4 c_3 App\, k_3\right)} + \frac{c_2 c_3 App\, k_3 \epsilon P_{Total}}{c_2 k_2 \left(c_3 App + 1\right) \epsilon + c_4 c_3 App\, k_3} + \frac{c_3 App\, P_{Total} \epsilon k_2 c_2}{c_2 k_2 \left(c_3 App + 1\right) \epsilon + c_4 c_3 App\, k_3} + \frac{c_4 c_3 App\, k_3 P_{Total}}{c_2 k_2 \left(c_3 App + 1\right) \epsilon + c_4 c_3 App\, k_3} - A_{Total}$$

$$KCon = \frac{\epsilon^2 P_{Total} k_2 c_2}{c_2 k_2 \left(c_3 App + 1\right) \epsilon + c_4 c_3 App\, k_3} + \frac{k_4 c_4 c_3 App\, k_3 P_{Total}}{k_1 \left(c_2 k_2 \left(c_3 App + 1\right) \epsilon + c_4 c_3 App\, k_3\right)} + \frac{c_2 c_3 App\, k_3 \epsilon P_{Total}}{c_2 k_2 \left(c_3 App + 1\right) \epsilon + c_4 c_3 App\, k_3} - K_{Total}$$

If we differentiate both these with respect to the total substrate concentration in the system, we get

$$\frac{dACon}{dA_{Total}} = 0 = \frac{\partial ACon}{\partial A_{Total}} + \frac{\partial ACon}{\partial App} \cdot \frac{\partial App}{\partial A_{Total}} + \frac{\partial ACon}{\partial \epsilon} \cdot \frac{\partial \epsilon}{\partial A_{Total}}$$

$$\frac{dKCon}{dA_{Total}} = 0 = \frac{\partial KCon}{\partial App} \cdot \frac{\partial App}{\partial A_{Total}} + \frac{\partial KCon}{\partial \epsilon} \cdot \frac{\partial \epsilon}{\partial A_{Total}}$$

Now in order to show the presence of a substrate biphasic response and study its features, we begin with exploring the necessary features that the system must satisfy for the behavior to exist. We begin with the basic tenet that for the behavior there should exist a steady state of the system where $\frac{\partial App}{\partial A_{Total}} = 0$ is satisfied.

At this point then, the above expressions simply as follows

$$1 = \frac{\partial ACon}{\partial \epsilon} \cdot \frac{\partial \epsilon}{\partial A_{Total}}$$

$$0 = \frac{\partial KCon}{\partial \epsilon} \cdot \frac{\partial \epsilon}{\partial A_{Total}}$$

Note

This simplification is possible since the functions $\frac{\partial ACon}{\partial App}$, $\frac{\partial KCon}{\partial App}$ are finite and always have non-zero denominators (as shown below), and thus the products involving $\frac{\partial App}{\partial A_{Total}}$ can be zero.

$simplify(diff(ACon, App)) =$

$$\Big(\big(App^2 c_3^2 k_2 + \big((2\,App + P_{Total})\,k_2 + P_{Total}\,k_3\big)\,c_3 + k_2\big)\,c_2^3 c_1 k_2^2 k_1 \epsilon^4 + k_3\,\big(App^2 c_3^2 k_1 + 2\,App\,k_1\,(c_4\,App + 1)\,c_3 + 2\,App\,c_4 k_1 + P_{Total}\,c_4 k_1 + P_{Total}\,c_4 k_4 + k_1\big)\,c_2^2 c_1 k_2^2 c_3 \epsilon^3 + 2\left(\frac{k_2 k_4\,(c_3\,App + 1)^2 c_2}{2} + App\,c_1 c_3 k_1 k_3\left(c_3\,App + \frac{1}{2}\,c_4\,App + 1\right)\right) k_3 c_4 c_2 k_2 c_3 \epsilon^2 + App\,k_3^2 c_4^2\,\big(2\,k_2 k_4\,(c_3\,App + 1)\,c_2 + App\,c_1 c_3 k_1 k_3\big)\,c_3^2\,\epsilon + App^2 c_3^3 c_4^3 k_3^3 k_4\Big) \Big/ \Big(c_2 c_1 k_2 \epsilon^2 k_1\,\big(c_2 k_2\,(c_3\,App + 1)\,\epsilon + c_4 c_3\,App\,k_3\big)^2\Big)$$

$$simplify(diff(KCon, App)) = -\frac{\epsilon\,\big(k_1 c_2 k_2 \epsilon^2 - k_1 k_3\,(c_2 - c_4)\,\epsilon - k_4 c_4 k_3\big)\,c_3 c_2 P_{Total} k_2}{\big(c_2 k_2\,(c_3\,App + 1)\,\epsilon + c_4 c_3\,App\,k_3\big)^2 k_1}$$

Observing, the second expression above (from differentiation of the total kinase concentration) we can see that either $\frac{\partial KCon}{\partial \epsilon}$ or $\frac{\partial \epsilon}{\partial A_{Total}}$ must be equal to zero. However, $\frac{\partial \epsilon}{\partial A_{Total}}$ cannot be zero as, if it was indeed zero, then since the denominator of $\frac{\partial ACon}{\partial \epsilon}$ is non-zero, there would be a contradiction with the first expression (from differentiation of the total substrate concentration). Thus, $\frac{\partial KCon}{\partial \epsilon}$ must be equal to zero.

$Condition := simplify(diff(KCon, \text{epsilon}))$

$$Condition := \frac{1}{\big(App\,(\epsilon\,k_2 c_2 + c_4 k_3)\,c_3 + \epsilon\,k_2 c_2\big)^2 k_1}\left(\left(App^2 c_4 k_3\,(k_1 k_3 - k_2 k_4)\,c_3^2 + App\left(k_1 c_2 k_2 \epsilon^2 + 2\,k_3\left(k_1\,\epsilon - \frac{k_4}{2}\right) c_4\right) k_2 c_3 + \epsilon^2 k_2^2 c_2 k_1\right) c_2 P_{Total}\right) \quad \textbf{(2.1)}$$

Now for this expression to be zero, the numerator (which is a function of App and epsilon) must be equal to zero. isolating this, we get the following expression

$simplify(numer(Condition))$

$$\left(App^2 c_4 k_3\,(k_1 k_3 - k_2 k_4)\,c_3^2 + App\left(k_1 c_2 k_2 \epsilon^2 + 2\,k_3\left(k_1\,\epsilon - \frac{k_4}{2}\right) c_4\right) k_2 c_3 + \epsilon^2 k_2^2 c_2 k_1\right) c_2 P_{Total} \quad \textbf{(2.2)}$$

We recap here that, so long as this expression is zero for a feasible steady state concentrations of App and epsilon, we can find total concentrations of substrates and enzyme where the biphasic response is guaranteed. Keeping this in mind, we rewrite the expression as a polynomial function of epsilon as shown below

$collect(numer(Condition), \text{epsilon})$

$$\left(App\, k_1 c_2 k_2^2 c_3 + k_2^2 c_2 k_1\right) c_2 P_{Total}\, \epsilon^2 + 2\, App\, k_1 k_3 c_4 k_2 c_3 c_2 P_{Total}\, \epsilon + \left(App^2 c_3^2 c_4 k_1 k_3^2 - App^2 c_3^2 c_4 k_2 k_3 k_4 - App\, c_3 c_4 k_2 k_3 k_4\right) c_2 P_{Total} \quad \textbf{(2.3)}$$

This allows us to observe that so long as the constant term (which is itself a function of App) is negative, there is guaranteed to exist a steady state of the system where the expression is zero and thus where the system accommodate a biphasic dose response with total substrate concentration (A_{Total}).

Isolating the constant we can observe the grouping of catalytic constants from the enzyme biphasic responses' necessary condition appear here too.

$simplify\left(\left(App^2 c_3^2 c_4 k_1 k_3^2 - App^2 c_3^2 c_4 k_2 k_3 k_4 - App\, c_3 c_4 k_2 k_3 k_4\right) c_2 P_{Total}\right)$

$$App\, P_{Total}\, c_3 c_2 k_3 \left(App\left(k_1 k_3 - k_2 k_4\right) c_3 - k_2 k_4\right) c_4 \quad \textbf{(2.4)}$$

Now, if this catalytic constant grouping ($k_1 k_3 - k_2 k_4$) is negative, then the constant term is negative irrespective of the steady state concentration of App and thus the whole expression from earlier is zero, guaranteeing substrate biphasic response. This is because, if the key term is negative, for any given concentration of App, there will exist an ϵ root for which the polynomial in 2.3. However, if the grouping ($k_1 k_3 - k_2 k_4$) is positive, then the constant term can still be negative, however in this case App (at the biphasic peak) is necessarily to be less than $\dfrac{k_2 k_4}{c_3 \cdot \left(k_1 k_3 - k_2 k_4\right)}$

This is however still feasible since, App and ϵ can take any positive concentration and we can find a suitable total concentration of substrate and enzyme that will accommodate it as steady state concentrations (using ACon and KCon which we are yet to solve).

$$ACon = \frac{k_4 c_4 c_3 App\, k_3}{\epsilon^2 k_2 c_2 k_1 c_1} + \frac{c_3 App\, k_3}{\epsilon k_2 c_2} + App + \frac{k_4 c_4 c_3 App\, k_3 P_{Total}}{k_1\left(c_2 k_2\left(c_3 App + 1\right)\epsilon + c_4 c_3 App\, k_3\right)} + \frac{c_2 c_3 App\, k_3\, \epsilon P_{Total}}{c_2 k_2\left(c_3 App + 1\right)\epsilon + c_4 c_3 App\, k_3} + \frac{c_3 App\, P_{Total}\, \epsilon k_2 c_2}{c_2 k_2\left(c_3 App + 1\right)\epsilon + c_4 c_3 App\, k_3} + \frac{c_4 c_3 App\, k_3 P_{Total}}{c_2 k_2\left(c_3 App + 1\right)\epsilon + c_4 c_3 App\, k_3} - A_{Total}$$

$$KCon = \frac{\epsilon^2 P_{Total}\, k_2 c_2}{c_2 k_2\left(c_3 App + 1\right)\epsilon + c_4 c_3 App\, k_3} + \frac{k_4 c_4 c_3 App\, k_3 P_{Total}}{k_1\left(c_2 k_2\left(c_3 App + 1\right)\epsilon + c_4 c_3 App\, k_3\right)} + \frac{c_2 c_3 App\, k_3\, \epsilon P_{Total}}{c_2 k_2\left(c_3 App + 1\right)\epsilon + c_4 c_3 App\, k_3} - K_{Total}$$

Thus, what this implies is that, irrespective of the underlying grouping (or the sign of the catalytic constant grouping $k_1 k_3 - k_2 k_4$), substrate biphasic behavior is guaranteed to exist for some total concentration of substrate and enzyme in the system.

However, we wish to note here that depending on the sign of the catalytic constant grouping $k_1 k_3 - k_2 k_4$ it is either more likely or less likely to observe substrate biphasic response in App with total amount of substrate in the system. Further, depending on the sign of the catalytic constant grouping (if $k_1 k_3 - k_2 k_4 > 0$), the peak concentration of the biphasic response capable of being observed is capped at

$\frac{k_2 k_4}{c_3 \cdot (k_1 k_3 - k_2 k_4)}$ which is not the case when ($k_1 k_3 - k_2 k_4 < 0$) when App can take any value at biphasic peak (given that the total amounts are completely flexible.

Double site phosphorylation system (DSP) [enzyme combinations - same/different acting on the two modification sites]

Features and requirements to obtain substrate and enzyme biphasic behavior in the maximally modified substrate

In this folder we detail analytical results pertaining to biphasic response in the maximally modified substrate in various models of the DSP (with common or different enzymes acting on the two modification sites). Specifically, we study each system's capacity to exhibit biphasic responses in the with changing total concentration of substrate (substrate biphasic) and total concentration of enzyme (enzyme biphasic).

A summary of the results is presented in the table below.

nd Enzyme biphasic dose responses in the double site ordered modification system with common/different en

System	Substrate Biphasic	Enzyme Biphasic
Common Kinase<br>Seperate Phophatase	Present	Not possible
Separate Kinase<br>Common Phosphatase	Not possible	Present with $K2_{Total}$<br>Not possible with $K1_{Total}$
Separate Kinase<br>Separate Phosphatase	Not possible	Not possible

Double site phosphorylation system (DSP)
[common kinase and separate phosphatase]
Features and requirements of obtaining biphasic response in the maximally modified substrate

In this file we analytically study the presence of enzyme and substrate biphasic in the maximally modified substrate form. In doing so we establish the following key results with regard to the behavior.

Enzyme biphasic response (biphasic behavior in the dose response curve of App as K_{Total} changes)
1. The system is incapable of exhibiting enzyme biphasic dose response with increasing amounts of total kinase.

Substrate biphasic response (biphasic behavior in the dose response curve of App as A_{Total} changes)
1. Substrate biphasic response is possible in App for any kinetic regime (i.e. for any choice of underlying kinetics the system is capable of exhibiting substrate biphasic dose response at some total concentration of enzymes)

We note that the key signature of biphasic behavior in the dose response curve of the system is the presence of a steady state of the system that satisfies the following condition.

$$\frac{dApp}{dK_{Total}} = 0 \ \text{(for enzyme biphasic)}$$
$$\frac{dApp}{dA_{Total}} = 0 \ \text{(for substrate biphasic)}$$

Model DSP: We first describe the model of double site phosphorylation with common kinase and different phosphatase acting on each modification site.

We initialize the *Maple* file with the *restart* command and load the relevant libraries of inbuilt *Maple* functions (*LinearAlgebra, VectorCalculus, Student[LinearAlgebra]*)

$restart : with(LinearAlgebra) : with(VectorCalculus) : with(Student[LinearAlgebra]) :$

The system is modeled as a set of ODEs using the kinetic nomenclature described in the main text. Here dA represents d[A]/dt and similarly in the case of the other variables. At steady state thus, the right hand sides of each of these expressions will be equal to zero.

$$dA := k_4 \cdot ApP1 + k_{ub1} \cdot AK - k_{b1} \cdot A \cdot K :$$
$$dAp := k_1 \cdot AK + k_3 \cdot AppP2 + k_{ub2} \cdot (ApK) + k_{ub4} \cdot (ApP1) - k_{b2} \cdot (Ap) \cdot (K) - k_{b4} \cdot Ap \cdot P1 :$$
$$dApp := k_2 \cdot ApK + k_{ub3} \cdot AppP2 - k_{b3} \cdot App \cdot P2 :$$

$$dAK := k_{b1} \cdot A \cdot K - (k_{ub1} + k_1) \cdot AK :$$
$$dApK := k_{b2} \cdot Ap \cdot K - (k_{ub2} + k_2) \cdot ApK :$$
$$dAppP2 := k_{b3} \cdot App \cdot P2 - (k_{ub3} + k_3) \cdot AppP2 :$$
$$dApP1 := k_{b4} \cdot Ap \cdot P1 - (k_{ub4} + k_4) \cdot ApP1 :$$

$$dK := -k_{b1} A\, K + (k_{ub1} w + k_1) AK - k_{b2} Ap\, K + (k_{ub2} + k_2) ApK :$$
$$dP1 := -k_{b4} Ap\, P1 + (k_{ub4} + k_4) ApP1 :$$
$$dP2 := -k_{b3} App\, P2 + (k_{ub3} + k_3) AppP2 :$$

The model is also associated with conservation conditions which are described below. Here we store the conservation expressions as ACon, P1Con, P2Con and KCon for the substrate and the respective enzymes. Each of these expressions is always equal to zero (both in the transient and at steady state).

$$ACon := A + Ap + App + AK + ApK + AppP2 + ApP1 - A_{Total} :$$
$$P1Con := P1 + ApP1 - P1_{Total} :$$
$$P2Con := P2 + AppP2 - P2_{Total} :$$
$$KCon := K + AK + ApK - K_{Total} :$$

We now solve the system described at steady state to obtain expression linking the steady state concentrations of the various species. Here we use the Maple command *solve*

$assign(solve(\{dAK, dApK, dAppP2, dApP1\}, \{AK, ApK, AppP2, ApP1\})) :$
$assign(solve(\{dA, dApp\}, \{A, Ap\}))$

Simultaneously we introduce the following parameters (c_1, c_2, c_3, and c_4). This is done for the sake of brevity and easy tractability of the expressions obtained.

$$k_{b1} := c_1 \cdot (k_1 + k_{ub1}) : k_{b2} := c_2 \cdot (k_2 + k_{ub2}) : k_{b3} := c_3 \cdot (k_3 + k_{ub3}) : k_{b4} := c_4 \cdot (k_4 + k_{ub4}) :$$

Once this is done, we again solve for the steady states of the two phosphatases using the conservation expression for the enzymes (P1Con and P2Con).

$P1 := solve(P1Con, P1) :$
$P2 := solve(P2Con, P2) :$

This results in the following expressions for the steady state concentrations of the various species in terms of

the steady state substrate concentration App and K.

$$A = \frac{P1_{Total}\, k_4\, c_4\, App\, P2_{Total}\, k_3\, c_3}{\left(\frac{App\, P2_{Total}\, c_3\, c_4\, k_3}{App\, c_3 + 1} + K\, k_2\, c_2\right) K \left(App\, c_3 + 1\right) k_1\, c_1}$$

$$App = App$$

$$AK = \frac{P1_{Total}\, k_4\, c_4\, App\, P2_{Total}\, k_3\, c_3}{\left(\frac{App\, P2_{Total}\, c_3\, c_4\, k_3}{App\, c_3 + 1} + K\, k_2\, c_2\right) \left(App\, c_3 + 1\right) k_1}$$

$$ApK = \frac{App\, P2_{Total}\, k_3\, c_3}{\left(App\, c_3 + 1\right) k_2}$$

$$AppP2 = \frac{c_3\, App\, P2_{Total}}{App\, c_3 + 1}$$

$$ApP1 = \frac{c_4\, App\, P2_{Total}\, k_3\, c_3\, P1_{Total}}{\left(App\, c_3 + 1\right) \left(\frac{App\, P2_{Total}\, c_3\, c_4\, k_3}{App\, c_3 + 1} + K\, k_2\, c_2\right)}$$

Note that when App and We now have two expressions, ACon and KCon - the conservation of the substrate and kinase, whose solution for the variables define the steady state of the system.

Enzyme biphasic

In this subsection, we analytically show the absence of enzyme biphasic (in the full system). As noted earlier, the biphasic behavior is characterized by the following condition being satisfied for some steady state of the system.

$$\frac{dApp}{dK_{Total}} = 0$$

We now have two remaining conservations, KCon = 0 & ACon = 0 (see below) whose solutions to the variables App and K define the steady state of the system.

$$ACon =$$

$$\frac{P1_{Total}\, k_4\, c_4\, App\, P2_{Total}\, k_3\, c_3}{\left(\frac{App\, P2_{Total}\, c_3\, c_4\, k_3}{App\, c_3 + 1} + K\, k_2\, c_2\right) K \left(App\, c_3 + 1\right) k_1\, c_1} + \frac{App\, P2_{Total}\, k_3\, c_3}{\left(App\, c_3 + 1\right) K\, k_2\, c_2} + App$$
$$+ \frac{P1_{Total}\, k_4\, c_4\, App\, P2_{Total}\, k_3\, c_3}{\left(\frac{App\, P2_{Total}\, c_3\, c_4\, k_3}{App\, c_3 + 1} + K\, k_2\, c_2\right) \left(App\, c_3 + 1\right) k_1} + \frac{App\, P2_{Total}\, k_3\, c_3}{\left(App\, c_3 + 1\right) k_2} + \frac{c_3\, App\, P2_{Total}}{App\, c_3 + 1}$$
$$+ \frac{c_4\, App\, P2_{Total}\, k_3\, c_3\, P1_{Total}}{\left(App\, c_3 + 1\right) \left(\frac{App\, P2_{Total}\, c_3\, c_4\, k_3}{App\, c_3 + 1} + K\, k_2\, c_2\right)} - A_{Total}$$

$$KCon =$$

$$K + \frac{P1_{Total}\, k_4\, c_4\, App\, P2_{Total}\, k_3\, c_3}{\left(\frac{App\, P2_{Total}\, c_3\, c_4\, k_3}{App\, c_3 + 1} + K\, k_2\, c_2\right)\left(App\, c_3 + 1\right) k_1} + \frac{App\, P2_{Total}\, k_3\, c_3}{\left(App\, c_3 + 1\right) k_2} - K_{Total}$$

If we differentiate both these with respect to the total kinase concentration in the system, we get

$$\frac{dACon}{dK_{Total}} = 0 = \frac{\partial ACon}{\partial App} \cdot \frac{\partial App}{\partial K_{Total}} + \frac{\partial ACon}{\partial K} \cdot \frac{\partial K}{\partial K_{Total}}$$
$$\frac{dKCon}{dK_{Total}} = 0 = \frac{\partial KCon}{\partial K_{Total}} + \frac{\partial KCon}{\partial App} \cdot \frac{\partial App}{\partial K_{Total}} + \frac{\partial KCon}{\partial K} \cdot \frac{\partial K}{\partial K_{Total}}$$

Now in order to show the absence of an enzyme biphasic response we use a proof by contradiction. We begin with the assumption that there exista a biphasic beahvior. In which case there should exist a steady state of the system where $\frac{dApp}{dK_{Total}} = 0$ is satisfied.

At this point then, the above expressions simply as follows

$$0 = \frac{\partial ACon}{\partial K} \cdot \frac{\partial K}{\partial K_{Total}}$$
$$1 = \frac{\partial KCon}{\partial K} \cdot \frac{\partial K}{\partial K_{Total}}$$

Note

This simplification is possible since the functions $\frac{\partial ACon}{\partial App}, \frac{\partial KCon}{\partial App}$ are finite and always have non-zero denominators (as shown below), and thus the products involving $\frac{\partial App}{\partial K_{Total}}$ can be zero.

simplify(*diff*(*ACon*, *App*)) =

$$\Big(k_2^2 c_1 \big(App^2 k_2 c_3^2 + \big((P2_{Total} + 2\, App) k_2 + P2_{Total} k_3\big) c_3 + k_2\big) K^3 k_1 (App\, c_3 + 1)^2 c_2^3 + \big(2\, K\, App^3 c_1 c_4 k_1 k_2 c_3^2 + App\, \big(\big(\big(4\, App\, c_4 k_1 + c_4 k_1 P1_{Total} + 2\, c_4 k_1 P2_{Total} + k_4 P1_{Total} c_4 + k_1\big) k_2 + 2\, P2_{Total} c_4 k_1 k_3\big) c_1 K + k_4 P1_{Total} c_4 k_2\big) c_3 + k_2 \big(c_1 \big(2\, App\, c_4 k_1 + c_4 k_1 P1_{Total} + k_4 P1_{Total} c_4 + k_1\big) K + k_4 P1_{Total} c_4\big)\big) k_3 k_2 P2_{Total} K c_3 (App\, c_3 + 1) c_2^2 + App\, k_3^2 \big(App^3 c_4 k_2 c_3^2 + App\, \big(\big(2\, App\, c_4 + c_4 P2_{Total} + 2\big) k_2 + P2_{Total} c_4 k_3\big) c_3 + k_2 (App\, c_4 + 2)\big) c_4 P2_{Total}^2 c_1 K k_1 c_3^2 c_2 + App^2 c_1 c_3^3 c_4^2 k_1 k_3^3 P2_{Total}^3\Big) \Big/ \Big(c_2 k_2 c_1 K k_1 \big(App\, P2_{Total} c_3 c_4 k_3 + K k_2 c_2 (App\, c_3 + 1)\big)^2 (App\, c_3 + 1)^2\Big)$$

simplify(*diff*(*KCon*, *App*)) =

$$\Big(c_3 P2_{Total} k_3 \big(App^2 \big(K c_2 (K c_2 k_1 + k_4 P1_{Total} c_4) k_2^2 + 2\, K\, P2_{Total} c_2 c_4 k_1 k_3 k_2 + P2_{Total}^2 c_4^2 k_1 k_3^2\big) c_3^2 + 2\, App\, \big((K c_2 k_1 + k_4 P1_{Total} c_4) k_2 + P2_{Total} c_4 k_1 k_3\big) c_2 K k_2 c_3 + K c_2 (K c_2 k_1 + k_4 P1_{Total} c_4) k_2^2\big)\Big) \Big/ \Big((App\, c_3 + 1)^2 k_1 \big(App\, (K k_2 c_2 + P2_{Total} c_4 k_3) c_3 + K k_2 c_2\big)^2 k_2\Big)$$

We know that the denominator of $\frac{\partial KCon}{\partial K}$ is non-zero (see below), thus this implies that $\frac{\partial K}{\partial K_{Total}}$ has to

be non-zero in order to satisfy the second expression above.

$$simplify(diff(KCon, K)) = \frac{1}{k_1 \left(App\, P2_{Total}\, c_3\, c_4\, k_3 + K\, k_2\, c_2 \left(App\, c_3 + 1\right)\right)^2} \left(K^2 k_1 k_2^2 \left(App\, c_3 + 1\right)^2 c_2^2 + 2\, c_3\, P2_{Total}\, App \left(App\, c_3 + 1\right) k_3 \left(K\, k_1 - \frac{P1_{Total}\, k_4}{2}\right) k_2\, c_4\, c_2 + App^2 c_3^2 c_4^2 k_1 k_3^2 P2_{Total}^2\right)$$

This insight then informs us that in order to satisfy the first expression, $\frac{\partial ACon}{\partial K}$ must be equal to zero.

$$Condition := simplify(diff(ACon, K))$$

$$Condition := -\left(App\, k_3 \left(k_2^2 K \left(c_1 \left(P1_{Total} \left(k_4 + k_1\right) c_4 + k_1\right) K + 2\, k_4\, P1_{Total}\, c_4\right) \left(App\, c_3 + 1\right)^2 c_2^2 + 2\, App \left(K\, c_1\, k_1 + \frac{k_4\, P1_{Total}\, c_4}{2}\right) k_3\, k_2\, c_4\, P2_{Total}\, c_3 \left(App\, c_3 + 1\right) c_2 + App^2 c_1\, c_3^2 c_4^2 k_1 k_3^2 P2_{Total}^2\right) P2_{Total}\, c_3\right) \Big/ \left(c_2\, k_2\, c_1\, K^2 k_1 \left(App\, P2_{Total}\, c_3\, c_4\, k_3 + K\, k_2\, c_2 \left(App\, c_3 + 1\right)\right)^2 \left(App\, c_3 + 1\right)\right) \qquad \textbf{(1.1)}$$

However, from above we can see that this is not possible as the expression is always negative irrespective of choice of parameters or steady state concentrations of App and K.

Thus we have a contradiction, indicating that the system is incapable of exhibiting enzyme biphasic response in steady state concentration of App with total kinase concentration.

Substrate biphasic

As seen in the main text, substrate biphasic response is need possible in the ordered double site with common kinase and separate phosphatase. In this section we illustrate analytically the features of such biphasic responses and characterize the kinetic parameter dependency of the behavior.

In order to do this, we begin with the assumption that there exists a substrate biphasic response in App. i. e. there exists a steady state of the system where $\frac{dApp}{dA_{Total}} = 0$ is satisfied.

We now have two remaining conservations, KCon = 0 & ACon = 0 (see below) whose solutions to the variables App and K define the steady state of the system.

$$ACon = \frac{P1_{Total}\, k_4\, c_4\, App\, P2_{Total}\, k_3\, c_3}{\left(\frac{App\, P2_{Total}\, c_3\, c_4\, k_3}{App\, c_3 + 1} + K\, k_2\, c_2\right) K \left(App\, c_3 + 1\right) k_1\, c_1} + \frac{App\, P2_{Total}\, k_3\, c_3}{\left(App\, c_3 + 1\right) K\, k_2\, c_2} + App + \frac{P1_{Total}\, k_4\, c_4\, App\, P2_{Total}\, k_3\, c_3}{\left(\frac{App\, P2_{Total}\, c_3\, c_4\, k_3}{App\, c_3 + 1} + K\, k_2\, c_2\right) \left(App\, c_3 + 1\right) k_1} + \frac{App\, P2_{Total}\, k_3\, c_3}{\left(App\, c_3 + 1\right) k_2} + \frac{c_3\, App\, P2_{Total}}{App\, c_3 + 1}$$

$$+ \frac{c_4 App\, P2_{Total} k_3 c_3 P1_{Total}}{\left(App\, c_3 + 1\right)\left(\frac{App\, P2_{Total} c_3 c_4 k_3}{App\, c_3 + 1} + K k_2 c_2\right)} - A_{Total}$$

$$KCon = K + \frac{P1_{Total} k_4 c_4 App\, P2_{Total} k_3 c_3}{\left(\frac{App\, P2_{Total} c_3 c_4 k_3}{App\, c_3 + 1} + K k_2 c_2\right)\left(App\, c_3 + 1\right) k_1} + \frac{App\, P2_{Total} k_3 c_3}{\left(App\, c_3 + 1\right) k_2} - K_{Total}$$

If we differentiate both these with respect to the total substrate concentration in the system, we get

$$\frac{dACon}{dA_{Total}} = 0 = \frac{\partial ACon}{\partial A_{Total}} + \frac{\partial ACon}{\partial App} \cdot \frac{\partial App}{\partial A_{Total}} + \frac{\partial ACon}{\partial K} \cdot \frac{\partial K}{\partial A_{Total}}$$

$$\frac{dKCon}{dA_{Total}} = 0 = \frac{\partial KCon}{\partial App} \cdot \frac{\partial App}{\partial A_{Total}} + \frac{\partial KCon}{\partial K} \cdot \frac{\partial K}{\partial A_{Total}}$$

At this point then, the above expressions simply as follows (evaluated at the biphasic peak where $\frac{dApp}{dA_{Total}} = 0$

$$1 = \frac{\partial ACon}{\partial K} \cdot \frac{\partial K}{\partial A_{Total}}$$

$$0 = \frac{\partial KCon}{\partial K} \cdot \frac{\partial K}{\partial A_{Total}}$$

Note

This simplification is possible since the functions $\frac{\partial ACon}{\partial App}, \frac{\partial KCon}{\partial App}$ are finite and always have non-zero denominators (as shown below), and thus the products involving $\frac{\partial App}{\partial A_{Total}}$ can be zero.

simplify(*diff*(*ACon*, *App*)) =

$$\Big(\big(App^2 k_2 c_3^2 + \big(\big(P2_{Total} + 2\, App\big) k_2 + P2_{Total} k_3\big) c_3 + k_2\big) k_1 c_1 k_2^2 K^3 \left(App\, c_3 + 1\right)^2 c_2^3 + \Big(2\, K App^3 c_1 c_4 k_1 k_2 c_3^2 + App\, \Big(\Big(\big(\big(4\, App\, c_4 k_1 + c_4 k_1 P1_{Total} + 2\, c_4 k_1 P2_{Total} + k_4 P1_{Total} c_4 + k_1\big) k_2 + 2\, P2_{Total} c_4 k_1 k_3\big) c_1 K + k_4 P1_{Total} c_4 k_2\Big) c_3 + \big(c_1 \big(2\, App\, c_4 k_1 + c_4 k_1 P1_{Total} + k_4 P1_{Total} c_4 + k_1\big) K + k_4 P1_{Total} c_4\big) k_2\Big) k_3 P2_{Total} c_3 k_2 K \left(App\, c_3 + 1\right) c_2^2 + App\, k_3^2 k_1 P2_{Total}^{\;2} c_3^2 c_1 K \Big(App^3 c_4 k_2 c_3^2 + App\, \big(\big(2\, App\, c_4 + c_4 P2_{Total} + 2\big) k_2 + P2_{Total} c_4 k_3\big) c_3 + k_2 \left(App\, c_4 + 2\right)\Big) c_4 c_2 + App^2 c_1 c_3^3 c_4^2 k_1 k_3^3 P2_{Total}^{\;3}\Big) \Big/ \Big(k_1 c_1 k_2 \left(App\, P2_{Total} c_3 c_4 k_3 + K k_2 c_2 \left(App\, c_3 + 1\right)\right)^2 K \left(App\, c_3 + 1\right)^2 c_2\Big)$$

simplify(*diff*(*KCon*, *App*)) =

$$\Big(P2_{Total} \Big(App^2 \big(K c_2 \left(K c_2 k_1 + k_4 P1_{Total} c_4\right) k_2^2 + 2\, K P2_{Total} c_2 c_4 k_1 k_3 k_2 + P2_{Total}^{\;2} c_4^2 k_1 k_3^2\big) c_3^2 + 2\, App\, k_2 c_2 \big(\big(\left(K c_2 k_1 + k_4 P1_{Total} c_4\right) k_2 + P2_{Total} c_4 k_1 k_3\big) K c_3 + K c_2 \left(K c_2 k_1 + k_4 P1_{Total} c_4\right) k_2^2\big) k_3 c_3\Big) \Big/ \Big(\left(App\, c_3 + 1\right)^2 \left(App\, \left(K k_2 c_2 + P2_{Total} c_4 k_3\right) c_3 + K k_2 c_2\right)^2 k_2 k_1\Big)$$

We know that the denominator of $\frac{\partial ACon}{\partial K}$ is non-zero (see below), thus this implies that $\frac{\partial K}{\partial A_{Total}}$ has to be non-zero in order to satisfy the first expression above.

$simplify(diff(ACon, K))$

$$-\left(App\, k_3 \left(k_2^2 K (App\, c_3 + 1)^2 \left(\left(P1_{Total} (k_4 + k_1)\, c_4 + k_1\right) c_1 K + 2\, k_4 P1_{Total} c_4\right) c_2^2 + 2\, App\, k_3\, P2_{Total}\, c_3\, k_2\, c_4 \left(K c_1 k_1 + \frac{k_4 P1_{Total} c_4}{2}\right) (App\, c_3 + 1)\, c_2 + App^2 c_1 c_3^2 c_4^2 k_1 k_3^2 P2_{Total}^2\right) P2_{Total}\, c_3\right) \Big/ \left(k_1 c_1 k_2 \left(App\, P2_{Total}\, c_3\, c_4\, k_3 + K k_2 c_2 (App\, c_3 + 1)\right)^2 K^2 (App\, c_3 + 1)\, c_2\right) \tag{2.1}$$

Hence in order for the biphasic response to exist, $\frac{\partial KCon}{\partial K}$ has to be zero. This expression is shown below (stored as the variable *Condition*).

$Condition := simplify(diff(KCon, K))$

$$Condition := \left(K^2 k_1 k_2^2 (App\, c_3 + 1)^2 c_2^2 + 2\, App\, (App\, c_3 + 1)\, P2_{Total} \left(K k_1 - \frac{P1_{Total} k_4}{2}\right) k_2 c_4 k_3 c_3 c_2 + App^2 c_3^2 c_4^2 k_1 k_3^2 P2_{Total}^2\right) \Big/ \left(\left(App\, P2_{Total}\, c_3\, c_4\, k_3 + K k_2 c_2 (App\, c_3 + 1)\right)^2 k_1\right) \tag{2.2}$$

Now writing the numerator of *Conditon* as a polynomial in K (as shown below), reveals that irrespective of the steady state concentrations of App the parameter values, the coeffecient of the first and second exponent of K are positive.

$collect(numer(Condition), K)$

$$\left(App^2 c_2^2 c_3^2 k_1 k_2^2 + 2\, App\, c_2^2 c_3 k_1 k_2^2 + c_2^2 k_1 k_2^2\right) K^2 + \left(2\, App^2 c_2 c_3^2 c_4 k_1 k_2 k_3 P2_{Total} + 2\, App\, c_2 c_3 c_4 k_1 k_2 k_3 P2_{Total}\right) K - App^2 c_2 c_3^2 c_4 k_2 k_3 k_4 P1_{Total} P2_{Total} + App^2 c_3^2 c_4^2 k_1 k_3^2 P2_{Total}^2 - P1_{Total} k_4 c_4\, App\, P2_{Total} k_3 c_3 k_2 c_2 \tag{2.3}$$

The constant term however depending on the concentration of App can be negative or positive. Now this polynomial (*Condition*) has to be zero for the biphasic to exist, implying the constant term has to necessarily be negative (which will guarentee the existence of a positive concentration of K that is a root of the polynomial). This requirement of negativity for the constant term provides us with the following condition involving the concentration of App

$App < solve\left(-App^2 c_2 c_3^2 c_4 k_2 k_3 k_4 P1_{Total} P2_{Total} + App^2 c_3^2 c_4^2 k_1 k_3^2 P2_{Total}^2 - P1_{Total} k_4 c_4\, App\, P2_{Total} k_3 c_3 k_2 c_2, App\right)[2]$

$$App < -\frac{c_2 k_2 k_4 P1_{Total}}{c_3 \left(c_2 k_2 k_4 P1_{Total} - P2_{Total} c_4 k_1 k_3\right)} \tag{2.4}$$

Depending on whether $-c_2 k_2 k_4 P1_{Total} + P2_{Total} c_4 k_1 k_3$ is positive or negative, this condition is trvially satified or isn't. In addition to kinetic constants, the sign of this grouping can also be manipulated by

suitable choices of total phosphatase concentrations ($P1_{Total}$ & $P2_{Total}$).
Now, if thiss grouping ($-c_2 k_2 k_4 P1_{Total} + P2_{Total} c_4 k_1 k_3$) is negative, then the constant term is negative irrespective of the steady state concentration of App and thus the whole expression from earlier guarentees substrate biphasic response at some total concentration. However, if the grouping ($-c_2 k_2 k_4 P1_{Total} + P2_{Total} c_4 k_1 k_3$) is positive, then the constant term can still be negative, however in this case App (at the biphasic peak) is necessarily to be less than

$$\frac{c_2 k_2 k_4 P1_{Total}}{c_3 \left(-c_2 k_2 k_4 P1_{Total} + P2_{Total} c_4 k_1 k_3\right)}.$$

This is however still feasible since, App and epsilon can take any positive concentration and we can find a suitable total concentration of substrate and enzyme that will accommodate it as steady state concentrations (using ACon and KCon which we are yet to solve).

$$ACon = \frac{P1_{Total} k_4 c_4 App\, P2_{Total} k_3 c_3}{\left(\frac{App\, P2_{Total} c_3 c_4 k_3}{App\, c_3 + 1} + K k_2 c_2\right) K \left(App\, c_3 + 1\right) k_1 c_1} + \frac{App\, P2_{Total} k_3 c_3}{\left(App\, c_3 + 1\right) K k_2 c_2} + App$$
$$+ \frac{P1_{Total} k_4 c_4 App\, P2_{Total} k_3 c_3}{\left(\frac{App\, P2_{Total} c_3 c_4 k_3}{App\, c_3 + 1} + K k_2 c_2\right) \left(App\, c_3 + 1\right) k_1} + \frac{App\, P2_{Total} k_3 c_3}{\left(App\, c_3 + 1\right) k_2} + \frac{c_3 App\, P2_{Total}}{App\, c_3 + 1}$$
$$+ \frac{c_4 App\, P2_{Total} k_3 c_3 P1_{Total}}{\left(App\, c_3 + 1\right) \left(\frac{App\, P2_{Total} c_3 c_4 k_3}{App\, c_3 + 1} + K k_2 c_2\right)} - A_{Total}$$

$$KCon = K + \frac{P1_{Total} k_4 c_4 App\, P2_{Total} k_3 c_3}{\left(\frac{App\, P2_{Total} c_3 c_4 k_3}{App\, c_3 + 1} + K k_2 c_2\right) \left(App\, c_3 + 1\right) k_1} + \frac{App\, P2_{Total} k_3 c_3}{\left(App\, c_3 + 1\right) k_2} - K_{Total}$$

Thus, what this implies is that, irrespective of the underlying grouping (or the sign of the catalytic constant grouping $-c_2 k_2 k_4 P1_{Total} + P2_{Total} c_4 k_1 k_3$), substrate biphasic behavior is guaranteed to exist for some total concentration of substrate and enzyme in the system.

However, we wish to note here that depending on the sign of the catalytic constant grouping $-c_2 k_2 k_4 P1_{Total} + P2_{Total} c_4 k_1 k_3$ it is either more likely or less likely to observe substrate biphasic response in App with total amount of substrate in the system. Further, depending on the sign of the catalytic constant grouping (if $-c_2 k_2 k_4 P1_{Total} + P2_{Total} c_4 k_1 k_3 > 0$), the peak concentration of the biphasic response capable of being observed is capped at $\frac{c_2 k_2 k_4 P1_{Total}}{c_3 \left(-c_2 k_2 k_4 P1_{Total} + P2_{Total} c_4 k_1 k_3\right)}$ which is not the case when ($-c_2 k_2 k_4 P1_{Total} + P2_{Total} c_4 k_1 k_3 < 0$) when App can take any value at biphasic peak (given that the total amounts are completely flexible.

Additionally, now we show how the parameter values used to generate main text figure 2, satisfy the requirements.

$k_1 := 3.5 : k_2 := 50 : k_3 := 100 : k_4 := 100 :$
$k_{b1} := 20 : k_{b2} := 75 : k_{b3} := 50 : k_{b4} := 30 :$
$k_{ub1} := 1 : k_{ub2} := 1 : k_{ub3} := 1 : k_{ub4} := 1 : P1_{Total} := 1 : P2_{Total} := 1 :$

$$c_1 := \frac{k_{b1}}{k_1 + k_{ub1}} : c_2 := \frac{k_{b2}}{k_2 + k_{ub2}} : c_3 := \frac{k_{b3}}{k_3 + k_{ub3}} : c_4 := \frac{k_{b4}}{k_4 + k_{ub4}} :$$

$App := 2 :$
$K := solve(Condition)[1] = 2.195303469$

$ACon = 6.526495239 - A_{Total}$
$KCon = 5.586607136 - K_{Total}$

Double site phosphorylation system (DSP)
[separate kinase and common phosphatase]
Features and requirements of obtaining biphasic response in the maximally modified substrate

In this file we analytically study the absence of enzyme (with $K1_{Total}$) and substrate biphasic in the maximally modified substrate form. We also show the presence of enzyme biphasic (with K2Total) and the discern the kinetic conditions required to enable the behavior in the system. In doing so we establish the following key results with regard to the behavior.

Enzyme biphasic response (biphasic behavior in the dose response curve of App as $K1_{Total}$ changes)

1. The system is incapable of exhibiting enzyme biphasic dose response with increasing amounts of total kinase K1.

Enzyme biphasic response (biphasic behavior in the dose response curve of App as $K2_{Total}$ changes)

1. Presence of enzyme biphasic dose response with increasing amounts of total kinase K2, and the necessary conditions (kinetic constraints) to enable the behavior.

Substrate biphasic response (biphasic behavior in the dose response curve of App as A_{Total} changes)

1. Substrate biphasic response is impossible in App for any kinetic regime (i.e. for any choice of underlying kinetics the system is incapable of exhibiting substrate biphasic dose response at some total concentration of enzymes)

We note that the key signature of biphasic behavior in the dose response curve of the system is the presence of a steady state of the system that satisfies the following condition.

$$\frac{dApp}{dK_{Total}} = 0 \text{ (for enzyme biphasic)}$$

$$\frac{dApp}{dA_{Total}} = 0 \text{ (for substrate biphasic)}$$

Model DSP with separate kinase and common phosphatse: We first describe the model of double site phosphorylation with different kinase and common phosphatase acting on each modification site.

We initialize the *Maple* file with the *restart* command and load the relevant libraries of inbuilt *Maple* functions (*LinearAlgebra, VectorCalculus, Student[LinearAlgebra]*)

$restart : with(LinearAlgebra) : with(VectorCalculus) : with(Student[LinearAlgebra]) :$

The system is modeled as a set of ODEs using the kinetic nomenclature described in the main text. Here dA represents d[A]/dt and similarly in the case of the other variables. At steady state thus, the right hand sides of each of these expressions will be equal to zero.

$dA := k_4 \cdot ApP + k_{ub1} \cdot AK1 - k_{b1} \cdot A \cdot K1 :$
$dAp := k_1 \cdot AK1 + k_3 \cdot AppP + k_{ub2} \cdot (ApK2) + k_{ub4} \cdot (ApP) - k_{b2} \cdot (Ap) \cdot (K2) - k_{b4} \cdot Ap \cdot P :$
$dApp := k_2 \cdot ApK2 + k_{ub3} \cdot AppP - k_{b3} \cdot App \cdot P :$

$dAK1 := k_{b1} \cdot A \cdot K1 - (k_{ub1} + k_1) \cdot AK1 :$
$dApK2 := k_{b2} \cdot Ap \cdot K2 - (k_{ub2} + k_2) \cdot ApK2 :$
$dAppP := k_{b3} \cdot App \cdot P - (k_{ub3} + k_3) \cdot AppP :$
$dApP := k_{b4} \cdot Ap \cdot P - (k_{ub4} + k_4) \cdot ApP :$

$dK1 := -k_{b1} A\, K1 + (k_{ub1} + k_1)\, AK1 :$
$dK2 := -k_{b2} Ap\, K2 + (k_{ub2} + k_2)\, ApK2 :$
$dP := -k_{b3} App\, P + (k_{ub3} + k_3)\, AppP - k_{b4} Ap\, P + (k_{ub4} + k_4)\, ApP :$

The model is also associated with conservation conditions which are described below. Here we store the conservation expressions as ACon, PCon, K1Con and K2Con for the substrate and the respective enzymes. Each of these expressions is always equal to zero (both in the transient and at steady state).

$ACon := A + Ap + App + AK1 + ApK2 + AppP + ApP - A_{Total} :$
$PCon := P + AppP + ApP - P_{Total} :$
$K1Con := K1 + AK1 - K1_{Total} :$
$K2Con := K2 + ApK2 - K2_{Total} :$

We now solve the system described at steady state to obtain expression linking the steady state concentrations of the various species. Here we use the Maple command *solve* to solve the equations for a given variable as shown below. We pursue this to finally obtain the steady state concentrations of most species in terms of App , K1 and a ratio ϵ (defined below).

$$AK1 := solve(dAK1, AK1) = \frac{k_{b1}\, A\, K1}{k_{ub1} + k_1}$$
$assign(solve(\{dApK2, dAppP, dApP\}, \{ApK2, AppP, ApP\}))$
$assign(solve(\{dA, dAp\}, \{A, Ap\}))$

Further we now introduce a ratio, ϵ = K2/P (defined as the ratio of the free enzymes). Simultaneously we introduce the following parameters (c_1, c_2, c_3, and c_4). This is done for the sake of brevity and easy tractability of the expressions obtained.

$k_{b1} := c_1 \cdot (k_1 + k_{ub1}) : k_{b2} := c_2 \cdot (k_2 + k_{ub2}) : k_{b3} := c_3 \cdot (k_3 + k_{ub3}) : k_{b4} := c_4 \cdot (k_4 + k_{ub4}) :$

$K2 := \text{epsilon} \cdot P :$

Once this is done, we again solve for the steady state of the phosphatase using the conservation expression for the enzyme (PCon).

$$P := simplify(solve(PCon, P)) = \frac{P_{Total} \epsilon k_2 c_2}{c_2 k_2 (App\, c_3 + 1) \epsilon + c_4 App\, k_3 c_3}$$

This results in the following expressions for the steady state concentrations of the various species in terms of the steady state substrate concentration App and ϵ

$$A := simplify(A) = \frac{App\, P_{Total} k_3 k_4 c_3 c_4}{(c_2 k_2 (App\, c_3 + 1) \epsilon + c_4 App\, k_3 c_3)\, K1\, k_1 c_1}$$

$$Ap = \frac{App\, k_3 c_3}{\epsilon k_2 c_2}$$

$$AK1 = \frac{App\, P_{Total} k_3 k_4 c_3 c_4}{(c_2 k_2 (c_3 App + 1) \epsilon + c_4 App\, k_3 c_3)\, k_1}$$

$$ApK2 = \frac{c_2 App\, k_3 c_3 \epsilon P_{Total}}{c_2 k_2 (c_3 App + 1) \epsilon + c_4 App\, k_3 c_3}$$

$$AppP = \frac{c_3 App\, P_{Total} \epsilon k_2 c_2}{c_2 k_2 (c_3 App + 1) \epsilon + c_4 App\, k_3 c_3}$$

$$ApP = \frac{c_4 App\, k_3 c_3 P_{Total}}{c_2 k_2 (c_3 App + 1) \epsilon + c_4 App\, k_3 c_3}$$

Note that when App, K1 ϵ are positive, steady state concentrations of the other variable concentrations are positive as well. Thus we have solved the system of equations at steady state to arrive at expressions linking the steady state concentrations of the variables with that of App, K1 and ϵ. We now have three expressions, ACon, K1Con and K2Con - the conservation of the substrate and kinases, whose solution for the variables define the steady state of the system.

$$ACon = \frac{App\, P_{Total} k_3 k_4 c_3 c_4}{(c_2 k_2 (c_3 App + 1) \epsilon + c_4 App\, k_3 c_3)\, K1\, k_1 c_1} + \frac{App\, k_3 c_3}{\epsilon k_2 c_2} + App + \frac{App\, P_{Total} k_3 k_4 c_3 c_4}{(c_2 k_2 (c_3 App + 1) \epsilon + c_4 App\, k_3 c_3)\, k_1} + \frac{c_2 App\, k_3 c_3 \epsilon P_{Total}}{c_2 k_2 (c_3 App + 1) \epsilon + c_4 App\, k_3 c_3} + \frac{c_3 App\, P_{Total} \epsilon k_2 c_2}{c_2 k_2 (c_3 App + 1) \epsilon + c_4 App\, k_3 c_3} + \frac{c_4 App\, k_3 c_3 P_{Total}}{c_2 k_2 (c_3 App + 1) \epsilon + c_4 App\, k_3 c_3} - A_{Total}$$

$$K1Con = K1 + \frac{App\, P_{Total} k_3 k_4 c_3 c_4}{(c_2 k_2 (c_3 App + 1) \epsilon + c_4 App\, k_3 c_3)\, k_1} - K1_{Total}$$

$$K2Con = \frac{\epsilon^2 P_{Total} k_2 c_2}{c_2 k_2 (c_3 App + 1) \epsilon + c_4 App\, k_3 c_3} + \frac{c_2 App\, k_3 c_3 \epsilon P_{Total}}{c_2 k_2 (c_3 App + 1) \epsilon + c_4 App\, k_3 c_3} - K2_{Total}$$

Substrate biphasic (Absence)

Now in order to show the absence of substrate biphasic response in the maximally modified substrate form (App) with changing A_{Total}, we use a proof by contradiction. Thus we begin with the assumption that substrate biphasic exists, meaning there exists a steady state of the system where $\frac{\partial App}{\partial A_{Total}}$ equals zero.

Now if we were to differentiate ACon, K1Con and K2Con with A_{Total}, we obtain the following (note that K2Con is a function of App and ϵ only)

$$\frac{dACon}{dA_{Total}} = 0 = \frac{\partial ACon}{\partial A_{Total}} + \frac{\partial ACon}{\partial App} \cdot \frac{\partial App}{\partial A_{Total}} + \frac{\partial ACon}{\partial \epsilon} \cdot \frac{\partial \epsilon}{\partial A_{Total}} + \frac{\partial ACon}{\partial K1} \cdot \frac{\partial K1}{\partial A_{Total}}$$

$$\frac{dK1Con}{dA_{Total}} = 0 = \frac{\partial K1Con}{\partial K1} \cdot \frac{\partial K1}{\partial A_{Total}} + \frac{\partial K1Con}{\partial App} \cdot \frac{\partial App}{\partial A_{Total}} + \frac{\partial K1Con}{\partial \epsilon} \cdot \frac{\partial \epsilon}{\partial A_{Total}}$$

$$\frac{dK2Con}{dA_{Total}} = 0 = \frac{\partial K2Con}{\partial App} \cdot \frac{\partial App}{\partial A_{Total}} + \frac{\partial K2Con}{\partial \epsilon} \cdot \frac{\partial \epsilon}{\partial A_{Total}}$$

Now, since we assume that there exists substrate biphasic, we can simplify these expressions further as shown below

$$1 = \frac{\partial ACon}{\partial \epsilon} \cdot \frac{\partial \epsilon}{\partial A_{Total}} + \frac{\partial ACon}{\partial K1} \cdot \frac{\partial K1}{\partial A_{Total}}$$

$$0 = \frac{\partial K1}{\partial A_{Total}} + \frac{\partial K1Con}{\partial \epsilon} \cdot \frac{\partial \epsilon}{\partial A_{Total}}$$

$$0 = \frac{\partial K2Con}{\partial \epsilon} \cdot \frac{\partial \epsilon}{\partial A_{Total}}$$

Note

This simplification is possible since the functions $\frac{\partial ACon}{\partial App}$, $\frac{\partial K2Con}{\partial App}$ and $\frac{\partial K1Con}{\partial App}$ are finite and always have non-zero denominators (as shown below), and thus the products involving $\frac{\partial App}{\partial A_{Total}}$ can be zero.

simplify(*diff*(*ACon*, *App*))

$$\Big(k_2^2\, K1\, c_2^3\, k_1 \Big(\Big(\big(1 + App^2 c_3^2 + (P_{Total} + 2\, App)\, c_3\big)\, k_2 + P_{Total}\, c_3\, k_3\Big)\, c_1\, \epsilon^3 + k_3\, k_2^2\, c_2^2\, c_3 \Big(K1\, App^2\, c_1\, c_3^2\, k_1 + 2\, K1\, App\, c_1\, k_1\, (App\, c_4 + 1)\, c_3 + K1\, \Big(\big((2\, App\, c_4 + P_{Total}\, c_4 + 1)\, k_1 + c_4\, k_4\, P_{Total}\big)\, c_1 + c_4\, k_4\, P_{Total}\Big)\Big)\, \epsilon^2 + 2\, c_4\, k_3^2\, k_2\, K1 \left(App\, c_3 + \frac{1}{2}\, App\, c_4 + 1\right) App\, c_2\, k_1\, c_1\, c_3^2\, \epsilon + App^2\, K1\, c_1\, c_3^3\, c_4^2\, k_1\, k_3^3\Big) \Big/ \Big(\epsilon\, k_2\, K1\, \big(c_2\, k_2\, (App\, c_3 + 1)\, \epsilon + c_4\, App\, k_3\, c_3\big)^2\, c_2\, k_1\, c_1\Big) \tag{1.1.1}$$

simplify(*diff*(*K2Con*, *App*)) =

$$simplify(diff(K1Con, App)) = -\frac{P_{Total}\left(\left(\epsilon k_2 - k_3\right) c_2 + c_4 k_3\right) c_2 \epsilon^2 c_3 k_2}{\left(c_2 k_2 \left(App\, c_3 + 1\right) \epsilon + c_4 App\, k_3 c_3\right)^2} \frac{P_{Total} k_3 k_4 c_3 c_4 c_2 \epsilon k_2}{\left(c_2 k_2 \left(App\, c_3 + 1\right) \epsilon + c_4 App\, k_3 c_3\right)^2 k_1}$$

We know that the denominator of $\frac{\partial K2Con}{\partial \epsilon}$ is non-zero (see below), thus this implies that $\frac{\partial \epsilon}{\partial A_{Total}}$ has to be zero in order to satisfy the first expression above.

$$simplify(diff(K2Con, \epsilon)) = \frac{\left(\epsilon^2 k_2^2 c_2 \left(App\, c_3 + 1\right) + 2\, App\, c_3 c_4 \epsilon k_2 k_3 + App^2 c_3^2 c_4 k_3^2\right) P_{Total} c_2}{\left(c_2 k_2 \left(App\, c_3 + 1\right) \epsilon + c_4 App\, k_3 c_3\right)^2}$$

This insight then informs us that in order to satisfy the second expression, $\frac{\partial K1}{\partial A_{Total}}$ must be equal to zero. Again this simplification is possible since the denominator of $\frac{\partial K1Con}{\partial \epsilon}$ is non-zero (see below).

$$simplify(diff(K1Con, \text{epsilon})) = -\frac{App\, P_{Total} k_3 k_4 c_3 c_4 c_2 k_2 \left(App\, c_3 + 1\right)}{\left(c_2 k_2 \left(App\, c_3 + 1\right) \epsilon + c_4 App\, k_3 c_3\right)^2 k_1}$$

Put together, the fact that $\frac{\partial K1}{\partial A_{Total}}$ and $\frac{\partial \epsilon}{\partial A_{Total}}$ are both zero provides a contradiction with the requirements of the first expression (obtained upon differentiation of the total substrate concentration equation).

Note

This assertion is possible since the denominators of $\frac{\partial ACon}{\partial K1}$ and $\frac{\partial ACon}{\partial \epsilon}$ are finite and always have non-zero denominators (as shown below),

$$simplify(diff(ACon, K1)) = -\frac{App\, P_{Total} k_3 k_4 c_3 c_4}{K1^2 \left(c_2 k_2 \left(App\, c_3 + 1\right) \epsilon + c_4 App\, k_3 c_3\right) k_1 c_1}$$

$$simplify(diff(ACon, \text{epsilon})) = -\Big(k_3 App \Big(k_2 c_2^2 \Big(\Big(K1 \left(App^2 k_1 c_3^2 + App \left(c_4 k_4 P_{Total} + 2 k_1\right) c_3 + P_{Total} \left(k_4 + k_1\right) c_4 + k_1\right) c_1 + k_4 P_{Total} c_4 \left(App\, c_3 + 1\right)\Big)\Big) k_2 - K1\, P_{Total} App\, c_1 c_3 c_4 k_1 k_3\Big) \epsilon^2 + 2\, K1\, App\, c_1 c_2 c_3 c_4 k_1 k_2 k_3 \left(App\, c_3 + 1\right) \epsilon + App^2 K1\, c_1 c_3^2 c_4^2 k_1 k_3^2\Big) c_3\Big) \Big/ \left(\epsilon^2 k_2 K1 \left(c_2 k_2 \left(App\, c_3 + 1\right) \epsilon + c_4 App\, k_3 c_3\right)^2 c_2 k_1 c_1\right)$$

Thus we have a contradiction, indicating that the system is incapable of exhibiting substrate biphasic response in steady state concentration of App with total substrate concentration.

Biphasic with K1-Total (Absence)

Now in order to show the absence of enzyme biphasic response in the maximally modified substrate form (App) with changing $K1_{Total}$, we use a proof by contradiction. Thus we begin with the assumption

that enzyme biphasic exists, meaning there exists a steady state of the system where $\frac{\partial App}{\partial K1_{Total}}$ equals zero.

Now if we were to differentiate ACon, K1Con and K2Con with $K1_{Total}$, we obtain the following (note that K2Con is a function of App and ϵ only)

$$\frac{dACon}{dK1_{Total}} = 0 = \frac{\partial ACon}{\partial App} \cdot \frac{\partial App}{\partial K1_{Total}} + \frac{\partial ACon}{\partial \epsilon} \cdot \frac{\partial \epsilon}{\partial K1_{Total}} + \frac{\partial ACon}{\partial K1} \cdot \frac{\partial K1}{\partial K1_{Total}}$$
$$\frac{dK1Con}{dK1_{Total}} = 0 = \frac{\partial K1Con}{\partial K1_{Total}} + \frac{\partial K1Con}{\partial K1} \cdot \frac{\partial K1}{\partial K1_{Total}} + \frac{\partial K1Con}{\partial App} \cdot \frac{\partial App}{\partial K1_{Total}} + \frac{\partial K1Con}{\partial \epsilon} \cdot \frac{\partial \epsilon}{\partial K1_{Total}}$$
$$\frac{dK2Con}{dK1_{Total}} = 0 = \frac{\partial K2Con}{\partial App} \cdot \frac{\partial App}{\partial K1_{Total}} + \frac{\partial K2Con}{\partial \epsilon} \cdot \frac{\partial \epsilon}{\partial K1_{Total}}$$

Now, since we assume that there exists substrate biphasic, we can simplify these expressions further as shown below

$$0 = \frac{\partial ACon}{\partial \epsilon} \cdot \frac{\partial \epsilon}{\partial K1_{Total}} + \frac{\partial ACon}{\partial K1} \cdot \frac{\partial K1}{\partial K1_{Total}}$$
$$1 = \frac{\partial K1}{\partial K1_{Total}} + \frac{\partial K1Con}{\partial \epsilon} \cdot \frac{\partial \epsilon}{\partial K1_{Total}}$$
$$0 = \frac{\partial K2Con}{\partial \epsilon} \cdot \frac{\partial \epsilon}{\partial K1_{Total}}$$

Note

This simplification is possible since the functions $\frac{\partial ACon}{\partial App}$, $\frac{\partial K2Con}{\partial App}$ and $\frac{\partial K1Con}{\partial App}$ are finite and always have non-zero denominators (as shown below), and thus the products involving $\frac{\partial App}{\partial K1_{Total}}$ can be zero.

simplify(*diff*(*ACon*, *App*))

$$\Big(k_2^2\, K1\, c_2^3\, k_1\, \big(\big(\big(1 + App^2\, c_3^2 + \big(P_{Total} + 2\, App\big)\, c_3\big)\, k_2 + P_{Total}\, c_3\, k_3\big)\, c_1\, \epsilon^3 + k_3\, k_2^2\, c_2^2\, c_3\, \big(K1\, App^2\, c_1\, c_3^2\, k_1 + 2\, K1\, App\, c_1\, k_1\, \big(App\, c_4 + 1\big)\, c_3 + K1\, \big(\big(2\, App\, c_4 + P_{Total}\, c_4 + 1\big)\, k_1 + c_4\, k_4\, P_{Total}\big)\, c_1 + c_4\, k_4\, P_{Total}\big)\, \epsilon^2 + 2\, c_4\, k_3^2\, k_2\, K1\, \Big(App\, c_3 + \frac{1}{2}\, App\, c_4 + 1\Big)\, App\, c_2\, k_1\, c_1\, c_3^2\, \epsilon + App^2\, K1\, c_1\, c_3^3\, c_4^2\, k_1\, k_3^3\Big) \Big/ \Big(\epsilon\, k_2\, K1\, \big(c_2\, k_2\, \big(App\, c_3 + 1\big)\, \epsilon + c_4\, App\, k_3\, c_3\big)^2\, c_2\, k_1\, c_1\Big) \tag{2.1.1}$$

$$simplify(diff(K2Con, App)) = -\frac{P_{Total}\, \big(\big(\epsilon\, k_2 - k_3\big)\, c_2 + c_4\, k_3\big)\, c_2\, \epsilon^2\, c_3\, k_2}{\big(c_2\, k_2\, \big(App\, c_3 + 1\big)\, \epsilon + c_4\, App\, k_3\, c_3\big)^2}$$

simplify(*diff*(*K1Con*, *App*)) =

$$\frac{P_{Total}\, k_3\, k_4\, c_3\, c_4\, c_2\, \epsilon\, k_2}{\left(c_2\, k_2\, \left(App\, c_3 + 1\right)\, \epsilon + c_4\, App\, k_3\, c_3\right)^2\, k_1}$$

Now from equation 3 above (obtained from differentiating K2Con), we can observe that $\frac{\partial \epsilon}{\partial K1_{Total}}$ is zero, (since $\frac{\partial K2Con}{\partial \epsilon}$ cannot be equal to zero, see note below).

This implies that (from analyzing equation 1 above, obtained from differentiating ACon), $\frac{\partial K1}{\partial K1_{Total}}$ is also equal to zero since $\frac{\partial ACon}{\partial \epsilon}$ is finite and has a non-zero denominator, and $\frac{\partial ACon}{\partial K1}$ is non-zero always (see note below).

Note

$$simplify(diff(K2Con, \text{epsilon})) = \frac{c_2\, \left(\epsilon^2\, k_2^2\, c_2\, \left(App\, c_3 + 1\right) + 2\, App\, c_3\, c_4\, \epsilon\, k_2\, k_3 + App^2\, c_3^2\, c_4\, k_3^2\right)\, P_{Total}}{\left(c_2\, k_2\, \left(App\, c_3 + 1\right)\, \epsilon + c_4\, App\, k_3\, c_3\right)^2}$$

$$simplify(diff(ACon, K1)) = -\frac{App\, P_{Total}\, k_3\, k_4\, c_3\, c_4}{c_1\, k_1\, \left(c_2\, k_2\, \left(App\, c_3 + 1\right)\, \epsilon + c_4\, App\, k_3\, c_3\right)\, K1^2}$$

$$simplify(diff(ACon, \text{epsilon})) = -\Big(k_3\, App\, c_3\, \Big(\Big(\Big(K1\, \left(App^2\, k_1\, c_3^2 + App\, \left(c_4\, k_4\, P_{Total} + 2\, k_1\right)\, c_3 + P_{Total}\, \left(k_4 + k_1\right)\, c_4 + k_1\right)\, c_1 + k_4\, P_{Total}\, c_4\, \left(App\, c_3 + 1\right)\Big)\, k_2 - K1\, P_{Total}\, App\, c_1\, c_3\, c_4\, k_1\, k_3\Big)\, k_2\, c_2^2\, \epsilon^2 + 2\, K1\, App\, c_1\, c_2\, c_3\, c_4\, k_1\, k_2\, k_3\, \left(App\, c_3 + 1\right)\, \epsilon + App^2\, K1\, c_1\, c_3^2\, c_4^2\, k_1\, k_3^2\Big)\Big) \Big/ \left(c_1\, \epsilon^2\, k_1\, \left(c_2\, k_2\, \left(App\, c_3 + 1\right)\, \epsilon + c_4\, App\, k_3\, c_3\right)^2\, K1\, k_2\, c_2\right)$$

Thus, from the above inferences ($\frac{\partial K1}{\partial K1_{Total}}$ and $\frac{\partial \epsilon}{\partial K1_{Total}}$ are both zero), we find that a contradiction in equation 2 (obtained from differentiating K1Con)

Thus contradiction. The conditions can't be satisfied implying that a biphasic response in App is not possible with total enzyme concentration $K1_{Total}$.

Biphasic with K2-Total (Presence)

In this subsection, we show the presence of enzyme biphasic response in the maximally modified substrate form (App) with changing $K2_{Total}$. We begin with the assumption that enzyme biphasic exists, meaning there exists a steady state of the system where $\frac{\partial App}{\partial K1_{Total}}$ equals zero, and show how the system can permit this provided a given kinetic condition is satisfied.

Now if we were to differentiate ACon, K1Con and K2Con with $K2_{Total}$, we obtain the following (note that K2Con is a function of App and ϵ only)

$$\frac{dACon}{dK2_{Total}} = 0 = \frac{\partial ACon}{\partial App} \cdot \frac{\partial App}{\partial K2_{Total}} + \frac{\partial ACon}{\partial \epsilon} \cdot \frac{\partial \epsilon}{\partial K2_{Total}} + \frac{\partial ACon}{\partial K1} \cdot \frac{\partial K1}{\partial K2_{Total}}$$

$$\frac{dK1Con}{dK2_{Total}} = 0 = \frac{\partial K1Con}{\partial K2_{Total}} + \frac{\partial K1Con}{\partial K1} \cdot \frac{\partial K1}{\partial K2_{Total}} + \frac{\partial K1Con}{\partial App} \cdot \frac{\partial App}{\partial K2_{Total}} + \frac{\partial K1Con}{\partial \epsilon} \cdot \frac{\partial \epsilon}{\partial K2_{Total}}$$

$$\frac{dK2Con}{dK2_{Total}} = 0 = \frac{\partial K2Con}{\partial App} \cdot \frac{\partial App}{\partial K2_{Total}} + \frac{\partial K2Con}{\partial \epsilon} \cdot \frac{\partial \epsilon}{\partial K2_{Total}}$$

Now, since we assume that there exists substrate biphasic, we can simplify these expressions further as shown below

$$0 = \frac{\partial ACon}{\partial \epsilon} \cdot \frac{\partial \epsilon}{\partial K2_{Total}} + \frac{\partial ACon}{\partial K1} \cdot \frac{\partial K1}{\partial K2_{Total}}$$

$$0 = \frac{\partial K1}{\partial K2_{Total}} + \frac{\partial K1Con}{\partial \epsilon} \cdot \frac{\partial \epsilon}{\partial K2_{Total}}$$

$$1 = \frac{\partial K2Con}{\partial \epsilon} \cdot \frac{\partial \epsilon}{\partial K2_{Total}}$$

Note

This simplification is possible since the functions $\frac{\partial ACon}{\partial App}$, $\frac{\partial K2Con}{\partial App}$ and $\frac{\partial K1Con}{\partial App}$ are finite and always have non-zero denominators (as shown below), and thus the products involving $\frac{\partial App}{\partial K2_{Total}}$ can be zero.

simplify(*diff* (*ACon*, *App*))

$$\Big(k_2^2 c_1 k_1 c_2^3 K1 \left(\left(1 + App^2 c_3^2 + \left(P_{Total} + 2\, App\right) c_3\right) k_2 + P_{Total} c_3 k_3\right) \epsilon^3 + c_3 k_2^2 k_3 c_2^2 \Big(K1\, App^2 c_1 c_3^2 k_1 + 2\, c_1 k_1\, App\, K1 \left(App\, c_4 + 1\right) c_3 + \left(\left(2\, App\, c_4 + c_4 P_{Total} + 1\right) k_1 + k_4 P_{Total} c_4\right) K1\, c_1 + k_4 P_{Total} c_4\Big) \epsilon^2 + 2\, c_3^2 k_2\, App\, c_1 k_1 \left(c_3\, App + \frac{1}{2}\, App\, c_4 + 1\right) k_3^2 c_2 K1\, c_4 \epsilon + App^2 K1\, c_1 c_3^3 c_4^2 k_1 k_3^3\Big) \Big/ \left(k_2 c_1 k_1 \left(c_2 k_2 \left(c_3\, App + 1\right) \epsilon + c_4\, App\, k_3 c_3\right)^2 c_2\, K1\, \epsilon\right) \quad \textbf{(3.1.1)}$$

$$simplify(diff(K2Con, App)) = -\frac{k_2 P_{Total} c_2 c_3 \epsilon^2 \left(\left(\epsilon k_2 - k_3\right) c_2 + c_4 k_3\right)}{\left(c_2 k_2 \left(c_3\, App + 1\right) \epsilon + c_4\, App\, k_3 c_3\right)^2}$$

$$simplify(diff(K1Con, App)) = \frac{P_{Total} k_3 k_4 c_3 c_4 \epsilon k_2 c_2}{k_1 \left(c_2 k_2 \left(c_3\, App + 1\right) \epsilon + c_4\, App\, k_3 c_3\right)^2}$$

We know that the denominator of $\frac{\partial K2Con}{\partial \epsilon}$ is non-zero (see below), thus this implies that $\frac{\partial \epsilon}{\partial K2_{Total}}$ has to be non-zero in order to satisfy the third expression above, i.e. $\frac{\partial \epsilon}{\partial K2_{Total}} = \frac{\partial K2Con}{\partial \epsilon} \neq 0$

$simplify(diff(K2Con, \text{epsilon})) =$

$$\frac{\left(\epsilon^2 k_2^2 c_2 (c_3 App + 1) + 2\,App\,c_3 c_4 \epsilon k_2 k_3 + App^2 c_3^2 c_4 k_3^2\right) P_{Total}\, c_2}{\left(c_2 k_2 (c_3 App + 1)\,\epsilon + c_4 App\, k_3 c_3\right)^2}$$

Similarly from the first expression we can discern that $\frac{\partial K1}{\partial K2_{Total}} = -\frac{\partial K1Con}{\partial \epsilon} \cdot \frac{\partial \epsilon}{\partial K2_{Total}}$. Resubstituting this in the first expression above yeilds,

$$0 = \frac{\partial ACon}{\partial \epsilon} \cdot \frac{\partial \epsilon}{\partial K2_{Total}} + \frac{\partial ACon}{\partial K1} \cdot -\frac{\partial K1Con}{\partial \epsilon} \cdot \frac{\partial \epsilon}{\partial K2_{Total}}$$

which further simplifies to

$$0 = \frac{\partial \epsilon}{\partial K2_{Total}} \cdot \left(\frac{\partial ACon}{\partial \epsilon} - \frac{\partial ACon}{\partial K1} \cdot \frac{\partial K1Con}{\partial \epsilon} \right)$$

Now as mentioned earlier, $\frac{\partial \epsilon}{\partial K2_{Total}} \neq 0$. Thus, in order for the above expression to be satisfied, $\left(\frac{\partial ACon}{\partial \epsilon} - \frac{\partial ACon}{\partial K1} \cdot \frac{\partial K1Con}{\partial \epsilon} \right) = 0$

We evaluate the expression, and store the numerator of the expression below as T.

$T := collect(simplify(numer(diff(ACon, \text{epsilon}) - diff(ACon, K1) \cdot diff(K1Con, \text{epsilon}))), K1)$

$$\begin{aligned} T := &-App\, k_3 c_3 \left(k_2^2 k_1 (c_3 App + 1) c_2^3 \left(\left(c_1 \left(App^2 c_3^2 + 2 c_3 App + c_4 P_{Total} + 1\right) k_1 \right.\right.\right. \\ &\left.\left.\left. + k_4 P_{Total} c_4 (c_3 App + 1) c_1\right) k_2 - P_{Total} App\, c_1 c_3 c_4 k_1 k_3\right) \epsilon^3 \right. \\ &+ c_3 k_2 App\, k_3 c_2^2 \left(\left(c_1 \left(3 App^2 c_3^2 + 6 c_3 App + c_4 P_{Total} + 3\right) k_1^2 + k_4 P_{Total} c_4 (c_3 App \right.\right. \\ &\left.\left. + 1) c_1 k_1\right) k_2 - P_{Total} App\, c_1 c_3 c_4 k_1^2 k_3\right) c_4 \epsilon^2 + 3 App^2 c_1 c_2 c_3^2 c_4^2 k_1^2 k_2 k_3^2 (c_3 App \\ &\left. + 1)\,\epsilon + App^3 c_1 c_3^3 c_4^3 k_1^2 k_3^3\right) K1^2 - App\, k_3 c_3 \left(k_2^3 k_1 (c_3 App + 1)^2 c_2^3 k_4 P_{Total} c_4 \epsilon^3 \right. \\ &\left. + c_3 k_2^2 App\, k_3 c_2^2 k_4 P_{Total} c_4^2 (c_3 App + 1) k_1 \epsilon^2\right) K1 \\ &- App^2 P_{Total}^2 k_3^2 k_4^2 c_3^2 c_4^2 c_2^2 k_2^2 (c_3 App + 1)\,\epsilon^2 \end{aligned} \tag{3.1}$$

Collected above as a polynomial in K1, we can clearly discern the strucutre of this polynomial as a quadratic in K1. More specifically the coeffecient of the first exponent and the constant are all negative for all feasible parameter and steady state values of the variables.

Thus in order for a feasible steady state admitting a enzyme biphasic (with $K2_{Total}$) to exist, the coeffecient of the leading coeffecient must be positive for some feasible steady state concentrations and kinetic parameter values.

We isoalte this coeffecient blow and simplify it further using the inbuilt *simplify* command.

$$\begin{aligned} simplify\Big(&-App\, k_3 c_3 \left(k_2^2 k_1 (c_3 App + 1) c_2^3 \left(\left(c_1 \left(App^2 c_3^2 + 2 c_3 App + c_4 P_{Total} + 1\right) k_1 \right.\right.\right. \\ &\left.\left. + k_4 P_{Total} c_4 (c_3 App + 1) c_1\right) k_2 - P_{Total} App\, c_1 c_3 c_4 k_1 k_3\right) \epsilon^3 \\ &+ c_3 k_2 App\, k_3 c_2^2 \left(\left(c_1 \left(3 App^2 c_3^2 + 6 c_3 App + c_4 P_{Total} + 3\right) k_1^2 + k_4 P_{Total} c_4 (c_3 App \right.\right. \\ &\left.\left. + 1) c_1 k_1\right) k_2 - P_{Total} App\, c_1 c_3 c_4 k_1^2 k_3\right) c_4 \epsilon^2 + 3 App^2 c_1 c_2 c_3^2 c_4^2 k_1^2 k_2 k_3^2 (c_3 App + 1)\,\epsilon \end{aligned}$$

$$+ App^3 c_1 c_3^3 c_4^3 k_1^2 k_3^3))$$

$$-\left(c_2 k_2 \left(c_3 App + 1\right) \epsilon + c_4 App\, k_3 c_3\right) k_3 c_1 c_3 App \left(c_2^2 k_2 \left(\left(App^2 c_3^2 k_1 + App \left(k_4 P_{Total} c_4 + 2 k_1\right) c_3 + P_{Total} \left(k_1 + k_4\right) c_4 + k_1\right) k_2 - App\, P_{Total} c_3 c_4 k_1 k_3\right) \epsilon^2 + 2\, App\, c_2 c_3 c_4 k_1 k_2 k_3 \left(c_3 App + 1\right) \epsilon + App^2 c_3^2 c_4^2 k_1 k_3^2\right) k_1 \quad \mathbf{(3.2)}$$

As we can see the coeffecient factors in to the product of two expressions. The leading exponent is always negative ($-\left(c_2 k_2 \left(c_3 App + 1\right) \epsilon + c_4 App\, k_3 c_3\right) k_3 c_1 c_3 App$). Thus this implies that there must exist a feasible steady state concentration when the second expression in this factorization must be negative (to make the whole coeffecient positive).

Isolating and simplifying this as a polynomial in P_{Total} further yeilds the following

$$collect\left(c_2^2 \left(\left(App^2 c_3^2 k_1 + App \left(k_4 P_{Total} c_4 + 2 k_1\right) c_3 + P_{Total} \left(k_1 + k_4\right) c_4 + k_1\right) k_2 - App\, P_{Total} c_3 c_4 k_1 k_3\right) k_2 \epsilon^2 + 2\, App\, c_2 c_3 c_4 k_1 k_2 k_3 \left(App\, c_3 + 1\right) \epsilon + App^2 c_3^2 c_4^2 k_1 k_3^2, P_{Total}\right)$$

$$c_2^2 \left(\left(App\, c_4 k_4 c_3 + \left(k_1 + k_4\right) c_4\right) k_2 - App\, c_3 c_4 k_1 k_3\right) k_2 \epsilon^2 P_{Total} + c_2^2 \left(App^2 c_3^2 k_1 + 2\, App\, k_1 c_3 + k_1\right) k_2^2 \epsilon^2 + 2\, App\, c_2 c_3 c_4 k_1 k_2 k_3 \left(App\, c_3 + 1\right) \epsilon + App^2 c_3^2 c_4^2 k_1 k_3^2 \quad \mathbf{(3.3)}$$

Here again we can observe that the expression is a linear expression in P_{Total}, with the constant term (or terms independent of P_{Total}) are all positive for feasible values. The coeffecient of P_{Total} however can be negative. Isolating this further,

$$\left(collect\left(\left(App\, c_4 k_4 c_3 + \left(k_1 + k_4\right) c_4\right) k_2 - App\, c_3 c_4 k_1 k_3, App\right)\right)$$

$$\left(-c_3 c_4 k_1 k_3 + c_4 k_4 c_3 k_2\right) App + \left(k_1 + k_4\right) c_4 k_2 \quad \mathbf{(3.4)}$$

We can see above that there exists a grouping of kinetic constants for which this expression can be negative. i.e., $-c_3 c_4 k_1 k_3 + c_4 k_4 c_3 k_2 < 0$ then there exists a sufficiently large App for which the coeffecient of P_{Total} is negative in the expressions above.

By extending that logic to the expressions and polynomials obtained earliar, for a sufficiently large P_{Total} and given App, the coeffecient of the second term in the quadratic polynomial involving K1 can be positive. This implies in turn that there exists a feasible steady state K1 where the conditions of the enzyme biphasic response (with $K2_{Total}$) are satisfied for the given kinetic parameters.

This grouping thus can be simplified as

$$simplify\left(-c_3 c_4 k_1 k_3 + c_4 k_4 c_3 k_2 < 0\right)$$

$$-c_3 c_4 \left(k_1 k_3 - k_2 k_4\right) < 0 \quad \mathbf{(3.5)}$$

or

$$simplify\left(k_1 k_3 - k_2 k_4 > 0\right)$$

$$0 < k_1 k_3 - k_2 k_4 \quad \mathbf{(3.6)}$$

Thus, should the expression above involving kinetic parameters be satisfied, there exists some total amounts of kinase (K1 & K2), phosphatase and substrate, where the enzyme biphasic requirements (with $K2_{Total}$) are satisfied and can be obtained.

Necessary condition for enzyme biphasic dose response:

$$k_1 k_3 - k_2 k_4 > 0$$

Interestingly thiss is the same kinetic constraint required to be satisfied to obtain enzyme biphasic in the ordered double site system with common kinase and common phosphatase - see section 2.1

Double site phosphorylation system (DSP)
[separate kinase and separate phosphatase]
Features and requirements of obtaining biphasic response in the maximally modified substrate

In this file we analytically prove the absence of enzyme and substrate biphasic in the maximally modified substrate form (App), with changes in $K1_{Total}$, $K2_{Total}$ and A_{Total} respectively.

We note that the key signature of biphasic behavior in the dose response curve of the system is the presence of a steady state of the system that satisfies the following condition.

$\frac{\partial App}{\partial K_{Total}} = 0$ (for enzyme biphasic, $K1_{Total}$ or $K2_{Total}$ depending on the enzyme dose considered)

$\frac{\partial App}{\partial A_{Total}} = 0$ (for substrate biphasic)

Model DSP with separate kinase and separate phosphatse: We first describe the model of double site phosphorylation with different kinase and separate phosphatase acting on each modification site.

We initialize the *Maple* file with the *restart* command and load the relevant libraries of inbuilt *Maple* functions (*LinearAlgebra, VectorCalculus, Student[LinearAlgebra]*)

$restart : with(LinearAlgebra) : with(VectorCalculus) : with(Student[LinearAlgebra]) :$

The system is modeled as a set of ODEs using the kinetic nomenclature described in the main text. Here dA represents d[A]/dt and similarly in the case of the other variables. At steady state thus, the right hand sides of each of these expressions will be equal to zero.

$$dA := -k_{b1} \cdot A \cdot K1 + k_{ub1} \cdot AK1 + k_4 \cdot ApP1 :$$
$$dApp := k_2 \cdot ApK2 - k_{b3} \cdot App \cdot P2 + k_{ub3} \cdot AppP2 :$$
$$dAp := k_1 \cdot AK1 - k_{b2} \cdot Ap \cdot K2 - k_{b4} \cdot Ap \cdot P1 + k_{ub2} \cdot ApK2 + k_{ub4} \cdot ApP1 + k_3 \cdot AppP2 :$$

$$dAK1 := k_{b1} \cdot A \cdot K1 - \left(k_{ub1} + k_1\right) \cdot AK1 :$$
$$dApK2 := k_{b2} \cdot Ap \cdot K2 - \left(k_{ub2} + k_2\right) \cdot ApK2 :$$
$$dAppP2 := k_{b3} \cdot App \cdot P2 - \left(k_{ub3} + k_3\right) \cdot AppP2 :$$
$$dApP1 := k_{b4} \cdot Ap \cdot P1 - \left(k_{ub4} + k_4\right) \cdot ApP1 :$$

$$dP1 := -k_{b4} \cdot Ap \cdot P1 + \left(k_{ub4} + k_4\right) \cdot ApP1 :$$
$$dP2 := -k_{b3} \cdot App \cdot P2 + \left(k_{ub3} + k_3\right) \cdot AppP2 :$$
$$dK1 := -k_{b1} \cdot A \cdot K1 + \left(k_{ub1} + k_1\right) \cdot AK1 :$$
$$dK2 := -k_{b2} \cdot Ap \cdot K2 + \left(k_{ub2} + k_2\right) \cdot ApK2 :$$

The model is also associated with conservation conditions which are described below. Here we store the conservation expressions as ACon, P1Con, P2Con, K1Con and K2Con for the substrate and the respective enzymes. Each of these expressions is always equal to zero (both in the transient and at steady state).

$$P1Con := P1 + ApP1 - P1_{Total} :$$
$$P2Con := P2 + AppP2 - P2_{Total} :$$
$$K1Con := K1 + AK1 - K1_{Total} :$$
$$K2Con := K2 + ApK2 - K2_{Total} :$$
$$ACon := A_{Total} - Ap - A - App - AK1 - ApK2 - AppP2 - ApP1 :$$

We now solve the system described at steady state to obtain expression linking the steady state concentrations of the various species. Here we use the Maple command *solve* to solve the equations for a given variable as shown below. We pursue this to finally obtain the steady state concentrations of most species in terms of App.

$$AK1 := solve(dAK1, AK1) = \frac{k_{b1}\, A\, K1}{k_{ub1} + k_1}$$
$$assign(solve(\{dApK2, dAppP2, dApP1\}, \{ApK2, AppP2, ApP1\})) :$$
$$assign(solve(\{dA, dAp\}, \{A, Ap\})) :$$

Simultaneously we introduce the following parameters (c_1, c_2, c_3, and c_4). This is done for the sake of brevity and easy tractability of the expressions obtained.

$k_{b1} := c_1 \cdot (k_1 + k_{ub1}) : k_{b2} := c_2 \cdot (k_2 + k_{ub2}) : k_{b3} := c_3 \cdot (k_3 + k_{ub3}) : k_{b4} := c_4 \cdot (k_4 + k_{ub4}) :$

Once this is done, we again solve for the steady state of the phosphatases using the conservation expression for the enzymes (P1Con and P2Con).

$$P2 := solve(P2Con, P2) = \frac{P2_{Total}}{App\ c_3 + 1}$$

$$P1 := solve(P1Con, P1) = \frac{P1_{Total}\,(App\ c_3 + 1)\ K2\ k_2\ c_2}{App\ K2\ c_2\ c_3\ k_2 + App\ c_3\ c_4\ k_3\ P2_{Total} + K2\ c_2\ k_2}$$

This results in the following expressions for the steady state concentrations of the various species in terms of the steady state substrate concentration App, K1 and K2.

$$A := simplify(A) = \frac{P1_{Total}\ k_4\ c_4\ c_3\ App\ P2_{Total}\ k_3}{c_1\ \left((App\ c_3 + 1)\ K2\ k_2\ c_2 + c_4\ c_3\ App\ P2_{Total}\ k_3\right)\ K1\ k_1}$$

$$Ap = \frac{c_3\ App\ P2_{Total}\ k_3}{(App\ c_3 + 1)\ K2\ k_2\ c_2}$$

$$AK1 = \frac{P1_{Total}\ k_4\ c_4\ c_3\ App\ P2_{Total}\ k_3}{\left((App\ c_3 + 1)\ K2\ k_2\ c_2 + c_4\ c_3\ App\ P2_{Total}\ k_3\right)\ k_1}$$

$$ApK2 = \frac{c_3\ App\ P2_{Total}\ k_3}{(App\ c_3 + 1)\ k_2}$$

$$AppP2 = \frac{c_3\ App\ P2_{Total}}{App\ c_3 + 1}$$

$$ApP1 = \frac{c_4\ c_3\ App\ P2_{Total}\ k_3\ P1_{Total}}{App\ K2\ c_2\ c_3\ k_2 + c_4\ c_3\ App\ P2_{Total}\ k_3 + K2\ k_2\ c_2}$$

Note that when App, K1 and K2 steady state concentrations of the other variable concentrations are positive as well. Thus we have solved the system of equations at steady state to arrive at expressions linking the steady state concentrations of the variables with that of App, K1 and K2. We now have two expressions, ACon, K1Con and K2Con - the conservation of the substrate and kinase, whose solution for the variables define the steady state of the system.

$$ACon =$$

$$A_{Total} - \frac{c_3\ App\ P2_{Total}\ k_3}{(App\ c_3 + 1)\ K2\ k_2\ c_2} - \frac{P1_{Total}\ k_4\ c_4\ c_3\ App\ P2_{Total}\ k_3}{c_1\ \left((App\ c_3 + 1)\ K2\ k_2\ c_2 + c_4\ c_3\ App\ P2_{Total}\ k_3\right)\ K1\ k_1} - App$$
$$- \frac{P1_{Total}\ k_4\ c_4\ c_3\ App\ P2_{Total}\ k_3}{\left((App\ c_3 + 1)\ K2\ k_2\ c_2 + c_4\ c_3\ App\ P2_{Total}\ k_3\right)\ k_1} - \frac{c_3\ App\ P2_{Total}\ k_3}{(App\ c_3 + 1)\ k_2} - \frac{c_3\ App\ P2_{Total}}{App\ c_3 + 1}$$
$$- \frac{c_4\ c_3\ App\ P2_{Total}\ k_3\ P1_{Total}}{App\ K2\ c_2\ c_3\ k_2 + c_4\ c_3\ App\ P2_{Total}\ k_3 + K2\ k_2\ c_2}$$

$$K1Con = K1 + \frac{P1_{Total}\ k_4\ c_4\ c_3\ App\ P2_{Total}\ k_3}{\left((App\ c_3 + 1)\ K2\ k_2\ c_2 + c_4\ c_3\ App\ P2_{Total}\ k_3\right)\ k_1} - K1_{Total}$$

$$K2Con = K2 + \frac{c_3\ App\ P2_{Total}\ k_3}{(App\ c_3 + 1)\ k_2} - K2_{Total}$$

Substrate biphasic

Now in order to show the absence of substrate biphasic response in the maximally modified substrate form (App) with changing A_{Total}, we use a proof by contradiction.

We begin with the assumption that substrate biphasic exists, meaning there exists a steady state of the system where $\frac{\partial App}{\partial A_{Total}}$ equals zero. Now if we were to differentiate ACon, K1Con and K2Con with A_{Total}, we obtain the following (note that K2Con is a function of App and K2 only).

$$\frac{dACon}{dA_{Total}} = 0 = \frac{\partial ACon}{\partial A_{Total}} + \frac{\partial ACon}{\partial App} \cdot \frac{\partial App}{\partial A_{Total}} + \frac{\partial ACon}{\partial K2} \cdot \frac{\partial K2}{\partial A_{Total}} + \frac{\partial ACon}{\partial K1} \cdot \frac{\partial K1}{\partial A_{Total}}$$

$$\frac{dK1Con}{dA_{Total}} = 0 = \frac{\partial K1Con}{\partial K1} \cdot \frac{\partial K1}{\partial A_{Total}} + \frac{\partial K1Con}{\partial App} \cdot \frac{\partial App}{\partial A_{Total}} + \frac{\partial K1Con}{\partial K2} \cdot \frac{\partial K2}{\partial A_{Total}}$$

$$\frac{dK2Con}{dA_{Total}} = 0 = \frac{\partial K2Con}{\partial App} \cdot \frac{\partial App}{\partial A_{Total}} + \frac{\partial K2Con}{\partial K2} \cdot \frac{\partial K2}{\partial A_{Total}}$$

Now, since we assume that there exists substrate biphasic, we can simplify these expressions further as shown below

$$1 = \frac{\partial ACon}{\partial K2} \cdot \frac{\partial K2}{\partial A_{Total}} + \frac{\partial ACon}{\partial K1} \cdot \frac{\partial K1}{\partial A_{Total}}$$

$$0 = \frac{\partial K1}{\partial A_{Total}} + \frac{\partial K1Con}{\partial K2} \cdot \frac{\partial K2}{\partial A_{Total}}$$

$$0 = \frac{\partial K2}{\partial A_{Total}}$$

Note

This simplification is possible since the functions $\frac{\partial ACon}{\partial App}$, $\frac{\partial K2Con}{\partial App}$ and $\frac{\partial K1Con}{\partial App}$ are finite and always has non-zero denominators (as shown below), and thus the products involving $\frac{\partial App}{\partial A_{Total}}$ can be zero.

simplify(*diff*(*ACon*, *App*))

$$\Big(-(App\, c_3+1)^2 k_2^2 k_1 c_1 K1 \big(App^2 k_2 c_3^2 + ((P2_{Total} + 2\, App) k_2 + k_3 P2_{Total}) c_3 + k_2\big) K2^3 c_2^3 - 2\,(App\, c_3 + 1) k_2 k_3 \Big(K1\, App^3 c_1 c_3^2 c_4 k_1 k_2 + \Big(\Big(\frac{K1\,(4\, App\, c_4 k_1 + c_4 k_1 P1_{Total} + 2\, c_4 k_1 P2_{Total} + k_4 P1_{Total} c_4 + k_1)\, c_1}{2} + \frac{k_4 P1_{Total} c_4}{2}\Big) k_2 + K1\, k_3 P2_{Total} c_1 c_4 k_1\Big) App\, c_3 + \frac{k_2 (K1\,(2\, App\, c_4 k_1 + c_4 k_1 P1_{Total} + k_4 P1_{Total} c_4 + k_1)\, c_1 + k_4 P1_{Total} c_4)}{2}\Big) \qquad \textbf{(1.1.1)}$$

$$c_3 P2_{Total} K2^2 c_2^2 - c_4 \left(App^3 c_4 k_2 c_3^2 + \left(\left(2\, App\, c_4 + c_4 P2_{Total} + 2\right) k_2 + k_3 P2_{Total} c_4\right) App\, c_3 + k_2 \left(App\, c_4 + 2\right)\right) k_3^2 App\, k_1 c_3^2 c_1 P2_{Total}^2 K1\, K2\, c_2 - App^2 K1\, c_1 c_3^3 c_4^2 k_1 k_3^3 P2_{Total}^3\Big) \Big/ \left(\left(App\, c_3 + 1\right)^2 k_2 \left(\left(App\, c_3 + 1\right) K2\, k_2 c_2 + c_4 c_3 App\, P2_{Total} k_3\right)^2 c_2 k_1 c_1 K1\, K2\right)$$

$$simplify(diff(K2Con, App)) = \frac{c_3 P2_{Total} k_3}{\left(App\, c_3 + 1\right)^2 k_2}$$

$$simplify(diff(K1Con, App)) = \frac{P1_{Total} k_4 c_4 c_3 P2_{Total} k_3 K2\, c_2 k_2}{\left(\left(App\, c_3 + 1\right) K2\, k_2 c_2 + c_4 c_3 App\, P2_{Total} k_3\right)^2 k_1}$$

Thus $\frac{\partial K2}{\partial A_{Total}}$ has to be zero in order to satisfy the third expression above (obtained from differentiating K2Con).

This insight then informs us that in order to satisfy the second expression (obtained from differentiating K1Con), $\frac{\partial K1}{\partial A_{Total}}$ must be equal to zero. (Again this simplification is possible since the denominator of $\frac{\partial K1Con}{\partial K2}$ is non-zero (see below).

$$simplify(diff(K1Con, K2)) = -\frac{P1_{Total} k_4 c_4 c_3 App\, P2_{Total} k_3 \left(App\, c_3 + 1\right) k_2 c_2}{\left(\left(App\, c_3 + 1\right) K2\, k_2 c_2 + c_4 c_3 App\, P2_{Total} k_3\right)^2 k_1}$$

Put together, the fact that $\frac{\partial K1}{\partial A_{Total}}$ and $\frac{\partial K2}{\partial A_{Total}}$ are both zero provides a contradiction with the requirements of the first expression (obtained upon differentiation of the total substrate concentration equation).

Note

This assertion is possible since $\frac{\partial ACon}{\partial K1}$ and $\frac{\partial ACon}{\partial K2}$ are finite and always has non-zero denominators (as shown below),.

$$simplify(diff(ACon, K1)) = \frac{P1_{Total} k_4 c_4 c_3 App\, P2_{Total} k_3}{\left(\left(App\, c_3 + 1\right) K2\, k_2 c_2 + c_4 c_3 App\, P2_{Total} k_3\right) k_1 c_1 K1^2}$$

$$simplify(diff(ACon, K2)) = \Big(\Big(\Big(\left(P1_{Total} \left(k_4 + k_1\right) c_4 + k_1\right) K1\, c_1 + k_4 P1_{Total} c_4\Big) \left(App\, c_3 + 1\right)^2 k_2^2 K2^2 c_2^2 + 2\, K1\, k_3 P2_{Total} K2\, App\, c_1 c_3 c_4 k_1 k_2 \left(App\, c_3 + 1\right) c_2 + App^2 K1\, c_1 c_3^2 c_4^2 k_1 k_3^2 P2_{Total}^2\Big) k_3 App\, c_3 P2_{Total}\Big) \Big/ \left(\left(App\, c_3 + 1\right) k_2 \left(\left(App\, c_3 + 1\right) K2\, k_2 c_2 + c_4 c_3 App\, P2_{Total} k_3\right)^2 c_2 k_1 c_1 K1\, K2^2\right)$$

Thus we have a contradiction, indicating that the system is incapable of exhibiting substrate biphasic response in steady state concentration of App with total substrate concentration.

Enzyme biphasic

Enzyme biphasic in App with changing $K1_{Total}$

Now in order to show the absence of enzyme biphasic response in the maximally modified substrate form (App) with changing $K1_{Total}$, we use a proof by contradiction.

We begin with the assumption that enzyme biphasic exists, meaning there exists a steady state of the system where $\frac{\partial App}{\partial K1_{Total}}$ equals zero. Now if we were to differentiate ACon, K1Con and K2Con with $K1_{Total}$, we obtain the following (note that K2Con is a function of App and K2 only).

$$\frac{dACon}{dK1_{Total}} = 0 = \frac{\partial ACon}{\partial App} \cdot \frac{\partial App}{\partial K1_{Total}} + \frac{\partial ACon}{\partial K2} \cdot \frac{\partial K2}{\partial K1_{Total}} + \frac{\partial ACon}{\partial K1} \cdot \frac{\partial K1}{\partial K1_{Total}}$$
$$\frac{dK1Con}{dK1_{Total}} = 0 = \frac{\partial K1\ Con}{\partial K1_{Total}} + \frac{\partial K1Con}{\partial K1} \cdot \frac{\partial K1}{\partial K1_{Total}} + \frac{\partial K1Con}{\partial App} \cdot \frac{\partial App}{\partial K1_{Total}} + \frac{\partial K1Con}{\partial K2} \cdot \frac{\partial K2}{\partial K1_{Total}}$$
$$\frac{dK2Con}{dK1_{Total}} = 0 = \frac{\partial K2Con}{\partial App} \cdot \frac{\partial App}{\partial K1_{Total}} + \frac{\partial K2Con}{\partial K2} \cdot \frac{\partial K2}{\partial K1_{Total}}$$

Now, since we assume that there exists enzyme biphasic, we can simplify these expressions further as shown below

$$0 = \frac{\partial ACon}{\partial K2} \cdot \frac{\partial K2}{\partial K1_{Total}} + \frac{\partial ACon}{\partial K1} \cdot \frac{\partial K1}{\partial K1_{Total}}$$
$$1 = \frac{\partial K1}{\partial K1_{Total}} + \frac{\partial K1Con}{\partial K2} \cdot \frac{\partial K2}{\partial K1_{Total}}$$
$$0 = \frac{\partial K2}{\partial K1_{Total}}$$

Note

This simplification is possible since the functions $\frac{\partial ACon}{\partial App}$, $\frac{\partial K2Con}{\partial App}$ and $\frac{\partial K1Con}{\partial App}$ are finite and always have a non-zero denominator (as shown below) and thus the products involving $\frac{\partial App}{\partial A_{Total}}$ can be zero.

simplify(diff(ACon, App))

$$\Big(-(App\, c_3 + 1)^2 k_2^2 k_1 c_1 K1 \left(App^2 k_2 c_3^2 + \left(\left(P2_{Total} + 2\, App\right) k_2 + k_3 P2_{Total}\right) c_3 + k_2\right) K2^3 c_2^3 - 2\left(App\, c_3 + 1\right) k_2 k_3 \Big(K1\, App^3 c_1 c_3^2 c_4 k_1 k_2 + \Big(\Big(\frac{K1\left(4\, App\, c_4 k_1 + c_4 k_1 P1_{Total} + 2\, c_4 k_1 P2_{Total} + k_4 P1_{Total} c_4 + k_1\right) c_1}{2} \tag{2.1.1.1}$$

$$+\frac{k_4 P1_{Total} c_4}{2}\Big) k_2 + K1\, k_3 P2_{Total} c_1 c_4 k_1\Big) App\, c_3$$
$$+\frac{k_2 \left(K1 \left(2\, App\, c_4 k_1 + c_4 k_1 P1_{Total} + k_4 P1_{Total} c_4 + k_1\right) c_1 + k_4 P1_{Total} c_4\right)}{2}\Bigg)$$
$$c_3 P2_{Total} K2^2 c_2^2 - c_4 \Big(App^3 c_4 k_2 c_3^2 + \big(\big(2\, App\, c_4 + c_4 P2_{Total} + 2\big) k_2$$
$$+ k_3 P2_{Total} c_4\big) App\, c_3 + k_2 \left(App\, c_4 + 2\right)\Big) k_3^2 App\, k_1 c_3^2 c_1 P2_{Total}^2 K1\, K2\, c_2$$
$$- App^2 K1\, c_1 c_3^3 c_4^2 k_1 k_3^3 P2_{Total}^3\Big) \Big/ \Big(\left(App\, c_3 + 1\right)^2 k_2 \big(\big(App\, c_3$$
$$+ 1\big) K2\, k_2 c_2 + c_4 c_3 App\, P2_{Total} k_3\big)^2 c_2 k_1 c_1 K1\, K2\Big)$$

$$simplify(diff(K2Con, App)) = \frac{c_3 P2_{Total} k_3}{\left(App\, c_3 + 1\right)^2 k_2}$$

$$simplify(diff(K1Con, App)) = \frac{P1_{Total} k_4 c_4 c_3 P2_{Total} k_3 K2\, c_2 k_2}{\left(\left(App\, c_3 + 1\right) K2\, k_2 c_2 + c_4 c_3 App\, P2_{Total} k_3\right)^2 k_1}$$

We can observe from the third expression above (obtained from differentiating K2Con), that $\frac{\partial K2}{\partial K1_{Total}}$ is equal to zero. Thus, resubstituting this in the other expressions we can further infer that, $1 = \frac{\partial K1}{\partial K1_{Total}}$ (from the second expression, obtained from differentiating K1Con).

Resubsituing this in the first expression, we get

$$0 = \frac{\partial ACon}{\partial K1}$$

Note

This simplification was possible since the denominator of $\frac{\partial ACon}{\partial K2}$ is non-zero, allowing the product with $\frac{\partial K2}{\partial K1_{Total}}$ to be zero (see below)

$simplify(diff(ACon, K2)) =$

$$\Big(App\, \big(\big(\big(P1_{Total} \left(k_4 + k_1\right) c_4 + k_1\big) K1\, c_1 + c_4 k_4 P1_{Total}\big) \left(App\, c_3 + 1\right)^2 k_2^2 K2^2 c_2^2 \quad \textbf{(2.1.2.1)}$$
$$+ 2\, K1\, k_3 P2_{Total} K2\, App\, c_1 c_3 c_4 k_1 k_2 \left(App\, c_3 + 1\right) c_2$$
$$+ App^2 K1\, c_1 c_3^2 c_4^2 k_1 k_3^2 P2_{Total}^2\big) k_3 c_3 P2_{Total}\Big) \Big/ \Big(c_2 c_1 \big(App\, c_3$$
$$+ 1\big) \left(\left(App\, c_3 + 1\right) K2\, k_2 c_2 + c_4 c_3 App\, P2_{Total} k_3\right)^2 K1\, k_1 k_2 K2^2\Big)$$

However we can quickly observe from the expression for $\frac{\partial ACon}{\partial K1}$ (see below), that it can never be equal to zero for any feasible concentration of steady state of the system (when all the variables are positive).

$simplify(diff(ACon, K1))$

$$\frac{k_3\, P2_{Total}\, App\, c_3\, k_4\, P1_{Total}\, c_4}{k_1\, c_1\, K1^2 \left(c_3 \left(c_2\, k_2\, K2_{Total} - P2_{Total}\, k_3 \left(c_2 - c_4\right)\right) App + c_2\, k_2\, K2_{Total}\right)} \quad \textbf{(2.1.1)}$$

Thus we have a contradiction, indicating that the system is incapable of exhibiting enzyme biphasic response in steady state concentration of App with total enzyme concentration ($K1_{Total}$).

Enzyme biphasic in App with changing $K2_{Total}$

Now in order to show the absence of substrate biphasic response in the maximally modified substrate form (App) with changing A_{Total}, we use a proof by contradiction.
Thus we begin with the assumption that substrate biphasic exists, meaning there exists a steady state of the system where $\frac{\partial App}{\partial A_{Total}}$ equals zero. Now if we were to differentiate ACon, K1Con and K2Con with A_{Total}, we obtain the following (note that K2Con is a function of App and K2 only).

$$\frac{dACon}{dK2_{Total}} = 0 = \frac{\partial ACon}{\partial App} \cdot \frac{\partial App}{\partial K2_{Total}} + \frac{\partial ACon}{\partial K2} \cdot \frac{\partial K2}{\partial K2_{Total}} + \frac{\partial ACon}{\partial K1} \cdot \frac{\partial K1}{\partial K2_{Total}}$$
$$\frac{dK1Con}{dK2_{Total}} = 0 = \frac{\partial K1Con}{\partial K1} \cdot \frac{\partial K1}{\partial K2_{Total}} + \frac{\partial K1Con}{\partial App} \cdot \frac{\partial App}{\partial K2_{Total}} + \frac{\partial K1Con}{\partial K2} \cdot \frac{\partial K2}{\partial K2_{Total}}$$
$$\frac{dK2Con}{dK2_{Total}} = 0 = \frac{\partial K2Con}{\partial K2_{Total}} + \frac{\partial K2Con}{\partial App} \cdot \frac{\partial App}{\partial K2_{Total}} + \frac{\partial K2Con}{\partial K2} \cdot \frac{\partial K2}{\partial K2_{Total}}$$

Now, since we assume that there exists substrate biphasic, we can simplify these expressions further as shown below

$$0 = \frac{\partial ACon}{\partial K2} \cdot \frac{\partial K2}{\partial K2_{Total}} + \frac{\partial ACon}{\partial K1} \cdot \frac{\partial K1}{\partial K2_{Total}}$$
$$0 = \frac{\partial K1}{\partial K2_{Total}} + \frac{\partial K1Con}{\partial K2} \cdot \frac{\partial K2}{\partial K2_{Total}}$$
$$1 = \frac{\partial K2}{\partial K2_{Total}}$$

Note

This simplification is possible since the functions $\frac{\partial ACon}{\partial App}$, $\frac{\partial K2Con}{\partial App}$ and $\frac{\partial K1Con}{\partial App}$ are finite and always have non-zero denominators (as shown below), and thus the products involving $\frac{\partial App}{\partial A_{Total}}$ can be zero.

simplify(*diff*(*ACon*, *App*))

$$\Big(-\left(App\, c_3 + 1\right)^2 k_2^2\, k_1\, c_1\, K1 \left(App^2\, k_2\, c_3^2 + \left(\left(P2_{Total} + 2\, App\right) k_2 + k_3\, P2_{Total}\right) c_3 + k_2\right) K2^3\, c_2^3 - 2 \left(App\, c_3 + 1\right) k_2\, k_3 \Big(K1\, App^3\, c_1\, c_3^2\, c_4\, k_1\, k_2 \quad \textbf{(2.2.1.1)}$$

$$+\left(\left(\frac{K1\left(4\,App\,c_4\,k_1+c_4\,k_1\,P1_{Total}+2\,c_4\,k_1\,P2_{Total}+k_4\,P1_{Total}\,c_4+k_1\right)c_1}{2}+\frac{k_4\,P1_{Total}\,c_4}{2}\right)k_2+K1\,k_3\,P2_{Total}\,c_1\,c_4\,k_1\right)App\,c_3+\frac{k_2\left(K1\left(2\,App\,c_4\,k_1+c_4\,k_1\,P1_{Total}+k_4\,P1_{Total}\,c_4+k_1\right)c_1+k_4\,P1_{Total}\,c_4\right)}{2}\Bigg)$$

$$c_3\,P2_{Total}\,K2^2\,c_2^2-c_4\left(App^3\,c_4\,k_2\,c_3^2+\left(\left(2\,App\,c_4+c_4\,P2_{Total}+2\right)k_2+k_3\,P2_{Total}\,c_4\right)App\,c_3+k_2\left(App\,c_4+2\right)\right)k_3^2\,App\,k_1\,c_3^2\,c_1\,P2_{Total}^2\,K1\,K2\,c_2-App^2\,K1\,c_1\,c_3^3\,c_4^2\,k_1\,k_3^3\,P2_{Total}^3\Big)\Big/\left(\left(App\,c_3+1\right)^2k_2\left(\left(App\,c_3+1\right)K2\,k_2\,c_2+c_4\,c_3\,App\,P2_{Total}\,k_3\right)^2c_2\,k_1\,c_1\,K1\,K2\right)$$

$$simplify(diff(K2Con, App))=\frac{c_3\,P2_{Total}\,k_3}{\left(App\,c_3+1\right)^2k_2}$$

$$simplify(diff(K1Con, App))=\frac{P1_{Total}\,k_4\,c_4\,c_3\,P2_{Total}\,k_3\,K2\,c_2\,k_2}{\left(\left(App\,c_3+1\right)K2\,k_2\,c_2+c_4\,c_3\,App\,P2_{Total}\,k_3\right)^2k_1}$$

This thus allows us to compute $\frac{\partial K1}{\partial K2_{Total}}$ as shown below

$$\frac{\partial K1}{\partial K2_{Total}}=-\frac{P1_{Total}\,k_4\,c_4\,c_3\,App\,P2_{Total}\,k_3\left(App\,c_3+1\right)k_2\,c_2}{\left(\left(App\,c_3+1\right)K2\,k_2\,c_2+c_4\,c_3\,App\,P2_{Total}\,k_3\right)^2k_1}$$

Substuting this into the expression obtained from differntiating ACon, we get

$$0=\frac{\partial ACon}{\partial K2}\cdot\frac{\partial K2}{\partial K2_{Total}}+\frac{\partial ACon}{\partial K1}\cdot\left(-\frac{P1_{Total}\,k_4\,c_4\,c_3\,App\,P2_{Total}\,k_3\left(App\,c_3+1\right)k_2\,c_2}{\left(\left(App\,c_3+1\right)K2\,k_2\,c_2+c_4\,c_3\,App\,P2_{Total}\,k_3\right)^2k_1}\right)$$

which is evaulated below by Maple and stored as the expression called *Condition*

$$Condition := simplify(diff(ACon, K2)-(diff(K1Con, K2))\cdot(diff(ACon, K1)))$$

$$Condition := \Big(c_3\,P2_{Total}\left(K2^3\left(K1\,c_1\left(c_4\,P1_{Total}+1\right)k_1+k_4\,P1_{Total}\,c_4\left(K1\,c_1+1\right)\right)k_2^3\,k_1\,K1\left(App\,c_3+1\right)^3c_2^3+K2^2\,k_2^2\,c_3\,P2_{Total}\left(K1^2\,c_1\left(c_4\,P1_{Total}+3\right)k_1^2+K1\,k_4\,P1_{Total}\,c_4\left(K1\,c_1+1\right)k_1+k_4^2\,P1_{Total}^2\,c_4\right)k_3\,App\left(App\,c_3+1\right)^2c_4\,c_2^2+3\,K1^2\,k_3^2\,P2_{Total}^2\,K2\,App^2\,c_1\,c_3^2\,c_4^2\,k_1^2\,k_2\left(App\,c_3+1\right)c_2+App^3\,K1^2\,c_1\,c_3^3\,c_4^3\,k_1^2\,k_3^3\,P2_{Total}^3\right)k_3\,App\Big)\Big/\left(K2^2\,k_2\,c_1\,k_1^2\,K1^2\,c_2\left(App\,c_3+1\right)\left(\left(App\,c_3+1\right)K2\,k_2\,c_2+c_4\,c_3\,App\,P2_{Total}\,k_3\right)^3\right) \qquad \textbf{(2.2.1)}$$

We can see however the expression Condition can never be equal to zero as it is a sum of positive parameters and variables K1, K2, and App, implying that the expression is purely positive for all feasible steady state concentrations.

Thus we have a contradiction, indicating that the system is incapable of exhibiting enzyme biphasic response in steady state concentration of App with total enzyme concentration ($K2_{Total}$).

Enzymatic networks beyond multisite modification networks

In this folder we detail analytical results pertaining to biphasic dose response by substrates involved in enzymatic networks beyond covalent modification networks/multisite modification of proteins, such as coupled covalent modification network and cascaded enzymatic network.

Two tier cascaded enzymatic network

[modified form of one substrate acts as a kinase modifying another substrate]

In this file, we analytically establish the impossibility of different biphasic responses in the two tier cascaded enzymatic network. In such a network, there are two covalent modification cycles (undergoing phosphorylation and dephosphorylation); wherein the dephosphorylation is effected by either a common or separate phosphatases, while a kinase effects the phosphorylation of the first tier of covalent modification (A -> Ap) and the modified form of the first tier substrate (Ap) acts as the kinase for the modification of the second tier substrate (B -> Bp); See figure 2 in the main text for schematic.

In this manuscript we establish the following results regarding biphasic response in the modified substrates to various doses (substrate/enzyme amounts).

1. Common phosphatase network
   a. Enzyme biphasic response is not possible with kinase in either of the maximally modified substrate forms (Ap or Bp)
   b. Substrate biphasic response is not possible in the modified substrate of the first tier (Ap with A_{Total})

Note: Please note that substrate-biphasic response in Bp with total substrate concentration B_{Total} is possible and is shown computationally for a parameter point in the figure 2 in the main text.

2. Separate phosphatase network
   a. Enzyme biphasic response is not possible with kinase in either of the maximally modified substrate forms (Ap or Bp)
   b. Substrate biphasic response is not possible in the modified substrate of the first tier or the second tier (Ap with A_{Total} or in Bp with B_{Total})

These results are summarized in the following tabular column.

Table 1: Substrate and Enzyme biphasic dose responses in the two tier cascaded enzymatic network

System	Substrate Biphasic	Enzyme Biphasic
Common Phosphatase	Not possible with A_{Total} Possible with B_{Total} (See figure 2)	Not possible
Separate Phosphatases	Not possible with A_{Total} or B_{Total}	Not possible

Common phosphatase model

We initialize the *Maple* file with the *restart* command and load the relevant libraries of inbuilt *Maple* functions (*LinearAlgebra, VectorCalculus, Student[LinearAlgebra]*)

$restart : with(LinearAlgebra) : with(VectorCalculus) : with(Student[LinearAlgebra]) :$

The system is modeled as a set of ODEs using the kinetic nomenclature described in the main text. Here dA represents d[A]/dt and similarly in the case of the other variables. At steady state thus, the right hand sides of each of these expressions will be equal to zero.

$$dA := k_2 \cdot ApP - k_{b1} \cdot A \cdot K + k_{ub1} \cdot AK :$$
$$dAp := k_1 \cdot AK + k_{ub2} \cdot ApP - k_{b2} \cdot Ap \cdot P - p_{b1} \cdot B \cdot Ap + (p_{ub1} + p_1) \cdot BAp :$$

$$dAK := k_{b1} \cdot A \cdot K - (k_{ub1} + k_1) \cdot AK :$$
$$dApP := k_{b2} \cdot Ap \cdot P - (k_{ub2} + k_2) \cdot ApP :$$

$$dB := -p_{b1} \cdot B \cdot Ap + p_{ub1} \cdot BAp + p_2 \cdot BpP :$$
$$dBp := p_1 \cdot BAp - p_{b2} \cdot Bp \cdot P + p_{ub2} \cdot BpP :$$

$$dBAp := p_{b1} \cdot B \cdot Ap - (p_{ub1} + p_1) \cdot BAp :$$
$$dBpP := p_{b2} \cdot Bp \cdot P - (p_{ub2} + p_2) \cdot BpP :$$

$$dK := -k_{b1} \cdot A \cdot K + (k_{ub1} + k_1) \cdot AK :$$
$$dP := -k_{b2} \cdot Ap \cdot P + (k_{ub2} + k_2) \cdot ApP - p_{b2} \cdot Bp \cdot P + (p_{ub2} + p_2) \cdot BpP :$$

The model is also associated with conservation conditions which are described below. Here we store the conservation expressions as ACon, BCon, PCon and KCon for the substrate and the respective enzymes. Each of these expressions is always equal to zero (both in the transient and at steady state).

$$ACon := A + Ap + ApP + AK + BAp - A_{Total} :$$
$$KCon := K + AK - K_{Total} :$$
$$PCon := P + ApP + BpP - P_{Total} :$$
$$BCon := B + BpP + BAp + Bp - B_{Total} :$$

Now we begin by solving the system of equations to obtain expressions linking the steady state concentrations of the variables, primarily to obtain expressions for the steady state concentrations of variables as a function of concentrations of Ap and Bp. For this purpose we use an inbuilt *Maple* command *solve* as shown with the example below.

$$AK := solve(dAK, AK) = \frac{k_{b1} A K}{k_{ub1} + k_1}$$

We similarly solve for the other variables using the same command.

$assign(solve(\{dApP, dBAp, dBpP\}, \{ApP, BAp, BpP\}))$
$assign(solve(\{dA, dB\}, \{A, B\}))$

We introduce the following parameters (c_1, c_2, d_1, and d_2). This is done for the sake of brevity and easy tractability of the expressions obtained.

$k_{b1} := c_1 \cdot (k_1 + k_{ub1}) : k_{b2} := c_2 \cdot (k_2 + k_{ub2}) :$
$p_{b1} := d_1 \cdot (p_1 + p_{ub1}) : p_{b2} := d_2 \cdot (p_2 + p_{ub2}) :$

Once this is done, we again solve for the steady state of the phosphatase using the conservation expression for the enzyme (PCon).

$$P := solve(PCon, P) = \frac{P_{Total}}{Ap\ c_2 + Bp\ d_2 + 1}$$

This results in the following expressions for the steady state concentrations of the various species in terms of the steady state substrate concentration Ap and Bp

$$A = \frac{c_2\ Ap\ P_{Total}\ k_2}{(Ap\ c_2 + Bp\ d_2 + 1)\ K k_1\ c_1}$$
$$B = \frac{d_2\ Bp\ P_{Total}\ p_2}{(Ap\ c_2 + Bp\ d_2 + 1)\ Ap\ p_1\ d_1}$$
$$AK = \frac{c_2\ Ap\ P_{Total}\ k_2}{(Ap\ c_2 + Bp\ d_2 + 1)\ k_1}$$
$$ApP = \frac{c_2\ Ap\ P_{Total}}{Ap\ c_2 + Bp\ d_2 + 1}$$
$$BAp = \frac{d_2\ Bp\ P_{Total}\ p_2}{(Ap\ c_2 + Bp\ d_2 + 1)\ p_1}$$
$$BpP = \frac{d_2\ Bp\ P_{Total}}{Ap\ c_2 + Bp\ d_2 + 1}$$
$$P = \frac{P_{Total}}{Ap\ c_2 + Bp\ d_2 + 1}$$

Note that when Ap and Bp are positive, steady state concentrations of the other variable concentrations are positive as well. Thus we have solved the system of equations at steady state to arrive at expressions linking the steady state concentrations of the variables with that of Ap and Bp. We now have three expressions, ACon, BCon and KCon - the conservation of the substrates and kinase, whose solution for the variables define the steady state of the system.

1. Absence of enzyme-biphasic dose response in the modified forms (Ap and Bp) with total kinase concentration

In this subsection we show the absence of enzyme biphasic in either of the modified forms of the substrates with total kinase amounts. As mentioned earlier, feasible solutions to the three coupled expressions (ACon, BCon, KCon - shown below) would define the steady state of the system.

ACon

$$\frac{c_2\ Ap\ P_{Total}\ k_2}{(Ap\ c_2 + Bp\ d_2 + 1)\ K k_1\ c_1} + Ap + \frac{c_2\ Ap\ P_{Total}}{Ap\ c_2 + Bp\ d_2 + 1} + \frac{c_2\ Ap\ P_{Total}\ k_2}{(Ap\ c_2 + Bp\ d_2 + 1)\ k_1} + \frac{d_2\ Bp\ P_{Total}\ p_2}{(Ap\ c_2 + Bp\ d_2 + 1)\ p_1} - A_{Total} \quad \textbf{(1.1.1)}$$

BCon

$$\frac{d_2\, Bp\, P_{Total}\, p_2}{\left(Ap\, c_2 + Bp\, d_2 + 1\right) Ap\, p_1\, d_1} + \frac{d_2\, Bp\, P_{Total}}{Ap\, c_2 + Bp\, d_2 + 1} + \frac{d_2\, Bp\, P_{Total}\, p_2}{\left(Ap\, c_2 + Bp\, d_2 + 1\right) p_1} + Bp - B_{Total} \tag{1.1.2}$$

KCon

$$K + \frac{c_2\, Ap\, P_{Total}\, k_2}{\left(Ap\, c_2 + Bp\, d_2 + 1\right) k_1} - K_{Total} \tag{1.1.3}$$

If we are to differentiate the expressions by total kinase amounts we would have the following expressions

$$\frac{dACon}{dK_{Total}} = 0 = \frac{\partial ACon}{\partial Ap} \cdot \frac{\partial Ap}{\partial K_{Total}} + \frac{\partial ACon}{\partial Bp} \cdot \frac{\partial Bp}{\partial K_{Total}} + \frac{\partial ACon}{\partial K} \cdot \frac{\partial K}{\partial K_{Total}}$$

$$\frac{dBCon}{dK_{Total}} = 0 = \frac{\partial BCon}{\partial Ap} \cdot \frac{\partial Ap}{\partial K_{Total}} + \frac{\partial BCon}{\partial Bp} \cdot \frac{\partial Bp}{\partial K_{Total}}$$

$$\frac{dKCon}{dK_{Total}} = 0 = \frac{\partial KCon}{\partial K_{Total}} + \frac{\partial KCon}{\partial K} \cdot \frac{\partial K}{\partial K_{Total}} + \frac{\partial KCon}{\partial Ap} \cdot \frac{\partial Ap}{\partial K_{Total}} + \frac{\partial KCon}{\partial Bp} \cdot \frac{\partial Bp}{\partial K_{Total}}$$

Absence of biphasic response in Ap with total kinase concentration

We first begin by showing the absence of a biphasic response in the concentration of Ap as K_{Total} changes. We show this with a proof by contradiction.

If we assume that a biphasic response exists, then there should exist a steady state of the system where the following should be satisfied (at the biphasic peak)

$$\frac{\partial Ap}{\partial K_{Total}} = 0$$

Thus, the above expressions obtained after differentiation can be simplified as follows

$$0 = \frac{\partial ACon}{\partial Bp} \cdot \frac{\partial Bp}{\partial K_{Total}} + \frac{\partial ACon}{\partial K} \cdot \frac{\partial K}{\partial K_{Total}}$$

$$0 = \frac{\partial BCon}{\partial Bp} \cdot \frac{\partial Bp}{\partial K_{Total}}$$

$$1 = \frac{\partial KCon}{\partial Bp} \cdot \frac{\partial Bp}{\partial K_{Total}} + \frac{\partial K}{\partial K_{Total}}$$

Note

This simplification is possible since the functions $\frac{\partial ACon}{\partial Ap}, \frac{\partial BCon}{\partial Ap}, \frac{\partial KCon}{\partial Ap}$ are finite and always have non-zero denominators (as shown below), and thus the products involving $\frac{\partial Ap}{\partial K_{Total}}$ can be zero.

simplify(*diff*(*ACon*, *Ap*)) =

$$\frac{1}{\left(Ap\, c_2 + Bp\, d_2 + 1\right)^2 K\, k_1\, c_1\, p_1} \left(\left(\left(Ap^2\, k_1\, c_2^{\,2} + \left(\left(P_{Total} + 2\, Ap\right) k_1 + P_{Total}\, k_2\right) \left(Bp\, d_2\right.\right.\right.\right.$$

$$+1\big)c_2 + k_1\left(Bp\,d_2+1\right)^2\big)K c_1 + P_{Total}\,c_2\,k_2\left(Bp\,d_2+1\right)\big)p_1 - d_2\,Bp\,P_{Total}\,p_2\,c_2\,K\,k_1\,c_1\big)$$

$$simplify(diff(BCon, Ap)) = -\frac{\left(c_2\,d_1\left(p_1+p_2\right)Ap^2 + 2\,p_2\,c_2\,Ap + p_2\left(Bp\,d_2+1\right)\right)d_2\,Bp\,P_{Total}}{\left(Ap\,c_2+Bp\,d_2+1\right)^2 Ap^2\,p_1\,d_1}$$

$$simplify(diff(KCon, Ap)) = \frac{P_{Total}\,c_2\,k_2\left(Bp\,d_2+1\right)}{\left(Ap\,c_2+Bp\,d_2+1\right)^2 k_1}$$

Now we can make the following inference that since $\frac{\partial BCon}{\partial Bp}$ is never zero (as shown below), that $\frac{\partial Bp}{\partial K_{Total}}$ has to be necessarily zero (at the biphasic peak).

$$simplify(diff(BCon, Bp)) = \frac{1}{\left(Ap\,c_2+Bp\,d_2+1\right)^2 Ap\,p_1\,d_1}\Bigg(Ap^3\,c_2^2\,d_1\,p_1 + 2\left(\left(\left(Bp+\frac{P_{Total}}{2}\right)p_1 + \frac{p_2\,P_{Total}}{2}\right)d_2 + p_1\right)d_1\,c_2\,Ap^2 + \left(\left(Bp^2\,d_2^2\,p_1 + \left(\left(2\,Bp+P_{Total}\right)p_1 + p_2\,P_{Total}\right)d_2 + p_1\right)d_1 + c_2\,d_2\,p_2\,P_{Total}\right)Ap + d_2\,P_{Total}\,p_2\Bigg)$$

Now since $\frac{\partial ACon}{\partial Bp}$ and $\frac{\partial KCon}{\partial Bp}$ are finite and always have non-zero denominators (as shown below)

$$simplify(diff(ACon, Bp)) = \frac{P_{Total}\,d_2\left(\left(Ap\left(\left(p_2-p_1\right)k_1 - k_2\,p_1\right)c_2 + p_2\,k_1\right)K\,c_1 - c_2\,Ap\,k_2\,p_1\right)}{\left(Ap\,c_2+Bp\,d_2+1\right)^2 K\,k_1\,c_1\,p_1}$$

$$simplify(diff(KCon, Bp)) = -\frac{c_2\,Ap\,P_{Total}\,k_2\,d_2}{\left(Ap\,c_2+Bp\,d_2+1\right)^2 k_1}$$

the differentiated expressions further simplify to

$$0 = \frac{\partial ACon}{\partial K}\cdot\frac{\partial K}{\partial K_{Total}}$$

$$0 = \frac{\partial Bp}{\partial K_{Total}}$$

$$1 = \frac{\partial K}{\partial K_{Total}}$$

This implies that $\frac{\partial ACon}{\partial K} = 0$ is a necessary condition for the biphasic behavior to exist. However we can also see that $\frac{\partial ACon}{\partial K} = 0$ is not possible for any feasible steady state of the system (see below)

$$simplify(diff(ACon, K)) = -\frac{c_2\,Ap\,P_{Total}\,k_2}{\left(Ap\,c_2+Bp\,d_2+1\right)K^2\,k_1\,c_1}$$

Thus a biphasic response in Ap cannot be possible with total kinase concentration (K_{Total}).

Absence of biphasic response in Bp with total kinase concentration

In a similar manner we can show that a biphasic response in Bp can also not exist with total kinase.

We proceed similarly, with a proof by contradiction, starting with the assumption that there exists a biphasic response in Bp with total kinase.

$$\frac{\partial Bp}{\partial K_{Total}} = 0$$

Thus, the above expressions obtained after differentiation can be simplified as follows

$$0 = \frac{\partial ACon}{\partial Ap} \cdot \frac{\partial Ap}{\partial K_{Total}} + \frac{\partial ACon}{\partial K} \cdot \frac{\partial K}{\partial K_{Total}}$$
$$0 = \frac{\partial BCon}{\partial Ap} \cdot \frac{\partial Ap}{\partial K_{Total}}$$
$$1 = \frac{\partial K}{\partial K_{Total}} + \frac{\partial KCon}{\partial Ap} \cdot \frac{\partial Ap}{\partial K_{Total}}$$

Note

This simplification is possible since the functions $\frac{\partial ACon}{\partial Bp}, \frac{\partial BCon}{\partial Bp}, \frac{\partial KCon}{\partial Bp}$ are finite and always have non-zero denominators (as shown below), and thus the products involving $\frac{\partial Bp}{\partial K_{Total}} = 0$ can be zero.

$$simplify(diff(ACon, Bp)) = \frac{P_{Total}\, d_2 \left(\left(Ap \left(\left(p_2 - p_1\right) k_1 - k_2 p_1\right) c_2 + p_2 k_1\right) K c_1 - c_2 Ap\, k_2 p_1\right)}{\left(Ap\, c_2 + Bp\, d_2 + 1\right)^2 K k_1 c_1 p_1}$$

$$simplify(diff(BCon, Bp)) = \frac{1}{\left(Ap\, c_2 + Bp\, d_2 + 1\right)^2 Ap\, p_1 d_1} \left(Ap^3 c_2^{\,2} d_1 p_1 + 2 \left(\left(\left(Bp + \frac{P_{Total}}{2}\right) p_1 + \frac{p_2 P_{Total}}{2}\right) d_2 + p_1\right) d_1 c_2 Ap^2 + \left(\left(Bp^2 d_2^{\,2} p_1 + \left(\left(2\, Bp + P_{Total}\right) p_1 + p_2 P_{Total}\right) d_2 + p_1\right) d_1 + c_2 d_2 p_2 P_{Total}\right) Ap + d_2 P_{Total}\, p_2\right)$$

$$simplify(diff(KCon, Bp)) = -\frac{c_2 Ap\, P_{Total}\, k_2 d_2}{\left(Ap\, c_2 + Bp\, d_2 + 1\right)^2 k_1}$$

Now we can make the following inference that since $\frac{\partial BCon}{\partial Ap}$ is never zero (as shown below), that $\frac{\partial Ap}{\partial K_{Total}}$ has to be necessarily zero (at the biphasic peak).

$simplify(diff(BCon, Ap)) =$

$$-\frac{\left(c_2 d_1 \left(p_1+p_2\right) Ap^2+2 p_2 c_2 Ap+p_2 \left(Bp\, d_2+1\right)\right) d_2 Bp\, P_{Total}}{\left(Ap\, c_2+Bp\, d_2+1\right)^2 Ap^2 p_1 d_1}$$

Now since $\frac{\partial ACon}{\partial Ap}$ and $\frac{\partial KCon}{\partial Ap}$ are finite and always have non-zero denominators (as shown below)

$$simplify(diff(ACon, Ap)) = \frac{1}{\left(Ap\, c_2+Bp\, d_2+1\right)^2 K k_1 c_1 p_1}\Big(\Big(\Big(\left(Ap^2 k_1 c_2^{\,2}+\left(\left(P_{Total}+2\, Ap\right) k_1+P_{Total} k_2\right) \left(Bp\, d_2+1\right)\right) c_2+k_1 \left(Bp\, d_2+1\right)^2\Big) K c_1+P_{Total} c_2 k_2 \left(Bp\, d_2+1\right)\Big) p_1 - d_2 Bp\, P_{Total} p_2 c_2 K k_1 c_1\Big)$$

$$simplify(diff(KCon, Ap)) = \frac{P_{Total} c_2 k_2 \left(Bp\, d_2+1\right)}{\left(Ap\, c_2+Bp\, d_2+1\right)^2 k_1}$$

the differentiated expressions further simplify to

$$0 = \frac{\partial ACon}{\partial K} \cdot \frac{\partial K}{\partial K_{Total}}$$

$$0 = \frac{\partial Ap}{\partial K_{Total}}$$

$$1 = \frac{\partial K}{\partial K_{Total}}$$

However we can also see that $\frac{\partial ACon}{\partial K} = 0$ is not possible for any feasible steady state of the system.

$$simplify(diff(ACon, K)) = -\frac{c_2 Ap\, P_{Total} k_2}{\left(Ap\, c_2+Bp\, d_2+1\right) K^2 k_1 c_1}$$

Thus a biphasic response in Bp cannot be possible with total kinase concentration (K_{Total}).

2. Absence of substrate-biphasic dose response in Ap with total substrate concentration (A_{Total})

In this subsection we show the absence of substrate-biphasic in the modified form of the first tier substrate (Ap) with total substrate concentration (A_{Total}). As mentioned earlier, feasible solutions to the three coupled expressions (ACon, BCon, KCon - shown below) would define the steady state of the system.

ACon

$$\frac{c_2 Ap\, P_{Total} k_2}{\left(Ap\, c_2+Bp\, d_2+1\right) K k_1 c_1}+Ap+\frac{c_2 Ap\, P_{Total}}{Ap\, c_2+Bp\, d_2+1}+\frac{c_2 Ap\, P_{Total} k_2}{\left(Ap\, c_2+Bp\, d_2+1\right) k_1}+\frac{d_2 Bp\, P_{Total} p_2}{\left(Ap\, c_2+Bp\, d_2+1\right) p_1}-A_{Total} \quad \textbf{(1.2.1)}$$

BCon

$$\frac{d_2\, Bp\, P_{Total}\, p_2}{\left(Ap\, c_2 + Bp\, d_2 + 1\right) Ap\, p_1\, d_1} + \frac{d_2\, Bp\, P_{Total}}{Ap\, c_2 + Bp\, d_2 + 1} + \frac{d_2\, Bp\, P_{Total}\, p_2}{\left(Ap\, c_2 + Bp\, d_2 + 1\right) p_1} + Bp - B_{Total} \quad \textbf{(1.2.2)}$$

KCon

$$K + \frac{c_2\, Ap\, P_{Total}\, k_2}{\left(Ap\, c_2 + Bp\, d_2 + 1\right) k_1} - K_{Total} \quad \textbf{(1.2.3)}$$

If we are to differentiate the expressions by total substrate amount A_{Total} we would have the following expressions

$$\frac{dACon}{dA_{Total}} = 0 = \frac{\partial ACon}{\partial A_{Total}} + \frac{\partial ACon}{\partial Ap} \cdot \frac{\partial Ap}{\partial A_{Total}} + \frac{\partial ACon}{\partial Bp} \cdot \frac{\partial Bp}{\partial A_{Total}} + \frac{\partial ACon}{\partial K} \cdot \frac{\partial K}{\partial A_{Total}}$$
$$\frac{dBCon}{dA_{Total}} = 0 = \frac{\partial BCon}{\partial Ap} \cdot \frac{\partial Ap}{\partial A_{Total}} + \frac{\partial BCon}{\partial Bp} \cdot \frac{\partial Bp}{\partial A_{Total}}$$
$$\frac{dKCon}{dA_{Total}} = 0 = \frac{\partial KCon}{\partial K} \cdot \frac{\partial K}{\partial A_{Total}} + \frac{\partial KCon}{\partial Ap} \cdot \frac{\partial Ap}{\partial A_{Total}} + \frac{\partial KCon}{\partial Bp} \cdot \frac{\partial Bp}{\partial A_{Total}}$$

We now show the absence of a biphasic response in the concentration of Ap with A_{Total} using a proof by contradiction.

Assuming that a biphasic response exists, there should exist a steady state of the system where the following should be satisfied (at the biphasic peak)

$$\frac{\partial Ap}{\partial A_{Total}} = 0$$

Thus, the above expressions obtained after differentiation can be simplified as follows

$$1 = \frac{\partial ACon}{\partial Bp} \cdot \frac{\partial Bp}{\partial A_{Total}} + \frac{\partial ACon}{\partial K} \cdot \frac{\partial K}{\partial A_{Total}}$$
$$0 = \frac{\partial BCon}{\partial Bp} \cdot \frac{\partial Bp}{\partial A_{Total}}$$
$$0 = \frac{\partial KCon}{\partial Bp} \cdot \frac{\partial Bp}{\partial A_{Total}} + \frac{\partial KCon}{\partial K} \cdot \frac{\partial K}{\partial A_{Total}}$$

Note

This simplification is possible since the functions $\frac{\partial ACon}{\partial Ap}, \frac{\partial BCon}{\partial Ap}, \frac{\partial KCon}{\partial Ap}$ are finite and always have non-zero denominators (as shown below), and thus the products involving $\frac{\partial Ap}{\partial A_{Total}} = 0$ can be zero.

simplify(*diff*(*ACon*, *Ap*)) =

$$\frac{1}{\left(Ap\, c_2 + Bp\, d_2 + 1\right)^2 K\, k_1\, c_1\, p_1} \Big(\Big(\Big(Ap^2\, k_1\, c_2^{\,2} + \left(Bp\, d_2 + 1\right) \left(\left(P_{Total} + 2\, Ap\right) k_1 + P_{Total}\, k_2\right) c_2 + k_1 \left(Bp\, d_2 + 1\right)^2 \Big) K\, c_1 + P_{Total}\, c_2\, k_2 \left(Bp\, d_2 + 1\right) \Big) p_1 - d_2\, Bp\, P_{Total}\, p_2\, c_2\, K\, k_1\, c_1 \Big)$$

$simplify(diff(BCon, Ap)) =$

$$-\frac{Bp\left(c_2 d_1 (p_1+p_2) Ap^2 + 2 p_2 c_2 Ap + p_2 (Bp\, d_2+1)\right) d_2 P_{Total}}{(Ap\, c_2 + Bp\, d_2 + 1)^2 Ap^2 p_1 d_1}$$

$$simplify(diff(KCon, Ap)) = \frac{P_{Total} c_2 k_2 (Bp\, d_2 + 1)}{(Ap\, c_2 + Bp\, d_2 + 1)^2 k_1}$$

Now we can make the following inference that since $\frac{\partial BCon}{\partial Bp}$ is never zero (as shown below), that $\frac{\partial Bp}{\partial A_{Total}}$ has to be necessarily zero (at the biphasic peak).

$simplify(diff(BCon, Bp))$

$$\frac{1}{(Ap\, c_2 + Bp\, d_2 + 1)^2 Ap\, p_1 d_1}\left(Ap^3 c_2^2 d_1 p_1 + 2 d_1 \left(\left(\left(Bp + \frac{P_{Total}}{2}\right) p_1 + \frac{p_2 P_{Total}}{2}\right) d_2 + p_1\right) c_2 Ap^2 + \left(\left(Bp^2 d_2^2 p_1 + \left((2\, Bp + P_{Total}) p_1 + p_2 P_{Total}\right) d_2 + p_1\right) d_1 + c_2 d_2 p_2 P_{Total}\right) Ap + d_2 P_{Total} p_2\right) \quad \textbf{(1.2.4)}$$

Now since $\frac{\partial ACon}{\partial Bp}$ and $\frac{\partial KCon}{\partial Bp}$ are finite and always have non-zero denominators (as shown below)

$simplify(diff(ACon, Bp)) =$

$$\frac{P_{Total}\left(\left(Ap\left((p_2 - p_1) k_1 - k_2 p_1\right) c_2 + p_2 k_1\right) K c_1 - c_2 Ap\, k_2 p_1\right) d_2}{(Ap\, c_2 + Bp\, d_2 + 1)^2 K k_1 c_1 p_1}$$

$$simplify(diff(KCon, Bp)) = -\frac{c_2 Ap\, P_{Total} k_2 d_2}{(Ap\, c_2 + Bp\, d_2 + 1)^2 k_1}$$

the differentiated expressions further simplify to

$$1 = \frac{\partial ACon}{\partial K} \cdot \frac{\partial K}{\partial A_{Total}}$$

$$0 = \frac{\partial Bp}{\partial A_{Total}}$$

$$0 = \frac{\partial K}{\partial A_{Total}}$$

However we now notice a contradiction, $0 = \frac{\partial K}{\partial A_{Total}}$ has to be true, while $1 = \frac{\partial ACon}{\partial K} \cdot \frac{\partial K}{\partial A_{Total}}$.

This is not possible since $\frac{\partial ACon}{\partial K}$ is finite and has a non-zero denominator (see below) and thus the product of $\frac{\partial ACon}{\partial K}$ and $\frac{\partial K}{\partial A_{Total}}$ cannot be 1, while the latter is necessarily zero.

$$simplify(diff(ACon, K)) = -\frac{c_2 Ap\, P_{Total} k_2}{(Ap\, c_2 + Bp\, d_2 + 1) K^2 k_1 c_1}$$

Thus a biphasic response in Ap is not possible with total substrate concentration A_{Total}.

3. Presence of substrate-biphasic dose response in Bp with total substrate concentration (B_{Total})

In this subsection we show the presence of substrate-biphasic in the modified form of the second tier substrate (Bp) with total substrate concentration (B_{Total}). As mentioned earlier, feasible solutions to the three coupled expressions (ACon, BCon, KCon - shown below) would define the steady state of the system.

$ACon$

$$\frac{c_2\,Ap\,P_{Total}\,k_2}{(Ap\,c_2+Bp\,d_2+1)\,K\,k_1\,c_1}+Ap+\frac{c_2\,Ap\,P_{Total}}{Ap\,c_2+Bp\,d_2+1}+\frac{c_2\,Ap\,P_{Total}\,k_2}{(Ap\,c_2+Bp\,d_2+1)\,k_1}+\frac{d_2\,Bp\,P_{Total}\,p_2}{(Ap\,c_2+Bp\,d_2+1)\,p_1}-A_{Total} \quad \textbf{(1.3.1)}$$

$BCon$

$$\frac{d_2\,Bp\,P_{Total}\,p_2}{(Ap\,c_2+Bp\,d_2+1)\,Ap\,p_1\,d_1}+\frac{d_2\,Bp\,P_{Total}}{Ap\,c_2+Bp\,d_2+1}+\frac{d_2\,Bp\,P_{Total}\,p_2}{(Ap\,c_2+Bp\,d_2+1)\,p_1}+Bp-B_{Total} \quad \textbf{(1.3.2)}$$

$KCon$

$$K+\frac{c_2\,Ap\,P_{Total}\,k_2}{(Ap\,c_2+Bp\,d_2+1)\,k_1}-K_{Total} \quad \textbf{(1.3.3)}$$

If we are to differentiate the expressions by total substrate amount B_{Total} we would have the following expressions

$$\frac{dACon}{dB_{Total}}=0=\frac{\partial ACon}{\partial B_{Total}}+\frac{\partial ACon}{\partial Ap}\cdot\frac{\partial Ap}{\partial B_{Total}}+\frac{\partial ACon}{\partial Bp}\cdot\frac{\partial Bp}{\partial B_{Total}}+\frac{\partial ACon}{\partial K}\cdot\frac{\partial K}{\partial B_{Total}}$$

$$\frac{dBCon}{dB_{Total}}=0=\frac{\partial BCon}{\partial B_{Total}}+\frac{\partial BCon}{\partial Ap}\cdot\frac{\partial Ap}{\partial B_{Total}}+\frac{\partial BCon}{\partial Bp}\cdot\frac{\partial Bp}{\partial B_{Total}}$$

$$\frac{dKCon}{dB_{Total}}=0=\frac{\partial KCon}{\partial K}\cdot\frac{\partial K}{\partial B_{Total}}+\frac{\partial KCon}{\partial Ap}\cdot\frac{\partial Ap}{\partial B_{Total}}+\frac{\partial KCon}{\partial Bp}\cdot\frac{\partial Bp}{\partial B_{Total}}$$

Now in order to show the presence of the behavior, we assume that the biphasic dose response in Bp with increasing substrate dose exists.

Thus, assuming that a biphasic response exists, there should exist a steady state of the system where the following should be satisfied (at the biphasic peak)

$$\frac{\partial Bp}{\partial B_{Total}}=0$$

Thus, the above expressions obtained after differentiation can be simplified as follows

$$0=\frac{\partial ACon}{\partial Ap}\cdot\frac{\partial Ap}{\partial B_{Total}}+\frac{\partial ACon}{\partial K}\cdot\frac{\partial K}{\partial B_{Total}}$$

$$1 = \frac{\partial BCon}{\partial Ap} \cdot \frac{\partial Ap}{\partial B_{Total}}$$

$$0 = \frac{\partial KCon}{\partial Ap} \cdot \frac{\partial Ap}{\partial B_{Total}} + 1 \cdot \frac{\partial K}{\partial B_{Total}}$$

Note

This simplification is possible since the functions $\frac{\partial ACon}{\partial Bp}, \frac{\partial BCon}{\partial Bp}, \frac{\partial KCon}{\partial Bp}$ are finite and always have non-zero denominators (as shown below), and thus the products involving $\frac{\partial Ap}{\partial B_{Total}} = 0$ can be zero.

$$simplify(diff(ACon, Bp)) = \frac{P_{Total}\left(K\left(\left(\left(p_2 - p_1\right)k_1 - k_2 p_1\right) Ap\, c_2 + p_2 k_1\right) c_1 - c_2 Ap\, k_2 p_1\right) d_2}{\left(Ap\, c_2 + Bp\, d_2 + 1\right)^2 K k_1 c_1 p_1}$$

$$simplify(diff(BCon, Bp)) = \frac{1}{\left(Ap\, c_2 + Bp\, d_2 + 1\right)^2 Ap\, p_1 d_1} \Bigg(Ap^3 c_2^2 d_1 p_1 + 2\, c_2 d_1 \left(\left(\left(Bp + \frac{P_{Total}}{2}\right) p_1 + \frac{p_2 P_{Total}}{2}\right) d_2 + p_1\right) Ap^2 + \left(\left(Bp^2 d_2^2 p_1 + \left(\left(2\, Bp + P_{Total}\right) p_1 + p_2 P_{Total}\right) d_2 + p_1\right) d_1 + c_2 d_2 p_2 P_{Total}\right) Ap + d_2 P_{Total} p_2\Bigg)$$

$$simplify(diff(KCon, Bp)) = -\frac{c_2 Ap\, P_{Total} k_2 d_2}{\left(Ap\, c_2 + Bp\, d_2 + 1\right)^2 k_1}$$

Solving the third expression for $\frac{\partial K}{\partial B_{Total}}$ and resubstituting it in the first expression leads to the following,

$$0 = \frac{\partial Ap}{\partial B_{Total}} \cdot \left(\frac{\partial ACon}{\partial Ap} - \frac{\partial ACon}{\partial K} \cdot \frac{\partial KCon}{\partial Ap}\right)$$

$$1 = \frac{\partial BCon}{\partial Ap} \cdot \frac{\partial Ap}{\partial B_{Total}}$$

Now, since $\frac{\partial BCon}{\partial Ap}$ has a non-zero denominator, $\frac{\partial Ap}{\partial B_{Total}}$ must be non-zero. This implies that the factor of terms $\left(\frac{\partial ACon}{\partial Ap} - \frac{\partial ACon}{\partial K} \cdot \frac{\partial KCon}{\partial Ap}\right)$ must be equal to zero. We group these terms as shown below in the expression T,

$$T := simplify(numer(diff(ACon, Ap) - diff(ACon, K) \cdot diff(KCon, Ap)))$$

$$T := -c_1 K^2 \left(\left(-Ap^2 c_2^2 - \left(Bp\, d_2 + 1\right)\left(P_{Total} + 2\, Ap\right) c_2 - \left(Bp\, d_2 + 1\right)^2\right) p_1 + d_2 Bp\, P_{Total} p_2 c_2\right)\left(Ap\, c_2 + Bp\, d_2 + 1\right) k_1^2 + K P_{Total} c_2 k_2 p_1 \left(Bp\, d_2 + 1\right)\left(Ap\, c_2 + Bp\, d_2 + 1\right)\left(K c_1 + 1\right) k_1 + c_2^2 Ap\, P_{Total}^2 k_2^2 \left(Bp\, d_2 + 1\right) p_1 \quad \textbf{(1.3.4)}$$

We now isolate this as a polynomial in K. As we can see the resulting expression is a quadratic in K

$collect(T, K)$

$$\left(-c_1\left(\left(-Ap^2 c_2^2 - (Bp\, d_2 + 1)(P_{Total} + 2\, Ap)\, c_2 - (Bp\, d_2 + 1)^2\right) p_1 + d_2\, Bp\, P_{Total}\, p_2\, c_2\right)(Ap\, c_2 + Bp\, d_2 + 1)\, k_1^2 + P_{Total}\, c_2\, k_2\, p_1\, (Bp\, d_2 + 1)(Ap\, c_2 + Bp\, d_2 + 1)\, c_1\, k_1\right) K^2 + P_{Total}\, c_2\, k_2\, p_1\, (Bp\, d_2 + 1)(Ap\, c_2 + Bp\, d_2 + 1)\, k_1\, K + c_2^2\, Ap\, P_{Total}^{\,2}\, k_2^2\, (Bp\, d_2 + 1)\, p_1 \tag{1.3.5}$$

The coeffecient of the first and zeroth exponent of K is always positive for any feasible kinetic condition and concentration of Ap. Thus, the sign of the leading coeffecient determines if expression T can have any feasible roots in K such that T = 0. Note that if such a root can be accomodated, the system admits to a biphasic response having satisfied the neccessary conditions for the same.

$simplify\left(-c_1\left(\left(-Ap^2 c_2^2 - (Bp\, d_2 + 1)(P_{Total} + 2\, Ap)\, c_2 - (Bp\, d_2 + 1)^2\right) p_1 + d_2\, Bp\, P_{Total}\, p_2\, c_2\right)(Ap\, c_2 + Bp\, d_2 + 1)\, k_1^2 + P_{Total}\, c_2\, k_2\, p_1\, (Bp\, d_2 + 1)(Ap\, c_2 + Bp\, d_2 + 1)\, c_1\, k_1\right)$

$$c_1\left(\left(Ap^2 c_2^2\, k_1 + 2\left(\left(Ap + \frac{P_{Total}}{2}\right) k_1 + \frac{P_{Total}\, k_2}{2}\right)(Bp\, d_2 + 1)\, c_2 + k_1\, (Bp\, d_2 + 1)^2\right) p_1 - P_{Total}\, Bp\, c_2\, d_2\, k_1\, p_2\right)(Ap\, c_2 + Bp\, d_2 + 1)\, k_1 \tag{1.3.6}$$

We now isolate the resulting expression as a function of P_{Total}.

$collect\left(\left(Ap^2 c_2^2\, k_1 + 2\left(\left(Ap + \frac{P_{Total}}{2}\right) k_1 + \frac{P_{Total}\, k_2}{2}\right)(Bp\, d_2 + 1)\, c_2 + k_1\, (Bp\, d_2 + 1)^2\right) p_1 - P_{Total}\, Bp\, c_2\, d_2\, k_1\, p_2, P_{Total}\right)$

$$\left((k_1 + k_2)(Bp\, d_2 + 1)\, c_2\, p_1 - Bp\, c_2\, d_2\, k_1\, p_2\right) P_{Total} + \left(Ap^2 c_2^2\, k_1 + 2\, k_1\, Ap\, (Bp\, d_2 + 1)\, c_2 + k_1\, (Bp\, d_2 + 1)^2\right) p_1 \tag{1.3.7}$$

We can see that the constant term here is strictly positive for all feasible concentrations of Ap and Bp, and kinetic rate constants. Thus depending on the sign of the coeffecient of P_{Total}, the expression can be negative. we bow isolate this coeffecient of PTotal as shown below

$simplify\left(collect\left((k_1 + k_2)(Bp\, d_2 + 1)\, c_2\, p_1 - Bp\, c_2\, d_2\, k_1\, p_2, Bp\right)\right)$

$$\left((k_1 + k_2)(Bp\, d_2 + 1)\, p_1 - Bp\, d_2\, k_1\, p_2\right) c_2 \tag{1.3.8}$$

Thus as seen above, if the condition below is satisfied, the coeffecient of PTotal, can be negative. Thus using this, for a sufficiently large PTotal (arbitrarily choosen), the coeffecient of the second exponent of K is negative, guaranteeing a positive feasible root in K, for all feasible concentrations of Ap and Bp. This condition is shown below.

$Condition := (p_1 - p_2)\, k_1 + k_2\, p_1 < 0$

$$Condition := (p_1 - p_2)\, k_1 + k_2\, p_1 < 0 \tag{1.3.9}$$

Thus for a substrate biphasic respose to exist in Bp with increasing BTotal, the above condition would need to be satisfied. This is also a necessary condition to obtain the behavior for some total amounts.

Different phosphatase model

We initialize the *Maple* file with the *restart* command and load the relevant libraries of inbuilt *Maple* functions (*LinearAlgebra, VectorCalculus, Student[LinearAlgebra]*)

$restart: \ with(LinearAlgebra): with(VectorCalculus): with(Student[LinearAlgebra]):$

The system is modeled as a set of ODEs using the kinetic nomenclature described in the main text. Here dA represents d[A]/dt and similarly in the case of the other variables. At steady state thus, the right hand sides of each of these expressions will be equal to zero.

$$dA := k_2 \cdot ApP - k_{b1} \cdot A \cdot K + k_{ub1} \cdot AK:$$
$$dAp := k_1 \cdot AK + k_{ub2} \cdot ApP - k_{b2} \cdot Ap \cdot P1 - p_{b1} \cdot B \cdot Ap + \left(p_{ub1} + p_1\right) \cdot BAp:$$

$$dAK := k_{b1} \cdot A \cdot K - \left(k_{ub1} + k_1\right) \cdot AK:$$
$$dApP := k_{b2} \cdot Ap \cdot P1 - \left(k_{ub2} + k_2\right) \cdot ApP:$$

$$dB := -p_{b1} \cdot B \cdot Ap + p_{ub1} \cdot BAp + p_2 \cdot BpP:$$
$$dBp := p_1 \cdot BAp - p_{b2} \cdot Bp \cdot P2 + p_{ub2} \cdot BpP:$$

$$dBAp := p_{b1} \cdot B \cdot Ap - \left(p_{ub1} + p_1\right) \cdot BAp:$$
$$dBpP := p_{b2} \cdot Bp \cdot P2 - \left(p_{ub2} + p_2\right) \cdot BpP:$$

$$dK := -k_{b1} \cdot A \cdot K + \left(k_{ub1} + k_1\right) \cdot AK:$$
$$dP1 := -k_{b2} \cdot Ap \cdot P1 + \left(k_{ub2} + k_2\right) \cdot ApP:$$
$$dP2 := -p_{b2} \cdot Bp \cdot P2 + \left(p_{ub2} + p_2\right) \cdot BpP:$$

The model is also associated with conservation conditions which are described below. Here we store the conservation expressions as ACon, BCon, PCon and KCon for the substrate and the respective enzymes. Each of these expressions is always equal to zero (both in the transient and at steady state).

$$ACon := A + Ap + ApP + AK + BAp - A_{Total}:$$
$$KCon := K + AK - K_{Total}:$$
$$P1Con := P1 + ApP - P1_{Total}:$$
$$P2Con := P2 + BpP - P2_{Total}:$$
$$BCon := B + BpP + BAp + Bp - B_{Total}:$$

Now we begin by solving the system of equations to obtain expressions linking the steady state concentrations of the variables, primarily to obtain expressions for the steady state concentrations of variables as a function of concentrations of Ap and Bp. For this purpose we use an inbuilt *Maple* command *solve* as shown with the example below.

$$AK := solve(dAK, AK) = \frac{k_{b1}\, A\, K}{k_{ub1} + k_1}$$

We similarly solve for the other variables using the same command.

$$assign(solve(\{dApP, dBAp, dBpP\}, \{ApP, BAp, BpP\}))$$
$$assign(solve(\{dA, dB\}, \{A, B\}))$$

We introduce the following parameters (c_1, c_2, d_1, and d_2). This is done for the sake of brevity and easy

tractability of the expressions obtained.

$$k_{b1} := c_1 \cdot (k_1 + k_{ub1}) : k_{b2} := c_2 \cdot (k_2 + k_{ub2}) :$$
$$p_{b1} := d_1 \cdot (p_1 + p_{ub1}) : p_{b2} := d_2 \cdot (p_2 + p_{ub2}) :$$

Once this is done, we again solve for the steady state of the phosphatase using the conservation expression for the enzyme (PCon).

$$P1 := solve(P1Con, P1) = \frac{P1_{Total}}{Ap\, c_2 + 1}$$
$$P2 := solve(P2Con, P2) = \frac{P2_{Total}}{Bp\, d_2 + 1}$$

This results in the following expressions for the steady state concentrations of the various species in terms of the steady state substrate concentration Ap and Bp

$$A = \frac{c_2\, Ap\, P1_{Total}\, k_2}{(Ap\, c_2 + 1)\, Kk_1\, c_1}$$
$$B = \frac{d_2\, Bp\, P2_{Total}\, p_2}{(Bp\, d_2 + 1)\, Ap\, p_1\, d_1}$$
$$AK = \frac{c_2\, Ap\, P1_{Total}\, k_2}{(Ap\, c_2 + 1)\, k_1}$$
$$ApP = \frac{c_2\, Ap\, P1_{Total}}{Ap\, c_2 + 1}$$
$$BAp = \frac{d_2\, Bp\, P2_{Total}\, p_2}{(Bp\, d_2 + 1)\, p_1}$$
$$BpP = \frac{d_2\, Bp\, P2_{Total}}{Bp\, d_2 + 1}$$

Note that when Ap and Bp are positive, steady state concentrations of the other variable concentrations are positive as well. Thus we have solved the system of equations at steady state to arrive at expressions linking the steady state concentrations of the variables with that of Ap and Bp. We now have three expressions, ACon, BCon and KCon - the conservation of the substrates and kinase, whose solution for the variables define the steady state of the system.

1. Absence of enzyme-biphasic dose response in the modified forms (Ap and Bp) with total kinase concentration

In this subsection we show the absence of enzyme biphasic in either of the modified forms of the substrates with total kinase amounts. As mentioned earlier, feasible solutions to the three coupled expressions (ACon, BCon, KCon - shown below) would define the steady state of the system.

ACon

$$\frac{c_2\, Ap\, P1_{Total}\, k_2}{(Ap\, c_2 + 1)\, Kk_1\, c_1} + Ap + \frac{c_2\, Ap\, P1_{Total}}{Ap\, c_2 + 1} + \frac{c_2\, Ap\, P1_{Total}\, k_2}{(Ap\, c_2 + 1)\, k_1} + \frac{d_2\, Bp\, P2_{Total}\, p_2}{(Bp\, d_2 + 1)\, p_1} \qquad \textbf{(2.1.1)}$$

$$- A_{Total}$$

$BCon$

$$\frac{d_2\, Bp\, P2_{Total}\, p_2}{(Bp\, d_2 + 1)\, Ap\, p_1\, d_1} + \frac{d_2\, Bp\, P2_{Total}}{Bp\, d_2 + 1} + \frac{d_2\, Bp\, P2_{Total}\, p_2}{(Bp\, d_2 + 1)\, p_1} + Bp - B_{Total} \tag{2.1.2}$$

$KCon$

$$K + \frac{c_2\, Ap\, P1_{Total}\, k_2}{(Ap\, c_2 + 1)\, k_1} - K_{Total} \tag{2.1.3}$$

If we are to differentiate the expressions by total kinase amounts we would have the following expressions

$$\frac{dACon}{dK_{Total}} = 0 = \frac{\partial ACon}{\partial Ap} \cdot \frac{\partial Ap}{\partial K_{Total}} + \frac{\partial ACon}{\partial Bp} \cdot \frac{\partial Bp}{\partial K_{Total}} + \frac{\partial ACon}{\partial K} \cdot \frac{\partial K}{\partial K_{Total}}$$

$$\frac{dBCon}{dK_{Total}} = 0 = \frac{\partial BCon}{\partial Ap} \cdot \frac{\partial Ap}{\partial K_{Total}} + \frac{\partial BCon}{\partial Bp} \cdot \frac{\partial Bp}{\partial K_{Total}}$$

$$\frac{dKCon}{dK_{Total}} = 0 = \frac{\partial KCon}{\partial K_{Total}} + \frac{\partial KCon}{\partial K} \cdot \frac{\partial K}{\partial K_{Total}} + \frac{\partial KCon}{\partial Ap} \cdot \frac{\partial Ap}{\partial K_{Total}} + \frac{\partial KCon}{\partial Bp} \cdot \frac{\partial Bp}{\partial K_{Total}}$$

Absence of biphasic response in Ap with total kinase concentration

We first begin by showing the absence of a biphasic response in the concentration of Ap as K_{Total} changes. We show this with a proof by contradiction.

If we assume that a biphasic response exists, then there should exist a steady state of the system where the following should be satisfied (at the biphasic peak)

$$\frac{\partial Ap}{\partial K_{Total}} = 0$$

Thus, the above expressions obtained after differentiation can be simplified as follows

$$0 = \frac{\partial ACon}{\partial Bp} \cdot \frac{\partial Bp}{\partial K_{Total}} + \frac{\partial ACon}{\partial K} \cdot \frac{\partial K}{\partial K_{Total}}$$

$$0 = \frac{\partial BCon}{\partial Bp} \cdot \frac{\partial Bp}{\partial K_{Total}}$$

$$1 = \frac{\partial KCon}{\partial Bp} \cdot \frac{\partial Bp}{\partial K_{Total}} + \frac{\partial K}{\partial K_{Total}}$$

Note

This simplification is possible since the functions $\frac{\partial ACon}{\partial Ap}, \frac{\partial BCon}{\partial Ap}, \frac{\partial KCon}{\partial Ap}$ are finite and always have non-zero denominators (as shown below), and thus the products involving $\frac{\partial Ap}{\partial K_{Total}}$ can be zero.

$simplify(diff(ACon, Ap)) =$

$$\frac{\left(Ap^2\, c_2^2\, k_1 + \left((2\, Ap + P1_{Total})\, k_1 + k_2\, P1_{Total}\right) c_2 + k_1\right) K\, c_1 + c_2\, P1_{Total}\, k_2}{(Ap\, c_2 + 1)^2\, K\, k_1\, c_1}$$

$$simplify(diff(BCon, Ap)) = -\frac{d_2\, Bp\, P2_{Total}\, p_2}{(Bp\, d_2 + 1)\, Ap^2\, p_1\, d_1}$$

$$simplify(diff(KCon, Ap)) = \frac{c_2\, P1_{Total}\, k_2}{(Ap\, c_2 + 1)^2\, k_1}$$

Now we can make the following inference that since $\frac{\partial BCon}{\partial Bp}$ is never zero (as shown below), that $\frac{\partial Bp}{\partial K_{Total}}$ has to be necessarily zero (at the biphasic peak).

$$simplify(diff(BCon, Bp)) = \frac{(Bp^2\, d_2^2\, p_1 + ((2\, Bp + P2_{Total})\, p_1 + p_2\, P2_{Total})\, d_2 + p_1)\, Ap\, d_1 + d_2\, P2_{Total}\, p_2}{(Bp\, d_2 + 1)^2\, Ap\, p_1\, d_1}$$

Now since $\frac{\partial ACon}{\partial Bp}$ and $\frac{\partial KCon}{\partial Bp}$ are finite and always have non-zero denominators (as shown below)

$$simplify(diff(ACon, Bp)) = \frac{d_2\, P2_{Total}\, p_2}{(Bp\, d_2 + 1)^2\, p_1}$$

$$simplify(diff(KCon, Bp)) = 0$$

the differentiated expressions further simplify to

$$0 = \frac{\partial ACon}{\partial K} \cdot \frac{\partial K}{\partial K_{Total}}$$

$$0 = \frac{\partial Bp}{\partial K_{Total}}$$

$$1 = \frac{\partial K}{\partial K_{Total}}$$

This implies that $\frac{\partial ACon}{\partial K} = 0$ is a necessary condition for the biphasic behavior to exist. However we can also see that $\frac{\partial ACon}{\partial K} = 0$ is not possible for any feasible steady state of the system (see below)

$$simplify(diff(ACon, K)) = -\frac{c_2\, Ap\, P1_{Total}\, k_2}{(Ap\, c_2 + 1)\, K^2\, k_1\, c_1}$$

Thus a biphasic response in Ap cannot be possible with total kinase concentration (K_{Total}).

Absence of biphasic response in Bp with total kinase concentration

In a similar manner we can show that a biphasic response in Bp can also not exist with total kinase.

We proceed similarly, with a proof by contradiction, starting with the assumption that there exists a biphasic response in Bp with total kinase.

$$\frac{\partial Bp}{\partial K_{Total}} = 0$$

Thus, the above expressions obtained after differentiation can be simplified as follows

$$0 = \frac{\partial ACon}{\partial Ap} \cdot \frac{\partial Ap}{\partial K_{Total}} + \frac{\partial ACon}{\partial K} \cdot \frac{\partial K}{\partial K_{Total}}$$
$$0 = \frac{\partial BCon}{\partial Ap} \cdot \frac{\partial Ap}{\partial K_{Total}}$$
$$1 = \frac{\partial K}{\partial K_{Total}} + \frac{\partial KCon}{\partial Ap} \cdot \frac{\partial Ap}{\partial K_{Total}}$$

Note

This simplification is possible since the functions $\frac{\partial ACon}{\partial Bp}, \frac{\partial BCon}{\partial Bp}, \frac{\partial KCon}{\partial Bp}$ are finite and always have non-zero denominators (as shown below), and thus the products involving $\frac{\partial Bp}{\partial K_{Total}} = 0$ can be zero.

$$simplify(diff(ACon, Bp)) = \frac{d_2 P2_{Total} p_2}{(Bp\, d_2 + 1)^2 p_1}$$
$$simplify(diff(BCon, Bp)) = \frac{\left(Bp^2 d_2^2 p_1 + \left((2\, Bp + P2_{Total})\, p_1 + p_2 P2_{Total}\right) d_2 + p_1\right) Ap\, d_1 + d_2 P2_{Total} p_2}{(Bp\, d_2 + 1)^2 Ap\, p_1 d_1}$$
$$simplify(diff(KCon, Bp)) = 0$$

Now we can make the following inference that since $\frac{\partial BCon}{\partial Ap}$ is never zero (as shown below), that $\frac{\partial Ap}{\partial K_{Total}}$ has to be necessarily zero (at the biphasic peak).

$$simplify(diff(BCon, Ap)) = -\frac{d_2\, Bp\, P2_{Total} p_2}{(Bp\, d_2 + 1)\, Ap^2 p_1 d_1}$$

Now since $\frac{\partial ACon}{\partial Ap}$ and $\frac{\partial KCon}{\partial Ap}$ are finite and always have non-zero denominators (as shown below)

$$simplify(diff(ACon, Ap)) = \frac{\left(Ap^2 c_2^2 k_1 + \left((2\, Ap + P1_{Total})\, k_1 + k_2 P1_{Total}\right) c_2 + k_1\right) K c_1 + c_2 P1_{Total} k_2}{(Ap\, c_2 + 1)^2 K k_1 c_1}$$
$$simplify(diff(KCon, Ap)) = \frac{c_2 P1_{Total} k_2}{(c_2 Ap + 1)^2 k_1}$$

the differentiated expressions further simplify to

$$0 = \frac{\partial ACon}{\partial K} \cdot \frac{\partial K}{\partial K_{Total}}$$

$$0 = \frac{\partial Ap}{\partial K_{Total}}$$

$$1 = \frac{\partial K}{\partial K_{Total}}$$

However we can also see that $\frac{\partial ACon}{\partial K} = 0$ is not possible for any feasible steady state of the system.

$$simplify(diff(ACon, K)) = -\frac{c_2\,Ap\,P1_{Total}\,k_2}{(c_2\,Ap + 1)\,K^2\,k_1\,c_1}$$

Thus a biphasic response in Bp cannot be possible with total kinase concentration (K_{Total}).

2. Absence of substrate biphasic dose response in Ap with total substrate concentration (A_{Total})

In this subsection we show the absence of substrate-biphasic in the modified form of the first tier substrate (Ap) with total substrate concentration (A_{Total}). As mentioned earlier, feasible solutions to the three coupled expressions (ACon, BCon, KCon - shown below) would define the steady state of the system.

$ACon$

$$\frac{c_2\,Ap\,P1_{Total}\,k_2}{(Ap\,c_2 + 1)\,K\,k_1\,c_1} + Ap + \frac{c_2\,Ap\,P1_{Total}}{Ap\,c_2 + 1} + \frac{c_2\,Ap\,P1_{Total}\,k_2}{(Ap\,c_2 + 1)\,k_1} + \frac{d_2\,Bp\,P2_{Total}\,p_2}{(Bp\,d_2 + 1)\,p_1} - A_{Total} \tag{2.2.1}$$

$BCon$

$$\frac{d_2\,Bp\,P2_{Total}\,p_2}{(Bp\,d_2 + 1)\,Ap\,p_1\,d_1} + \frac{d_2\,Bp\,P2_{Total}}{Bp\,d_2 + 1} + \frac{d_2\,Bp\,P2_{Total}\,p_2}{(Bp\,d_2 + 1)\,p_1} + Bp - B_{Total} \tag{2.2.2}$$

$KCon$

$$K + \frac{c_2\,Ap\,P1_{Total}\,k_2}{(Ap\,c_2 + 1)\,k_1} - K_{Total} \tag{2.2.3}$$

If we are to differentiate the expressions by total substrate amount A_{Total} we would have the following expressions

$$\frac{dACon}{dA_{Total}} = 0 = \frac{\partial ACon}{\partial A_{Total}} + \frac{\partial ACon}{\partial Ap}\cdot\frac{\partial Ap}{\partial A_{Total}} + \frac{\partial ACon}{\partial Bp}\cdot\frac{\partial Bp}{\partial A_{Total}} + \frac{\partial ACon}{\partial K}\cdot\frac{\partial K}{\partial A_{Total}}$$

$$\frac{dBCon}{dA_{Total}} = 0 = \frac{\partial BCon}{\partial Ap}\cdot\frac{\partial Ap}{\partial A_{Total}} + \frac{\partial BCon}{\partial Bp}\cdot\frac{\partial Bp}{\partial A_{Total}}$$

$$\frac{dKCon}{dA_{Total}} = 0 = \frac{\partial KCon}{\partial K}\cdot\frac{\partial K}{\partial A_{Total}} + \frac{\partial KCon}{\partial Ap}\cdot\frac{\partial Ap}{\partial A_{Total}} + \frac{\partial KCon}{\partial Bp}\cdot\frac{\partial Bp}{\partial A_{Total}}$$

We now show the absence of a biphasic response in the concentration of Ap with A_{Total} using a proof by contradiction.

Assuming that a biphasic response exists, there should exist a steady state of the system where the following should be satisfied (at the biphasic peak)

$$\frac{\partial Ap}{\partial A_{Total}} = 0$$

Thus, the above expressions obtained after differentiation can be simplified as follows

$$1 = \frac{\partial ACon}{\partial Bp} \cdot \frac{\partial Bp}{\partial A_{Total}} + \frac{\partial ACon}{\partial K} \cdot \frac{\partial K}{\partial A_{Total}}$$
$$0 = \frac{\partial BCon}{\partial Bp} \cdot \frac{\partial Bp}{\partial A_{Total}}$$
$$0 = \frac{\partial KCon}{\partial Bp} \cdot \frac{\partial Bp}{\partial A_{Total}} + \frac{\partial KCon}{\partial K} \cdot \frac{\partial K}{\partial A_{Total}}$$

Note

This simplification is possible since the functions $\frac{\partial ACon}{\partial Ap}$, $\frac{\partial BCon}{\partial Ap}$, $\frac{\partial KCon}{\partial Ap}$ are finite and always have non-zero denominators (as shown below), and thus the products involving $\frac{\partial Ap}{\partial A_{Total}} = 0$ can be zero.

$$simplify(diff(ACon, Ap)) = \frac{K\left(Ap^2 c_2^2 k_1 + \left(\left(2\,Ap + P1_{Total}\right) k_1 + k_2 P1_{Total}\right) c_2 + k_1\right) c_1 + c_2 P1_{Total} k_2}{\left(Ap\, c_2 + 1\right)^2 K k_1 c_1}$$

$$simplify(diff(BCon, Ap)) = -\frac{d_2\, Bp\, P2_{Total}\, p_2}{\left(Bp\, d_2 + 1\right) Ap^2 p_1 d_1}$$

$$simplify(diff(KCon, Ap)) = \frac{c_2 P1_{Total} k_2}{\left(Ap\, c_2 + 1\right)^2 k_1}$$

Now we can make the following inference that since $\frac{\partial BCon}{\partial Bp}$ is never zero (as shown below), that $\frac{\partial Bp}{\partial A_{Total}}$ has to be necessarily zero (at the biphasic peak).

$simplify(diff(BCon, Bp))$

$$\frac{Ap\left(Bp^2 d_2^2 p_1 + \left(\left(2\,Bp + P2_{Total}\right) p_1 + p_2 P2_{Total}\right) d_2 + p_1\right) d_1 + d_2 P2_{Total} p_2}{\left(Bp\, d_2 + 1\right)^2 Ap\, p_1 d_1} \qquad \textbf{(2.2.4)}$$

Now since $\frac{\partial ACon}{\partial Bp}$ and $\frac{\partial KCon}{\partial Bp}$ are finite and always have non-zero denominators (as shown below)

$$simplify(diff(ACon, Bp)) = \frac{d_2 P2_{Total} p_2}{\left(Bp\, d_2 + 1\right)^2 p_1}$$
$$simplify(diff(KCon, Bp)) = 0$$

the differentiated expressions further simplify to

$$1 = \frac{\partial ACon}{\partial K} \cdot \frac{\partial K}{\partial A_{Total}}$$

$$0 = \frac{\partial Bp}{\partial A_{Total}}$$

$$0 = \frac{\partial K}{\partial A_{Total}}$$

However we now notice a contradiction, $0 = \frac{\partial K}{\partial A_{Total}}$ has to be true, while $1 = \frac{\partial ACon}{\partial K} \cdot \frac{\partial K}{\partial A_{Total}}$.

This is not possible since $\frac{\partial ACon}{\partial K}$ is finite and has a non-zero denominator (see note below) and thus the product of $\frac{\partial ACon}{\partial K}$ and $\frac{\partial K}{\partial A_{Total}}$ cannot be 1, while the latter is necessarily zero.

Note

This simplification is possible since the functions $\frac{\partial ACon}{\partial K}$ is finite and has a non-zero denominator (as shown below).

$$simplify(diff(ACon, K)) = -\frac{c_2\, Ap\, P1_{Total}\, k_2}{(Ap\, c_2 + 1)\, K^2\, k_1\, c_1}$$

Thus a biphasic response in Ap is not possible with total substrate concentration A_{Total}.

3. Absence of substrate biphasic dose response in Bp with total substrate concentration (B_{Total})

In this subsection we show the absence of substrate-biphasic in the modified form of the second tier substrate (Bp) with total substrate concentration (B_{Total}). As mentioned earlier, feasible solutions to the three coupled expressions (ACon, BCon, KCon - shown below) would define the steady state of the system.

$ACon$

$$\frac{c_2\, Ap\, P1_{Total}\, k_2}{(Ap\, c_2 + 1)\, K k_1\, c_1} + Ap + \frac{c_2\, Ap\, P1_{Total}}{Ap\, c_2 + 1} + \frac{c_2\, Ap\, P1_{Total}\, k_2}{(Ap\, c_2 + 1)\, k_1} + \frac{d_2\, Bp\, P2_{Total}\, p_2}{(Bp\, d_2 + 1)\, p_1} - A_{Total} \quad \textbf{(2.3.1)}$$

$BCon$

$$\frac{d_2\, Bp\, P2_{Total}\, p_2}{(Bp\, d_2 + 1)\, Ap\, p_1\, d_1} + \frac{d_2\, Bp\, P2_{Total}}{Bp\, d_2 + 1} + \frac{d_2\, Bp\, P2_{Total}\, p_2}{(Bp\, d_2 + 1)\, p_1} + Bp - B_{Total} \quad \textbf{(2.3.2)}$$

$KCon$

$$K + \frac{c_2\, Ap\, P1_{Total}\, k_2}{(Ap\, c_2 + 1)\, k_1} - K_{Total} \quad \textbf{(2.3.3)}$$

If we are to differentiate the expressions by total substrate amount B_{Total} we would have the following expressions

$$\frac{dACon}{dB_{Total}} = 0 = \frac{\partial ACon}{\partial Ap} \cdot \frac{\partial Ap}{\partial B_{Total}} + \frac{\partial ACon}{\partial Bp} \cdot \frac{\partial Bp}{\partial B_{Total}} + \frac{\partial ACon}{\partial K} \cdot \frac{\partial K}{\partial B_{Total}}$$
$$\frac{dBCon}{dB_{Total}} = 0 = \frac{\partial BCon}{\partial B_{Total}} + \frac{\partial BCon}{\partial Ap} \cdot \frac{\partial Ap}{\partial B_{Total}} + \frac{\partial BCon}{\partial Bp} \cdot \frac{\partial Bp}{\partial B_{Total}}$$
$$\frac{dKCon}{dB_{Total}} = 0 = \frac{\partial KCon}{\partial K} \cdot \frac{\partial K}{\partial B_{Total}} + \frac{\partial KCon}{\partial Ap} \cdot \frac{\partial Ap}{\partial B_{Total}}$$

We now show the absence of a biphasic response in the concentration of Bp with B_{Total} using a proof by contradiction.

Assuming that a biphasic response exists, there should exist a steady state of the system where the following should be satisfied (at the biphasic peak)

$$\frac{\partial Bp}{\partial B_{Total}} = 0$$

Thus, the above expressions obtained after differentiation can be simplified as follows

$$0 = \frac{\partial ACon}{\partial Ap} \cdot \frac{\partial Ap}{\partial B_{Total}} + \frac{\partial ACon}{\partial K} \cdot \frac{\partial K}{\partial B_{Total}}$$
$$1 = \frac{\partial BCon}{\partial Ap} \cdot \frac{\partial Ap}{\partial B_{Total}}$$
$$0 = \frac{\partial KCon}{\partial Ap} \cdot \frac{\partial Ap}{\partial B_{Total}} + 1 \cdot \frac{\partial K}{\partial B_{Total}}$$

Note

This simplification is possible since the functions $\frac{\partial ACon}{\partial Bp}, \frac{\partial BCon}{\partial Bp}, \frac{\partial KCon}{\partial Bp}$ are finite and always have non-zero denominators (as shown below), and thus the products involving $\frac{\partial Ap}{\partial B_{Total}} = 0$ can be zero.

$$simplify(diff(ACon, Bp)) = \frac{\left(Ap^2 c_2^2 k_1 + \left(\left(2\,Ap + P1_{Total}\right) k_1 + k_2\,P1_{Total}\right) c_2 + k_1\right) K c_1 + c_2\,P1_{Total}\,k_2}{\left(Ap\,c_2 + 1\right)^2 K k_1 c_1}$$
$$simplify(diff(BCon, Bp)) = -\frac{d_2\,Bp\,P2_{Total}\,p_2}{\left(Bp\,d_2 + 1\right) Ap^2 p_1 d_1}$$
$$simplify(diff(KCon, Bp)) = \frac{c_2\,P1_{Total}\,k_2}{\left(Ap\,c_2 + 1\right)^2 k_1}$$

Now by solving equation 3 above (obtained from differentiating KCon) for $\frac{\partial K}{\partial B_{Total}}$ and substituting it in the first equation (obtained from differentiating ACon) we get,

$$0 = \frac{\partial Ap}{\partial B_{Total}} \cdot \left(\frac{\partial ACon}{\partial Ap} - \frac{\partial ACon}{\partial K} \cdot \frac{\partial KCon}{\partial Ap}\right)$$

$$1 = \frac{\partial BCon}{\partial Ap} \cdot \frac{\partial Ap}{\partial B_{Total}}$$

Now from the first expression above we can infer that $\left(\frac{\partial ACon}{\partial Ap} - \frac{\partial ACon}{\partial K} \cdot \frac{\partial KCon}{\partial Ap} \right)$ should be equal to zero (since otherwise, $\frac{\partial Ap}{\partial B_{Total}}$ must be equal to zero and this would violate the second expression, as $\frac{\partial BCon}{\partial Ap}$ is finite and has a non-zero denominator - see below).

$$simplify(diff(BCon, Ap)) = -\frac{d_2 \, Bp \, P2_{Total} \, p_2}{(Bp \, d_2 + 1) \, Ap^2 \, p_1 \, d_1}$$

However, we can see below that the expression $\left(\frac{\partial ACon}{\partial Ap} - \frac{\partial ACon}{\partial K} \cdot \frac{\partial KCon}{\partial Ap} \right)$ is never zero (see below).

$$simplify(diff(ACon, Ap) - diff(ACon, K) \cdot diff(KCon, Ap))$$

$$\frac{1}{(c_2 \, Ap + 1)^3 \, K^2 \, k_1^2 \, c_1} \Bigg(Ap^3 \, K^2 \, c_1 \, c_2^3 \, k_1^2 + 3 \, Ap \bigg(K^2 \, c_1 \left(Ap + \frac{P1_{Total}}{3} \right) k_1^2 + \frac{K \, k_2 \, P1_{Total} \, (K \, c_1 + 1) \, k_1}{3} + \frac{k_2^2 \, P1_{Total}^2}{3} \bigg) c_2^2 + 3 \, K \, k_1 \bigg(K \, c_1 \left(Ap + \frac{P1_{Total}}{3} \right) k_1 + \frac{k_2 \, P1_{Total} \, (K \, c_1 + 1)}{3} \bigg) c_2 + K^2 \, k_1^2 \, c_1 \Bigg) \quad \textbf{(2.3.4)}$$

Thus contradiction. The conditions can't be satisfied implying that a biphasic response in Ap is not possible with total substrate concentration B_{Total}.

Coupled covalent modification systems
[coupled through enzyme sharing]

In this file, we study the propensity for substrate and enzyme biphasic dose responses in modified substrate form in coupled covalent modification systems. We consider (as mentioned in the main text), a suite of models of two coupled covalent modification systems (A=Ap and B=Bp) where the coupling is achieved through enzyme sharing (one or more enzymes). By systematically studying models with different degrees of coupling (common kinase and phosphatase, common kinase only, common phosphatase only) we show the following key results

1. Common enzymes (kinase and phosphatase) model:
   a. Enzyme biphasic response is impossible in the modified substrate forms of either modification cycles (Ap or Bp) with changing total kinase concentration (K_{Total})

Note: Substrate biphasic response however in the modified form of either substrate is possible along total substrate amount and is shown in figure 9 in the main text.

2. Separate kinase - common phosphatase model:
   a. Substrate biphasic response is impossible in the modified substrate forms (Ap or Bp) with changing total substrate concentrations (total substrate concentrations of A or B respectively)
   b. Enzyme biphasic response is impossible in the modified substrate forms with either total kinase concentrations.

3. Common kinase - separate phosphatase model:
   a. Substrate biphasic response is impossible in the modified substrate forms (Ap or Bp) with changing total substrate concentrations (total substrate concentration of A or B respectively)
   b. Enzyme biphasic response is impossible in the modified form of either substrate with total kinase concentrations.

These results are summarized in the following tabular column.

Table 1: Substrate and Enzyme biphasic dose responses in the coupled covalent modification system

System	Substrate Biphasic	Enzyme Biphasic
Common Kinase Common Phosphatase	Yes (see figure N-1 in main text)	Not possible
Separate Kinase Common Phosphatase	Not possible	Not possible
Common Kinase Separate Phosphatase	Not possible	Not possible

Common enzyme model: Presence of substrate biphasic and absence of enzyme biphasic response

We initialize the *Maple* file with the *restart* command and load the relevant libraries of inbuilt *Maple* functions (*LinearAlgebra, VectorCalculus, Student[LinearAlgebra]*)

$restart : with(LinearAlgebra) : with(VectorCalculus) : with(Student[LinearAlgebra]) :$

The system is modeled as a set of ODEs using the kinetic nomenclature described in the main text. Here dA represents d[A]/dt and similarly in the case of the other variables. At steady state thus, the right hand sides of each of these expressions will be equal to zero.

$$dA := k_2 \cdot ApP - k_{b1} \cdot A \cdot K + k_{ub1} \cdot AK :$$
$$dAp := k_1 \cdot AK + k_{ub2} \cdot ApP - k_{b2} \cdot Ap \cdot P :$$
$$dAK := k_{b1} \cdot A \cdot K - (k_{ub1} + k_1) \cdot AK :$$
$$dApP := k_{b2} \cdot Ap \cdot P - (k_{ub2} + k_2) \cdot ApP :$$

$$dB := p_2 \cdot BpP - p_{b1} \cdot B \cdot K + p_{ub1} \cdot BK :$$
$$dBp := p_1 \cdot BK + p_{ub2} \cdot BpP - p_{b2} \cdot Bp \cdot P :$$
$$dBK := p_{b1} \cdot B \cdot K - (p_{ub1} + p_1) \cdot BK :$$
$$dBpP := p_{b2} \cdot Bp \cdot P - (p_{ub2} + p_2) \cdot BpP :$$

$$dK := -k_{b1} \cdot A \cdot K + (k_{ub1} + k_1) \cdot AK - p_{b1} \cdot B \cdot K + (p_{ub1} + p_1) \cdot BK :$$
$$dP := -k_{b2} \cdot Ap \cdot P + (k_{ub2} + k_2) \cdot ApP - p_{b2} \cdot Bp \cdot P + (p_{ub2} + p_2) \cdot BpP :$$

The model is also associated with conservation conditions which are described below. Here we store the conservation expressions as ACon, BCon, PCon and KCon for the substrate and the respective enzymes. Each of these expressions is always equal to zero (both in the transient and at steady state).

$$ACon := A + Ap + ApP + AK - A_{Total} :$$
$$BCon := B + Bp + BpP + BK - B_{Total} :$$
$$KCon := K + AK + BK - K_{Total} :$$
$$PCon := P + ApP + BpP - P_{Total} :$$

Now we begin by solving the system of equations to obtain expressions linking the steady state concentrations of the variables, primarily to obtain expressions for the steady state concentrations of variables as a function of concentrations of Ap and Bp. For this purpose we use an inbuilt *Maple* command *solve* as shown with the example below.

$$AK := solve(dAK, AK) = \frac{k_{b1} A K}{k_{ub1} + k_1}$$

We similarly solve for the other variables using the same command.

$assign(solve(\{dBK, dApP, dBpP\}, \{BK, ApP, BpP\}))$

$assign(solve(\{dA, dB\}, \{A, B\}))$

We introduce the following parameters (c_1, c_2, d_1, and d_2). This is done for the sake of brevity and easy tractability of the expressions obtained.

$$k_{b1} := c_1 \cdot (k_1 + k_{ub1}) : k_{b2} := c_2 \cdot (k_2 + k_{ub2}) :$$
$$p_{b1} := d_1 \cdot (p_1 + p_{ub1}) : p_{b2} := d_2 \cdot (p_2 + p_{ub2}) :$$

Once this is done, we again solve for the steady state of the phosphatase, using the conservation expression for the enzyme (PCon).

$$P := solve(PCon, P) = \frac{P_{Total}}{Ap\, c_2 + Bp\, d_2 + 1}$$

This results in the following expressions for the steady state concentrations of the various species in terms of the steady state substrate concentration Ap and Bp

$$A = \frac{c_2\, Ap\, P_{Total}\, k_2}{(Ap\, c_2 + Bp\, d_2 + 1)\, Kk_1\, c_1}$$
$$B = \frac{d_2\, Bp\, P_{Total}\, p_2}{(Ap\, c_2 + Bp\, d_2 + 1)\, Kp_1\, d_1}$$
$$AK = \frac{c_2\, Ap\, P_{Total}\, k_2}{(Ap\, c_2 + Bp\, d_2 + 1)\, k_1}$$
$$BK = \frac{d_2\, Bp\, P_{Total}\, p_2}{(Ap\, c_2 + Bp\, d_2 + 1)\, p_1}$$
$$ApP = \frac{c_2\, Ap\, P_{Total}}{Ap\, c_2 + Bp\, d_2 + 1}$$
$$BpP = \frac{d_2\, Bp\, P_{Total}}{Ap\, c_2 + Bp\, d_2 + 1}$$

Note that when Ap and Bp are positive, steady state concentrations of the other variable concentrations are positive as well. Thus we have solved the system of equations at steady state to arrive at expressions linking the steady state concentrations of the variables with that of Ap and Bp. We now have three expressions, ACon, BCon and KCon - the conservation of the substrates and kinase, whose solution for the variables define the steady state of the system.

Enzyme biphasic

In this subsection we show the absence of enzyme biphasic in either of the modified forms of the substrates with total kinase amounts. As mentioned earlier, feasible solutions to the three coupled expressions (ACon, BCon, KCon - shown below) would define the steady state of the system.

Note: Since the kinase and the phosphatase are shared by the two covalent modification cycles, the individual covalent cycles (A=Ap, B=Bp) are equivalent. Thus, a proof presenting the absence of enzyme biphasic in one covalent modification cycle is tantamount to proving the absence in both modification cycle.

$ACon$

$$\frac{c_2\, Ap\, P_{Total}\, k_2}{(Ap\, c_2 + Bp\, d_2 + 1)\, K\, k_1\, c_1} + Ap + \frac{c_2\, Ap\, P_{Total}}{Ap\, c_2 + Bp\, d_2 + 1} + \frac{c_2\, Ap\, P_{Total}\, k_2}{(Ap\, c_2 + Bp\, d_2 + 1)\, k_1} - A_{Total} \tag{1.1.1}$$

BCon

$$\frac{d_2\, Bp\, P_{Total}\, p_2}{(Ap\, c_2 + Bp\, d_2 + 1)\, K\, p_1\, d_1} + Bp + \frac{d_2\, Bp\, P_{Total}}{Ap\, c_2 + Bp\, d_2 + 1} + \frac{d_2\, Bp\, P_{Total}\, p_2}{(Ap\, c_2 + Bp\, d_2 + 1)\, p_1} - B_{Total} \tag{1.1.2}$$

KCon

$$K + \frac{c_2\, Ap\, P_{Total}\, k_2}{(Ap\, c_2 + Bp\, d_2 + 1)\, k_1} + \frac{d_2\, Bp\, P_{Total}\, p_2}{(Ap\, c_2 + Bp\, d_2 + 1)\, p_1} - K_{Total} \tag{1.1.3}$$

If we are to differentiate the expressions by total kinase amounts we would have the following expressions

$$\frac{dACon}{dK_{Total}} = 0 = \frac{\partial ACon}{\partial Ap} \cdot \frac{\partial Ap}{\partial K_{Total}} + \frac{\partial ACon}{\partial Bp} \cdot \frac{\partial Bp}{\partial K_{Total}} + \frac{\partial ACon}{\partial K} \cdot \frac{\partial K}{\partial K_{Total}}$$

$$\frac{dBCon}{dK_{Total}} = 0 = \frac{\partial BCon}{\partial Ap} \cdot \frac{\partial Ap}{\partial K_{Total}} + \frac{\partial BCon}{\partial Bp} \cdot \frac{\partial Bp}{\partial K_{Total}} + \frac{\partial BCon}{\partial K} \cdot \frac{\partial K}{\partial K_{Total}}$$

$$\frac{dKCon}{dK_{Total}} = 0 = \frac{\partial KCon}{\partial K_{Total}} + \frac{\partial KCon}{\partial K} \cdot \frac{\partial K}{\partial K_{Total}} + \frac{\partial KCon}{\partial Ap} \cdot \frac{\partial Ap}{\partial K_{Total}} + \frac{\partial KCon}{\partial Bp} \cdot \frac{\partial Bp}{\partial K_{Total}}$$

Now in order to show the absence of biphasic response in Bp with total kinase concentration, we proceed with a proof by contradiction.

If we assume that such a biphasic response exists, then there should exist a steady state of the system where the following should be satisfied (at the biphasic peak)

$$\frac{\partial Bp}{\partial K_{Total}} = 0$$

Thus, the above expressions obtained after differentiation can be simplified as follows

$$0 = \frac{\partial ACon}{\partial Ap} \cdot \frac{\partial Ap}{\partial K_{Total}} + \frac{\partial ACon}{\partial K} \cdot \frac{\partial K}{\partial K_{Total}}$$

$$0 = \frac{\partial BCon}{\partial Ap} \cdot \frac{\partial Ap}{\partial K_{Total}} + \frac{\partial BCon}{\partial K} \cdot \frac{\partial K}{\partial K_{Total}}$$

$$1 = \frac{\partial K}{\partial K_{Total}} + \frac{\partial KCon}{\partial Ap} \cdot \frac{\partial Ap}{\partial K_{Total}}$$

The first two equations are homogenous linear equations in $\frac{\partial Ap}{\partial K_{Total}}$ and $\frac{\partial K}{\partial K_{Tota}}$. If the determinant of these equations are strictly non-zero, then the only solutions are for $\frac{\partial Ap}{\partial K_{Total}}$ and $\frac{\partial K}{\partial K_{Tota}}$ are zero. We evaluate this determinant below as shown,

simplify(*diff*(*ACon*, *Ap*)·*diff*(*BCon*, *K*) − *diff*(*ACon*, *K*)·*diff*(*BCon*, *Ap*))

$$-\frac{1}{\left(Ap\, c_2 + Bp\, d_2 + 1\right)^3 K^3 k_1 c_1 p_1 d_1} \left(\left(\left(Ap \left(\left(Ap\, c_1 k_1 + d_1 k_2 P_{Total} \right) K + P_{Total} k_2 \right) c_2^2 + \left(Bp\, d_2 + 1 \right) \left(c_1 \left(\left(k_1 + k_2 \right) P_{Total} + 2\, Ap\, k_1 \right) K + P_{Total} k_2 \right) c_2 + K c_1 k_1 \left(Bp\, d_2 + 1 \right)^2 \right) p_2 + Ap\, K c_2^2 d_1 k_2 p_1 P_{Total} \right) Bp\, d_2 P_{Total} \right) \qquad \textbf{(1.1.4)}$$

As we can see this is non-zero for any feasible concentration of Ap, K, Bp, and kinetic rate constants. Hence $\frac{\partial Ap}{\partial K_{Total}} = 0$ and $\frac{\partial K}{\partial K_{Tota}} = 0$

This leads to a contradiction as from the third expression above, $1 = \frac{\partial K}{\partial K_{Total}} + \frac{\partial KCon}{\partial Ap} \cdot \frac{\partial Ap}{\partial K_{Total}}$ for a biphasic response to exist. Since the denominator of $\frac{\partial KCon}{\partial Ap}$ is non-zero, this equation cannot be satisfied (see below).

$$simplify(diff(KCon, Ap)) = -\frac{c_2 P_{Total} \left(Bp \left(p_2 k_1 - k_2 p_1 \right) d_2 - k_2 p_1 \right)}{\left(Ap\, c_2 + Bp\, d_2 + 1 \right)^2 k_1 p_1}$$

Thus enzyme biphasic cannot exist in Bp with total kinase concentration (K_{Total}).

Substrate biphasic

In this subsection we show the presnce of substrate biphasic in the modified form of a substrate form with the respecitve total amount of substrate as dose. We will further show how this substrate biphasic is guaranteed to exist in exactly one of the covalent modification cycles irrespective of the kinetic regime of the (de)modifications. As mentioned earlier, feasible solutions to the three coupled expressions (ACon, BCon, KCon - shown below) would define the steady state of the system.

Note: Since the kinase and the phosphatase are shared by the two covalent modification cycles, the individual covalent cycles (A=Ap, B=Bp) are equivalent. Thus, a proof presenting the presence of substrate biphasic in one covalent modification cycle is tantamount to proving the presence in the other modification cycle. We thus proceed by focussing on the presence of biphasic dose response with A_{Total} in this sub-section first.

ACon

$$\frac{c_2\, Ap\, P_{Total} k_2}{\left(Ap\, c_2 + Bp\, d_2 + 1 \right) K k_1 c_1} + Ap + \frac{c_2\, Ap\, P_{Total}}{Ap\, c_2 + Bp\, d_2 + 1} + \frac{c_2\, Ap\, P_{Total} k_2}{\left(Ap\, c_2 + Bp\, d_2 + 1 \right) k_1} - A_{Total} \qquad \textbf{(1.2.1)}$$

KCon

$$K + \frac{c_2\, Ap\, P_{Total} k_2}{\left(Ap\, c_2 + Bp\, d_2 + 1 \right) k_1} + \frac{d_2\, Bp\, P_{Total} p_2}{\left(Ap\, c_2 + Bp\, d_2 + 1 \right) p_1} - K_{Total} \qquad \textbf{(1.2.2)}$$

BCon

$$\frac{d_2\, Bp\, P_{Total} p_2}{\left(Ap\, c_2 + Bp\, d_2 + 1 \right) K p_1 d_1} + Bp + \frac{d_2\, Bp\, P_{Total}}{Ap\, c_2 + Bp\, d_2 + 1} + \frac{d_2\, Bp\, P_{Total} p_2}{\left(Ap\, c_2 + Bp\, d_2 + 1 \right) p_1} - B_{Total} \qquad \textbf{(1.2.3)}$$

If we are to differentiate the expressions by total substrate amount (A_{Total}) we would have the following expressions

$$\frac{dACon}{dA_{Total}} = 0 = \frac{\partial ACon}{\partial A_{Total}} + \frac{\partial ACon}{\partial Ap} \cdot \frac{\partial Ap}{\partial A_{Total}} + \frac{\partial ACon}{\partial Bp} \cdot \frac{\partial Bp}{\partial A_{Total}} + \frac{\partial ACon}{\partial K} \cdot \frac{\partial K}{\partial A_{Total}}$$

$$\frac{dBCon}{dA_{Total}} = 0 = \frac{\partial BCon}{\partial Ap} \cdot \frac{\partial Ap}{\partial A_{Total}} + \frac{\partial BCon}{\partial Bp} \cdot \frac{\partial Bp}{\partial A_{Total}} + \frac{\partial BCon}{\partial K} \cdot \frac{\partial K}{\partial A_{Total}}$$

$$\frac{dKCon}{dA_{Total}} = 0 = \frac{\partial KCon}{\partial K} \cdot \frac{\partial K}{\partial A_{Total}} + \frac{\partial KCon}{\partial Ap} \cdot \frac{\partial Ap}{\partial A_{Total}} + \frac{\partial KCon}{\partial Bp} \cdot \frac{\partial Bp}{\partial A_{Total}}$$

Now in order to show the presence of biphasic response in Ap with total substrate concentration, we proceed by checking if the necessary conditions for the behavior to exist can be satisfied.

If we assume that such a biphasic response exists, then there should exist a steady state of the system where the following should be satisfied (at the biphasic peak)

$$\frac{\partial Ap}{\partial A_{Total}} = 0$$

Thus, the above expressions obtained after differentiation can be simplified as follows

$$1 = \frac{\partial ACon}{\partial Bp} \cdot \frac{\partial Bp}{\partial A_{Total}} + \frac{\partial ACon}{\partial K} \cdot \frac{\partial K}{\partial A_{Total}}$$

$$0 = \frac{\partial BCon}{\partial Bp} \cdot \frac{\partial Bp}{\partial A_{Total}} + \frac{\partial BCon}{\partial K} \cdot \frac{\partial K}{\partial A_{Total}}$$

$$0 = 1 \cdot \frac{\partial K}{\partial A_{Total}} + \frac{\partial KCon}{\partial Bp} \cdot \frac{\partial Bp}{\partial A_{Total}}$$

Note

This simplification is possible since the functions $\frac{\partial ACon}{\partial Ap}, \frac{\partial BCon}{\partial Ap}, \frac{\partial KCon}{\partial Ap}$ are finite and always have non-zero denominators (as shown below), and thus the products involving $\frac{\partial Ap}{\partial A_{Total}}$ can be zero.

$$simplify(diff(ACon, Ap)) = -\frac{P_{Total}\, Ap\, d_2\, c_2\, \left(K\left(k_1 + k_2\right) c_1 + k_2\right)}{\left(Ap\, c_2 + Bp\, d_2 + 1\right)^2 K\, k_1\, c_1}$$

$$simplify(diff(BCon, Ap)) = \frac{1}{\left(Ap\, c_2 + Bp\, d_2 + 1\right)^2 K\, p_1\, d_1} \left(K\left(Bp^2\, p_1\, d_2^2 + 2\left(\left(Bp + \frac{P_{Total}}{2}\right) p_1 + \frac{p_2\, P_{Total}}{2}\right)\left(Ap\, c_2 + 1\right) d_2 + p_1\left(Ap\, c_2 + 1\right)^2\right) d_1 + d_2\, p_2\, P_{Total}\left(Ap\, c_2 + 1\right)\right)$$

$$simplify(diff(KCon, Ap)) = \frac{P_{Total}\left(Ap\left(p_2\, k_1 - k_2\, p_1\right) c_2 + p_2\, k_1\right) d_2}{\left(Ap\, c_2 + Bp\, d_2 + 1\right)^2 k_1\, p_1}$$

Now solving the final expression obtained above after differentiation for

$\frac{\partial K}{\partial K_{Total}}$ and resubstituting it in the other expressions (as shown below) we get,

$$1 = \frac{\partial Bp}{\partial A_{Total}} \cdot \left(\frac{\partial ACon}{\partial Bp} - \frac{\partial ACon}{\partial K} \cdot \frac{\partial KCon}{\partial Bp} \right)$$
$$0 = \frac{\partial Bp}{\partial A_{Total}} \cdot \left(\frac{\partial BCon}{\partial Bp} - \frac{\partial BCon}{\partial K} \cdot \frac{\partial KCon}{\partial Bp} \right)$$
$$\frac{\partial K}{\partial A_{Total}} = -\frac{\partial KCon}{\partial Bp} \cdot \frac{\partial Bp}{\partial A_{Total}}$$

From the first expression, it is easy to discern that $\frac{\partial Bp}{\partial A_{Total}}$ cannot be equal to zero. Thus this implies that the the expression contained in the brackets in the second equation has to equal zero for a biphasic dose response to exist. We thus isolate this expression as shown below,

$simplify(diff(BCon, Bp) - diff(BCon, K) \cdot diff(KCon, Bp))$

$$\frac{1}{(Ap\, c_2 + Bp\, d_2 + 1)^3 K^2 p_1^2 d_1 k_1} \Big(\big((Bp^2 d_2^2 + (Ap\, c_2 + 1)(P_{Total} + 2\, Bp)\, d_2 + (Ap\, c_2 + 1)^2)(Ap\, c_2 + Bp\, d_2 + 1) K^2 d_1 p_1^2 + K p_2 P_{Total} d_2 (Ap\, c_2 + 1)(Ap\, c_2 + Bp\, d_2 + 1)(K d_1 + 1) p_1 + p_2^2 P_{Total}^2\, Bp\, d_2^2 (Ap\, c_2 + 1) \big) k_1 - Ap\, Bp\, c_2 d_2^2 k_2 p_1 p_2 P_{Total}^2 \Big) \tag{1.2.4}$$

Collecting only the numerator, and writing it as a polynomial in P_{Total}, we get

$collect\Big(\big((Bp^2 d_2^2 + (Ap\, c_2 + 1)(P_{Total} + 2\, Bp)\, d_2 + (Ap\, c_2 + 1)^2)(Ap\, c_2 + Bp\, d_2 + 1) K^2 d_1 p_1^2 + K p_2 P_{Total} d_2 (Ap\, c_2 + 1)(Ap\, c_2 + Bp\, d_2 + 1)(K d_1 + 1) p_1 + p_2^2 P_{Total}^2\, Bp\, d_2^2 (Ap\, c_2 + 1) \big) k_1 - Ap\, Bp\, c_2 d_2^2 k_2 p_1 p_2 P_{Total}^2, P_{Total} \Big)$

$$\left(p_2^2\, Bp\, d_2^2 (Ap\, c_2 + 1) k_1 - Ap\, Bp\, c_2 d_2^2 k_2 p_1 p_2\right) P_{Total}^2 + \big((Ap\, c_2 + 1) d_2 (Ap\, c_2 + Bp\, d_2 + 1) K^2 d_1 p_1^2 + K p_2 d_2 (Ap\, c_2 + 1)(Ap\, c_2 + Bp\, d_2 + 1)(K d_1 + 1) p_1 \big) k_1 P_{Total} + \left(Bp^2 d_2^2 + 2\,(Ap\, c_2 + 1)\, Bp\, d_2 + (Ap\, c_2 + 1)^2\right)(Ap\, c_2 + Bp\, d_2 + 1) K^2 d_1 p_1^2 k_1 \tag{1.2.5}$$

We can thus observe that for a feasible steady state of the system, only the coeffecient of the second (leading) exponent of PTotal, can be negative. The other coeffecients are strictly positive. We now isolate this coeffecient below,

$collect\left(simplify\left(Ap\, c_2^2 k_2^2 (Bp\, d_2 + 1) p_1 - Ap\, Bp\, c_2^2 d_2 k_1 k_2 p_2\right), \{Bp, Ap, d_2\}\right)$

$$\left(-(k_1 p_2 - p_1 k_2) k_2 c_2^2 d_2\, Bp + c_2^2 k_2^2 p_1\right) Ap \tag{1.2.6}$$

This implies that, depending on the sign of $k_1 p_2 - k_2 p_1$, the coeffecient can be negative or strictly positive (in which case the equation, 1.5, can never be zero for a feasible steady state ruling out substrate biphasic dose response). If

$k_1 p_2 - p_1 k_2 > 0$, then for a sufficiently large Bp concentration, the coeffecient is negative (irrespective of the value of Ap), guaranteeing that there exists a solution for equation 1.5 (featuring any value of Ap and K at some P_{Total}).

Thus for some finite amount of K_{Total} and B_{Total}, it is possible to obtain substrate biphasic response in Ap for changing A_{Total}, if $k_1 p_2 - p_1 k_2 > 0$.

However if $k_1 p_2 - p_1 k_2 < 0$, substrate biphasic in Ap with ATotal is strictly not possible.

Here by leveraging the fact that the two covalent cycles are essentially equivalent (A=Ap with the kinetic nomenclature k_i, and B=Bp with the kinetic nomenclature p_i), we can see that if $k_1 p_2 - p_1 k_2 < 0$, then the condition for substrate biphasic in Bp with B_{Total} is trivially satisfied again indicating that there exists some total finite amount of K_{Total} and A_{Total}, where substrate biphasic in Bp with B_{Total} is guaranteed to exist.

Thus to conclude - this proves that irrespective of the kinetic regime, either Ap or Bp is exclusively guaranteed to (for some finite amount of substrate and enzyme) present with biphasic dose repsonse with the respective substrates. Specifically, if $k_1 p_2 - p_1 k_2 > 0$ then Ap is capable of substrate biphasic while Bp is not, and when $k_1 p_2 - p_1 k_2 < 0$ then Bp is capable of substrate biphasic while Ap is not.

Separate kinase common phosphatase model: Absence of substrate and enzyme biphasic dose response

We initialize the *Maple* file with the *restart* command and load the relevant libraries of inbuilt *Maple* functions (*LinearAlgebra, VectorCalculus, Student[LinearAlgebra]*)

restart : *with*(*LinearAlgebra*) : *with*(*VectorCalculus*) : *with*(*Student*[*LinearAlgebra*]) :

The system is modeled as a set of ODEs using the kinetic nomenclature described in the main text. Here dA represents d[A]/dt and similarly in the case of the other variables. At steady state thus, the right hand sides of each of these expressions will be equal to zero.

$dA := k_2 \cdot ApP - k_{b1} \cdot A \cdot K1 + k_{ub1} \cdot AK1$:
$dAp := k_1 \cdot AK1 + k_{ub2} \cdot ApP - k_{b2} \cdot Ap \cdot P$:

$dAK1 := k_{b1} \cdot A \cdot K1 - (k_{ub1} + k_1) \cdot AK1$:
$dApP := k_{b2} \cdot Ap \cdot P - (k_{ub2} + k_2) \cdot ApP$:

$dB := p_2 \cdot BpP - p_{b1} \cdot B \cdot K2 + p_{ub1} \cdot BK2$:
$dBp := p_1 \cdot BK2 + p_{ub2} \cdot BpP - p_{b2} \cdot Bp \cdot P$:

$dBK2 := p_{b1} \cdot B \cdot K2 - (p_{ub1} + p_1) \cdot BK2$:
$dBpP := p_{b2} \cdot Bp \cdot P - (p_{ub2} + p_2) \cdot BpP$:

$dK1 := -k_{b1} \cdot A \cdot K1 + (k_{ub1} + k_1) \cdot AK1$:
$dK2 := -p_{b1} \cdot B \cdot K2 + (p_{ub1} + p_1) \cdot BK2$:

$dP := -p_{b1} \cdot B \cdot K2 + \left(p_{ub1} + p_1\right) \cdot BK2 - p_{b2} \cdot Bp \cdot P + \left(p_{ub2} + p_2\right) \cdot BpP :$

The model is also associated with conservation conditions which are described below. Here we store the conservation expressions as ACon, BCon, PCon, K1Con and K2Con for the substrates and the respective enzymes. Each of these expressions is always equal to zero (both in the transient and at steady state).

$ACon := A + Ap + ApP + AK1 - A_{Total} :$
$BCon := B + Bp + BpP + BK2 - B_{Total} :$
$K1Con := K1 + AK1 - K1_{Total} :$
$K2Con := K2 + BK2 - K2_{Total} :$
$PCon := P + ApP + BpP - P_{Total} :$

We introduce the following parameters (c_1, c_2, d_1, and d_2). This is done for the sake of brevity and easy tractability of the expressions obtained.

$k_{b1} := c_1 \cdot \left(k_1 + k_{ub1}\right) : k_{b2} := c_2 \cdot \left(k_2 + k_{ub2}\right) :$
$p_{b1} := d_1 \cdot \left(p_1 + p_{ub1}\right) : p_{b2} := d_2 \cdot \left(p_2 + p_{ub2}\right) :$

Once this is done, we again solve for the steady state of the phosphatase, using the conservation expression for the enzyme (PCon).

$AK1 := solve(dAK1, AK1) = K1\ A\ c_1$

We similarly solve for the other variables using the same command.

$assign(solve(\{dBK2, dApP, dBpP\}, \{BK2, ApP, BpP\}))$
$assign(solve(\{dA, dB\}, \{A, B\}))$

Once this is done, we again solve for the steady state of the phosphatase, using the conservation expression for the enzyme (PCon).

$$P := simplify(solve((PCon), P)) = \frac{P_{Total}}{Ap\ c_2 + Bp\ d_2 + 1}$$

This results in the following expressions for the steady state concentrations of the various species in terms of the steady state substrate concentration Ap and Bp

$$A = \frac{c_2\ Ap\ P_{Total}\ k_2}{\left(Ap\ c_2 + Bp\ d_2 + 1\right) K1\ c_1\ k_1}$$

$$B = \frac{d_2\ Bp\ P_{Total}\ p_2}{\left(Ap\ c_2 + Bp\ d_2 + 1\right) K2\ d_1\ p_1}$$

$$AK1 = \frac{c_2\ Ap\ P_{Total}\ k_2}{\left(Ap\ c_2 + Bp\ d_2 + 1\right) k_1}$$

$$BK2 = \frac{d_2\ Bp\ P_{Total}\ p_2}{\left(Ap\ c_2 + Bp\ d_2 + 1\right) p_1}$$

$ApP =$

$$\frac{c_2\, Ap\, P_{Total}}{Ap\, c_2 + Bp\, d_2 + 1}$$

$$BpP = \frac{d_2\, Bp\, P_{Total}}{Ap\, c_2 + Bp\, d_2 + 1}$$

Substrate biphasic

In this subsection we show the absence of substrate biphasic in either of the modified forms of the substrates with total substrate amounts. As mentioned earlier, feasible solutions to the three coupled expressions (ACon, BCon, K1Con, K2Con - shown below) would define the steady state of the system.

Note: Since the two covalent modification cycles are virtually identical to one and other with a shared phosphatase and two unique kinases acting on them, a proof presenting the absence of substrate biphasic in one covalent modification cycle is tantamount to proving the absence in both modification cycle.

$ACon$

$$\frac{c_2\, Ap\, P_{Total}\, k_2}{\left(Ap\, c_2 + Bp\, d_2 + 1\right) K1\, c_1\, k_1} + Ap + \frac{c_2\, Ap\, P_{Total}}{Ap\, c_2 + Bp\, d_2 + 1} + \frac{c_2\, Ap\, P_{Total}\, k_2}{\left(Ap\, c_2 + Bp\, d_2 + 1\right) k_1} - A_{Total} \tag{2.1.1}$$

$BCon$

$$\frac{d_2\, Bp\, P_{Total}\, p_2}{\left(Ap\, c_2 + Bp\, d_2 + 1\right) K2\, d_1\, p_1} + Bp + \frac{d_2\, Bp\, P_{Total}}{Ap\, c_2 + Bp\, d_2 + 1} + \frac{d_2\, Bp\, P_{Total}\, p_2}{\left(Ap\, c_2 + Bp\, d_2 + 1\right) p_1} - B_{Total} \tag{2.1.2}$$

$K1Con$

$$K1 + \frac{c_2\, Ap\, P_{Total}\, k_2}{\left(Ap\, c_2 + Bp\, d_2 + 1\right) k_1} - K1_{Total} \tag{2.1.3}$$

$K2Con$

$$K2 + \frac{d_2\, Bp\, P_{Total}\, p_2}{\left(Ap\, c_2 + Bp\, d_2 + 1\right) p_1} - K2_{Total} \tag{2.1.4}$$

Now differentiating each of these expressions with respect to B_{Total}, we get the following

$$\frac{dACon}{dB_{Total}} = 0 = \frac{\partial ACon}{\partial Ap} \cdot \frac{\partial Ap}{\partial B_{Total}} + \frac{\partial ACon}{\partial Bp} \cdot \frac{\partial Bp}{\partial B_{Total}} + \frac{\partial ACon}{\partial K1} \cdot \frac{\partial K1}{\partial B_{Total}}$$

$$\frac{dBCon}{dB_{Total}} = 0 = \frac{\partial BCon}{\partial B_{Total}} + \frac{\partial BCon}{\partial Ap} \cdot \frac{\partial Ap}{\partial B_{Total}} + \frac{\partial BCon}{\partial Bp} \cdot \frac{\partial Bp}{\partial B_{Total}} + \frac{\partial BCon}{\partial K2} \cdot \frac{\partial K2}{\partial B_{Total}}$$

$$\frac{dK1Con}{dB_{Total}} = 0 = \frac{\partial K1Con}{\partial K1} \cdot \frac{\partial K1}{\partial B_{Total}} + \frac{\partial K1Con}{\partial Ap} \cdot \frac{\partial Ap}{\partial B_{Total}} + \frac{\partial K1Con}{\partial Bp} \cdot \frac{\partial Bp}{\partial B_{Total}}$$

$$\frac{dK2Con}{dB_{Total}} = 0 = \frac{\partial K2Con}{\partial K2} \cdot \frac{\partial K2}{\partial B_{Total}} + \frac{\partial K2Con}{\partial Ap} \cdot \frac{\partial Ap}{\partial B_{Total}} + \frac{\partial K2Con}{\partial Bp} \cdot \frac{\partial Bp}{\partial B_{Total}}$$

Now in order to show the absence of biphasic response in Bp with total substrate concentration (B_{Total}), we proceed with a proof by contradiction. Note that the same procedure of which will be

valid to show the absence of substrate biphasic response in modified form Ap with A_{Total}.

If we assume that such a biphasic response exists, then there should exist a steady state of the system where the following should be satisfied (at the biphasic peak)

$$\frac{\partial Bp}{\partial B_{Total}} = 0$$

Thus, the above expressions obtained after differentiation can be simplified as follows

$$0 = \frac{\partial ACon}{\partial Ap} \cdot \frac{\partial Ap}{\partial B_{Total}} + \frac{\partial ACon}{\partial K1} \cdot \frac{\partial K1}{\partial B_{Total}}$$
$$1 = \frac{\partial BCon}{\partial Ap} \cdot \frac{\partial Ap}{\partial B_{Total}} + \frac{\partial BCon}{\partial K2} \cdot \frac{\partial K2}{\partial B_{Total}}$$
$$0 = \frac{\partial K1Con}{\partial K1} \cdot \frac{\partial K1}{\partial B_{Total}} + \frac{\partial K1Con}{\partial Ap} \cdot \frac{\partial Ap}{\partial B_{Total}}$$
$$0 = \frac{\partial K2Con}{\partial K2} \cdot \frac{\partial K2}{\partial B_{Total}} + \frac{\partial K2Con}{\partial Ap} \cdot \frac{\partial Ap}{\partial B_{Total}}$$

Note

This simplification is possible since the functions $\frac{\partial ACon}{\partial Bp}, \frac{\partial BCon}{\partial Bp}, \frac{\partial K1Con}{\partial Bp}, \frac{\partial K2Con}{\partial Bp}$ are fintie and always have non-zero denominators (as shown below), and thus the products involving $\frac{\partial Bp}{\partial B_{Total}}$ can be zero.

$$simplify(diff(ACon, Bp)) = -\frac{P_{Total}\, d_2\, c_2\, (K1\,(k_1 + k_2)\, c_1 + k_2)\, Ap}{(Ap\, c_2 + Bp\, d_2 + 1)^2\, K1\, c_1\, k_1}$$

$$simplify(diff(BCon, Bp)) = \frac{1}{(Ap\, c_2 + Bp\, d_2 + 1)^2\, K2\, d_1\, p_1} \left(K2 \left(Bp^2\, p_1\, d_2^2 + 2\,(Ap\, c_2 + 1)\left(\left(Bp + \frac{P_{Total}}{2}\right) p_1 + \frac{p_2\, P_{Total}}{2}\right) d_2 + p_1\,(Ap\, c_2 + 1)^2\right) d_1 + d_2\, p_2\, P_{Total}\,(Ap\, c_2 + 1)\right)$$

$$simplify(diff(K1Con, Bp)) = -\frac{c_2\, Ap\, P_{Total}\, k_2\, d_2}{(Ap\, c_2 + Bp\, d_2 + 1)^2\, k_1}$$

$$simplify(diff(K2Con, Bp)) = \frac{d_2\, p_2\, P_{Total}\,(Ap\, c_2 + 1)}{(Ap\, c_2 + Bp\, d_2 + 1)^2\, p_1}$$

Now solving the expression obtained by differentiating K1Con above for $\frac{\partial K1}{\partial B_{Total}}$ and resubstituting it in the first expression (as shown below) we get,

$$0 = \frac{\partial ACon}{\partial Ap} \cdot \frac{\partial Ap}{\partial B_{Total}} + \frac{\partial ACon}{\partial K1} \cdot \left(-\frac{\partial K1Con}{\partial Ap} \cdot \frac{\partial Ap}{\partial B_{Total}}\right)$$

which further simplifies to

$$0 = \frac{\partial Ap}{\partial B_{Total}} \cdot \left(\frac{\partial ACon}{\partial Ap} - \frac{\partial ACon}{\partial K1} \cdot \frac{\partial K1Con}{\partial Ap} \right)$$

This thus leads to the following conclusion, either $\frac{\partial Ap}{\partial B_{Total}}$ is zero, or $\frac{\partial ACon}{\partial Ap} - \frac{\partial ACon}{\partial K1} \cdot \frac{\partial K1Con}{\partial Ap}$ is zero. The former cannot be possible (see note below).

Note

Suppose, $\frac{\partial Ap}{\partial B_{Total}}$ is zero at such a point. Then, by analyzing the last two expressions, we get $\frac{\partial K1}{\partial B_{Total}}$ and $\frac{\partial K2}{\partial B_{Total}}$ to be zero (since $\frac{\partial K1Con}{\partial Ap}$, $\frac{\partial K2Con}{\partial Ap}$ are finite and the denominators are always non-zero, and $\frac{\partial K1Con}{\partial K1}$, $\frac{\partial K2Con}{\partial K2}$ are always non-zero).

$$simplify(diff(K1Con, Ap)) = \frac{c_2 P_{Total} k_2 (Bp\, d_2 + 1)}{(c_2 Ap + Bp\, d_2 + 1)^2 k_1}$$

$$simplify(diff(K2Con, Ap)) = -\frac{d_2 Bp\, P_{Total} p_2 c_2}{(c_2 Ap + Bp\, d_2 + 1)^2 p_1}$$

Thus, the second expression would be violated under this scenario. Hence, $\frac{\partial Ap}{\partial B_{Total}}$ is non-zero.

However the latter can also not be possible for a feasible steady state of the system (see below).

$simplify(diff(ACon, Ap) - diff(ACon, K1) \cdot diff(K1Con, Ap))$

$$\frac{1}{(Ap\, c_2 + Bp\, d_2 + 1)^3 K1^2 c_1 k_1^2} \Bigg(c_1 K1^2 \Big(Ap^2 c_2^2 + 2 \Big(Ap + \frac{P_{Total}}{2} \Big) (Bp\, d_2 + 1) c_2 + (Bp\, d_2 + 1)^2 \Big) (Ap\, c_2 + Bp\, d_2 + 1) k_1^2 + K1\, c_2 k_2 P_{Total} (K1\, c_1 + 1) (Bp\, d_2 + 1) (Ap\, c_2 + Bp\, d_2 + 1) k_1 + c_2^2 Ap\, P_{Total}^2 k_2^2 (Bp\, d_2 + 1) \Bigg) \quad \textbf{(2.1.5)}$$

Since all the parameters and variables involved in the expression are positive, the expression is non-zero always.

We have a contradiction.

Thus, our assumption of the presence of a substrate biphasic in Bp with B_{Total} is wrong.

Hence substrate biphasic cannot exist in the model.

Enzyme biphasic

In this subsection we show the absence of enzyme biphasic in either of the modified forms of the substrates with total kinase concentration (between the respective substrate enzyme pair). As

mentioned earlier, feasible solutions to the three coupled expressions (ACon, BCon, K1Con, K2Con - shown below) would define the steady state of the system.

Note: Since the two covalent modification cycles are virtually identical to one and other with a shared phosphatase and two unique kinases acting on them, a proof presenting the absence of enzyme biphasic in one covalent modification cycle is tantamount to proving the absence in both modification cycle.

$ACon$

$$\frac{c_2 Ap\, P_{Total}\, k_2}{(Ap\, c_2 + Bp\, d_2 + 1)\, K1\, c_1\, k_1} + Ap + \frac{c_2 Ap\, P_{Total}}{Ap\, c_2 + Bp\, d_2 + 1} + \frac{c_2 Ap\, P_{Total}\, k_2}{(Ap\, c_2 + Bp\, d_2 + 1)\, k_1} - A_{Total} \tag{2.2.1}$$

$BCon$

$$\frac{d_2 Bp\, P_{Total}\, p_2}{(Ap\, c_2 + Bp\, d_2 + 1)\, K2\, d_1\, p_1} + Bp + \frac{d_2 Bp\, P_{Total}}{Ap\, c_2 + Bp\, d_2 + 1} + \frac{d_2 Bp\, P_{Total}\, p_2}{(Ap\, c_2 + Bp\, d_2 + 1)\, p_1} - B_{Total} \tag{2.2.2}$$

$K1Con$

$$K1 + \frac{c_2 Ap\, P_{Total}\, k_2}{(Ap\, c_2 + Bp\, d_2 + 1)\, k_1} - K1_{Total} \tag{2.2.3}$$

$K2Con$

$$K2 + \frac{d_2 Bp\, P_{Total}\, p_2}{(Ap\, c_2 + Bp\, d_2 + 1)\, p_1} - K2_{Total} \tag{2.2.4}$$

Now differentiating each of these expressions with respect to $K1_{Total}$, we get the following

$$\frac{dACon}{dK2_{Total}} = 0 = \frac{\partial ACon}{\partial Ap} \cdot \frac{\partial Ap}{\partial K2_{Total}} + \frac{\partial ACon}{\partial Bp} \cdot \frac{\partial Bp}{\partial K2_{Total}} + \frac{\partial ACon}{\partial K1} \cdot \frac{\partial K1}{\partial K2_{Total}}$$

$$\frac{dBCon}{dK2_{Total}} = 0 = \frac{\partial BCon}{\partial Ap} \cdot \frac{\partial Ap}{\partial K2_{Total}} + \frac{\partial BCon}{\partial Bp} \cdot \frac{\partial Bp}{\partial K2_{Total}} + \frac{\partial BCon}{\partial K2} \cdot \frac{\partial K2}{\partial K2_{Total}}$$

$$\frac{dK1Con}{dK2_{Total}} = 0 = \frac{\partial K1Con}{\partial K1} \cdot \frac{\partial K1}{\partial K2_{Total}} + \frac{\partial K1Con}{\partial Ap} \cdot \frac{\partial Ap}{\partial K2_{Total}} + \frac{\partial K1Con}{\partial Bp} \cdot \frac{\partial Bp}{\partial K2_{Total}}$$

$$\frac{dK2Con}{dK2_{Total}} = 0 = \frac{\partial K2Con}{\partial K2_{Total}} + \frac{\partial K2Con}{\partial K2} \cdot \frac{\partial K2}{\partial K2_{Total}} + \frac{\partial K2Con}{\partial Ap} \cdot \frac{\partial Ap}{\partial K2_{Total}} + \frac{\partial K2Con}{\partial Bp} \cdot \frac{\partial Bp}{\partial K2_{Total}}$$

Now in order to show the absence of biphasic response in Bp with total kinase concentration ($K2_{Total}$), we proceed with a proof by contradiction. Note that the same procedure of which will be valid to show the absence of substrate biphasic response in modified form Ap with $K1_{Total}$.

If we assume that such a biphasic response exists, then there should exist a steady state of the system where the following should be satisfied (at the biphasic peak)

$$\frac{\partial Bp}{\partial K2_{Total}} = 0$$

Thus, the above expressions obtained after differentiation can be simplified as follows

$$0 = \frac{\partial ACon}{\partial Ap} \cdot \frac{\partial Ap}{\partial K2_{Total}} + \frac{\partial ACon}{\partial K1} \cdot \frac{\partial K1}{\partial K2_{Total}}$$

$$0 = \frac{\partial BCon}{\partial Ap} \cdot \frac{\partial Ap}{\partial K2_{Total}} + \frac{\partial BCon}{\partial K2} \cdot \frac{\partial K2}{\partial K2_{Total}}$$
$$0 = \frac{\partial K1}{\partial K2_{Total}} + \frac{\partial K1Con}{\partial Ap} \cdot \frac{\partial Ap}{\partial K2_{Total}}$$
$$1 = \frac{\partial K2}{\partial K2_{Total}} + \frac{\partial K2Con}{\partial Ap} \cdot \frac{\partial Ap}{\partial K2_{Total}}$$

Note

This simplification is possible since the functions $\frac{\partial ACon}{\partial Bp}, \frac{\partial BCon}{\partial Bp}, \frac{\partial K1Con}{\partial Bp}, \frac{\partial K2Con}{\partial Bp}$ are fintie and always have non-zero denominators (as shown below), and thus the products involving $\frac{\partial Bp}{\partial K2_{Total}}$ can be zero.

$$simplify(diff(ACon, Bp)) = -\frac{P_{Total}\, d_2\, c_2\, (K1\, (k_1 + k_2)\, c_1 + k_2)\, Ap}{(Ap\, c_2 + Bp\, d_2 + 1)^2\, K1\, c_1\, k_1}$$

$$simplify(diff(BCon, Bp)) = \frac{1}{(Ap\, c_2 + Bp\, d_2 + 1)^2\, K2\, d_1\, p_1} \Bigg(K2 \bigg(Bp^2\, p_1\, d_2^2 + 2\, (Ap\, c_2 + 1) \bigg(\bigg(Bp + \frac{P_{Total}}{2}\bigg)\, p_1 + \frac{p_2\, P_{Total}}{2}\bigg)\, d_2 + p_1\, (Ap\, c_2 + 1)^2\bigg)\, d_1 + d_2\, p_2\, P_{Total}\, (Ap\, c_2 + 1)\Bigg)$$

$$simplify(diff(K1Con, Bp)) = -\frac{c_2\, Ap\, P_{Total}\, k_2\, d_2}{(Ap\, c_2 + Bp\, d_2 + 1)^2\, k_1}$$

$$simplify(diff(K2Con, Bp)) = \frac{d_2\, p_2\, P_{Total}\, (Ap\, c_2 + 1)}{(Ap\, c_2 + Bp\, d_2 + 1)^2\, p_1}$$

Now solving the expression obtained by differentiating K1Con above for $\frac{\partial K1}{\partial K2_{Total}}$ and resubstituting it in the first expression (as shown below) we get,

$$0 = \frac{\partial ACon}{\partial Ap} \cdot \frac{\partial Ap}{\partial K2_{Total}} + \frac{\partial ACon}{\partial K1} \cdot \left(-\frac{\partial K1Con}{\partial Ap} \cdot \frac{\partial Ap}{\partial K2_{Total}}\right)$$

which further simplifies to

$$0 = \frac{\partial Ap}{\partial K2_{Total}} \cdot \left(\frac{\partial ACon}{\partial Ap} - \frac{\partial ACon}{\partial K1} \cdot \frac{\partial K1Con}{\partial Ap}\right)$$

This thus leads to the following conclusion, either $\frac{\partial Ap}{\partial K2_{Total}}$ is zero, or $\frac{\partial ACon}{\partial Ap} - \frac{\partial ACon}{\partial K1} \cdot \frac{\partial K1Con}{\partial Ap}$ is zero. The former cannot be possible (see note below).

Note

Suppose, $\frac{\partial Ap}{\partial K2_{Total}}$ is zero at such a point. Then, by analyzing the second, we get $\frac{\partial K2}{\partial K2_{Total}}$ to be equal to 1 (since $\frac{\partial BCon}{\partial Ap}$ & $\frac{\partial BCon}{\partial K2}$ are finite and have non-zero denominators).

$$simplify(diff(BCon, Ap)) = -\frac{(K2\ (p_1 + p_2)\ d_1 + p_2)\ P_{Total}\ d_2\ Bp\ c_2}{(Ap\ c_2 + Bp\ d_2 + 1)^2\ K2\ d_1\ p_1}$$

$$simplify(diff(BCon, K2)) = -\frac{d_2\ Bp\ P_{Total}\ p_2}{(Ap\ c_2 + Bp\ d_2 + 1)\ K2^2\ d_1\ p_1}$$

Then, by analyzing the third expression, we get $\frac{\partial K2}{\partial K2_{Total}}$ $\frac{\partial K1Con}{\partial Ap}$ are finite and have non-zero denominators).

$$simplify(diff(K1Con, Ap)) = \frac{c_2\ P_{Total}\ k_2\ (Bp\ d_2 + 1)}{(c_2\ Ap + Bp\ d_2 + 1)^2\ k_1}$$

This is a contradictino. Thus, the $\frac{\partial Ap}{\partial K2_{Total}}$ is not equal to zero.

However the latter can also not be possible for a feasible steady state of the system (see below).

$simplify(diff(ACon, Ap) - diff(ACon, K1) \cdot diff(K1Con, Ap))$

$$\frac{1}{(Ap\ c_2 + Bp\ d_2 + 1)^3\ K1^2\ c_1\ k_1^2}\Big(c_1\ K1^2\ \Big(Ap^2\ c_2^2 + 2\ \Big(Ap + \frac{P_{Total}}{2}\Big)\ (Bp\ d_2 + 1)\ c_2 + (Bp\ d_2 + 1)^2\Big)\ (Ap\ c_2 + Bp\ d_2 + 1)\ k_1^2 + K1\ c_2\ k_2\ P_{Total}\ (K1\ c_1 + 1)\ (Bp\ d_2 + 1)\ (Ap\ c_2 + Bp\ d_2 + 1)\ k_1 + c_2^2\ Ap\ P_{Total}^2\ k_2^2\ (Bp\ d_2 + 1)\Big) \quad \textbf{(2.2.5)}$$

Since all the parameters and variables involved in the expression are positive, the expression is non-zero always.

We have a contradiction.

Thus, our assumption of the presence of an enzyme biphasic in Bp with $K2_{Total}$ is wrong.

Hence enzyme biphasic cannot exist in the model.

Common kinase separate phosphatase model: Absence of substrate and enzyme biphasic dose response

We initialize the *Maple* file with the *restart* command and load the relevant libraries of inbuilt *Maple* functions (*LinearAlgebra, VectorCalculus, Student[LinearAlgebra]*)

restart : *with*(*LinearAlgebra*) : *with*(*VectorCalculus*) : *with*(*Student*[*LinearAlgebra*]) :

The system is modeled as a set of ODEs using the kinetic nomenclature described in the main text. Here dA represents d[A]/dt and similarly in the case of the other variables. At steady state thus, the right hand

sides of each of these expressions will be equal to zero.

$dA := -k_{b1} \cdot A \cdot K + k_{ub1} \cdot AK + k_2 \cdot ApP1 :$
$dAp := -k_{b2} \cdot Ap \cdot P1 + k_{ub2} \cdot ApP1 + k_1 \cdot AK :$
$dB := -p_{b1} \cdot B \cdot K + p_{ub1} \cdot BK + p_2 \cdot BpP2 :$
$dBp := -p_{b2} \cdot Bp \cdot P2 + p_{ub2} \cdot BpP2 + p_1 \cdot BK :$

$dAK := k_{b1} \cdot A \cdot K - \left(k_{ub1} + k_1\right) \cdot AK :$
$dApP1 := k_{b2} \cdot Ap \cdot P1 - \left(k_{ub2} + k_2\right) \cdot ApP1 :$
$dBK := p_{b1} \cdot B \cdot K - \left(p_{ub1} + p_1\right) \cdot BK :$
$dBpP2 := p_{b2} \cdot Bp \cdot P2 - \left(p_{ub2} + p_2\right) \cdot BpP2 :$

$dK := -k_{b1} \cdot A \cdot K + \left(k_{ub1} + k_1\right) \cdot AK - p_{b1} \cdot B \cdot K + \left(p_{ub1} + p_1\right) \cdot BK :$
$dP1 := -k_{b2} \cdot Ap \cdot P1 + \left(k_{ub2} + k_2\right) \cdot ApP1 :$
$dP2 := -p_{b2} \cdot Bp \cdot P2 + \left(p_{ub2} + p_2\right) \cdot BpP2 :$

The model is also associated with conservation conditions which are described below. Here we store the conservation expressions as ACon, BCon, KCon, P1Con and P2Con for the substrate and the respective enzymes. Each of these expressions is always equal to zero (both in the transient and at steady state).

$ACon := A + Ap + ApP1 + AK - A_{Total} :$
$BCon := B + Bp + BpP2 + BK - B_{Total} :$
$KCon := K + AK + BK - K_{Total} :$
$P1Con := P1 + ApP1 - P1_{Total} :$
$P2Con := P2 + BpP2 - P2_{Total} :$

Now we begin by solving the system of equations to obtain expressions linking the steady state concentrations of the variables, primarily to obtain expressions for the steady state concentrations of variables as a function of concentrations of Ap and Bp. For this purpose we use an inbuilt *Maple* command *solve* as shown with the example below.

$$AK := solve(dAK, AK) = \frac{k_{b1} A K}{k_{ub1} + k_1}$$

We similarly solve for the other variables using the same command.

$assign(solve(\{dBK, dApP1, dBpP2\}, \{BK, ApP1, BpP2\}))$
$assign(solve(\{dA, dB\}, \{A, B\}))$

We introduce the following parameters (c_1, c_2, d_1, and d_2). This is done for the sake of brevity and easy tractability of the expressions obtained.

$k_{b1} := c_1 \cdot \left(k_1 + k_{ub1}\right) : k_{b2} := c_2 \cdot \left(k_2 + k_{ub2}\right) :$
$p_{b1} := d_1 \cdot \left(p_1 + p_{ub1}\right) : p_{b2} := d_2 \cdot \left(p_2 + p_{ub2}\right) :$

Once this is done, we again solve for the steady state of the phosphatases, using the respective conservation expressions (P1Con and P2Con).

$$P1 := solve(P1Con, P1) = \frac{P1_{Total}}{Ap\, c_2 + 1}$$

$$P2 := solve(P2Con, P2) = \frac{P2_{Total}}{Bp\, d_2 + 1}$$

This results in the following expressions for the steady state concentrations of the various species in terms of the steady state substrate concentration Ap and Bp

$$A = \frac{c_2\, Ap\, P1_{Total}\, k_2}{(Ap\, c_2 + 1)\, K\, k_1\, c_1}$$

$$B = \frac{d_2\, Bp\, P2_{Total}\, p_2}{(Bp\, d_2 + 1)\, K\, p_1\, d_1}$$

$$AK = \frac{c_2\, Ap\, P1_{Total}\, k_2}{(Ap\, c_2 + 1)\, k_1}$$

$$BK = \frac{d_2\, Bp\, P2_{Total}\, p_2}{(Bp\, d_2 + 1)\, p_1}$$

$$ApP1 = \frac{c_2\, Ap\, P1_{Total}}{Ap\, c_2 + 1}$$

$$BpP2 = \frac{d_2\, Bp\, P2_{Total}}{Bp\, d_2 + 1}$$

Note that when Ap and Bp are positive, steady state concentrations of the other variable concentrations are positive as well. Thus we have solved the system of equations at steady state to arrive at expressions linking the steady state concentrations of the variables with that of Ap and Bp. We now have three expressions, ACon, BCon and KCon - the conservation of the substrates and kinase, whose solution for the variables define the steady state of the system.

Substrate biphasic

In this subsection we show the absence of substrate biphasic in either of the modified forms of the substrates with their respective total substrate amounts. As mentioned earlier, feasible solutions to the three coupled expressions (ACon, BCon, KCon - shown below) would define the steady state of the system.

Note: Since the two covalent modification cycles are virtually identical to one and other with a shared kinase and two unique phosphatases acting on them, a proof presenting the absence of substrate biphasic in one covalent modification cycle is tantamount to proving the absence in both modification cycle.

$ACon$

$$\frac{c_2\, Ap\, P1_{Total}\, k_2}{(Ap\, c_2 + 1)\, K\, k_1\, c_1} + Ap + \frac{c_2\, Ap\, P1_{Total}}{Ap\, c_2 + 1} + \frac{c_2\, Ap\, P1_{Total}\, k_2}{(Ap\, c_2 + 1)\, k_1} - A_{Total} \quad \textbf{(3.1.1)}$$

$BCon$

$$\frac{d_2\,Bp\,P2_{Total}\,p_2}{(Bp\,d_2+1)\,Kp_1\,d_1}+Bp+\frac{d_2\,Bp\,P2_{Total}}{Bp\,d_2+1}+\frac{d_2\,Bp\,P2_{Total}\,p_2}{(Bp\,d_2+1)\,p_1}-B_{Total} \qquad \textbf{(3.1.2)}$$

$KCon$

$$K+\frac{c_2\,Ap\,P1_{Total}\,k_2}{(Ap\,c_2+1)\,k_1}+\frac{d_2\,Bp\,P2_{Total}\,p_2}{(Bp\,d_2+1)\,p_1}-K_{Total} \qquad \textbf{(3.1.3)}$$

If we are to differentiate the expressions by total substrate amount (B_{Total}) we would have the following expressions

$$\frac{dACon}{dB_{Total}}=0=\frac{\partial ACon}{\partial Ap}\cdot\frac{\partial Ap}{\partial B_{Total}}+\frac{\partial ACon}{\partial K}\cdot\frac{\partial K}{\partial B_{Total}}$$
$$\frac{dBCon}{dB_{Total}}=0=\frac{\partial BCon}{\partial B_{Total}}+\frac{\partial BCon}{\partial Bp}\cdot\frac{\partial Bp}{\partial B_{Total}}+\frac{\partial BCon}{\partial K}\cdot\frac{\partial K}{\partial B_{Total}}$$
$$\frac{dKCon}{dB_{Total}}=0=\frac{\partial KCon}{\partial K}\cdot\frac{\partial K}{\partial B_{Total}}+\frac{\partial KCon}{\partial Ap}\cdot\frac{\partial Ap}{\partial B_{Total}}+\frac{\partial KCon}{\partial Bp}\cdot\frac{\partial Bp}{\partial B_{Total}}$$

Now in order to show the absence of biphasic response in Bp with total substrate concentration, we proceed with a proof by contradiction.

If we assume that such a biphasic response exists, then there should exist a steady state of the system where the following should be satisfied (at the biphasic peak)

$$\frac{\partial Bp}{\partial B_{Total}}=0$$

Thus, the above expressions obtained after differentiation can be simplified as follows

$$0=\frac{\partial ACon}{\partial Ap}\cdot\frac{\partial Ap}{\partial B_{Total}}+\frac{\partial ACon}{\partial K}\cdot\frac{\partial K}{\partial B_{Total}}$$
$$1=\frac{\partial BCon}{\partial K}\cdot\frac{\partial K}{\partial B_{Total}}$$
$$0=\frac{\partial K}{\partial B_{Total}}+\frac{\partial KCon}{\partial Ap}\cdot\frac{\partial Ap}{\partial B_{Total}}$$

Note

This simplification is possible since the functions $\frac{\partial BCon}{\partial Bp}, \frac{\partial KCon}{\partial Bp}$ are finite and always have non-zero denominators (as shown below), and thus the products involving $\frac{\partial Bp}{\partial K_{Total}}$ can be zero.

$$simplify(diff(BCon, Bp)) = \frac{\left(Bp^2\,d_2^2\,p_1+\left((2\,Bp+P2_{Total})\,p_1+p_2\,P2_{Total}\right)d_2+p_1\right)K\,d_1+d_2\,P2_{Total}\,p_2}{(Bp\,d_2+1)^2\,Kp_1\,d_1}$$

$$simplify(diff(KCon, Bp)) = \frac{d_2\,P2_{Total}\,p_2}{(Bp\,d_2+1)^2\,p_1}$$

We also know that $\frac{\partial BCon}{\partial K}$ has a non-zero denominator (see below). Thus we can assert that $\frac{\partial K}{\partial B_{Total}}$ is also non-zero.

$$simplify(diff(BCon, K)) = -\frac{d_2\, Bp\, P2_{Total}\, p_2}{(Bp\, d_2 + 1)\, K^2\, p_1\, d_1}$$

This allows us to solve for it using the expression obtained from differentiating KCon and substituting in the expression belonging to ACon.

$$0 = \frac{\partial ACon}{\partial Ap} \cdot \frac{\partial Ap}{\partial B_{Total}} + \frac{\partial ACon}{\partial K} \cdot \left(-\frac{\partial KCon}{\partial Ap} \cdot \frac{\partial Ap}{\partial B_{Total}} \right)$$

This expression can be further simplified as shown below

$$0 = \frac{\partial Ap}{\partial B_{Total}} \cdot \left(\frac{\partial ACon}{\partial Ap} - \frac{\partial ACon}{\partial K} \frac{\partial KCon}{\partial Ap} \right)$$

Now, in order for this to be true, either the $\frac{\partial Ap}{\partial B_{Total}}$ is zero (which it cannot be - see note below), or $\frac{\partial ACon}{\partial Ap} - \frac{\partial ACon}{\partial K} \frac{\partial KCon}{\partial Ap}$ is zero. We show below that latter can also not be zero for any feasible steady state concentration.

Note

Suppose $\frac{\partial Ap}{\partial B_{Total}}$ is zero. Then, this implies that $\frac{\partial ACon}{\partial K}$ must be zero (this is since, $\frac{\partial K}{\partial B_{Total}}$ is non-zero). However, $\frac{\partial ACon}{\partial K}$ is non-zero as shown below.

$$simplify(diff(ACon, K)) = -\frac{c_2\, Ap\, P1_{Total}\, k_2}{(Ap\, c_2 + 1)\, K^2\, k_1\, c_1}$$

$$simplify(diff(ACon, Ap) - diff(ACon, K) \cdot diff(KCon, Ap)) = \frac{1}{(c_2\, Ap + 1)^3\, K^2\, k_1^2\, c_1} \Bigg(Ap^3\, K^2\, c_1\, c_2^3\, k_1^2 + 3 \bigg(K^2\, c_1 \left(Ap + \frac{P1_{Total}}{3} \right) k_1^2 + \frac{K\, k_2\, P1_{Total}\, (K\, c_1 + 1)\, k_1}{3} + \frac{k_2^2\, P1_{Total}^2}{3} \bigg) Ap\, c_2^2 + 3 \bigg(K\, c_1 \left(Ap + \frac{P1_{Total}}{3} \right) k_1 + \frac{k_2\, P1_{Total}\, (K\, c_1 + 1)}{3} \bigg) k_1\, K\, c_2 + K^2\, k_1^2\, c_1 \Bigg)$$

Since all the parameters and variables involved in the expression are positive, the expression is non-zero always.

We have a contradiction.

Thus, our assumption of the presence of a substrate biphasic in Bp with B_{Total} is wrong.

Hence substrate biphasic cannot exist in the model.

Enzyme biphasic

In this subsection we show the absence of enzyme biphasic in either of the modified forms of the substrates with total kinase concentration (between the respective substrate enzyme pair). As mentioned earlier, feasible solutions to the three coupled expressions (ACon, BCon, KCon - shown below) would define the steady state of the system.

Note: Since the two covalent modification cycles are virtually identical to one and other with a shared kinase and two unique phosphatases acting on them, a proof presenting the absence of enzyme biphasic in one covalent modification cycle is tantamount to proving the absence in both modification cycle.

$ACon$

$$\frac{c_2\, Ap\, P1_{Total}\, k_2}{(Ap\, c_2+1)\, K\, k_1\, c_1} + Ap + \frac{c_2\, Ap\, P1_{Total}}{Ap\, c_2+1} + \frac{c_2\, Ap\, P1_{Total}\, k_2}{(Ap\, c_2+1)\, k_1} - A_{Total} \tag{3.2.1}$$

$BCon$

$$\frac{d_2\, Bp\, P2_{Total}\, p_2}{(Bp\, d_2+1)\, K p_1\, d_1} + Bp + \frac{d_2\, Bp\, P2_{Total}}{Bp\, d_2+1} + \frac{d_2\, Bp\, P2_{Total}\, p_2}{(Bp\, d_2+1)\, p_1} - B_{Total} \tag{3.2.2}$$

$KCon$

$$K + \frac{c_2\, Ap\, P1_{Total}\, k_2}{(Ap\, c_2+1)\, k_1} + \frac{d_2\, Bp\, P2_{Total}\, p_2}{(Bp\, d_2+1)\, p_1} - K_{Total} \tag{3.2.3}$$

Now differentiating each of these expressions with respect to $K1_{Total}$, we get the following

$$\frac{dACon}{dK_{Total}} = 0 = \frac{\partial ACon}{\partial Ap}\cdot\frac{\partial Ap}{\partial K_{Total}} + \frac{\partial ACon}{\partial K}\cdot\frac{\partial K}{\partial K_{Total}}$$

$$\frac{dBCon}{dK_{Total}} = 0 = \frac{\partial BCon}{\partial Bp}\cdot\frac{\partial Bp}{\partial K_{Total}} + \frac{\partial BCon}{\partial K}\cdot\frac{\partial K}{\partial K_{Total}}$$

$$\frac{dKCon}{dK_{Total}} = 0 = \frac{\partial KCon}{\partial K_{Total}} + \frac{\partial KCon}{\partial K}\cdot\frac{\partial K}{\partial K_{Total}} + \frac{\partial KCon}{\partial Ap}\cdot\frac{\partial Ap}{\partial K_{Total}} + \frac{\partial KCon}{\partial Bp}\cdot\frac{\partial Bp}{\partial K_{Total}}$$

Now in order to show the absence of biphasic response in Bp with total kinase concentration (K_{Total}), we proceed with a proof by contradiction. Note that the same procedure of which will be valid to show the absence of substrate biphasic response in modified form Ap with K_{Total}.

If we assume that such a biphasic response exists, then there should exist a steady state of the system where the following should be satisfied (at the biphasic peak)

$$\frac{\partial Bp}{\partial K_{Total}} = 0$$

Thus, the above expressions obtained after differentiation can be simplified as follows

$$0 = \frac{\partial ACon}{\partial Ap}\cdot\frac{\partial Ap}{\partial K_{Total}} + \frac{\partial ACon}{\partial K}\cdot\frac{\partial K}{\partial K_{Total}}$$

$$0 = \frac{\partial BCon}{\partial K} \cdot \frac{\partial K}{\partial K_{Total}}$$

$$1 = \frac{\partial K}{\partial K_{Total}} + \frac{\partial KCon}{\partial Ap} \cdot \frac{\partial Ap}{\partial K_{Total}}$$

Note

This simplification is possible since the functions $\frac{\partial BCon}{\partial Bp}$, $\frac{\partial KCon}{\partial Bp}$ are finite and always have non-zero denominators (as shown below), and thus the products involving $\frac{\partial Bp}{\partial K_{Total}}$ can be zero.

simplify(*diff*(*BCon*, *Bp*)) =

$$\frac{K\left(Bp^2 d_2^2 p_1 + \left(\left(2\,Bp + P2_{Total}\right) p_1 + p_2\,P2_{Total}\right) d_2 + p_1\right) d_1 + d_2\,P2_{Total}\,p_2}{\left(Bp\,d_2 + 1\right)^2 K\,p_1\,d_1}$$

$$simplify(diff(KCon, Bp)) = \frac{d_2\,P2_{Total}\,p_2}{\left(Bp\,d_2 + 1\right)^2 p_1}$$

Now since $\frac{\partial BCon}{\partial K}$ is non-zero (see below), $\frac{\partial K}{\partial K_{Total}}$ must be zero.

$$simplify(diff(BCon, K)) = -\frac{d_2\,Bp\,P2_{Total}\,p_2}{\left(Bp\,d_2 + 1\right) K^2\,p_1\,d_1}$$

Again, since $\frac{\partial ACon}{\partial K}$ has a non-zero denominator (see below), the products involving $\frac{\partial K}{\partial K_{Total}}$ can be zero.

$$simplify(diff(ACon, K)) = -\frac{c_2\,Ap\,P1_{Total}\,k_2}{\left(Ap\,c_2 + 1\right) K^2\,k_1\,c_1}$$

This results in the following expressions after reduction for the differentiated expressions

$$0 = \frac{\partial ACon}{\partial Ap} \cdot \frac{\partial Ap}{\partial K_{Total}}$$

$$1 = \frac{\partial KCon}{\partial Ap} \cdot \frac{\partial Ap}{\partial K_{Total}}$$

However, we have a contradiction here. Both $\frac{\partial ACon}{\partial Ap}$ and $\frac{\partial KCon}{\partial Ap}$ are non-zero and finite respectively (see below)

simplify(*diff*(*ACon*, *Ap*)) =

$$\frac{K\left(Ap^2 c_2^2 k_1 + \left(\left(2\,Ap + P1_{Total}\right) k_1 + k_2\,P1_{Total}\right) c_2 + k_1\right) c_1 + c_2\,P1_{Total}\,k_2}{\left(Ap\,c_2 + 1\right)^2 K\,k_1\,c_1}$$

$$simplify(diff(KCon, Ap)) = \frac{c_2\, P1_{Total}\, k_2}{(Ap\, c_2 + 1)^2\, k_1}$$

However this simultaneously implies that $\frac{\partial Ap}{\partial K_{Total}}$ must be zero and non-zero to satisfy the two remaining expressions from earlier.
We have a contradiction.

Thus, our assumption of the presence of an enzyme biphasic in Bp with K_{Total} is wrong.

Hence enzyme biphasic cannot exist in the model.

Biphasic interactions within common network motifs

The following document includes models N1-N4. As mentioned in the main text each of these models involve a basic 3-node motif representing, a. positive feedback (N1), b. negative feedback (N2), c. incoherent feedforward (N3), and d. coherent feedforward (N4). Each node consists of an active form and an inactive form shuttling between each other. In each of these models, the basic characteristic of the motif is represented following which the role of biphasic interactions in the pathway are analyzed by considering the interaction of the pathway node (responsible for the feedback/feedforward) to be biphasic. All models are constructed using simple mass action kinetics and the biphasic interaction is modelled using the following expression $f(x) = b_1 \cdot x e^{-b_2 \cdot x}$ where a and b are parameters. This ensures a biphasic response is acheieved at x value of $\frac{1}{b_2}$ and a corresponding maximum $f(x)$ of $\left(\frac{b_1}{b_2 \cdot e}\right)$.

Proof of absence of multistability in negative feedback motif (Open System)

$restart : with(LinearAlgebra) : with(VectorCalculus) : with(Student[LinearAlgebra]) :$

In this sub-section we show that the basic negative feedback motif (Open system) used in figure S3, is incapable of exhibiting multistability. To recap; figure S3 shows how the introduction of a simple biphasic response in the feedback interaction can allow the system to present multistability.

The system is modeled as a set of ODEs using the kinetic nomenclature described in the main text (Models and Methods section). Here dA represents d[A]/dt and similarly in the case of the other variables. At steady state thus, the right hand sides of each of these expressions will be equal to zero. The model is described here again (the code for the same can be found in the MatCont package under the name N5__Bi_NFB). In this instance, for the open system, Switch = 0.

$$dR := k_0 \cdot S - k_1 \cdot R \cdot Ap \cdot (1 - Switch) - Switch \cdot R \cdot Ap \cdot b_1 \cdot \exp(-b_2 \cdot Ap) - k_2 \cdot R :$$

$$dAp := \frac{k_3 \cdot A \cdot R}{K_3 + A} - \frac{k_4 \cdot Ap}{K_4 + Ap} :$$

$$dA := -\frac{k_3 \cdot A \cdot R}{K_3 + A} + \frac{k_4 \cdot Ap}{K_4 + Ap} :$$

$Switch := 0 :$

The model is also associated with conservation conditions which are described below. Here we store the conservation expressions as ACon, PCon and KCon for the substrate and the respective enzymes. Each of these expressions is always equal to zero (both in the transient and at steady state).

$ACon := A_{Total} - A - Ap :$

Now we begin solving the system of equations to obtain equations relating the steady state concentrations of the variables. Primarily to obtain equations relating the steady state concentrations of variables A and R. For this purpose we use an inbuilt *Maple* command *solve* as shown with the example below.

$$R := solve(dA, R) = \frac{k_4\, Ap\, (K_3 + A)}{(K_4 + Ap)\, k_3\, A}$$

$assign(solve(\{ACon\}, \{A\}))$:

Now having solved for the steady state of the system in terms of Ap, the only two equation that remains (which defines the steady state of the system) is the differential equation dR

$simplify(numer(-dR))$

$$k_1 k_4 Ap^3 + \left(\left(-K_3 k_1 - k_1 A_{Total} + k_2\right) k_4 - k_0 S k_3\right) Ap^2 + \left(-k_2 \left(A_{Total} + K_3\right) k_4 + S k_0 k_3 \left(A_{Total} - K_4\right)\right) Ap + K_4 S k_0 k_3 A_{Total} \quad \textbf{(1.1)}$$

The feasible solutions of the above equation for Ap defines the steady state of the system. We can also see that the equation is a third degree polynomial in Ap, which can atmost accept three feasible solutions. Note that the feasible solution for Ap should lie between 0 and A_{Total}.

We can also observe that the coeffecient of the third exponent and the constant term are both positive. This implies that one root of the cubic polynomial is necessarily negative. Similarly since the polynomial evaluated at Ap = A_{Total} is negative (see below), there exists exactly one solution.

$simplify\left(eval\left(T, Ap = A_{Total}\right)\right)$

$$-K_3 k_4 A_{Total} \left(k_1 A_{Total} + k_2\right) \quad \textbf{(1.2)}$$

Thus multistability is impossible in the negative feedback motif (open system).

Choice of parameters for biphasic in interaction

In this sub-section we show the rationale used to choose parameter values when comparing the behavior of a network motif with and without biphasic in interaction. The main aim, as stated in the models and methods is to maintain parity in the strength of the interaction modelled, whether using a bilinear response (modelled using the term: $f(x) = k \cdot x$) indicating general motif response, or using a biphasic response modelled using the following term $f(x) = b_1 \cdot x \cdot \exp(-b_2 \cdot x)$

$restart : with(LinearAlgebra) : with(Student[LinearAlgebra]) : with(VectorCalculus)$:

In order to ensure parity in the strength of the interaction. We first assume that the system is operating over the range (for values of x) 0 to a finite value X_{Total}. In such a case, we wish to choose parameters b_1 and b_2 for the biphasic interaction term, such that the averaged strength over 0 to X_{Total} is equal to that arising out of the bilinear (basal models') interaction. This can be written as shown below

$$\int_0^{X_{Total}} f_{Biphasic}(x)\, dx = \int_0^{X_{Total}} f_{basal}(x)\, dx$$

Substituiting the respective terms, this becomes

$$\int_0^{X_{Total}} b_1 \cdot x \cdot \exp\left(-b_2 \cdot x\right) \mathrm{d}x = \int_0^{X_{Total}} k \cdot x \, \mathrm{d}x :$$

Evaluating this expression we get

$$-\frac{b_1 \left(\mathrm{e}^{-b_2 X_{Total}} b_2 X_{Total} + \mathrm{e}^{-b_2 X_{Total}} - 1\right)}{b_2^2} = \frac{k X_{Total}^2}{2}$$

Now we assign b_2 the value of $b_2 = \frac{2^{0.5}}{X_{Total}}$. With this, the above expression becomes

$$0.2065321413 \, b_1 X_{Total}^2 = \frac{k X_{Total}^2}{2}$$

Thus, further simplification of this implies that b1 should take the value as shown below

$$solve\left(0.2065321413 \, b_1 X_{Total}^2 = \frac{k X_{Total}^2}{2}, b_1\right)$$

$$2.420930693 \, k \qquad \textbf{(2.1)}$$

Thus, in order to have parity in the averaged strength of both the biphasic interaction and the basal interaction over the range of operation (0 to X_{Total}). We approximate this and take the value of b_1 and b_2 to be as shown below.

$$\boldsymbol{b_1 := 2.5 \cdot k} \text{ and } \boldsymbol{b_2 := \frac{2^{0.5}}{X_{Total}}}$$

Note: In instances such as the upstream signal regulation not having an explicit total amount (unlike when the total amount, i.e. the range of activation, is evident when the regulation comes from a conserved substrate) the X_{Total} value is assumed to be taken arbitrarily such that it represents approximately half the range over which the bifurcation is carried out.

Biphasic interactions within integral feedback control motif

In this section we provide the following proof pertaining to the effect of biphasic interactions on integral feedback control motif responose.
1. The presence of a biphasic interaction can induce a saturation effect that can result in a reduced range of signal regulation for the model.
2. The biphasic interaction allows for in addition to the saturation effect above a loss of stability in one of the branches of steady state.

We first begin by describing the three relevant models,
1. Basic model of integral feedback control motif
2. The integral feedaback control model with biphasic signal regulation
3. Model with biphasic response within the motif interactions

The first two models are provided for completeness - however please only run the third model as appropriate for the proof that follows. Similar to the other worksheets, please run the model, and one of the proofs (not the whole worksheet together)

Basic model

$restart : with(Student[LinearAlgebra]) : with(VectorCalculus) :$

$dA := kaa * S - kda * A * M :$
$dP := kap * A - kdp :$
$dM := kam * P - kdm * M :$

Biphasic signal regulation model

$restart : with(Student[LinearAlgebra]) : with(VectorCalculus) :$

$dA := b1 * S * \exp(-b2 * S) - kda * A * M :$
$dP := kap * A - kdp :$
$dM := kam * P - kdm * M :$

Biphasic response within motif interactions

$restart : with(Student[LinearAlgebra]) : with(VectorCalculus) :$

$dA := kaa * S - kda * A * M :$
$dP := kap * A - kdp :$
$dM := b1 * P * \exp(-b2 * P) - kdm * M :$

In the subsequent subsections we provide proofs for the following insights.

1. The presence of a biphasic interaction can induce a saturation effect that can result in a reduced range of signal regulation for the model.
2. The biphasic interaction allows for a loss of stability in one of the branches of steady state (in addition to the saturation effect described above).

1. Saturating effect of interaction leads to a reduced range of signal regulation

Prereq: please run only model 3 before running this section.

In order to illustrate this proof, we solve for the system at steady state to obtain relationships between the steady state concentrations of A, and P as shown below. We do this by solving the differential equations for A and P at steady state.

$assign(solve(\{dA, dP\}, \{A, M\}))$

Once we do this and resubstitute, the remaining differential equation for M simplies as follows

$simplify(dM)$

$$\frac{b1\ P\ \mathrm{e}^{-b2\ P}\ kda\ kdp - kdm\ kaa\ S\ kap}{kda\ kdp} \tag{4.1}$$

Solving this equation for the signal value (at steady state) provides the following relationship between any upstream signal and the steady state concentration of P.

$solve(dM, S)$

$$\frac{b1\ P\ \mathrm{e}^{-b2\ P}\ kda\ kdp}{kdm\ kaa\ kap} \tag{4.2}$$

Now, the expression on the right hand side is the biphasic interaction term (in variable P), multiplied by a constant (combination of the kinetic constants). This maximum can be evaluated as a function of the kinetic parameters as shown below,

$$Max\_value\_of\_S := simplify\left(eval\left(\frac{b1\ P\ \mathrm{e}^{-b2\ P}\ kda\ kdp}{kdm\ kaa\ kap}, P = solve\left(diff\left(\frac{b1\ P\ \mathrm{e}^{-b2\ P}\ kda\ kdp}{kdm\ kaa\ kap}, P\right), P\right)\right)\right)$$

$$Max\_value\_of\_S := \frac{b1\ \mathrm{e}^{-1}\ kda\ kdp}{b2\ kdm\ kaa\ kap} \tag{4.3}$$

Thus, the right hand side has a maximum concentration that it can take as P varies. This implies that any signal value beyond that maximum (as given by the equation) cannot be supported as a steady state by the system.

Hence we have shown how the saturation effect of interaction leads to a reduced range of signal regulation. This can also be easily verified with a computation by choosing an input signal beyond the maximum value predicted by the equation above.

2. The biphasic interaction induces a loss of stability in one of the branches of steady state (necessarily)

Prereq: please run only model 3 before running this section.

In order to show this result pertaining to the stability of the steady states, we first make the point that there exists two steady states for any given choice of signal activation from this motif. This is apparent from the earlier steady states observed in proof 1. These steady state concentration relationships are rewritten below

$A := \dfrac{kdp}{kap} : M := \dfrac{kaa\ S\ kap}{kda\ kdp} :$

and $S := \dfrac{b1\ P\ \mathrm{e}^{-b2\ P}\ kda\ kdp}{kdm\ kaa\ kap} :$

Thus for any given value of S, A is fixed, M takes a specific value, and there are two values of P that will satisfy the last equation. This implies that there are two steady state branches (Defined by the steady states of A, M and P) for every signal value.

Having estabished that - we will show below that one of these branches necessarily is unstable, and in particular loses stabilty at the point of inflection (where the biphasic nature of the interaction begins or kicks in).

In order to ascertain information regarding the stability of the steady states, we calculate the Jacobian and the Characteristic polynomial of the system at any given steady state. This is done as shown below.

$J := Jacobian([dA, dP, dM], [A, P, M]) :$

$C := CharacteristicPolynomial(J, x) :$

$T := collect(eval(collect(C, x)), x)$

$$T := x^3 + (M\ kda + kdm)\ x^2 + M\ kda\ kdm\ x - (P\ b2 - 1)\ A\ b1\ kap\ kda\ \mathrm{e}^{-P\ b2} \quad \textbf{(5.1)}$$

From the above expression, for any feasible steady state we can observe that the coeffecient of the leading, second degree and first degree exponent of x are all positive. The roots of the polynomial (solutions) represents the eigen values of the system.

We solve the system to obtain steady state concentrations for A and M (as obtained in proof 1) and we resubstitute it into the expression T.

$assign(solve(\{dA, dM\}, \{M, A\}))$

We now extract the coeffecient of the exponents as shown below,

$C3 := coeff(T, x, 3) : C2 := coeff(T, x, 2) : C1 := coeff(T, x, 1) : C0 := coeff(T, x, 0) :$

Now at a given steady state, for the system to be stable, all eigen values of the steady state should be negative real or be complex conjugates with negative real parts. In this polynomial, this will get determined depending on the sign of the constant term.

However we can see that this term can be negative (giving rise to a unstable steady state).

C0

$$-\frac{(P\,b2-1)\,S\,kaa\,kdm\,kap}{P} \tag{5.2}$$

This constant term is negative when $P\,b2-1$ is positive for a given steady state.

Examining the biphasic in interaction term as shown in the model, we can see that it is precisely negative when the biphasic effect kicks in within the interaction, or rather there is a decreasing function value for increasing input value of P. This can be seen below by checking the sign of the gradient of the function as P changes.

simplify(*diff*(*b1* * *P* * exp(−*b2* * *P*), *P*))

$$-b1\,\mathrm{e}^{-P\,b2}\,(P\,b2-1) \tag{5.3}$$

As we can see from this gradient, when $P\,b2-1$ is positive, the gradient is negative. The system originally as we showed initially has two steady state solutions, one corresponding to the initial phase of the interaction, and the other belonging to the waning / latter phase of the interaction.

Hence proved Thow the biphasic interaction induces a loss of stability in one of the branches of steady state (necessarily).

These two insights show how the waning phase of the biphasic interaction necessarily is unstable. Thus even though the biphasic behavior introduces an additional steady state and non-linearity in to the system.. it only diminishes the range over which signal regualtion is capable and further, the steady state introduced is unstable.

Extracellular Regulated Kinase model (ERK)

Exploring the features and requirements of obtaining biphasic dose response in pYpTErk with total substrate and enzyme amounts

In this file we analytically study the presence of enzyme and substrate biphasic dose response in the doubly phosphorylated Erk (pYpTErk). In particular we establish the following key results regarding them,

1. Enzyme biphasic response in pYpTErk (biphasic behavior in the dose response curve of pYpTErk as Mek_{Total} (K_{Total}) changes): Presence of enzyme biphasic response is shown for a choice of parameters. Further, we show how certain analytical expressions (involving kinetic constants and total phosphatase amount) guarentees the presence of enzyme biphasic for some total concentration of Erk and Mek.

2. Substrate biphasic response (biphasic behavrior in the dose response curve of pYpTErk as Erk_{Total} changes): We show how for any choice of biochemistry kinetics, the Erk model is guarenteed to present with substrate biphasic response in pYpTErk (at some total concentration of Erk and Mek).

We note that the key signature of biphasic behavior in the dose response curve of the system is the presence of a steady state of the system, where the following conditions are satisfied.

$$\frac{dpYpTErk}{dMek_{Total}} = 0 \text{ (for enzyme biphasic in pYpTErk)}$$

$$\frac{dpYpTErk}{dErk_{Total}} = 0 \text{ (for substrate biphasic in pYpTErk)}$$

Please note that we'll be using K_{Total} interchangably for Mek_{Total} (and K for Mek) for the remainder of this script for brevity.

The Erk model: We first describe the mathematical description of the Erk model (adapted from Bluthgen et al., 2018).

Notation: pYErk, pTErk represent singly phosphorylated Erk (at different sites), while pYpTErk represents doubly phosphorylated Erk. C1 is the complex formed by the unphosphorylated Erk and Mek (K). CY2 and CT2 are the complexes formed by the single phosphorylated Erk (pYErk and pTErk respectively) and MeK (K). D2 is the complex formed by the fully phosphorylated Erk (pYpTErk) and the phosphatase. DY1 and DT1 are the complexes formed by the singly phosphorulated Erk (pYErk and pTErk respectively) and the phosphatase. K (Mek) and P represent the free active form of the kinase and phosphatase respectively.

We initialize the *Maple* file with the *restart* command and load the relevant libraries of inbuilt *Maple* functions (*LinearAlgebra, VectorCalculus, Student[LinearAlgebra]*)

restart : *with*(*LinearAlgebra*) : *with*(*VectorCalculus*) : *with*(*Student*[*LinearAlgebra*]) :

The system is modeled as a set of ODEs using the kinetic nomenclature described in the main text. Here dERk represents d[Erk]/dt and similarly in the case of the other variables. At steady state thus, the right hand sides of each of these expressions will be equal to zero.

$$dErk := -k_{b1} \cdot Erk \cdot K + k_{ub1} \cdot C1 + p_4 \cdot DT1 + k_4 \cdot DY1 :$$
$$dC1 := k_{b1} \cdot Erk \cdot K - \left(k_{ub1} + k_1\right) \cdot C1 :$$
$$dpYErk := k_1 \cdot C1 - k_{b4} \cdot pYErk \cdot P + k_{ub4} \cdot DY1 - k_{b2} \cdot pYErk \cdot K + k_{ub2} \cdot CY2 :$$
$$dDY1 := k_{b4} \cdot pYErk \cdot P - \left(k_{ub4} + k_4\right) \cdot DY1 :$$
$$dCY2 := k_{b2} \cdot pYErk \cdot K - \left(k_{ub2} + k_2\right) \cdot CY2 :$$
$$dpYpTErk := k_2 \cdot CY2 - p_{b3} \cdot pYpTErk \cdot P + p_{ub3} \cdot D2 + p_2 \cdot CT2 :$$
$$dD2 := p_{b3} \cdot pYpTErk \cdot P - \left(p_{ub3} + p_3\right) \cdot D2 :$$
$$dCT2 := p_{b2} \cdot pTErk \cdot K - \left(p_{ub2} + p_2\right) \cdot CT2 :$$
$$dpTErk := -p_{b2} \cdot pTErk \cdot K + p_{ub2} \cdot CT2 + p_3 \cdot D2 - p_{b4} \cdot pTErk \cdot P + p_{ub4} \cdot DT1 :$$
$$dDT1 := p_{b4} \cdot pTErk \cdot P - \left(p_{ub4} + p_4\right) \cdot DT1 :$$

$$dK := -k_{b1} \cdot Erk \cdot K + \left(k_{ub1} + k_1\right) \cdot C1 - k_{b2} \cdot pYErk \cdot K + \left(k_{ub2} + k_2\right) \cdot CY2 - p_{b2} \cdot pTErk \cdot K + \left(p_{ub2} + p_2\right) \cdot CT2 :$$
$$dP := -k_{b4} \cdot pYErk \cdot P + \left(k_{ub4} + k_4\right) \cdot DY1 - p_{b3} \cdot pYpTErk \cdot P + \left(p_{ub3} + p_3\right) \cdot D2 - p_{b4} \cdot pTErk \cdot P + \left(p_{ub4} + p_4\right) \cdot DT1 :$$

The model is also associated with conservation conditions which are described below. Here we store the conservation expressions as ACon, P1Con, P2Con and KCon for the substrate and the respective enzymes. Each of these expressions is always equal to zero (both in the transient and at steady state).

$$KCon := K_{Total} - K - C1 - CY2 - CT2 :$$
$$PCon := P_{Total} - P - DY1 - D2 - DT1 :$$
$$ErkCon := Erk_{Total} - Erk - pYErk - pTErk - pYpTErk - C1 - CY2 - CT2 - DY1 - D2 - DT1 :$$

We now solve the system described at steady state to obtain expression linking the steady state concentrations of the various species. Here we use the Maple command *solve*

assign(*solve*({*dC1*, *dCY2*, *dCT2*, *dDY1*, *dD2*, *dDT1*}, {*C1*, *CY2*, *CT2*, *DY1*, *D2*, *DT1*}))

Simultaneously we introduce the following parameters (c_1, c_2, c_4, d_2, d_3, and d_4). This is done for the sake of brevity and easy tractability of the expressions obtained.

$k_{b1} := c_1 \cdot (k_{ub1} + k_1) : k_{b2} := c_2 \cdot (k_{ub2} + k_2) : k_{b4} := c_4 \cdot (k_{ub4} + k_4) :$
$p_{b2} := d_2 \cdot (p_{ub2} + p_2) : p_{b3} := d_3 \cdot (p_{ub3} + p_3) : p_{b4} := d_4 \cdot (p_{ub4} + p_4) :$

Once this is done, we again solve for the steady states of the unmodified and partially modified Erks (in terms of the other variables and parameters).

$assign(solve(\{dErk, dpYErk, dpTErk\}, \{Erk, pYErk, pTErk\}))$

Further we now introduce a ratio, $\epsilon = K/P$ (defined as the ratio of the free enzymes), and solve for the steady state of the free phosphatase (using the conservation expression PCon).

$K := \text{epsilon} \cdot P :$
$P := simplify(solve(PCon, P)) =$

$$\frac{P_{Total}\, \epsilon\, c_2 k_2 (d_2 \epsilon p_2 + d_4 p_4)}{c_2 d_2 k_2 p_2 (d_3\, pYpTErk + 1)\, \epsilon^2 + c_2 d_4 (d_3 (p_3 + p_4)\, pYpTErk + p_4)\, k_2 \epsilon + c_4 d_3 d_4\, pYpTErk\, p_3 p_4}$$

Note: For the sake of brevity of the expressions further, we rename pYpTErk as App.

$pYpTErk := App :$

This systematic solving and renaming results in the following expressions for the steady state concentrations of the various species in terms of the steady state substrate concentration App (pYpTErk) and ϵ.

$$simplify(Erk) = \frac{(\epsilon c_2 k_2 + c_4 k_4)\, d_4 p_4 d_3\, App\, p_3}{k_2 c_2 \epsilon^2 k_1 c_1 (d_2 \epsilon p_2 + d_4 p_4)}$$

$$simplify(pYErk) = \frac{d_4 p_4 d_3\, App\, p_3}{\epsilon c_2 k_2 (d_2 \epsilon p_2 + d_4 p_4)}$$

$$simplify(pTErk) = \frac{d_3\, App\, p_3}{d_2 \epsilon p_2 + d_4 p_4}$$

$simplify(C1) =$

$$\frac{P_{Total} (\epsilon c_2 k_2 + c_4 k_4)\, d_4 p_4 d_3\, App\, p_3}{(c_2 d_2 k_2 p_2 (d_3\, App + 1)\, \epsilon^2 + c_2 d_4 (d_3 (p_3 + p_4)\, App + p_4)\, k_2 \epsilon + c_4 d_3 d_4\, App\, p_3 p_4)\, k_1}$$

$$simplify(CY2) = \frac{c_2 P_{Total} d_4 p_4 d_3\, App\, p_3 \epsilon}{c_2 d_2 k_2 p_2 (d_3\, App + 1)\, \epsilon^2 + c_2 d_4 (d_3 (p_3 + p_4)\, App + p_4)\, k_2 \epsilon + c_4 d_3 d_4\, App\, p_3 p_4}$$

$$simplify(CT2) = \frac{d_2 P_{Total} \epsilon^2 c_2 k_2 d_3\, App\, p_3}{c_2 d_2 k_2 p_2 (App\, d_3 + 1)\, \epsilon^2 + c_2 d_4 (d_3 (p_3 + p_4)\, App + p_4)\, k_2 \epsilon + c_4 d_3 d_4\, App\, p_3 p_4}$$

$$simplify(DY1) = \frac{c_4 P_{Total} d_4 p_4 d_3\, App\, p_3}{c_2 d_2 k_2 p_2 (App\, d_3 + 1)\, \epsilon^2 + c_2 d_4 (d_3 (p_3 + p_4)\, App + p_4)\, k_2 \epsilon + c_4 d_3 d_4\, App\, p_3 p_4}$$

$$simplify(D2) = \frac{d_3\, App\, P_{Total} \epsilon c_2 k_2 (\epsilon d_2 p_2 + d_4 p_4)}{c_2 d_2 k_2 p_2 (App\, d_3 + 1)\, \epsilon^2 + c_2 d_4 (d_3 (p_3 + p_4)\, App + p_4)\, k_2 \epsilon + c_4 d_3 d_4\, App\, p_3 p_4}$$

$$simplify(DY1) = \frac{c_4 P_{Total} d_4 p_4 d_3\, App\, p_3}{c_2 d_2 k_2 p_2 (App\, d_3 + 1)\, \epsilon^2 + c_2 d_4 (d_3 (p_3 + p_4)\, App + p_4)\, k_2 \epsilon + c_4 d_3 d_4\, App\, p_3 p_4}$$

Note that when the steady state concentrations of App and ϵ are positive, the steady state concentrations of the other variable concentrations are positive as well. Thus, so far, we have solved the system of equations at steady state to arrive at expressions linking the steady state concentrations of the variables with that of App and ϵ. We now have two expressions, ErkCon and KCon - the conservation of the Erk and Mek, whose solution for the variables define the steady state of the full system.

Substrate Biphasic

In this subsection, we analytically show the presence of substrate biphasic (in the full system) for any choice of underlying kinetics. As noted earlier, the biphasic behavior is characterized by the following condition being satisfied for some steady state of the system.

$$\frac{\partial App}{\partial Erk_{Total}} = 0$$

We now have two remaining conservations, KCon = 0 & ErkCon = 0 (see below) whose solutions to the variables App and ϵ define the steady state of the system.

If we differentiate both these with respect to the total substrate concentration in the system, we get

$$\frac{dErkCon}{dErk_{Total}} = 0 = \frac{\partial ErkCon}{\partial Erk_{Total}} + \frac{\partial ErkCon}{\partial App} \cdot \frac{\partial App}{\partial Erk_{Total}} + \frac{\partial ErkCon}{\partial \epsilon} \cdot \frac{\partial \epsilon}{\partial Erk_{Total}}$$

$$\frac{dKCon}{dErk_{Total}} = 0 = \frac{\partial KCon}{\partial App} \cdot \frac{\partial App}{\partial Erk_{Total}} + \frac{\partial KCon}{\partial \epsilon} \cdot \frac{\partial \epsilon}{\partial Erk_{Total}}$$

Now in order to show the presence of a substrate biphasic response and study its features, we begin by first imposing the necessary features that the system must satisfy for the behavior to exist. We begin with the basic tenet that for the behavior there should exist a steady state of the system where $\frac{\partial App}{\partial Erk_{Total}} = 0$ is satisfied.
At this point then, the above expressions simply as follows

$$1 = \frac{\partial ErkCon}{\partial \epsilon} \cdot \frac{\partial \epsilon}{\partial Erk_{Total}}$$

$$0 = \frac{\partial KCon}{\partial \epsilon} \cdot \frac{\partial \epsilon}{\partial Erk_{Total}}$$

Note

This simplification is possible since the functions $\frac{\partial ErkCon}{\partial App}, \frac{\partial KCon}{\partial App}$ are finite and always have non-zero denominators (as shown below), and thus the products involving $\frac{\partial App}{\partial Erk_{Total}}$ can be zero.

$simplify(diff(ErkCon, App)) =$

$$\Bigg(-d_2^3 \left(App^2 p_2 d_3^2 + \left(p_3 P_{Total} + 2\left(App + \frac{P_{Total}}{2}\right) p_2\right) d_3 + p_2\right) k_1 c_2^3 p_2^2 k_2^3 c_1 \epsilon^7$$

$$- d_2^2 c_2^3 \Bigg(\Bigg(App^2 p_2 p_3 d_3^3 k_1 + 2 k_1 App \left(App \left(p_3 + \frac{3 p_4}{2}\right) d_4 + p_3\right) p_2 d_3^2$$

$$+ \Bigg(\Bigg(\left(P_{Total} (p_2 + 2 k_1) p_3 + 6 k_1 \left(App + \frac{P_{Total}}{2}\right) p_2\right) p_4 + 2 k_1 \left(App + \frac{P_{Total}}{2}\right) p_3 p_2\Bigg) d_4$$

$$+ p_2 p_3 k_1 \Big) d_3 + 3\, p_2 p_4 d_4 k_1 \Big) k_2 + p_2 p_3 p_4 P_{Total} d_3 d_4 k_1 \Big) p_2 k_2^2 c_1 \epsilon^6$$

$$- 2\, d_2 c_2^2 d_4 \Bigg(\Bigg(\Bigg(\Bigg(\frac{\left(\left(2\, k_1 c_1 + p_2 d_2 \right) p_4 + 2\, p_3 c_1 k_1 \right) App^2 p_3 p_2 d_3^3}{2}$$

$$+ \frac{1}{2} \left(App\, p_2 \left(App\, c_1 k_1 \left(p_3 + 3\, p_4 \right) \left(p_3 + p_4 \right) d_4 + 2\, \left(\left(2\, k_1 c_1 + p_2 d_2 \right) p_4 \right.\right.\right.$$

$$\left.\left.\left. + p_3 c_1 k_1 \right) p_3 \right) d_3^2 \right) + 2\, p_4 \Bigg(\Bigg(\Bigg(\frac{P_{Total} \left(p_2 + \frac{k_1}{2} \right) p_3}{2} + \frac{3\, k_1 \left(App + \frac{P_{Total}}{2} \right) p_2}{2} \Bigg) p_4$$

$$+ k_1 \left(App + \frac{P_{Total}}{2} \right) p_3 p_2 \Bigg) c_1 d_4 + \frac{p_2 p_3 \left(2\, k_1 c_1 + p_2 d_2 \right)}{4} \Bigg) d_3 + \frac{3\, p_2 p_4^2 c_1 d_4 k_1}{2} \Bigg) k_2$$

$$+ p_2 p_3 p_4^2 P_{Total} c_1 d_3 d_4 k_1 \Bigg) c_2 + d_2 p_4 \Bigg(\frac{App^2 d_3^2 k_1}{2} + App\, k_1 \left(App\, c_4 + 1 \right) d_3 + App\, c_4 k_1$$

$$+ \left(\frac{1}{2} + \frac{P_{Total} c_4}{2} \right) k_1 + \frac{k_4 P_{Total} c_4}{2} \Bigg) p_3 d_3 p_2^2 c_1 \Bigg) k_2^2 \epsilon^5 - c_2^2 d_4 \Bigg(2 \Bigg(\Bigg(\Bigg(\Bigg(\Bigg(p_2 d_2$$

$$+ \frac{k_1 c_1}{2} \Bigg) p_4 + \frac{p_3 c_1 k_1}{2} \Bigg) App^2 p_3 \left(p_3 + p_4 \right) d_3^3$$

$$+ \frac{p_4 App \left(App\, c_1 k_1 \left(p_3 + p_4 \right)^2 d_4 + 2\, \left(\left(k_1 c_1 + 2\, p_2 d_2 \right) p_4 + p_3 \left(k_1 c_1 + p_2 d_2 \right) \right) p_3 \right) d_3^2}{2}$$

$$+ p_4^2 \Bigg(\Bigg(\Bigg(\frac{p_3 P_{Total}}{2} + k_1 \left(App + \frac{P_{Total}}{2} \right) \Bigg) \Bigg) p_4 + p_3 k_1 \left(App + \frac{P_{Total}}{2} \right) \Bigg) c_1 d_4 + p_3 \Bigg(p_2 d_2$$

$$+ \frac{k_1 c_1}{2} \Bigg) \Bigg) d_3 + \frac{p_4^3 c_1 d_4 k_1}{2} \Bigg) k_2 + \frac{p_3 p_4^3 P_{Total} c_1 d_3 d_4 k_1}{2} \Bigg) d_4 c_2 + d_2 p_4 \Bigg(\Big(2\, k_1 c_1 \Big(p_3$$

$$+ p_4 \Big) d_4 + c_4 \left(2\, p_3 c_1 k_1 + k_4 p_2 d_2 \right) \Big) App^2 d_3^2 + 2\, App \Big(k_1 c_1 \left(p_3 + 2\, p_4 \right) \left(App\, c_4 + 1 \right) d_4$$

$$+ c_4 \left(p_3 c_1 k_1 + k_4 p_2 d_2 \right) \Big) d_3 + 4\, p_4 \Bigg(App\, c_4 k_1 + \left(\frac{1}{2} + \frac{P_{Total} c_4}{2} \right) k_1 + \frac{k_4 P_{Total} c_4}{2} \Bigg) c_1 d_4$$

$$+ k_4 p_2 c_4 d_2 \Bigg) p_3 d_3 p_2 \Bigg) k_2^2 \epsilon^4 - 2\, p_4 c_2 p_3 d_3 \Bigg(\frac{\left(d_3 \left(p_3 + p_4 \right) App + p_4 \right)^2 d_4 k_2^2 c_2^2}{2}$$

$$+ \Bigg(\Bigg(\frac{k_1 c_1 \left(p_3 + p_4 \right)^2 d_4}{2} + \Big(\Big(p_3 \left(k_1 c_1 + p_2 d_2 \right) + k_4 p_2 d_2 \Big) p_4 + p_3 \Big(p_3 c_1 k_1$$

$$+ k_4 p_2 d_2 \Big) \Big) c_4 \Bigg) App^2 d_3^2 + App \Big(p_4 c_1 k_1 \left(p_3 + p_4 \right) \left(App\, c_4 + 1 \right) d_4 + \Big(\Big(p_3 \Big(k_1 c_1$$

$$+ p_2 d_2 \Big) + 2\, k_4 p_2 d_2 \Big) p_4 + k_4 p_2 p_3 d_2 \Big) c_4 \Big) d_3 + p_4 \Bigg(p_4 \Bigg(App\, c_4 k_1 + \left(\frac{1}{2} + \frac{P_{Total} c_4}{2} \right) k_1$$

$$+ \frac{k_4 P_{Total} c_4}{2} \Bigg) c_1 d_4 + k_4 p_2 c_4 d_2 \Bigg) \Bigg) k_2 c_2$$

$$+ \frac{App\, p_2 p_3 p_4 c_1 c_4 d_2 d_3 k_1 \left(App\, c_4 + 2\, App\, d_3 + 2 \right)}{2} \Bigg) d_4^2 k_2 \epsilon^3$$

$$- 2 p_4 c_2 p_3 d_3 d_4^2 k_2 c_4 \Big(\frac{1}{2} ((d_3 (p_3 + p_4) App + p_4) ((((k_4 + 2 p_3) p_4$$
$$+ k_4 p_3) App\, d_3 + k_4 p_4) d_4 k_2 c_2) + p_4 App\, p_3 d_3 ((k_1 c_1 (p_3 + p_4) d_4 + c_4 (k_4 p_2 d_2$$
$$+ \frac{p_3 c_1 k_1}{2})) App\, d_3 + \frac{p_4 c_1 k_1 (App\, c_4 + 2) d_4}{2} + k_4 p_2 c_4 d_2)) \epsilon^2 - 2 p_4^2 ((((k_4$$
$$+ \frac{p_3}{2}) p_4 + k_4 p_3) App\, d_3 + k_4 p_4) k_2 c_2 + \frac{App\, p_3 p_4 c_1 d_3 k_1}{2}) App\, p_3^2 d_3^2 d_4^3 c_4^2 \epsilon$$
$$- App^2 c_4^3 d_3^3 d_4^3 k_4 p_3^3 p_4^3 \Big) \Big/ (k_1 c_2 (c_2 d_2 k_2 p_2 (App\, d_3 + 1) \epsilon^2 + d_4 (d_3 (p_3$$
$$+ p_4) App + p_4) c_2 k_2 \epsilon + c_4 d_3 d_4 App\, p_3 p_4)^2 \epsilon^2 k_2 c_1 (d_2 \epsilon p_2 + d_4 p_4))$$

simplify(*diff*(*KCon*, *App*)) =

$$(d_3 (p_2 \epsilon^3 c_2 d_2 k_1 k_2 - ((-p_3 - p_4) d_4 + p_3 d_2) k_2 k_1 c_2 \epsilon^2 - ((k_1 + k_2) c_2 - k_1 c_4) p_4 d_4 p_3 \epsilon$$
$$- p_3 p_4 c_4 d_4 k_4) \epsilon k_2 P_{Total} (d_2 \epsilon p_2 + d_4 p_4) c_2) \Big/ (k_1 (c_2 d_2 k_2 p_2 (App\, d_3 + 1) \epsilon^2$$
$$+ k_2 ((App\, d_3 + 1) p_4 + App\, d_3 p_3) d_4 c_2 \epsilon + c_4 d_3 d_4 App\, p_3 p_4)^2)$$

Observing, the second expression above (from differentiation of the total kinase concentration) we can see that either $\frac{\partial KCon}{\partial \epsilon}$ or $\frac{\partial \epsilon}{\partial Erk_{Total}}$ must be equal to zero. However, $\frac{\partial \epsilon}{\partial Erk_{Total}}$ cannot be zero as, if it was indeed zero, then since the denominator of $\frac{\partial ErkCon}{\partial \epsilon}$ is non-zero, there would be a contradiction with the first expression (from differentiation of the total Erk concentration).

Thus, $\frac{\partial KCon}{\partial \epsilon}$ must be equal to zero. This expression is evaluated below and stored as T.

$$T := simplify\left(\frac{numer(simplify((diff(KCon, \mathrm{epsilon}))))}{P_{Total} \cdot c_2} \right)$$

$$T := \epsilon^2 c_2 (-(d_3 (p_3 + p_4) App + p_4) k_1 p_4 d_4^2 + d_2 (p_3 App^2 ((p_2 - k_1) p_4 - p_3 k_1) d_3^2 \qquad \textbf{(1.1)}$$
$$+ App ((-2 k_1 p_2 \epsilon + p_3 (p_2 - k_1)) p_4 - 2 k_1 p_2 \epsilon p_3) d_3 - 2 p_2 p_4 \epsilon k_1) d_4$$
$$- p_2^2 \epsilon^2 d_2^2 k_1 (d_3 App + 1)) k_2^2 + p_3 App\, d_3 p_4 \Big(- \Big(App ((p_3 - k_4) p_4 - k_4 p_3) d_3$$
$$+ 2 \left(\epsilon k_1 - \frac{k_4}{2} \right) p_4 \Big) c_4 d_4 + \epsilon d_2 (App (p_2 \epsilon c_2 k_1 + 2 c_4 (-p_3 k_1 + k_4 p_2)) d_3 + (k_1 (c_2$$
$$- 3 c_4) \epsilon + 2 c_4 k_4) p_2) \Big) d_4 k_2 - App^2 c_4 d_3^2 d_4^2 k_1 p_3^2 p_4^2$$

Thus as long as the above equation (expression T) is satsifed, there exists a steady state at some K_{Total} and Erk_{Total} corresponding to the conservation equations, where there exists a biphasic peak (behavior) in the dose response.

Now in order to show that the expression T is indeed satisfied for every choice of underlying kinetics, we rewrite $App = m \cdot \epsilon$

$App := m \cdot \mathrm{epsilon}$:

This simplifies the expression T as shown below.

$simplify(T)$

$$\Big(-m\,p_2^2\,\epsilon^4\,c_2\,d_2^2\,d_3\,k_1\,k_2^2 + c_2\,\Big(\Big(d_3\,\Big(\Big(\big(p_2-k_1\big)\,p_4 - p_3\,k_1\Big)\,p_3\,m\,d_3 - 2\,p_2\,k_1\,\big(p_3 + p_4\big)\Big)\,m\,d_4 - p_2^2\,d_2\,k_1\Big)\,k_2 + m^2\,p_2\,p_3\,p_4\,d_3^2\,d_4\,k_1\Big)\,d_2\,k_2\,\epsilon^3 + 2\,p_4\,\Bigg(\frac{c_2\,\big(-m\,d_3\,k_1\,(p_3+p_4)\,d_4 + \big(m\,p_3\,(p_2-k_1)\,d_3 - 2\,p_2\,k_1\big)\,d_2\big)\,k_2}{2} + \Bigg(m\,c_4\,\big(-p_3\,k_1 + k_4\,p_2\big)\,d_3 + \frac{p_2\,k_1\,(c_2 - 3\,c_4)}{2}\Bigg)\,d_3\,p_3\,m\,d_2\Bigg)\,d_4\,k_2\,\epsilon^2 - \Big(p_4\,c_2\,d_4\,k_1\,k_2^2 + d_3\,c_4\,p_3\,m\,\Big(\Big(\Big(\big(p_3-k_4\big)\,p_4 - p_3\,k_4\Big)\,m\,d_3 + 2\,p_4\,k_1\Big)\,d_4 - 2\,p_2\,d_2\,k_4\Big)\,k_2 + m^2\,p_3^2\,p_4\,c_4\,d_3^2\,d_4\,k_1\Big)\,p_4\,d_4\,\epsilon + m\,p_3\,p_4^2\,c_4\,d_3\,d_4^2\,k_2\,k_4\Big)\,\epsilon \qquad \textbf{(1.2)}$$

We can observe that the expression is now a fifth order polynomial in ϵ. One of the roots factors out resulting in a simplified quartic polynomial in ϵ, with the constant term always positive and the leading coeffecient always having a negative sign. This indicates that for any choice of value for m (which appears in all the coeffecients) there exists a postive solution in ϵ for this quartic expression.

This indicates that for every choice of underlying kinetics and total phosphatase concentration, expression T = 0 can be satisfied (guarenteeing the existence of biphasic response in the dose response for some total concentrations of ErkTotal and KTotal which can be arrived at by evaluating the respective conservation expressions).

Thus we have shown how substrate biphasic response is guarenteed to exist in pYpTErk, for some total concentration of Erk and Kinase in the system, for any choice of underlying kinetics.

Note: We illustrate the validity of this argument by providing an example of a prediction of the presence of a biphasic response for an arbitraty value of 1 for m (i.e. App = ϵ) and some abitrary choice of kinetics (all values are equal to 1), validated by computational bifurcation analysis.

$m := 1 :$

$k_1 := 1 : k_2 := 1 : k_4 := 1 : p_2 := 1 : p_3 := 1 : p_4 := 1 :$

$k_{b1} := 1 : k_{b2} := 1 : k_{b4} := 1 : p_{b2} := 1 : p_{b3} := 1 : p_{b4} := 1 : k_{ub1} := 1 : k_{ub2} := 1 : k_{ub4} := 1 : p_{b2} := 1 : p_{b3} := 1 : p_{b4} := 1 : p_{ub2} := 1 : p_{ub3} := 1 : p_{ub4} := 1 :$

$c_1 := \frac{k_{b1}}{k_1 + k_{ub1}} : c_2 := \frac{k_{b2}}{k_2 + k_{ub2}} : c_4 := \frac{k_{b4}}{k_4 + k_{ub4}} : d_3 := \frac{p_{b3}}{p_3 + p_{ub3}} : d_2 := \frac{p_{b2}}{p_2 + p_{ub2}} : d_4 := \frac{p_{b4}}{p_4 + p_{ub4}} :$

$P_{Total} := 1 :$

$\epsilon := evalf(solve(T, useassumptions) \text{ assuming } \epsilon > 0) = 0.236067977$

$App = 0.236067977$

$ErkCon = Erk_{Total} - 6.236067987$

$KCon = K_{Total} - 0.5278640449$

The computational evidence of the substrate biphasic dose response behavior predicted above is shown in figure 4.

Enzyme biphasic

In this subsection, we analytically show the presence of enzyme biphasic (in the full system) for some choice of kinetic parameter values. We further show the existence of an analytical condition involving kinetic constants that will guarantee the presense of biphasic dose response in pYpTErk with Mek_{Total} (K_{Total}). As noted earlier, the biphasic behavior is characterized by the following condition being satisfied for some steady state of the system.

$$\frac{\partial App}{\partial K_{Total}} = 0$$

We now have two remaining conservations, KCon = 0 & ErkCon = 0 (see below) whose solutions to the variables App and ϵ define the steady state of the system.

If we differentiate these both with respect to the total enzyme (kinase/Mek) concentration in the system, we get

$$\frac{dErkCon}{dK_{Total}} = 0 = \frac{\partial ErkCon}{\partial App} \cdot \frac{\partial App}{\partial K_{Total}} + \frac{\partial ErkCon}{\partial \epsilon} \cdot \frac{\partial \epsilon}{\partial K_{Total}}$$
$$\frac{dKCon}{dK_{Total}} = 0 = \frac{\partial KCon}{\partial K_{Total}} + \frac{\partial KCon}{\partial App} \cdot \frac{\partial App}{\partial K_{Total}} + \frac{\partial KCon}{\partial \epsilon} \cdot \frac{\partial \epsilon}{\partial K_{Total}}$$

Now in order to show the presence of a substrate biphasic response and study its features, we begin with exploring the necessary features that the system must satisfy for the behavior to exist. We begin with the basic tenet that for the behavior there should exist a steady state of the system where $\frac{\partial App}{\partial K_{Total}} = 0$ is satisfied.

At this point then, the above expressions simply as follows

$$0 = \frac{\partial ErkCon}{\partial \epsilon} \cdot \frac{\partial \epsilon}{\partial K_{Total}}$$
$$1 = \frac{\partial KCon}{\partial \epsilon} \cdot \frac{\partial \epsilon}{\partial K_{Total}}$$

Note

This simplification is possible since the functions $\frac{\partial ErkCon}{\partial App}, \frac{\partial KCon}{\partial App}$ are finite and always have non-zero denominators (as shown below), and thus the products involving $\frac{\partial App}{\partial Erk_{Total}}$ can be zero.

simplify(*diff*(*ErkCon*, *App*)) =

$$\Bigg(-k_1 \left(App^2 p_2 d_3^2 + \left(p_3 P_{Total} + 2 p_2 \left(App + \frac{P_{Total}}{2} \right) \right) d_3 + p_2 \right) p_2^2 d_2^3 k_2^3 c_2^3 c_1 \epsilon^7$$
$$- p_2 \left(\left(App^2 p_2 p_3 d_3^3 k_1 + 2 k_1 p_2 App \left(App \left(p_3 + \frac{3 p_4}{2} \right) d_4 + p_3 \right) d_3^2 + \left(\left(\left(P_{Total} (p_2 \right.\right.\right.\right.\right.$$

$$+ 2 k_1) p_3 + 6 k_1 p_2 \left(App + \frac{P_{Total}}{2}\right)) p_4 + 2 p_3 k_1 p_2 \left(App + \frac{P_{Total}}{2}\right)) d_4 + p_2 p_3 k_1) d_3$$

$$+ 3 p_2 p_4 d_4 k_1) k_2 + p_2 p_3 p_4 P_{Total} d_3 d_4 k_1) d_2^2 k_2^2 c_2^3 c_1 \epsilon^6$$

$$- 2 d_2 d_4 (((\frac{p_3 p_2 App^2 ((2 k_1 c_1 + p_2 d_2) p_4 + 2 p_3 c_1 k_1) d_3^3}{2}$$

$$+ \frac{1}{2} (p_2 App (App c_1 k_1 (p_3 + 3 p_4) (p_3 + p_4) d_4 + 2 p_3 ((2 k_1 c_1 + p_2 d_2) p_4$$

$$+ p_3 c_1 k_1)) d_3^2) + 2 p_4 ((((\frac{\left(p_2 + \frac{k_1}{2}\right) P_{Total} p_3}{2} + \frac{3 k_1 p_2 \left(App + \frac{P_{Total}}{2}\right)}{2}) p_4$$

$$+ p_3 k_1 p_2 \left(App + \frac{P_{Total}}{2}\right)) c_1 d_4 + \frac{p_2 p_3 (2 k_1 c_1 + p_2 d_2)}{4}) d_3 + \frac{3 p_2 p_4^2 c_1 d_4 k_1}{2}) k_2$$

$$+ p_2 p_3 p_4^2 P_{Total} c_1 d_3 d_4 k_1) c_2 + p_3 p_4 p_2^2 (\frac{App^2 d_3^2 k_1}{2} + App k_1 (App c_4 + 1) d_3$$

$$+ App c_4 k_1 + \left(\frac{1}{2} + \frac{P_{Total} c_4}{2}\right) k_1 + \frac{k_4 P_{Total} c_4}{2}) d_2 d_3 c_1) k_2^2 c_2^2 \epsilon^5$$

$$- (2 d_4 (((p_3 ((p_2 d_2 + \frac{k_1 c_1}{2}) p_4 + \frac{p_3 c_1 k_1}{2}) App^2 (p_3 + p_4) d_3^3$$

$$+ \frac{p_4 App (App c_1 k_1 (p_3 + p_4)^2 d_4 + 2 p_3 ((k_1 c_1 + 2 p_2 d_2) p_4 + p_3 (k_1 c_1 + p_2 d_2))) d_3^2}{2}$$

$$+ (((\frac{p_3 P_{Total}}{2} + k_1 \left(App + \frac{P_{Total}}{2}\right)) p_4 + p_3 k_1 \left(App + \frac{P_{Total}}{2}\right)) c_1 d_4 + p_3 (p_2 d_2$$

$$+ \frac{k_1 c_1}{2})) p_4^2 d_3 + \frac{p_4^3 c_1 d_4 k_1}{2}) k_2 + \frac{p_3 p_4^3 P_{Total} c_1 d_3 d_4 k_1}{2}) c_2$$

$$+ p_3 p_4 p_2 d_2 (App^2 (2 k_1 c_1 (p_3 + p_4) d_4 + c_4 (2 p_3 c_1 k_1 + k_4 p_2 d_2)) d_3^2$$

$$+ 2 App (k_1 c_1 (p_3 + 2 p_4) (App c_4 + 1) d_4 + c_4 (p_3 c_1 k_1 + k_4 p_2 d_2)) d_3 + 4 p_4 (App c_4 k_1$$

$$+ \left(\frac{1}{2} + \frac{P_{Total} c_4}{2}\right) k_1 + \frac{k_4 P_{Total} c_4}{2}) c_1 d_4 + k_4 p_2 c_4 d_2) d_3) d_4 k_2^2 c_2^2 \epsilon^4$$

$$- 2 (\frac{(d_3 (p_3 + p_4) App + p_4)^2 d_4 k_2^2 c_2^2}{2} + (App^2 (\frac{k_1 c_1 (p_3 + p_4)^2 d_4}{2}$$

$$+ c_4 ((p_3 (k_1 c_1 + p_2 d_2) + k_4 p_2 d_2) p_4 + p_3 (p_3 c_1 k_1 + k_4 p_2 d_2)))) d_3^2$$

$$+ App (p_4 c_1 k_1 (p_3 + p_4) (App c_4 + 1) d_4 + ((p_3 (k_1 c_1 + p_2 d_2) + 2 k_4 p_2 d_2) p_4$$

$$+ k_4 p_2 p_3 d_2) c_4) d_3 + p_4 (p_4 (App c_4 k_1 + \left(\frac{1}{2} + \frac{P_{Total} c_4}{2}\right) k_1 + \frac{k_4 P_{Total} c_4}{2}) c_1 d_4$$

$$+ k_4 p_2 c_4 d_2 \Big) \Big) k_2 c_2 + \frac{App\, p_2 p_3 p_4 c_1 c_4 d_2 d_3 k_1 \left(App\, c_4 + 2\, App\, d_3 + 2\right)}{2} \Big)$$

$$p_3 p_4 d_4^2 k_2 d_3 c_2 \epsilon^3$$

$$- 2\, p_3 p_4 c_4 d_4^2 \Bigg(\frac{1}{2} \Big(\Big(d_3 \left(p_3 + p_4\right) App + p_4 \Big) d_4 \Big(\Big(\Big(k_4 + 2\, p_3 \Big) p_4 + k_4 p_3 \Big) App\, d_3$$

$$+ k_4 p_4 \Big) k_2 c_2 \Big) + p_3 p_4 App \left(App \left(k_1 c_1 \left(p_3 + p_4\right) d_4 + c_4 \left(k_4 p_2 d_2 + \frac{p_3 c_1 k_1}{2} \right) \right) d_3\right.$$

$$\left. + \frac{p_4 c_1 k_1 \left(App\, c_4 + 2\right) d_4}{2} + k_4 p_2 c_4 d_2 \right) d_3 \Bigg) k_2 d_3 c_2 \epsilon^2 - 2\, p_3^2 p_4^2 App\, c_4^2 d_4^3 \Bigg(k_2 \Bigg(\Bigg(\Bigg(k_4$$

$$+ \frac{p_3}{2} \Bigg) p_4 + k_4 p_3 \Bigg) App\, d_3 + k_4 p_4 \Bigg) c_2 + \frac{App\, p_3 p_4 c_1 d_3 k_1}{2} \Bigg) d_3^2 \epsilon$$

$$- App^2 c_4^3 d_3^3 d_4^3 k_4 p_3^3 p_4^3 \Bigg) \Bigg/ \Big(k_1 \epsilon^2 \Big(c_2 d_2 k_2 p_2 \left(App\, d_3 + 1\right) \epsilon^2 + c_2 d_4 \Big(d_3 \Big(p_3$$

$$+ p_4 \Big) App + p_4 \Big) k_2 \epsilon + c_4 d_3 d_4 App\, p_3 p_4 \Big)^2 k_2 \left(\epsilon d_2 p_2 + d_4 p_4\right) c_2 c_1 \Big)$$

$simplify(diff(KCon, App)) =$

$$\Big(k_2 P_{Total} \epsilon \Big(p_2 \epsilon^3 c_2 d_2 k_1 k_2 - k_2 \Big(\left(-p_3 - p_4\right) d_4 + p_3 d_2 \Big) k_1 c_2 \epsilon^2 - p_4 p_3 \Big(\left(k_1 + k_2\right) c_2$$

$$- k_1 c_4 \Big) d_4 \epsilon - p_3 p_4 c_4 d_4 k_4 \Big) d_3 \left(\epsilon d_2 p_2 + d_4 p_4\right) c_2 \Big) \Big/ \Big(\Big(c_2 d_2 k_2 p_2 \left(App\, d_3 + 1\right) \epsilon^2$$

$$+ k_2 \Big(\left(App\, d_3 + 1\right) p_4 + d_3 App\, p_3 \Big) d_4 c_2 \epsilon + c_4 d_3 d_4 App\, p_3 p_4 \Big)^2 k_1 \Big)$$

Observing, the first expression above (from differentiation of the total Erk concentration) we can see that either $\frac{\partial ErkCon}{\partial \epsilon}$ or $\frac{\partial \epsilon}{\partial K_{Total}}$ must be equal to zero. However, $\frac{\partial \epsilon}{\partial K_{Total}}$ cannot be zero as, if it was indeed zero, then since the denominator of $\frac{\partial KCon}{\partial \epsilon}$ is non-zero, there would be a contradiction with the first expression (from differentiation of the total Erk concentration).

Thus, $\frac{\partial ErKCon}{\partial \epsilon}$ must be equal to zero. This expression (a polynomial in App and ϵ) is calculated as shown below and stored as T.

$T := simplify(numer(simplify(diff(ErkCon, \text{epsilon})))) :$

Now rewriting App as m/ϵ allows us to simplify T as a ninth degree polynomial in epsilon (whose coeffecients are functions of the variable m).

$$App := \frac{m}{\text{epsilon}} :$$

However, this rearrangement allows us to isolate and observe the leading coeffecient and constant term of the polynomial as shown below.

$T := simplify(T \cdot \epsilon^3) :$

$C0 := simplify(coeff(T, \text{epsilon}, 0)) = 2\, p_3^3 m^3 d_4^4 p_4^4 c_4^3 d_3^3 k_4$

$C9 := simplify(coeff(T, \text{epsilon}, 9)) =$

$$-p_3 k_2^2 m\, d_2^3 d_3 \Big(\Big(\Big(-d_4 \left(p_4 + k_1\right) P_{Total} - k_1 \Big) p_2 + p_4 P_{Total} d_4 k_1 \Big) k_2 - d_4 k_1 p_2 p_4 P_{Total} \Big) p_2^2 c_2^3 c_1$$

We can notice that the sign of C0 is always positive for all choices of kinetic parameters and feasible values of m, while the sign of C9 is decided purely by P_{Total} and the kinetic constants (and not m). We

can use this insight to conclude that, should C9 be negative in sign, then there exists necessarily a positive feasible solution of ϵ for every feasible value m, thereby guarenteeing the existence of some feasible App and ϵ where the expression is 0 (indicating the presence of biphasic behavior in the dose response of pYpTErk with K_{Total}).

i.e. If C9<0 then there is the guarenteed existence of biphasic dose response with kinase for some total concentrations. We further isolate this condition from C9 as shown below (stored as the expression *Condition*),

$$Condition := collect\Big(-\big(\big(\big((-d_4\,(p_4+k_1)\,P_{Total}-k_1)\,p_2+p_4\,P_{Total}\,d_4\,k_1\big)\,k_2-d_4\,k_1\,p_2\,p_4\,P_{Total}\big)\big) < 0, P_{Total}\Big)$$
$$= \big(-(-d_4\,(p_4+k_1)\,p_2+p_4\,d_4\,k_1)\,k_2+p_2\,p_4\,d_4\,k_1\big)\,P_{Total}+k_1\,k_2\,p_2 < 0$$

Now, when *Condition* is negative, the signs of C9 and C0 are negative and positive respectively indicating the presence presence of atleast one positive feasible solution for epsilon for every choice for a value of m (and thus App, and the concentration of the other variables).

Thus we have shown how enzyme biphasic response is guarenteed to exist in pYpTErk, for some total concentration of Erk and Kinase in the system, when the above condition is satisfied.

This is not a necessary condition though, biphasic responses can/might exist when the above condition is not satisfied. However this condition represents ONE possible sufficiency condition for obtaining the biphasic dose response in the substrate.

Note: We illustrate the validity of this argument by showing the method predicting the presence of a biphasic response for the arbitraty value of 1 for m (i.e. App = $\frac{3}{\epsilon}$) and some abitrary choice of kinetics that satisfies *Condition*.

$m := 3 :$

$k_1 := 1 : k_2 := 1 : k_4 := 1 : p_2 := 0.1 : p_3 := 1 : p_4 := 1 :$
$k_{b1} := 1 : k_{b2} := 1 : k_{b4} := 1 : p_{b2} := 1 : p_{b3} := 1 : p_{b4} := 1 : k_{ub1} := 1 : k_{ub2} := 1 : k_{ub4} := 1 : p_{b2} := 1 : p_{b3} := 1 : p_{b4} := 1 : p_{ub2} := 1 : p_{ub3} := 1 : p_{ub4} := 1 :$
$$c_1 := \frac{k_{b1}}{k_1+k_{ub1}} : c_2 := \frac{k_{b2}}{k_2+k_{ub2}} : c_4 := \frac{k_{b4}}{k_4+k_{ub4}} : d_3 := \frac{p_{b3}}{p_3+p_{ub3}} : d_2 := \frac{p_{b2}}{p_2+p_{ub2}} : d_4 := \frac{p_{b4}}{p_4+p_{ub4}} :$$
$P_{Total} := 1 :$

$Condition = -0.2500000000 < 0$

$\epsilon := evalf(solve(T, useassumptions)$ assuming $\epsilon > 0) = 4.551992988$
$App = 0.6590519818$
$ErkCon = Erk_{Total} - 2.771482748$
$KCon = K_{Total} - 4.159218072$

The computational evidence of the biphasic dose response behavior predicted above is shown in figure 4.
