# Supplementary material for: A unified approach to dissecting biphasic responses in cell signaling
Source: eLife. 2023 Dec 6;12:e86520. doi: 10.7554/eLife.86520 (PMC10928513; doi:10.7554/eLife.86520)
Supplement: Supplementary file 2. [file elife-86520-supp2.pdf]

Semi-analytical and numerical approaches to discerning biphasic behavior

Of the basic building block systems we have studied, there are five where biphasic behavior is seen along total amounts (either substrate or a total amounts of a particular enzyme).

These are

1. Couple covalent modification system (common kinase common phosphatase) - Substrate biphasic
2. Two tier cascaded system (common phosphatase) - Substrate biphasic
3. Double site phosphorylation (common kinase and separate phosphatase) - Substrate biphasic
4. Double site phosphorylation (Separate kinase and common phosphatase) - Enzyme biphasic (kinase 2)
5. Double site phosphorylation (common kinase and common phosphatase) - Substrate and enzyme biphasic

In this document, by starting with full mathematical description of their behavior, we analyze the steady state behavior of the system.
More specifically, we start with the conservation conditions, and an expression T, which is the mathematical equation which when satisfied denotes a point on the steady state solution space of the system, where the biphasic peak exists. The conservation conditions and the expression T, as seen earlier are obtained in our analytical study within other parts of this Maple document.

The systematic reduction of the variables is not shown here, and we begin straight away with the reduced version of these equations which are essentially a function of the kinetics, steady state concentrations of a few key variables and total amounts.

In summary, for the systems of concern, we then proceed as follows

1. Analytical exploration / insight: where analytical exploration is feasible given the mathematical complexity of the system description, we try to discern the "favorable region" where the behavior can be obtained - we show what this means for the system and general biophysics of the model.

2. Semi-analytical work: We show by way of illustration how partial awareness of the parameters from an experimental context, or otherwise, can allow for the readers to decipher how and where they might find the behavior of interest across other regions of parameter space.

3. Numerical analysis (reasonable ranges): Driven by experimentally observed parameters (sourced from Witzel et al;, 2018) we show how the reasonable ranges seen and used in literature can readily present the behavior.

In the following sections, the key insights and advances along each of the four strands described above are summarized model by model.

Note that there is another system involving the simple covalent modification cycle with an additional interaction presenting biphasic behavior with total amounts (as shown in the main text). However in this

document where our aim is to show the potential of semi-analytical and numerical approaches in allowing additional exploration and to draw insights pertinent to biphasic behavior, we don't explore that system.

Coupled covalent modification system (common kinase and common phosphatase) - substrate biphasic (substrate A)

$restart : with(plots) :$

The three conservation conditions after solving all the differential equations are

$$ACon := \frac{c_2\, Ap\, P_{Total}\, k_2}{(Ap\, c_2 + Bp\, d_2 + 1)\, K\, k_1\, c_1} + Ap + \frac{c_2\, Ap\, P_{Total}}{Ap\, c_2 + Bp\, d_2 + 1} + \frac{c_2\, Ap\, P_{Total}\, k_2}{(Ap\, c_2 + Bp\, d_2 + 1)\, k_1} - A_{Total} :$$

$$KCon := K + \frac{c_2\, Ap\, P_{Total}\, k_2}{(Ap\, c_2 + Bp\, d_2 + 1)\, k_1} + \frac{d_2\, Bp\, P_{Total}\, p_2}{(Ap\, c_2 + Bp\, d_2 + 1)\, p_1} - K_{Total} :$$

$$BCon := \frac{d_2\, Bp\, P_{Total}\, p_2}{(Ap\, c_2 + Bp\, d_2 + 1)\, K\, p_1\, d_1} + Bp + \frac{d_2\, Bp\, P_{Total}}{Ap\, c_2 + Bp\, d_2 + 1} + \frac{d_2\, Bp\, P_{Total}\, p_2}{(Ap\, c_2 + Bp\, d_2 + 1)\, p_1} - B_{Total} :$$

The condition to obtain a biphasic response, $\frac{\mathrm{d}A_p}{\mathrm{d}\, A_{Total}} = 0$ is

$$T := \left(\left(Bp^2\, d_2^{\,2} + (Ap\, c_2 + 1)\, (P_{Total} + 2\, Bp)\, d_2 + (Ap\, c_2 + 1)^2\right)\, (Ap\, c_2 + Bp\, d_2 + 1)\, K^2\, d_1\, p_1^{\,2} + K\, p_2\, P_{Total}\, d_2\, (Ap\, c_2 + 1)\, (Ap\, c_2 + Bp\, d_2 + 1)\, (K\, d_1 + 1)\, p_1 + p_2^{\,2}\, P_{Total}^{\;2}\, Bp\, d_2^{\,2}\, (Ap\, c_2 + 1)\right) k_1 - Ap\, Bp\, c_2\, d_2^{\,2}\, k_2\, p_1\, p_2\, P_{Total}^{\;2} :$$

Note that upto this point no assumption has been made about the system or the behavior

Firstly, we confirm the analytical condition to guarantee the existence of the behavior. To do so, note that the above equation is a second order polynomial in PTotal, and that for realistic (positive concentrations and kinetic parameters) values of parameters, the coefficients of all but the second exponent of PTotal are positive (as shown below)

$collect(T, P_{Total})$

$$\left(p_2^{\,2}\, Bp\, d_2^{\,2}\, (Ap\, c_2 + 1)\, k_1 - Ap\, Bp\, c_2\, d_2^{\,2}\, k_2\, p_1\, p_2\right) P_{Total}^{\;2} + \left((Ap\, c_2 + 1)\, d_2\, (Ap\, c_2 + Bp\, d_2 + 1)\, K^2\, d_1\, p_1^{\,2} + K\, p_2\, d_2\, (Ap\, c_2 + 1)\, (Ap\, c_2 + Bp\, d_2 + 1)\, (K\, d_1 + 1)\, p_1\right) k_1\, P_{Total} + \left(Bp^2\, d_2^{\,2} + 2\, (Ap\, c_2 + 1)\, Bp\, d_2 + (Ap\, c_2 + 1)^2\right)\, (Ap\, c_2 + Bp\, d_2 + 1)\, K^2\, d_1\, p_1^{\,2}\, k_1 \qquad \textbf{(1.1)}$$

However, should the coefficient of the second exponent of the polynomial be negative, there exists real and positive solutions to the expression T (i.e., some value of Ap and Bp irrespective of the kinetic

parameters and total amounts otherwise); This follows from the Descartes rule of signs; since the number of sign changes is 1 in the case of the coefficient being negative, guaranteeing one positive real root for the the second degree polynomial in PTotal. This in turn can be used to estimate (from the conservation equations) what values of total amounts the behavior could be seen at.

Thus, we explore this required and sufficient condition by isolating the coefficient of the second exponent.

$$simplify\left(coeff\left(T, P_{Total}, 2\right) < 0\right)$$

$$p_2\, Bp\, d_2^{\,2}\left(p_2\left(Ap\, c_2 + 1\right) k_1 - Ap\, c_2\, k_2\, p_1\right) < 0 \tag{1.2}$$

$$simplify\left(p_2\left(Ap\, c_2 + 1\right) k_1 - Ap\, c_2\, k_2\, p_1 < 0\right)$$

$$Ap\left(p_2\, k_1 - k_2\, p_1\right) c_2 + p_2\, k_1 < 0 \tag{1.3}$$

Thus if $\left(p_2\, k_1 - k_2\, p_1\right)$ is negative there can be a Ap where the coefficient of the second exponent of PTotal (in expression T) can have a real and positive solution for PTotal. This thus becomes our sufficient and necessary condition.

Insights

1. Condition can be interpreted as a difference between the effective ratio of phosphorylation to dephosphorylation in the first cycle vs second covalent cycle (substrate A vs substrate B)

Thus when the second cycle phosphorylates faster than cycle 1 (Bp formation is faster than Ap formation) then Ap will have a biphasic response with increasing ATotal. By a simple change of variables it can be established that this behavior is also true for Bp showing biphasic response with increasing BTotal.

2. There exists a minimum Ap below which there can be no biphasic response

$$Ap := simplify\left(solve\left(coeff\left(T, P_{Total}, 2\right), Ap\right)\right)$$

$$Ap := -\frac{p_2\, k_1}{c_2\left(p_2\, k_1 - k_2\, p_1\right)} \tag{1.1.1}$$

If we let the ratio of phosphorylation discussed above to be alpha and beta for the two cycles we get

$$k_1 := \text{alpha}\cdot k_2 : p_1 := \text{beta}\cdot p_2 :$$

$$simplify(Ap) = \frac{\alpha}{c_2\left(-\alpha + \beta\right)}$$

Thus this implies that the stronger the rate of phosphorylation of the second cycle the lower the (peak) concentration that Ap can take in the first cycle (irrespective of ATotal)

This can be used as follows - suppose we want to reduce the maximum expression of a modified substrate - it is as simple as injecting another substrate that gets phosphorylated faster (relatively). This caps the maximum expression of Ap possible, while also ensuring biphasic behavior.

Reasonable parameters

In this sub-section, we use kinetics reported by Witzel et al., 2018 for systems involving similar phosphorylation cycles (with some variation within reasonable ranges). The kinetic parameters have units. binding constants are in the units $\mu M^{-1} s^{-1}$, while binding and catalytic constants are in the units s^{-1}. The variables and the total amounts which denote concentrations are in the units μM.

$$k_{b1} := 0.18 : k_{b2} := 0.18 : p_{b1} := 0.18 : p_{b2} := 0.18 :$$
$$k_{ub1} := 0.27 : k_{ub2} := 0.27 : p_{ub1} := 0.27 : p_{ub2} := 0.27 :$$
$$k_1 := 0.0147 : k_2 := 0.107 : p_1 := 0.0385 : p_2 := 0.0515 :$$
$$c_1 := \frac{k_{b1}}{k_1 + k_{ub1}} : c_2 := \frac{k_{b2}}{k_2 + k_{ub2}} : d_1 := \frac{p_{b1}}{p_1 + p_{ub1}} : d_2 := \frac{p_{b2}}{p_2 + p_{ub2}} :$$

We then assume that the total PTotal in the system is 5, and that we'd want to observe the biphasic response when the concentration of free kinase in the system is 1.

$$P_{Total} := 5 : K := 1 :$$

With these parameters inputted, the expression T (which contained the necessary and sufficient condition to observe biphasic response) reduces to a bivariate expression (in Ap and Bp). We can solve this to estimate the concentrations of Ap and Bp where the curve has solutions.

This is shown below

$$Q := contourplot((T), Ap = 0.1 ..2, Bp = 0.1 ..15, contours = [0])$$

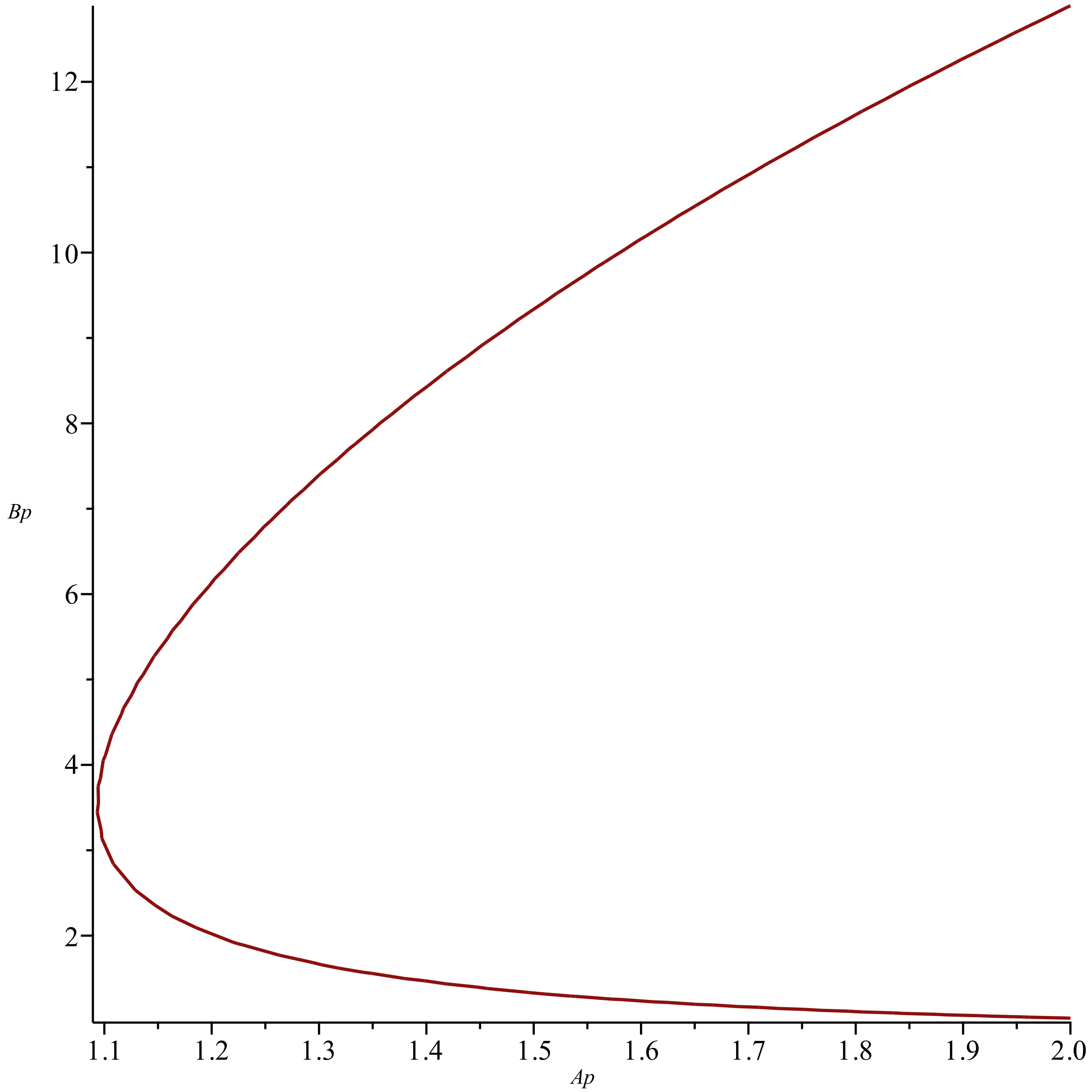

For each concentration Ap and Bp along this curve, the system admits to biphasic response for the concentrations of K and PTotal required (1 and 5 respectively), and the kinetic parameters enforced.

We can further explore the concentration of ATotal, KTotal and BTotal for each of these points along the curve. This is shown below for one sample point to reinforce the point that the behavior is accessible for reasonable ranges of total amounts.

```
dt := plottools:-getdata(Q) :
L := ListTools:-Flatten(select~(type, [dt], Matrix)) :
M := ⟨op(L)⟩ :

Qdata := Matrix(ListTools:-MakeUnique(convert(M, listlist))) :
```

$Ap\_values := Qdata[1..,1] : Bp\_values := Qdata[1..,2] :$

We pick a value from the contour plot now to show that the resulting total amounts behind these contours (which denote biphasic peaks)

$Ap\_values[30] = 1.15881350154579$
$Bp\_values[30] = 5.48034653621288$

$A_{Total} := solve(eval(ACon, \{Ap = Ap\_values[30], Bp = Bp\_values[30]\})) = 13.00576818$
$K_{Total} := solve(eval(KCon, \{Ap = Ap\_values[30], Bp = Bp\_values[30]\})) = 9.797444261$
$B_{Total} := solve(eval(BCon, \{Ap = Ap\_values[30], Bp = Bp\_values[30]\})) = 20.85070491$

Two tier cascaded system (common phosphatase) - substrate biphasic (substrate B)

$restart : with(plots) :$

The three conservation conditions after solving all the differential equations are

$$ACon := \frac{c_2\, Ap\, P_{Total}\, k_2}{(Ap\, c_2 + Bp\, d_2 + 1)\, K\, k_1\, c_1} + Ap + \frac{c_2\, Ap\, P_{Total}}{Ap\, c_2 + Bp\, d_2 + 1} + \frac{c_2\, Ap\, P_{Total}\, k_2}{(Ap\, c_2 + Bp\, d_2 + 1)\, k_1} + \frac{d_2\, Bp\, P_{Total}\, p_2}{(Ap\, c_2 + Bp\, d_2 + 1)\, p_1} - A_{Total} :$$

$$BCon := \frac{d_2\, Bp\, P_{Total}\, p_2}{(Ap\, c_2 + Bp\, d_2 + 1)\, Ap\, p_1\, d_1} + \frac{d_2\, Bp\, P_{Total}}{Ap\, c_2 + Bp\, d_2 + 1} + \frac{d_2\, Bp\, P_{Total}\, p_2}{(Ap\, c_2 + Bp\, d_2 + 1)\, p_1} + Bp - B_{Total} :$$

$$KCon := K + \frac{c_2\, Ap\, P_{Total}\, k_2}{(Ap\, c_2 + Bp\, d_2 + 1)\, k_1} - K_{Total} :$$

The condition to obtain a biphasic response, $\frac{dB_p}{d\,B_{Total}} = 0$ is

$$T := -c_1 K^2 \left(\left(-Ap^2 c_2^2 - (Bp\, d_2 + 1)\, (P_{Total} + 2\,Ap)\, c_2 - (Bp\, d_2 + 1)^2\right) p_1 + d_2\, Bp\, P_{Total}\, p_2\, c_2\right) (Ap\, c_2 + Bp\, d_2 + 1)\, k_1^2 + K\, P_{Total}\, c_2\, k_2\, p_1\, (Bp\, d_2 + 1)\, (Ap\, c_2 + Bp\, d_2 + 1)\, (K\, c_1 + 1)\, k_1 + c_2^2\, Ap\, P_{Total}^2\, k_2^2\, (Bp\, d_2 + 1)\, p_1 :$$

Note that upto this point no assumption has been made about the system or the behavior

Firstly, we confirm the analytical condition to guarantee the existence of the behavior. To do so, note that

the above equation is a second order polynomial in K, and that for realistic (positive concentrations and kinetic parameters) values of parameters, the coefficients of all but the second exponent of K are positive.

However, should the coefficient of the second exponent of the polynomial be negative, there exists real and positive solutions to the expression T (i.e., some value of Ap and Bp irrespective of the kinetic parameters and total amounts otherwise); This in turn can be used to estimate (from the conservation equations) what values of total amounts the behavior could be seen at.

Thus, we explore this required and sufficient condition by isolating the coefficient of the second exponent.

$simplify(coeff(T, K, 2)) =$

$$\left(\left(\left(Ap^2 c_2^2 + 2\left(\frac{P_{Total}}{2} + Ap\right)(Bp\, d_2 + 1)\, c_2 + (Bp\, d_2 + 1)^2\right) p_1 - Bp\, d_2\, P_{Total}\, p_2\, c_2\right) k_1 + P_{Total}\, c_2\, k_2\, p_1\, (Bp\, d_2 + 1)\right)(Ap\, c_2 + Bp\, d_2 + 1)\, k_1\, c_1$$

This factorizes well into two terms. The first term (which is $\left(\left(Ap^2 c_2^2 + 2\left(\frac{P_{Total}}{2} + Ap\right)(Bp\, d_2 + 1)\, c_2 + (Bp\, d_2 + 1)^2\right) p_1 - Bp\, d_2\, P_{Total}\, p_2\, c_2\right) k_1 + P_{Total}\, c_2\, k_2\, p_1\, (Bp\, d_2 + 1)$) can be negative positive or zero, while the second term (which is $(Ap\, c_2 + Bp\, d_2 + 1)$) is strictly positive for all feasible values of kinetic parameters and total amounts. We isolate the first term now, and observe it as a polynomial in PTotal (we do this since this term dictates whether the overall expression, the coefficient of the second exponent of K in T is negative or not.

$$collect\left(\left(\left(Ap^2 c_2^2 + 2\left(\frac{P_{Total}}{2} + Ap\right)(Bp\, d_2 + 1)\, c_2 + (Bp\, d_2 + 1)^2\right) p_1 - Bp\, d_2\, P_{Total}\, p_2\, c_2\right) k_1 + P_{Total}\, c_2\, k_2\, p_1\, (Bp\, d_2 + 1), P_{Total}\right)$$

$$\left(\left(2\left(\frac{Bp\, d_2}{2} + \frac{1}{2}\right) c_2\, p_1 - Bp\, d_2\, p_2\, c_2\right) k_1 + c_2\, k_2\, p_1\, (Bp\, d_2 + 1)\right) P_{Total} + \left(Ap^2 c_2^2 + 2\, Ap\, (Bp\, d_2 + 1)\, c_2 + (Bp\, d_2 + 1)^2\right) p_1\, k_1 \quad \textbf{(2.1)}$$

Here again, we notice that the term independent of PTotal, is always positive for all feasible values of kinetic parameters and total amounts, while the coefficient of PTotal, can be positive or negative depending on parameters and variable concentrations. Hence if the coefficient of PTotal is negative, for a sufficiently large PTotal, the overall expression is negative - implying the coefficient of the second exponent of K in T is negative.

$$collect\left(\left(2\left(\frac{Bp\, d_2}{2} + \frac{1}{2}\right) c_2\, p_1 - Bp\, d_2\, p_2\, c_2\right) k_1 + c_2\, k_2\, p_1\, (Bp\, d_2 + 1), Bp\right)$$

$$\left((d_2\, c_2\, p_1 - d_2\, c_2\, p_2)\, k_1 + c_2\, k_2\, p_1\, d_2\right) Bp + c_2\, p_1\, k_1 + c_2\, k_2\, p_1 \quad \textbf{(2.2)}$$

We notice here again that the constant term independent of Bp is always positive, while the coefficient of Bp can be negative / positive / zero. Thus similar to the argument above, if the coefficient of Bp is negative,

then for a sufficiently large Bp, the overall term (including the constant) is negative, and for a sufficiently large PTotal, the coefficient of the second exponent of K in T is negative guaranteeing a feasible root for T in K, i.e., biphasic behavior exists.

$$simplify\left(\left(d_2 c_2 p_1 - d_2 c_2 p_2\right) k_1 + c_2 k_2 p_1 d_2\right)$$

$$c_2 \left(k_1 \left(p_1 - p_2\right) + k_2 p_1\right) d_2 \tag{2.3}$$

Thus if $\left(k_1 \left(p_1 - p_2\right) + k_2 p_1\right)$ is negative there can be a sufficiently large Bp and sufficiently large PTotal where the behavior can be guaranteed

Insights

1. Condition can be interpreted as a difference between the effective ratio of phosphorylation in the first tier vs ratio of phosphorylation in the second tier (substrate A vs substrate B). When this difference is greater than 1, i.e., when the second tier substrate phosphorylates faster than the first tier substrate (Bp formation is faster than Ap formation) then Bp will have a biphasic response with increasing BTotal.

2. A higher amount of phosphatase promotes the behavior (or the possibility to be found). This is inferred since, with increasing PTotal, the above second exponent of K, becomes more negative, admitting real and positive solutions satisfying the condition.

Reasonable parameters

In this sub-section, we use kinetics reported by Witzel et al., 2018 for systems involving similar phosphorylation cycles (with some variation within reasonable ranges). The kinetic parameters have units. binding constants are in the units $\mu M^{-1} s^{-1}$, while binding and catalytic constants are in the units s^{-1}. The variables and the total amounts which denote concentrations are in the units μM.

$$k_{b1} := 0.18 : k_{b2} := 0.18 : p_{b1} := 0.18 : p_{b2} := 0.18 :$$
$$k_{ub1} := 0.27 : k_{ub2} := 0.27 : p_{ub1} := 0.27 : p_{ub2} := 0.27 :$$
$$k_1 := 0.1 : k_2 := 0.01 : p_1 := 0.01 : p_2 := 0.1 :$$
$$c_1 := \frac{k_{b1}}{k_1 + k_{ub1}} : c_2 := \frac{k_{b2}}{k_2 + k_{ub2}} : d_1 := \frac{p_{b1}}{p_1 + p_{ub1}} : d_2 := \frac{p_{b2}}{p_2 + p_{ub2}} :$$

We then assume that the total PTotal in the system is 2, and that we'd want to observe the biphasic response when the concentration of free kinase in the system is 0.2.

$$P_{Total} := 2 : K := 0.2 :$$

With these parameters inputted, the expression T (which contained the necessary and sufficient condition to observe biphasic response) reduces to a bivariate expression (in Ap and Bp). We can solve this to estimate the concentrations of Ap and Bp where the curve has solutions.

$$Q := contourplot((T), Ap = 0.1 \,..\, 2, Bp = 0.1 \,..\, 10, contours = [0])$$

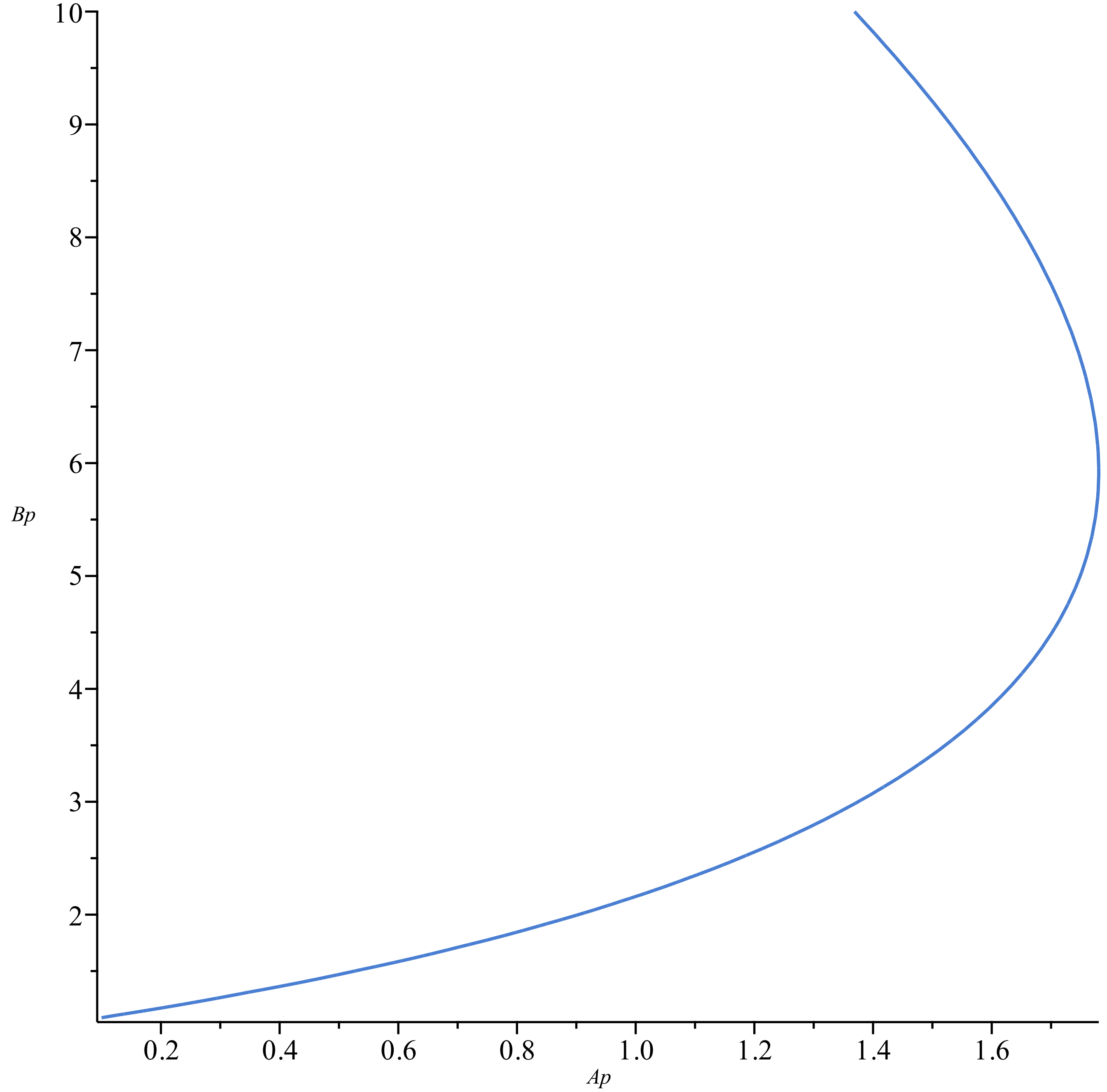

For each concentration Ap and Bp along this curve, the system admits to biphasic response for the concentrations of K and PTotal required (1 and 5 respectively), and the kinetic parameters enforced.

We can further explore the concentration of ATotal, KTotal and BTotal for each of these points along the curve. This is shown below for one sample point to reinforce the point that the behavior is accessible for reasonable ranges of total amounts.

```
dt := plottools:-getdata(Q) :
L := ListTools:-Flatten(select~(type, [dt], Matrix)) :
M := ⟨op(L)⟩ :

Qdata := Matrix(ListTools:-MakeUnique(convert(M, listlist))) :
```

$Ap\_values := Qdata[1..,1] : Bp\_values := Qdata[1..,2] :$

We pick a value from the contour plot now to show that the resulting total amounts behind these contours (which denote biphasic peaks)

$Ap\_values[30] = 0.487755102040817$
$Bp\_values[30] = 1.45618320775018$
$A_{Total} := solve(eval(ACon, \{Ap = Ap\_values[30], Bp = Bp\_values[30]\})) = 8.154844782$
$K_{Total} := solve(eval(KCon, \{Ap = Ap\_values[30], Bp = Bp\_values[30]\})) = 0.2310149809$
$B_{Total} := solve(eval(BCon, \{Ap = Ap\_values[30], Bp = Bp\_values[30]\})) = 31.51139339$

DSP (common kinase and sep phosphatase) - substrate biphasic

$restart : with(plots) :$

The conservation conditions describing the steady state of the system (after solving all the differential equations) are

$$ACon := \frac{P1_{Total}\, k_4\, c_4\, App\, P2_{Total}\, k_3\, c_3}{\left(\frac{App\, P2_{Total}\, c_3\, c_4\, k_3}{App\, c_3 + 1} + K\, k_2\, c_2\right) K \left(App\, c_3 + 1\right) k_1\, c_1} + \frac{App\, P2_{Total}\, k_3\, c_3}{\left(App\, c_3 + 1\right) K\, k_2\, c_2} + App$$
$$+ \frac{P1_{Total}\, k_4\, c_4\, App\, P2_{Total}\, k_3\, c_3}{\left(\frac{App\, P2_{Total}\, c_3\, c_4\, k_3}{App\, c_3 + 1} + K\, k_2\, c_2\right) \left(App\, c_3 + 1\right) k_1} + \frac{App\, P2_{Total}\, k_3\, c_3}{\left(App\, c_3 + 1\right) k_2} + \frac{c_3\, App\, P2_{Total}}{App\, c_3 + 1}$$
$$+ \frac{c_4\, App\, P2_{Total}\, k_3\, c_3\, P1_{Total}}{\left(App\, c_3 + 1\right) \left(\frac{App\, P2_{Total}\, c_3\, c_4\, k_3}{App\, c_3 + 1} + K\, k_2\, c_2\right)} - A_{Total} :$$

$$KCon := K + \frac{P1_{Total}\, k_4\, c_4\, App\, P2_{Total}\, k_3\, c_3}{\left(\frac{App\, P2_{Total}\, c_3\, c_4\, k_3}{App\, c_3 + 1} + K\, k_2\, c_2\right) \left(App\, c_3 + 1\right) k_1} + \frac{App\, P2_{Total}\, k_3\, c_3}{\left(App\, c_3 + 1\right) k_2} - K_{Total} :$$

The condition to obtain a biphasic response, $\frac{\mathrm{d}A_{pp}}{\mathrm{d}\,A_{Total}} = 0$ is

$$T := \frac{1}{\left(App\, P2_{Total}\, c_3\, c_4\, k_3 + K\, k_2\, c_2\, \left(App\, c_3 + 1\right)\right)^2 k_1} \left(K^2\, k_1\, k_2^2\, \left(App\, c_3 + 1\right)^2 c_2^2 + 2\, App\, \left(App\, c_3\right.\right.$$
$$\left.\left. + 1\right) P2_{Total} \left(K\, k_1 - \frac{P1_{Total}\, k_4}{2}\right) k_2\, c_4\, k_3\, c_3\, c_2 + App^2\, c_3^2\, c_4^2\, k_1\, k_3^2\, P2_{Total}^2\right) :$$

In order to explore this system better, we simplify the equations further, notably by solving expression T for P1Total, the conservation condition KCon for P2Total, and finally the conservation equation ACon for App as shown below.

$P1_{Total} := simplify(solve(T, P1_{Total}))$

$$P1_{Total} := \frac{(App\ (K\, k_2\, c_2 + c_4\, k_3\, P2_{Total})\ c_3 + K\, k_2\, c_2)^2\ k_1}{App\ (App\ c_3 + 1)\ P2_{Total}\, k_4\, k_2\, c_4\, k_3\, c_3\, c_2} \quad \textbf{(3.1)}$$

$P2_{Total} := simplify(solve(KCon, P2_{Total}))$

$$P2_{Total} := -\frac{2\, c_2 \left(K - \frac{K_{Total}}{2}\right) (App\ c_3 + 1)\ k_2}{App\ c_3\, k_3\, (c_2 + c_4)} \quad \textbf{(3.2)}$$

$App := simplify(solve(ACon, App)) =$

$$\frac{1}{K\, c_1\, k_3\, k_4\, (c_2 + c_4)} \Big(\big(-((-c_2 - c_4)\ k_4 + k_1\ (c_2 - c_4))\ c_1\, K^2 + ((((-K_{Total} + A_{Total})\ c_2 + 2 + (-K_{Total} + A_{Total})\ c_4)\ k_4 - K_{Total}\, c_4\, k_1)\ c_1 - k_4\ (c_2 - c_4))\ K - K_{Total}\, k_4\ (c_1 + c_4) \big)\ k_3 + 2\, c_2 \left(K - \frac{1}{2}\, K_{Total}\right) c_1\, k_4\, k_2\, K \Big)$$

Any feasible solutions for App (using feasible positive kinetic parameters, and for feasible positive concentrations of K) will allow us to estimate the total amounts of phosphatases required to obtain biphasic response.

For a given set of kinetic parameters, this way, it is easier to understand ranges of App, and K (and by extension total amounts) that will enable the behavior. For example, if the following parameters and total amounts of Kinase and substrate are valid for the system,

$$k_2 := 1 : k_1 := 1 : k_3 := 1 : k_4 := 1 : c_1 := \frac{1}{k_1} : c_2 := \frac{1}{k_2} : c_3 := \frac{1}{k_3} : c_4 := \frac{1}{k_4} : K_{Total} := 1 :$$
$$A_{Total} := 50 :$$

The following plot of App vs K denotes all feasible concentrations of App and free kinase K were the biphasic behavior is to obtained.

$$Q := plot\left(App, K = 0 .. \frac{K_{Total}}{2}\right)$$

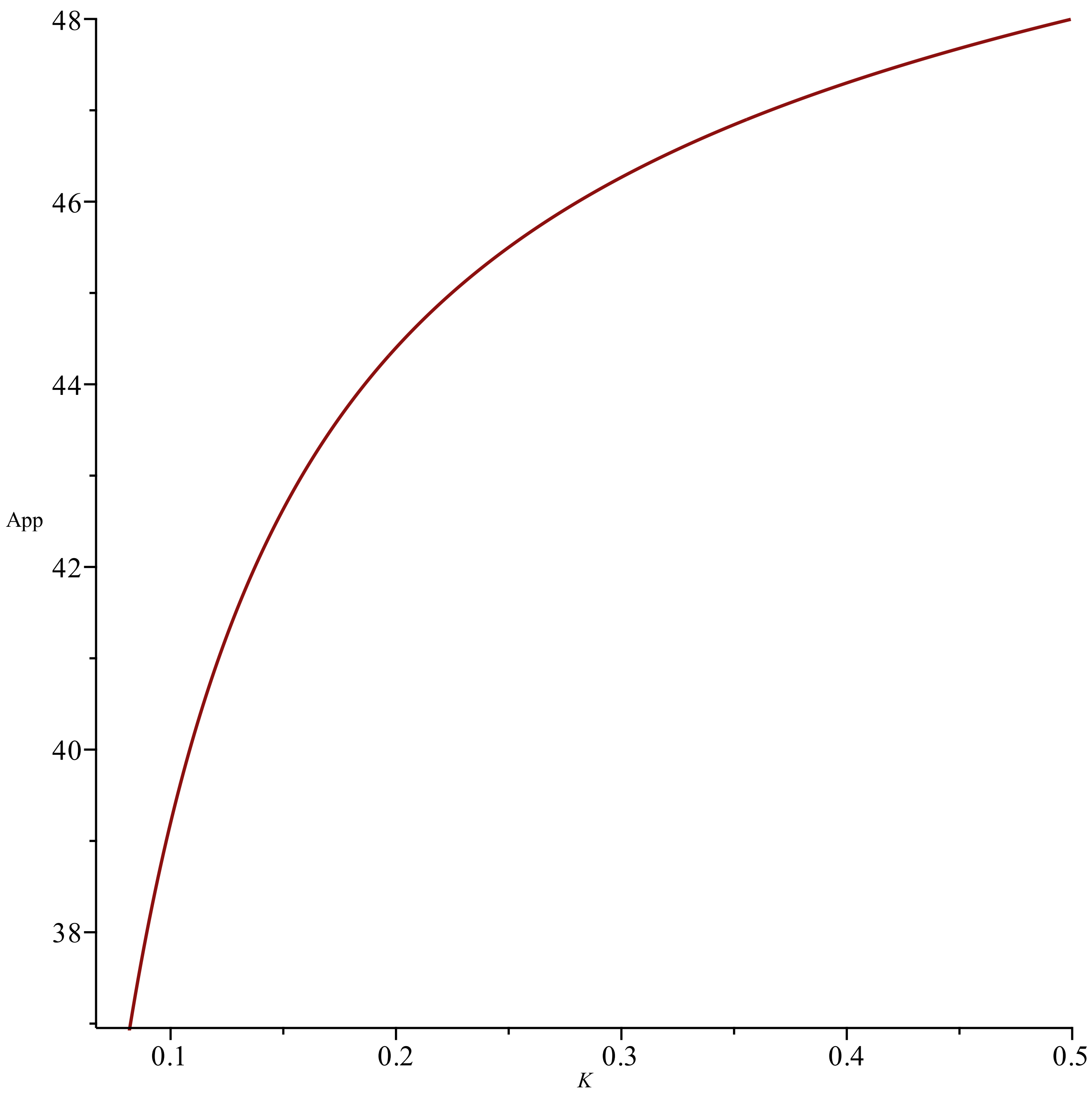

For each concentration App and K along this curve, the system admits to biphasic response

We can further explore the concentration of P1Total, P2Total for each of these points along the curve. This is shown below for one sample point to reinforce the point that the behavior is accessible for reasonable ranges of total amounts.

$dt \coloneqq plottools\text{:-}getdata(Q):$
$L \coloneqq ListTools\text{:-}Flatten(select\sim(type, [dt], Matrix)):$
$M \coloneqq \langle op(L) \rangle:$

$Qdata \coloneqq Matrix(ListTools\text{:-}MakeUnique(convert(M, listlist))):$

$K\_values := Qdata[1..,1] : App\_values := Qdata[1..,2] :$

We pick a value from the contour plot now to show that the resulting total amounts behind these contours (which denote biphasic peaks)

$K\_values[20] = 0.0256250000000000$
$App\_values[20] = 10.0268597500000$

$eval\left(P1_{Total}, \{App = App\_values[30], K = K\_values[30]\}\right) = 0.541271989185909$
$eval\left(P2_{Total}, \{App = App\_values[30], K = K\_values[30]\}\right) = 0.482091230500726$

(3.3)

Reasonable parameters

In this sub-section, we use kinetics reported by Witzel et al., 2018 for systems involving similar phosphorylation cycles (with some variation within reasonable ranges). The kinetic parameters have units. binding constants are in the units $\mu M^{-1} s^{-1}$, while binding and catalytic constants are in the units s^{-1}. The variables and the total amounts which denote concentrations are in the units μM.

$k_{b1} := 0.18 : k_{b2} := 0.18 : k_{b3} := 0.18 : k_{b4} := 0.18 :$
$k_{ub1} := 0.27 : k_{ub2} := 0.27 : k_{ub3} := 0.27 : k_{ub4} := 0.27 :$
$k_1 := 0.0747 : k_2 := 0.0357 : k_3 := 0.0585 : k_4 := 0.0315 :$

$$c_1 := \frac{k_{b1}}{k_1 + k_{ub1}} : c_2 := \frac{k_{b2}}{k_2 + k_{ub2}} : c_3 := \frac{k_{b3}}{k_3 + k_{ub3}} : c_4 := \frac{k_{b4}}{k_4 + k_{ub4}} :$$

We then assume that the total P1Total in the system is 5, and total P2Total in the system is 1

$P1_{Total} := 5 : P2_{Total} := 1 :$

With these parameters inputted, the expression T (which contained the necessary and sufficient condition to observe biphasic response) reduces to a bivariate expression (in K and App). We can solve this to estimate the concentrations of Ap and Bp where the curve has solutions.

$Q := contourplot((T), App = 0.1..5, K = 0.1..1, contours = [0])$

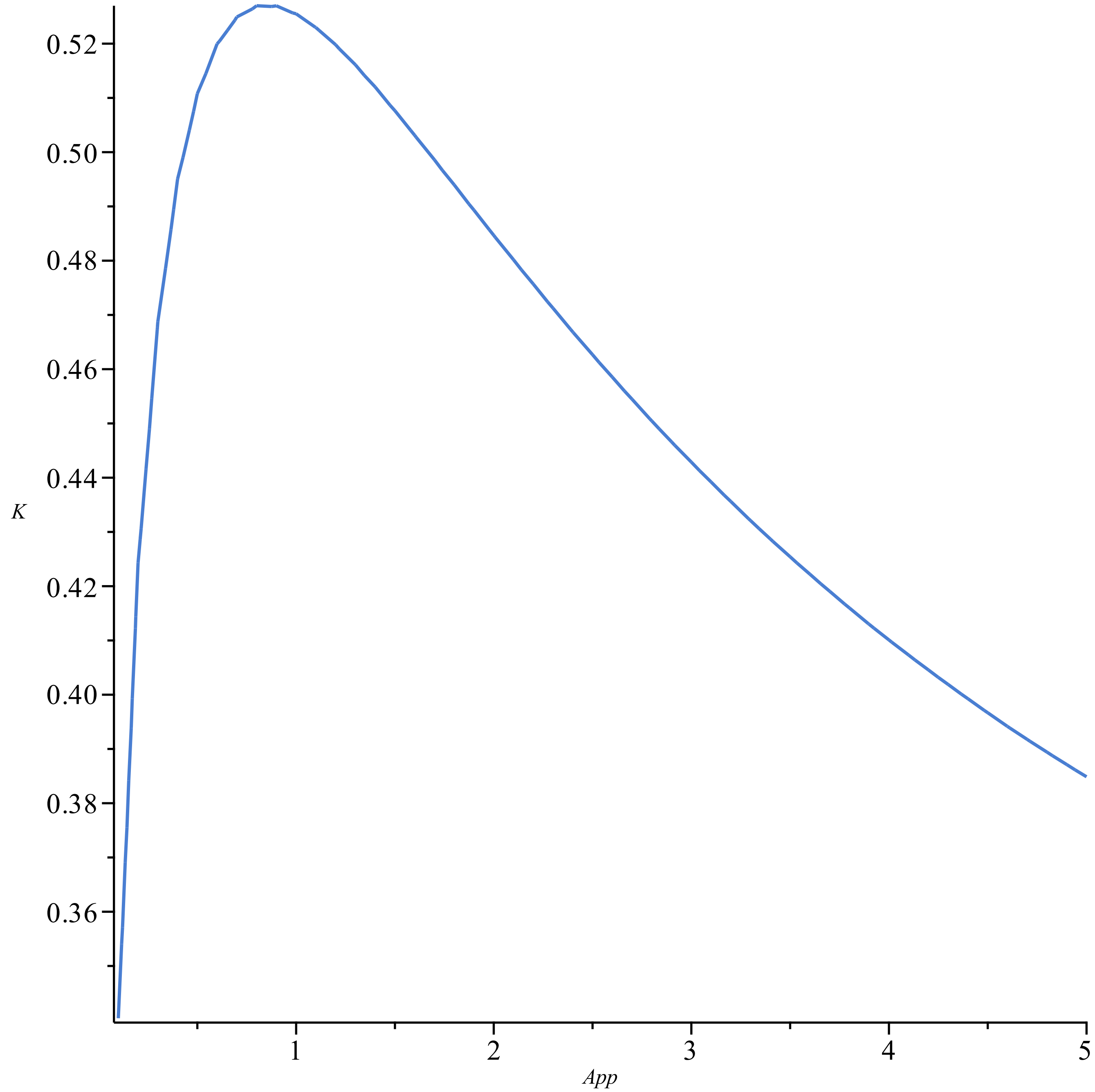

For each concentration K and App along this curve, the system admits to biphasic response for the concentrations of ATotal and KTotal required, and the kinetic parameters enforced.

We can further explore the concentration of ATotal and KTotal for each of these points along the curve. This is shown below for one sample point to reinforce the point that the behavior is accessible for reasonable ranges of total amounts.

$dt := plottools\text{:-}getdata(Q)$:
$L := ListTools\text{:-}Flatten(select\sim(type, [dt], Matrix))$:
$M := \langle op(L) \rangle$:

$Qdata := Matrix(ListTools\text{:-}MakeUnique(convert(M, listlist)))$:

$App\_values := Qdata[1..,1] : epsilon\_values := Qdata[1..,2] :$

We pick a value from the contour plot now to show that the resulting total amounts behind these contours (which denote biphasic peaks)

$App\_values[100] = 1.96846935938081$
$epsilon\_values[100] = 0.486122448979592$

$A_{Total} := solve(eval(ACon, \{App = App\_values[100], K = epsilon\_values[100]\})) = 16.16504184$
$K_{Total} := solve(eval(KCon, \{App = App\_values[100], K = epsilon\_values[100]\})) = 2.684676393$

DSP (sep kinase and common phosphatase) - enzyme biphasic (enzyme K2)

$restart : with(plots) :$

The conservation conditions describing the steady state of the system (after solving all the differential equations) are

$$ACon := \frac{App\, P_{Total}\, k_3\, k_4\, c_3\, c_4}{\left(c_2\, k_2\, (c_3\, App + 1)\, \epsilon + c_4\, App\, k_3\, c_3\right) c_1\, K1\, k_1} + \frac{App\, k_3\, c_3}{\epsilon\, k_2\, c_2} + App + \frac{App\, P_{Total}\, k_3\, k_4\, c_3\, c_4}{\left(c_2\, k_2\, (c_3\, App + 1)\, \epsilon + c_4\, App\, k_3\, c_3\right) k_1} + \frac{c_2\, App\, k_3\, c_3\, \epsilon\, P_{Total}}{c_2\, k_2\, (c_3\, App + 1)\, \epsilon + c_4\, App\, k_3\, c_3} + \frac{c_3\, App\, P_{Total}\, \epsilon\, k_2\, c_2}{c_2\, k_2\, (c_3\, App + 1)\, \epsilon + c_4\, App\, k_3\, c_3} + \frac{c_4\, App\, k_3\, c_3\, P_{Total}}{c_2\, k_2\, (c_3\, App + 1)\, \epsilon + c_4\, App\, k_3\, c_3} - A_{Total} :$$

$$K1Con := K1 + \frac{App\, P_{Total}\, k_3\, k_4\, c_3\, c_4}{\left(c_2\, k_2\, (c_3\, App + 1)\, \epsilon + c_4\, App\, k_3\, c_3\right) k_1} - K1_{Total} :$$

$$K2Con := \frac{\epsilon^2\, P_{Total}\, k_2\, c_2}{c_2\, k_2\, (App\, c_3 + 1)\, \epsilon + c_4\, App\, k_3\, c_3} + \frac{c_2\, App\, k_3\, c_3\, \epsilon\, P_{Total}}{c_2\, k_2\, (App\, c_3 + 1)\, \epsilon + c_4\, App\, k_3\, c_3} - K2_{Total} :$$

$$T := -\Big(k_2^2\, (App\, c_3 + 1)\, c_2^3\, k_1\, \Big(\big(c_1\, (App^2\, c_3^2 + 2\, App\, c_3 + c_4\, P_{Total} + 1)\, k_1 + k_4\, P_{Total}\, c_4\, (App\, c_3 + 1)\, c_1\big)\, k_2 - P_{Total}\, App\, c_1\, c_3\, c_4\, k_1\, k_3\Big)\, \epsilon^3 + c_3\, c_4\, \Big(\big(c_1\, (3\, App^2\, c_3^2 + 6\, App\, c_3 + c_4\, P_{Total} + 3)\, k_1^2 + k_4\, P_{Total}\, c_4\, (App\, c_3 + 1)\, c_1\, k_1\big)\, k_2 - P_{Total}\, App\, c_1\, c_3\, c_4\, k_1^2\, k_3\Big)\, k_2\, k_3\, c_2^2\, App\, \epsilon^2 + 3\, App^2\, c_1\, c_2\, c_3^2\, c_4^2\, k_1^2\, k_2\, k_3^2\, (App\, c_3 + 1)\, \epsilon + App^3\, c_1\, c_3^3\, c_4^3\, k_1^2\, k_3^3\Big)\, c_3\, k_3\, App\, K1^2 - \Big(k_2^3\, (App\, c_3 + 1)^2\, c_2^3\, k_1\, k_4\, P_{Total}\, c_4\, \epsilon^3 + c_3\, c_4^2\, k_4\, P_{Total}\, (App\, c_3 + 1)\, k_1\, k_2^2\, k_3\, c_2^2\, App\, \epsilon^2\Big)\, c_3\, k_3\, App\, K1 - App^2\, P_{Total}^2\, k_3^2\, k_4^2\, c_3^2\, c_4^2\, c_2^2\, k_2^2\, (App\, c_3 + 1)\, \epsilon^2 :$$

This system presents a challenge to analytically ascertain any insight as to how the parameters might enable biphasic behavior. However, using semi-analytical approaches it is possible to ascertain the role of individual parameters in enabling / not enabling the behavior.

Suppose all but one kinetic parameters are known, and we wish for biphasic behavior to be found for reasonable ranges of App then we can estimate the required kinetics and some total amounts as shown below (assuming some reasonable concentration of K1 and K2 in the process)

$$k_2 := 1 : k_3 := 10 : k_4 := 0.5 : c_1 := \frac{1}{k_1} : c_2 := \frac{1}{k_2} : c_3 := \frac{1}{k_3} : c_4 := \frac{1}{k_4} : P_{Total} := 1 : \text{epsilon} := 100 : App := 100 :$$

It is now possible to solve the conservation equations sequentially for ATotal and K1Total as shown below

$$A_{Total} := solve(ACon, A_{Total})$$

$$A_{Total} := \frac{1}{K1\, k_1}\left(4.000000000 \times 10^{-11}\left(2.740384618 \times 10^{12}\, K1\, k_1 + 1.923076923 \times 10^{9}\, K1 + 1.923076923 \times 10^{9}\, k_1\right)\right) \tag{4.1}$$

$$K1_{Total} := solve(K1Con, K1_{Total})$$

$$K1_{Total} := \frac{4.000000000 \times 10^{-11}\left(2.500000000 \times 10^{10}\, K1\, k_1 + 1.923076923 \times 10^{9}\right)}{k_1} \tag{4.2}$$

This simplifies the expression T as follows,

$$simplify(T) =$$
$$-1.100000000 \times 10^{9} + \left(3.770000000 \times 10^{10}\, k_1 - 1.430000000 \times 10^{10}\right) K1^2 - 1.430000000 \times 10^{10}\, K1\, k_1$$

We can solve this equation to estimate the concentrations of K1 for each value of kinetic parameter k1 where the curve has solutions.

$$Q := contourplot((T), K1 = 0.1\,..2, k_1 = 0.1\,..2, contours = [0])$$

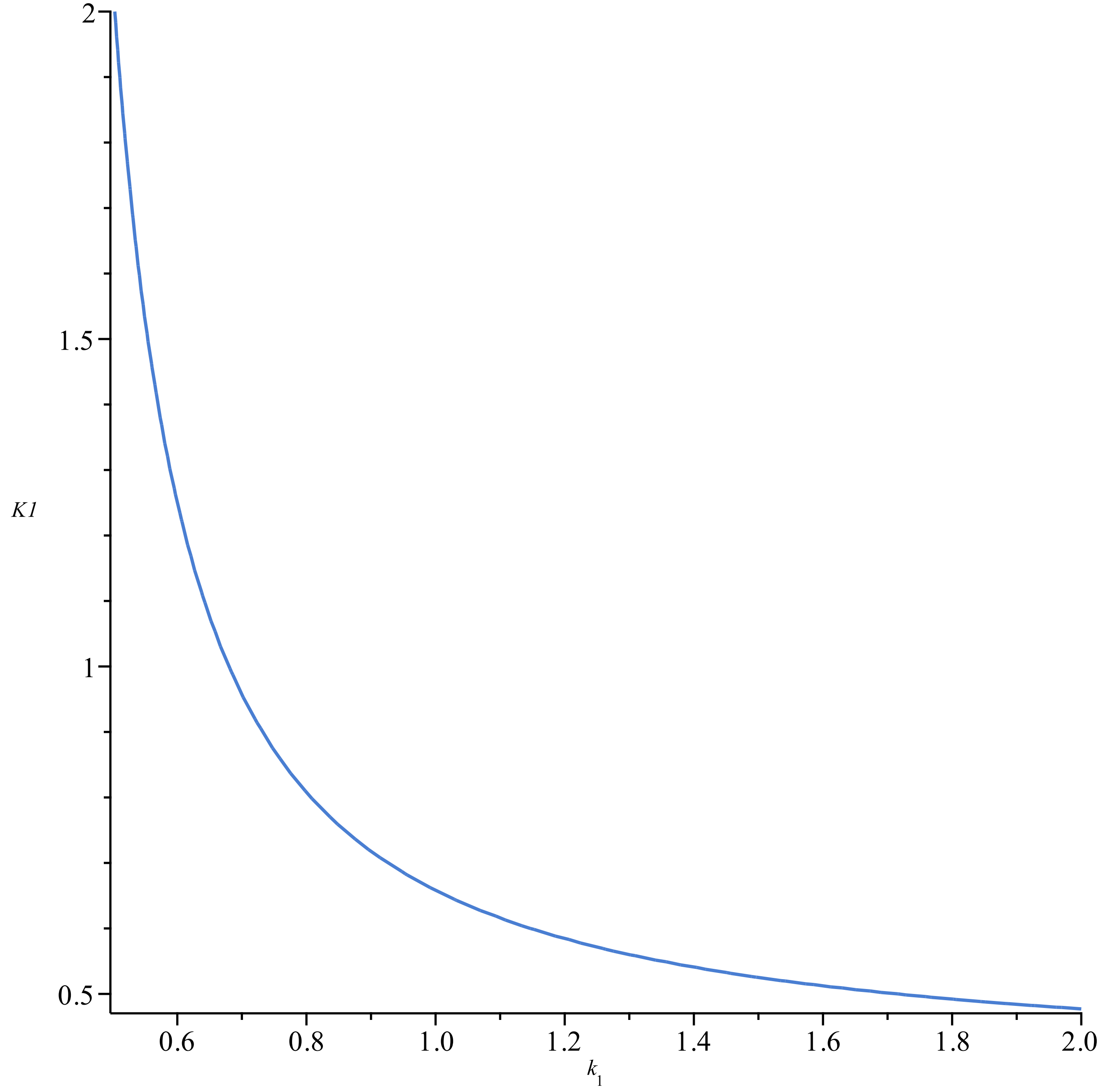

For each concentration K1 and parameter k1 along this curve, the system admits to biphasic response

We can further explore the concentration of ATotal, K1Total for each of these points along the curve. This is shown below for one sample point to reinforce the point that the behavior is accessible for reasonable ranges of total amounts.

$dt := plottools\text{:-}getdata(Q):$
$L := ListTools\text{:-}Flatten(select\sim(type, [dt], Matrix)):$
$M := \langle op(L) \rangle :$

$Qdata := Matrix(ListTools\text{:-}MakeUnique(convert(M, listlist))):$

$K1\_values := Qdata[1..,1] : k1\_values := Qdata[1..,2] :$

We pick a value from the contour plot now to show that the resulting total amounts behind these contours (which denote biphasic peaks)

$K1\_values[30] = 0.531422207331294$
$k1\_values[30] = 1.68490432328095$

$eval\left(K1_{Total}, \{K1 = K1\_values[30], k_1 = k1\_values[30]\}\right) = 0.577076477343618$
$eval\left(A_{Total}, \{K1 = K1\_values[30], k_1 = k1\_values[30]\}\right) = 109.805788448840$

Reasonable parameters

In this sub-section, we use kinetics reported by Witzel et al., 2018 for systems involving similar phosphorylation cycles (with some variation within reasonable ranges). The kinetic parameters have units. binding constants are in the units $\mu M^{-1} s^{-1}$, while binding and catalytic constants are in the units s^{-1}. The variables and the total amounts which denote concentrations are in the units μM.

$k_{b1} := 0.18 : k_{b2} := 0.18 : k_{b3} := 0.18 : k_{b4} := 0.18 :$
$k_{ub1} := 0.27 : k_{ub2} := 0.27 : k_{ub3} := 0.27 : k_{ub4} := 0.27 :$
$k_1 := 0.0747 : k_2 := 0.0357 : k_3 := 0.585 : k_4 := 0.0315 :$
$$c_1 := \frac{k_{b1}}{k_1 + k_{ub1}} : c_2 := \frac{k_{b2}}{k_2 + k_{ub2}} : c_3 := \frac{k_{b3}}{k_3 + k_{ub3}} : c_4 := \frac{k_{b4}}{k_4 + k_{ub4}} :$$

We then assume that the total PTotal in the system is 5, and the concentration of free kinase K1 in the system is 2

$P_{Total} := 5 : K1 := 2 :$

With these parameters inputted, the expression T (which contained the necessary and sufficient condition to observe biphasic response) reduces to a bivariate expression (in epsilon and App). We can solve this to estimate the concentrations of App and epsilon where the curve has solutions.

$Q := contourplot(T, App = 0.1..5, \text{epsilon} = 0.1..5, contours = [0])$

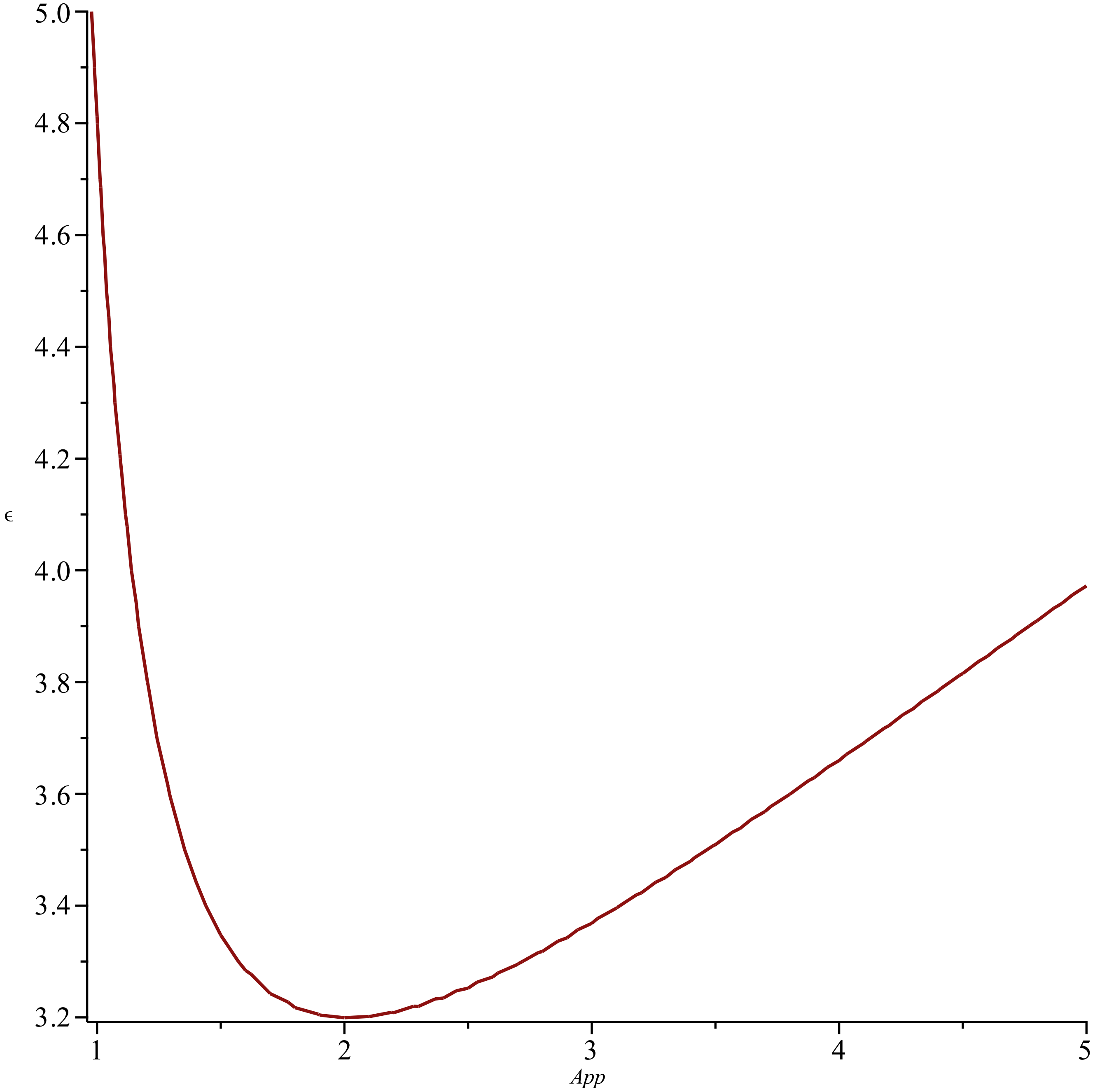

For each concentration epsilon and App along this curve, the system admits to biphasic response for the concentrations of ATotal and K2Total required, and the kinetic parameters enforced.

We can further explore the concentration of ATotal and KTotal for each of these points along the curve. This is shown below for one sample point to reinforce the point that the behavior is accessible for reasonable ranges of total amounts.

$dt := plottools\text{:-}getdata(Q)$:
$L := ListTools\text{:-}Flatten(select\sim(type, [dt], Matrix))$:
$M := \langle op(L) \rangle$:

$Qdata := Matrix(ListTools\text{:-}MakeUnique(convert(M, listlist)))$:

$App\_values := Qdata[1..,1] : epsilon\_values := Qdata[1..,2] :$

We pick a value from the contour plot now to show that the resulting total amounts behind these contours (which denote biphasic peaks)

$App\_values[100] = 3.50000000000000$
$epsilon\_values[100] = 3.50910534303243$

$A_{Total} := solve(eval(ACon, \{App = App\_values[100], \text{epsilon} = epsilon\_values[100]\})) =$
27.69534456
$K2_{Total} := solve(eval(K2Con, \{App = App\_values[100], \text{epsilon} = epsilon\_values[100]\})) =$
14.91057259

DSP (common kinase and common phosphatase) - substrate biphasic

$restart : with(plots) :$

The conservation conditions describing the steady state of the system (after solving all the differential equations) are

$$ACon := \frac{k_4 c_4 c_3 App\, k_3}{\epsilon^2 k_2 c_2 k_1 c_1} + \frac{c_3 App\, k_3}{\epsilon k_2 c_2} + App + \frac{k_4 c_4 c_3 App\, k_3 P_{Total}}{k_1 \left(c_2 k_2 \left(c_3 App + 1\right) \epsilon + c_4 c_3 App\, k_3\right)} + \frac{c_2 c_3 App\, k_3 \epsilon P_{Total}}{c_2 k_2 \left(c_3 App + 1\right) \epsilon + c_4 c_3 App\, k_3} + \frac{c_3 App\, P_{Total} \epsilon k_2 c_2}{c_2 k_2 \left(c_3 App + 1\right) \epsilon + c_4 c_3 App\, k_3} + \frac{c_4 c_3 App\, k_3 P_{Total}}{c_2 k_2 \left(c_3 App + 1\right) \epsilon + c_4 c_3 App\, k_3} - A_{Total} :$$

$$KCon := \frac{\epsilon^2 P_{Total} k_2 c_2}{c_2 k_2 \left(c_3 App + 1\right) \epsilon + c_4 c_3 App\, k_3} + \frac{k_4 c_4 c_3 App\, k_3 P_{Total}}{k_1 \left(c_2 k_2 \left(c_3 App + 1\right) \epsilon + c_4 c_3 App\, k_3\right)} + \frac{c_2 c_3 App\, k_3 \epsilon P_{Total}}{c_2 k_2 \left(c_3 App + 1\right) \epsilon + c_4 c_3 App\, k_3} - K_{Total} :$$

$$T := \frac{1}{\left(App \left(\epsilon k_2 c_2 + c_4 k_3\right) c_3 + \epsilon k_2 c_2\right)^2 k_1} \left(\left(App^2 c_4 k_3 \left(k_1 k_3 - k_2 k_4\right) c_3^2 + App \left(k_1 c_2 k_2 \epsilon^2 + 2 k_3 \left(k_1 \epsilon - \frac{k_4}{2} \right) c_4 \right) k_2 c_3 + \epsilon^2 k_2^2 c_2 k_1 \right) c_2 P_{Total} \right) :$$

(5.1)

Reasonable parameters

In this sub-section, we use kinetics reported by Witzel et al., 2018 for systems involving similar phosphorylation cycles (with some variation within reasonable ranges). The kinetic parameters have units. binding constants are in the units $\mu M^{-1} s^{-1}$, while binding and catalytic constants are in the units s^{-1}. The variables and the total amounts which denote concentrations are in the units μM.

$k_{b1} := 0.18 : k_{b2} := 0.18 : k_{b3} := 0.18 : k_{b4} := 0.18 :$

$k_{ub1} := 0.27 : k_{ub2} := 0.27 : k_{ub3} := 0.27 : k_{ub4} := 0.27 :$

$k_1 := 0.0747 : k_2 := 0.0357 : k_3 := 0.0585 : k_4 := 0.315 :$

$$c_1 := \frac{k_{b1}}{k_1 + k_{ub1}} : c_2 := \frac{k_{b2}}{k_2 + k_{ub2}} : c_3 := \frac{k_{b3}}{k_3 + k_{ub3}} : c_4 := \frac{k_{b4}}{k_4 + k_{ub4}} :$$

We then assume that the total PTotal in the system is 20

$P_{Total} := 20 :$

With these parameters inputted, the expression T (which contained the necessary and sufficient condition to observe biphasic response) reduces to a bivariate expression (in epsilon and App). We can solve this to estimate the concentrations of App and epsilon where the curve has solutions.

$Q := contourplot((T), App = 0.1 ..5, \text{epsilon} = 0.1 ..2, contours = [0])$

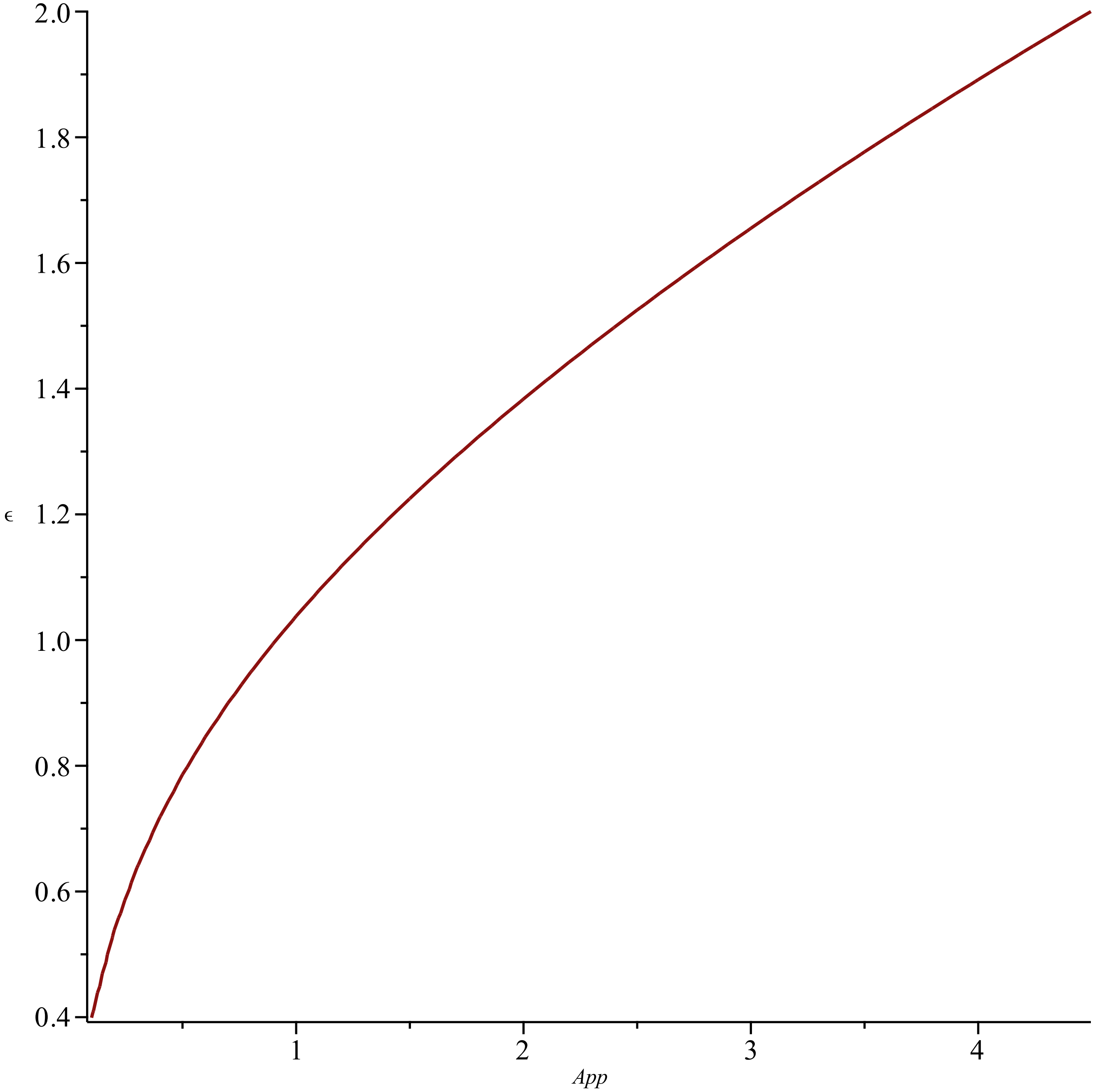

For each concentration epsilon and App along this curve, the system admits to biphasic response for the concentrations of ATotal and K2Total required, and the kinetic parameters enforced.

We can further explore the concentration of ATotal and KTotal for each of these points along the curve. This is shown below for one sample point to reinforce the point that the behavior is accessible for reasonable ranges of total amounts.

```
dt := plottools:-getdata(Q) :
L := ListTools:-Flatten(select~(type, [dt], Matrix)) :
M := ⟨op(L)⟩ :

Qdata := Matrix(ListTools:-MakeUnique(convert(M, listlist))) :
```

$App\_values := Qdata[1..,1] : epsilon\_values := Qdata[1..,2] :$

We pick a value from the contour plot now to show that the resulting total amounts behind these contours (which denote biphasic peaks)

$App\_values[100] = 1.90000000000000$
$epsilon\_values[100] = 1.35374177750885$

$A_{Total} := solve(eval(ACon, \{App = App\_values[100], \text{epsilon} = epsilon\_values[100]\})) =$
53.77183948
$K_{Total} := solve(eval(KCon, \{App = App\_values[100], \text{epsilon} = epsilon\_values[100]\})) =$
43.24075744

DSP (common kinase and common phosphatase) - enzyme biphasic

$restart : with(plots) :$

The conservation conditions describing the steady state of the system (after solving all the differential equations) are

$$ACon := \frac{k_4 c_4 c_3 App k_3}{\epsilon^2 k_2 c_2 k_1 c_1} + \frac{c_3 App k_3}{\epsilon k_2 c_2} + App + \frac{k_4 c_4 c_3 App k_3 P_{Total}}{k_1 \left(c_2 k_2 \left(c_3 App + 1\right) \epsilon + c_4 c_3 App k_3\right)} + \frac{c_2 c_3 App k_3 \epsilon P_{Total}}{c_2 k_2 \left(c_3 App + 1\right) \epsilon + c_4 c_3 App k_3} + \frac{c_3 App P_{Total} \epsilon k_2 c_2}{c_2 k_2 \left(c_3 App + 1\right) \epsilon + c_4 c_3 App k_3} + \frac{c_4 c_3 App k_3 P_{Total}}{c_2 k_2 \left(c_3 App + 1\right) \epsilon + c_4 c_3 App k_3} - A_{Total} :$$

$$KCon := \frac{\epsilon^2 P_{Total} k_2 c_2}{c_2 k_2 \left(c_3 App + 1\right) \epsilon + c_4 c_3 App k_3} + \frac{k_4 c_4 c_3 App k_3 P_{Total}}{k_1 \left(c_2 k_2 \left(c_3 App + 1\right) \epsilon + c_4 c_3 App k_3\right)} + \frac{c_2 c_3 App k_3 \epsilon P_{Total}}{c_2 k_2 \left(c_3 App + 1\right) \epsilon + c_4 c_3 App k_3} - K_{Total} :$$

$$T := App\, k_3 \Big(k_2 c_2^2 \Big(\big(App^2 c_3^2 k_1 + App \left(P_{Total} c_4 k_4 + 2 k_1\right) c_3 + P_{Total} \left(k_1 + k_4\right) c_4 + k_1\big) k_2 - App\, P_{Total} c_3 c_4 k_1 k_3\Big) c_1 \epsilon^3 + 2 k_2 \left(k_2 k_4 \left(c_3 App + 1\right) c_2 + App\, c_1 c_3 k_1 k_3\right) c_2 \left(c_3 App + 1\right) c_4 \epsilon^2 + \left(4 k_2 k_4 \left(c_3 App + 1\right) c_2 + App\, c_1 c_3 k_1 k_3\right) c_4^2 App\, k_3 c_3 \epsilon + 2 App^2 c_3^2 c_4^3 k_3^2 k_4\Big) c_3 :$$

Note that upto this point no assumption has been made about the system or the behavior

Firstly, we confirm the analytical condition to guarantee the existence of the behavior. To do so, note that the above equation is a first order polynomial in PTotal, and that for realistic (positive concentrations and kinetic parameters) values of parameters, the coefficients of all but the first exponent of PTotal are positive.

However, should the coefficient of the first exponent of the polynomial be negative, there exists real and positive solutions to the expression T (i.e., some value of App and epsilon irrespective of the kinetic parameters and total amounts otherwise); This in turn can be used to estimate (from the conservation equations) what values of total amounts the behavior could be seen at.

Thus, we explore this required and sufficient condition by isolating the coefficient of the second exponent.

$collect(T, P_{Total})$

$$App\, k_3\, k_2\, c_2^2\, \left(\left(App\, c_4\, k_4\, c_3 + (k_1 + k_4)\, c_4 \right) k_2 - App\, c_3\, c_4\, k_1\, k_3 \right) c_1\, \epsilon^3\, c_3\, P_{Total} + App\, k_3\, \left(k_2^2\, c_2^2\, \left(App^2\, c_3^2\, k_1 + 2\, App\, k_1\, c_3 + k_1 \right) c_1\, \epsilon^3 + 2\, k_2\, \left(k_2\, k_4\, (c_3\, App + 1)\, c_2 + App\, c_1\, c_3\, k_1\, k_3 \right) c_2\, (c_3\, App + 1)\, c_4\, \epsilon^2 + \left(4\, k_2\, k_4\, (c_3\, App + 1)\, c_2 + App\, c_1\, c_3\, k_1\, k_3 \right) c_4^2\, App\, k_3\, c_3\, \epsilon + 2\, App^2\, c_3^2\, c_4^3\, k_3^2\, k_4 \right) c_3 \tag{6.1}$$

Simplifying the first coefficient more, we get

$collect\left(\left(App\, c_4\, k_4\, c_3 + (k_1 + k_4)\, c_4 \right) k_2 - App\, c_3\, c_4\, k_1\, k_3, App \right) =$
$\left(-c_3\, c_4\, k_1\, k_3 + c_4\, k_4\, c_3\, k_2 \right) App + (k_1 + k_4)\, c_4\, k_2$
$simplify\left(-c_3\, c_4\, k_1\, k_3 + c_4\, k_4\, c_3\, k_2 < 0 \right) = -c_3\, c_4\, (k_1\, k_3 - k_2\, k_4) < 0$
$simplify\left(k_1\, k_3 - k_2\, k_4 > 0 \right) = 0 < k_1\, k_3 - k_2\, k_4$

Insight

1. If we ascertain the coefficient of PTotal from the expression T more carefully, we can see that should this coefficient be negative, there necessarily exists biphasic behavior (since a realistic solution for T is guaranteed) for the system at some total amounts

This coefficient is

$coeff(T, P_{Total}, 1) = App\, k_3\, k_2\, c_2^2\, \left(\left(App\, c_4\, k_4\, c_3 + (k_1 + k_4)\, c_4 \right) k_2 - App\, c_3\, c_4\, k_1\, k_3 \right) c_1\, \epsilon^3\, c_3$

We can see that this coefficient in turn is a first degree polynomial in App. Moreover irrespective of kinetic terms we can see that App needs to be greater than certain value in order for the overall coefficient to be negative. This lower bound for the concentration of App for biphasic behavior to be seen can be ascertained by solving the coefficient for App as shown below

$$solve\left(coeff(T, P_{Total}, 1), App \right)[2] = \frac{(k_1 + k_4)\, k_2}{c_3\, (k_1\, k_3 - k_2\, k_4)}$$

This implies that should this system exhibit biphasic response, there necessarily is a minimum threshold

value of App that the system will reach as more total amounts of kinase is added, until which biphasic behavior is impossible.

Beyond this limit, it follows that there is the guaranteed occurrence of biphasic response for some suitable total amounts of phosphatase, for the given App, and kinetic parameters.

2. Another insight we can ascertain through this analysis is that, since the expression T=0 (which is the necessary requirement for the presence of biphasic behavior) is of the form C1*PTotal + C0, where C1 and C0 are collection of terms involving kinetic constants, epsilon and App concentrations, with C1 can be negative, while C0 is strictly positive... A higher concentration of PTotal, allows for the behavior to be easily found in reasonable ranges of parameters and concentrations, when C1 is negative. this implies that increasing PTotal increases the propensity of obtaining biphasic behavior with some total concentrations of enzyme and substrate.

Reasonable parameters

In this sub-section, we use kinetics reported by Witzel et al., 2018 for systems involving similar phosphorylation cycles (with some variation within reasonable ranges). The kinetic parameters have units. binding constants are in the units $\mu M^{-1} s^{-1}$, while binding and catalytic constants are in the units s^{-1}. The variables and the total amounts which denote concentrations are in the units μM.

$k_{b1} := 0.18 : k_{b2} := 0.18 : k_{b3} := 0.18 : k_{b4} := 0.18 :$
$k_{ub1} := 0.27 : k_{ub2} := 0.27 : k_{ub3} := 0.27 : k_{ub4} := 0.27 :$
$k_1 := 0.0747 : k_2 := 0.0357 : k_3 := 0.0585 : k_4 := 0.0315 :$

$$c_1 := \frac{k_{b1}}{k_1 + k_{ub1}} : c_2 := \frac{k_{b2}}{k_2 + k_{ub2}} : c_3 := \frac{k_{b3}}{k_3 + k_{ub3}} : c_4 := \frac{k_{b4}}{k_4 + k_{ub4}} :$$

We then assume that the total PTotal in the system is 15

$P_{Total} := 15 :$

With these parameters inputted, the expression T (which contained the necessary and sufficient condition to observe biphasic response) reduces to a bivariate expression (in epsilon and App). We can solve this to estimate the concentrations of App and epsilon where the curve has solutions.

$Q := contourplot(\{T\}, App = 0 ..20, \text{epsilon} = 0.1 ..50, contours = [0])$

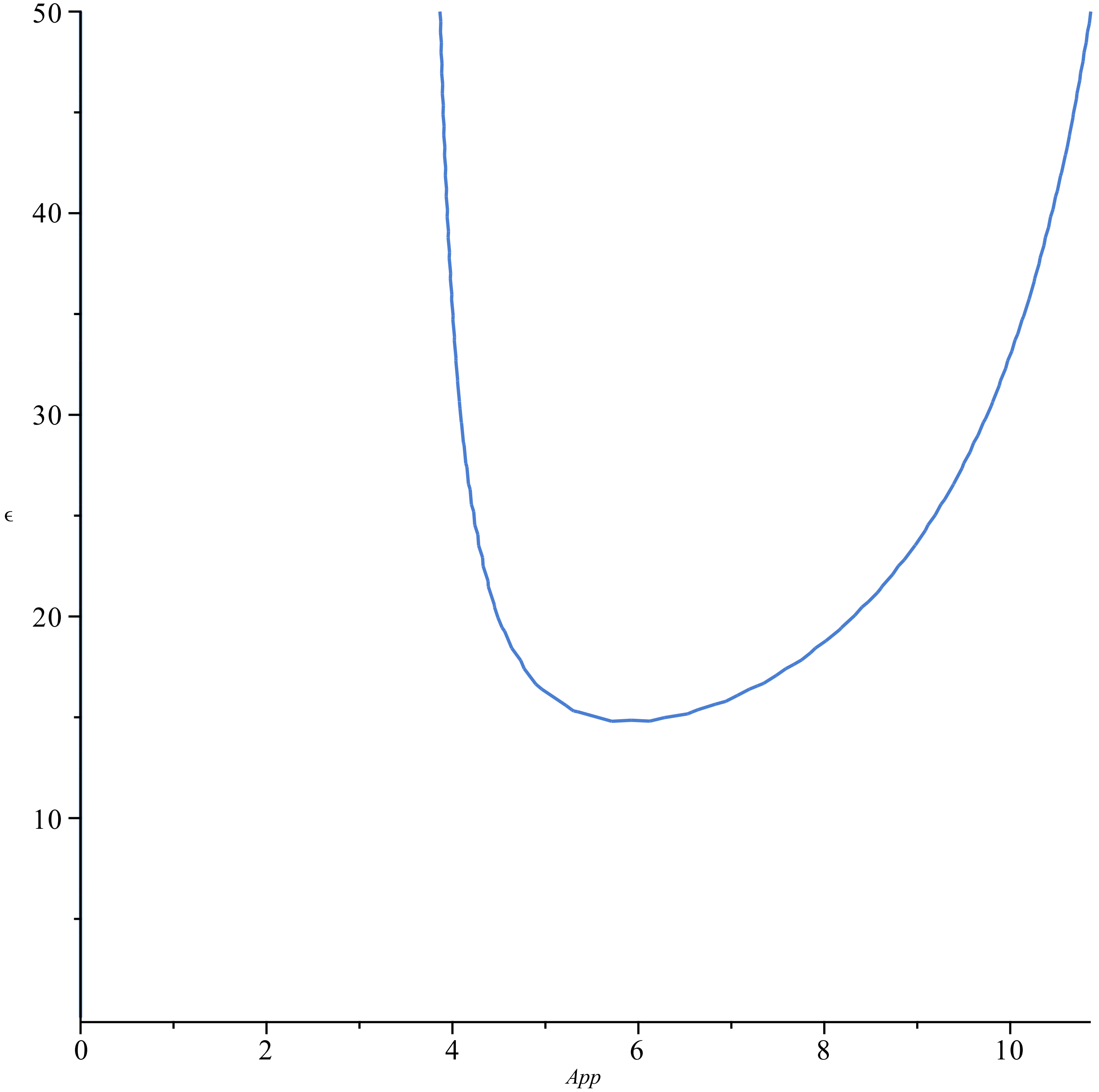

For each concentration epsilon and App along this curve, the system admits to biphasic response for the concentrations of ATotal and K2Total required, and the kinetic parameters enforced.

We can further explore the concentration of ATotal and KTotal for each of these points along the curve. This is shown below for one sample point to reinforce the point that the behavior is accessible for reasonable ranges of total amounts.

$dt := plottools\text{:-}getdata(Q)$:
$L := ListTools\text{:-}Flatten(select\sim(type, [dt], Matrix))$:
$M := \langle op(L) \rangle$:

$Qdata := Matrix(ListTools\text{:-}MakeUnique(convert(M, listlist)))$:

$App\_values := Qdata[1..,1] : epsilon\_values := Qdata[1..,2] :$

We pick a value from the contour plot now to show that the resulting total amounts behind these contours (which denote biphasic peaks)

$App\_values[100] = 4.45239553651952$
$epsilon\_values[100] = 20.4673469387755$

$A_{Total} := solve(eval(ACon, \{App = App\_values[100], \text{epsilon} = epsilon\_values[100]\}))$

$$A_{Total} := 32.49753651 \quad \textbf{(6.2.1)}$$

$K_{Total} := solve(eval(KCon, \{App = App\_values[100], \text{epsilon} = epsilon\_values[100]\}))$

$$K_{Total} := 101.2255269 \quad \textbf{(6.2.2)}$$
